# Phylogenetic and Epigenetic Footprinting of the Putative Enhancers of the *Peg3* Domain

**DOI:** 10.1371/journal.pone.0154216

**Published:** 2016-04-22

**Authors:** Joomyeong Kim, An Ye

**Affiliations:** Department of Biological Sciences, Louisiana State University, Baton Rouge, LA 70803, United States of America; McGill University, CANADA

## Abstract

The *Peg3* (Paternally Expressed Gene 3) imprinted domain is predicted to be regulated through a large number of evolutionarily conserved regions (ECRs) that are localized within its middle 200-kb region. In the current study, we characterized these potential *cis*-regulatory regions using phylogenetic and epigenetic approaches. According to the results, the majority of these ECRs are potential enhancers for the transcription of the *Peg3* domain. Also, these potential enhancers can be divided into two groups based on their histone modification and DNA methylation patterns: ubiquitous and tissue-specific enhancers. Phylogenetic and bioinformatic analyses further revealed that several *cis*-regulatory motifs are frequently associated with the ECRs, such as the E box, PITX2, NF-κB and RFX1 motifs. A series of subsequent ChIP experiments demonstrated that the trans factor MYOD indeed binds to the E box of several ECRs, further suggesting that MYOD may play significant roles in the transcriptional control of the *Peg3* domain. Overall, the current study identifies, for the first time, a set of *cis*-regulatory motifs and corresponding trans factors that may be critical for the transcriptional regulation of the *Peg3* domain.

## Introduction

*Peg3* (Paternally expressed gene 3) is an imprinted gene identified from human chromosome 19q13.4/mouse proximal chromosome 7 [1]. Mouse genetic studies have demonstrated that this gene is involved in various aspects of mammalian reproduction, including milk provision and maternal-caring behaviors [2–4]. Consistent with this, *Peg3* is highly expressed in brain, testis and ovary [1,5,6]. *Peg3* has also been known as *Pw1* as a DNA-binding transcription factor involved in myogenesis [6]. Recent studies further characterized *Peg3* as a transcriptional repressor controlling various downstream genes [7,8]. In human, *PEG3* has been often identified as a potential tumor suppressor based on the observation that its promoter is usually methylated in ovarian and breast cancers [9–11]. According to recent surveys in humans and mice, *PEG3* appears to be one of the most epigenetically unstable imprinted genes during tumorigenesis [12,13]. In fact, *in vitro* experiments demonstrated that the PEG3 protein has the potential to stop cell division in ovarian cancer cell lines [14]. The expression levels of *Peg3* are also dynamically fluctuated in response to various intrinsic and environmental cues, including nutritional starvation and hypoxic conditions [15,16]. Nevertheless, it is currently unknown how the transcription of *Peg3* is regulated to cope with various needs and challenges at the cellular and organism levels.

**Fig 1:**
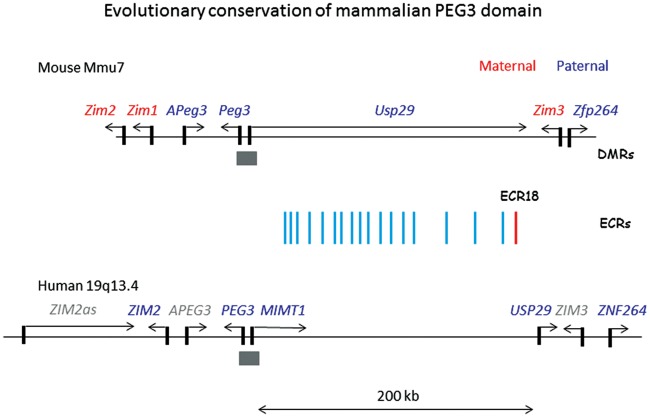
Genomic structure of mammalian *PEG3* domains. The 500-kb genomic intervals of mouse and human *PEG3* domains are represented in the following manner. Arrows indicate the transcriptional directions of imprinted genes; grey rectangles indicate the promoters with allele-specific methylation and vertical lines indicate the ECRs within the middle 200-kb genomic region. The imprinting status of some of human genes is currently unknown, thus marked with grey.

*Peg3* is the first imprinted gene identified from the 500-kb genomic interval that harbors 6 additional imprinted genes [17]. The 500-kb genomic intervals of human and mouse *Peg3* domains are well conserved in terms of gene content, orientation and distance [17] (Fig 1). This is particularly the case for the middle 200-kb interval, but this region lacks any obvious ORFs (Open Reading Frames). Instead, this interval contains 18 small genomic regions, size-ranging from 100 to 300 base pair (bp) in length, which maintain relatively high levels of sequence identity, greater than 75%, between human and mouse [18,19]. According to recent studies, these evolutionarily conserved regions (ECRs) may be potential enhancers based on their close association with two histone modification marks, H3K4me1 (monomethylation on lysine 4 of histone 3) and H3K27ac (acetylation on lysine 27 of histone 3) [19]. Interestingly, one particular ECR, ECR18, was shown to physically interact with several promoters of the *Peg3* domain [19], thus it has been hypothesized that ECR18 may play important roles as a shared enhancer for the long-range transcriptional control for the *Peg3* domain [17]. Given the observed evolutionary conservation, it is likely that the other ECRs should also play important roles for the transcription and imprinting of the *Peg3* domain.

As part of ongoing efforts, these ECRs were characterized in the current study using a series of phylogenetic and epigenetic approaches. According to the results, these ECRs have been well conserved during mammalian evolution. These putative enhancers can be further divided into two different types, ubiquitous and tissue-specific enhancers, based on their epigenetic profiles. Interestingly, two ubiquitous enhancers, ECR5 and ECR18, are shown to be epigenetically unstable during tumorigenesis. The ECRs of the *Peg3* domain are also associated with several *cis*-regulatory motifs, including the E box, PITX2, NF-κB and RFX1 motifs. A series of ChIP analyses further demonstrated that MYOD, one of the E box binding factors, indeed binds to the ECRs of the *Peg3* domain. More detailed information has been described below.

## Results

### Evolutionarily Conserved Regions (ECRs) within the Mammalian *Peg3* Domain

**Fig 2:**
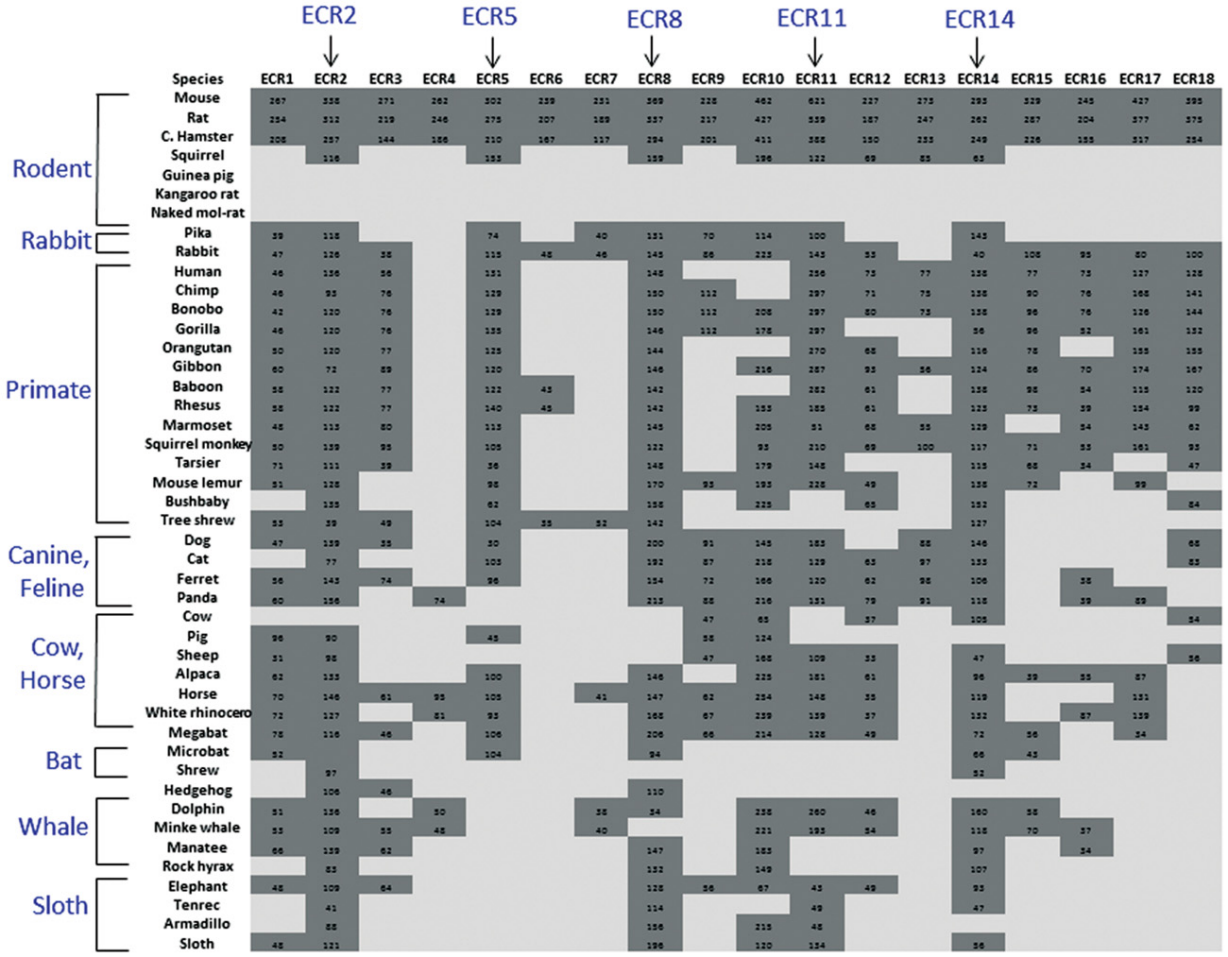
Evolutionarily Conserved Regions (ECRs) within the mammalian *Peg3* domain. This chart summarizes the outcomes of the BLAT search against 45 mammal genome sequences with a set of 18 mouse ECR sequences as probes. Each row represents one mammalian species whereas each column represents one ECR. If a given ECR from mouse detects the orthologous sequence from a given species, then this positive outcome is marked as dark grey with a numeric value derived from the BLAT search. The negative outcome is represented as light grey. Each mammalian species is grouped together based on its phylogeny. The most well conserved ECRs are also indicated with vertical arrows on top.

In the current study, we analyzed in detail the potential *cis*-regulatory regions, termed ECRs (Evolutionarily Conserved Regions), which are localized within the middle 200-kb interval of the *Peg3* domain (Fig 1). We first searched the draft genome sequences of 45 individual mammals using the BLAT program. This search used the sequences of a set of 18 ECRs derived from the mouse genome as probes [19] (S1 File). The results from this initial survey are summarized as follows. First, the ECRs of the *Peg3* domain were detected only from placental mammals, but not from the other vertebrates and mammals, such as marsupials and monotremes, indicating that the *Peg3* domain and its associated ECRs are unique to the eutherian lineage. Second, this survey identified the orthologous sequences for each ECR from many of the sequenced mammals: each ECR was confirmed to be present in the genome sequences of on average 20 out of 45 mammals (Fig 2). This result should be, however, regarded as a temporary tally, but not final outcome, since the majority of genomes have not been completely sequenced. This is the most apparent in the case of several rodents, including naked-mole rat, kangaroo rat, squirrel and guinea pig, in which we have not found any ECR so far (indicated by the light gray section on the top of Fig 2). Since the probe sequences are from the mouse, the majority of individual ECRs were also detected more frequently from the rodents than from the distantly related mammals, as shown in the dark gray section on top (rat and Chinese hamster) versus in the light gray section on bottom (hedgehog, tenrec, armadillo and sloth). Third, some of ECRs tend to be detected more frequently than the others, including ECR2, ECR5, ECR8, ECR11 and ECR14, which are represented as dark gray columns with arrows. This could be reflecting different levels of evolutionary conservation or functional constraints between individual ECRs. However, it is equally possible that some of ECRs may not be easily detectable due to their relatively short lengths, such as ECR4, ECR6 and ECR7. Thus, the conservation of these short ECRs needs to be further tested with more careful analyses in the future. Overall, this initial survey successfully identified the orthologous sequences of the 18 ECRs in many of mammalian genomes, confirming their conservation during mammalian evolution. It is also apparent that some of ECRs display greater levels of evolutionary conservation than the other ECRs, suggesting the presence of different degrees of functional constraints between individual ECRs.

### Epigenetic Profiles of ECRs

**Fig 3:**
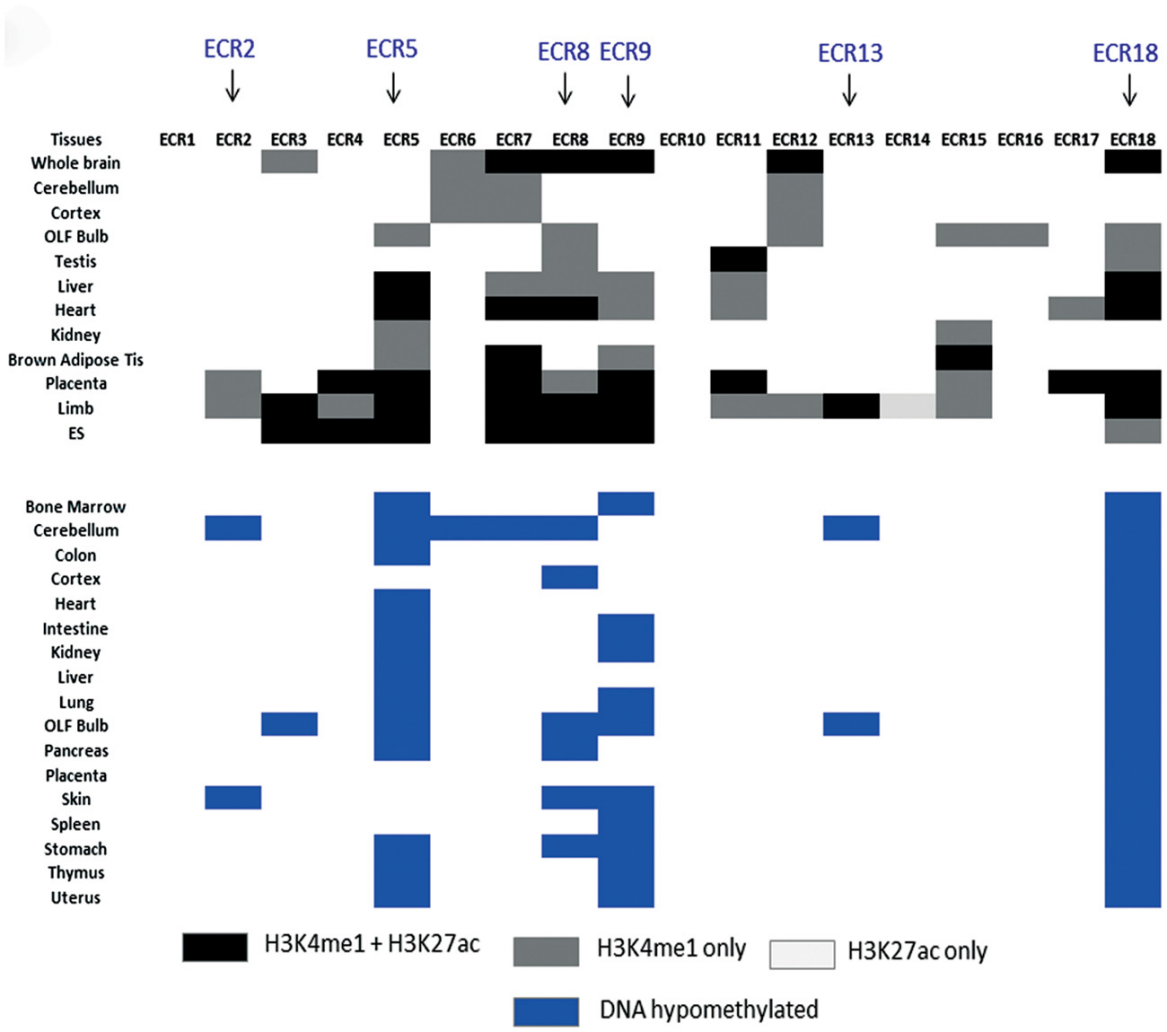
Epigenetic profiles of ECRs. The histone modifications (top) and DNA methylation patterns (bottom) are summarized using the publically available data set of the Epigenome consortium. Columns represent individual ECRs, whereas rows represent individual mouse tissues that have been used for histone and DNA modifications. Different levels of grey indicate various combinations of histone marks: dark (H3K4me1 plus H3K27ac), medium (H3K4me1 only), and light (H3K27ac only). Blue rectangles on bottom indicate hypomethylated ECRs. The ECRs showing the most histone modifications and DNA hypomethylations among tissues are indicated with arrows on top.

The epigenetic modifications of the ECRs were also analyzed using the data sets derived from the Epigenome consortium (Fig 3). This survey used two different types of epigenetic modifications: histone modifications and DNA methylation. The survey results from histone modifications indicated that the majority of the ECRs are associated with two particular histone modifications, H3K4me1 (monomethylation on lysine 4 of histone 3) and H3K27ac (acetylation on lysine 27 of histone 3), as summarized on the top section of Fig 3. These modifications are known to be associated with either poised (H3K4me1, light gray) or active enhancers (H3K4me1 and H3K27ac, dark gray) for the transcription of RNA polymerase II [20–22]. Thus, the majority of the ECRs are thought to be involved in the transcriptional control of the *Peg3* domain. According to detailed inspection, individual ECRs display different histone modification profiles. Some of the ECRs seem to show these histone modifications in the majority of the tested tissues, such as ECR5, ECR7, ECR8, ECR9 and ECR18. In contrast, the other ECRs exhibit the modifications only in one or two particular tissues, for instance ECR6 in neuronal cells and ECR14 in limb. This suggests that each ECR may function as an enhancer with a different range of tissue specificity. It is also interesting to note that the ECRs with a broader range of tissue specificity tend to be localized close to each other, for instance ECR5, ECR7, ECR8 and ECR9. These 4 ECRs are all localized within a 15-kb genomic distance. This suggests that this genomic region may play more prominent roles than the other regions in the transcriptional control of the *Peg3* domain. This prediction is further supported by the DNA methylation patterns on the ECRs as shown on the bottom of Fig 3. Four ECRs, including ECR5, ECR8, ECR9 and ECR18, display DNA hypomethylation in the majority of the tested tissues, confirming that these ECRs are likely active in a broad range of tissues. In contrast, three ECRs showed DNA hypomethylation only in a small number of tissues, for instance ECR2 in cerebellum and skin, ECR3 in olfactory bulb, and ECR13 in cerebellum and olfactory bulb. This suggests that these ECRs may act as tissue-specific enhancers. On the other hand, the remaining ECRs tend to show complete methylation in the tested tissues, indicating their relatively inactive states in the tested tissues. It is, however, possible that these ECRs may function as enhancers in very specific cell types and/or developmental stages given their histone modification profiles. Taken together, these two series of surveys indicated that the identified ECRs are likely transcriptional enhancers for the *Peg3* domain, and further that these potential enhancers can be divided into two groups: ubiquitous versus tissue-specific enhancers. In particular, the roles played by the two ECRs, ECR5 and ECR18, are predicted to be more ubiquitous than the others based on their histone and DNA methylation profiles.

### DNA Methylation Patterns of ECRs

**Fig 4:**
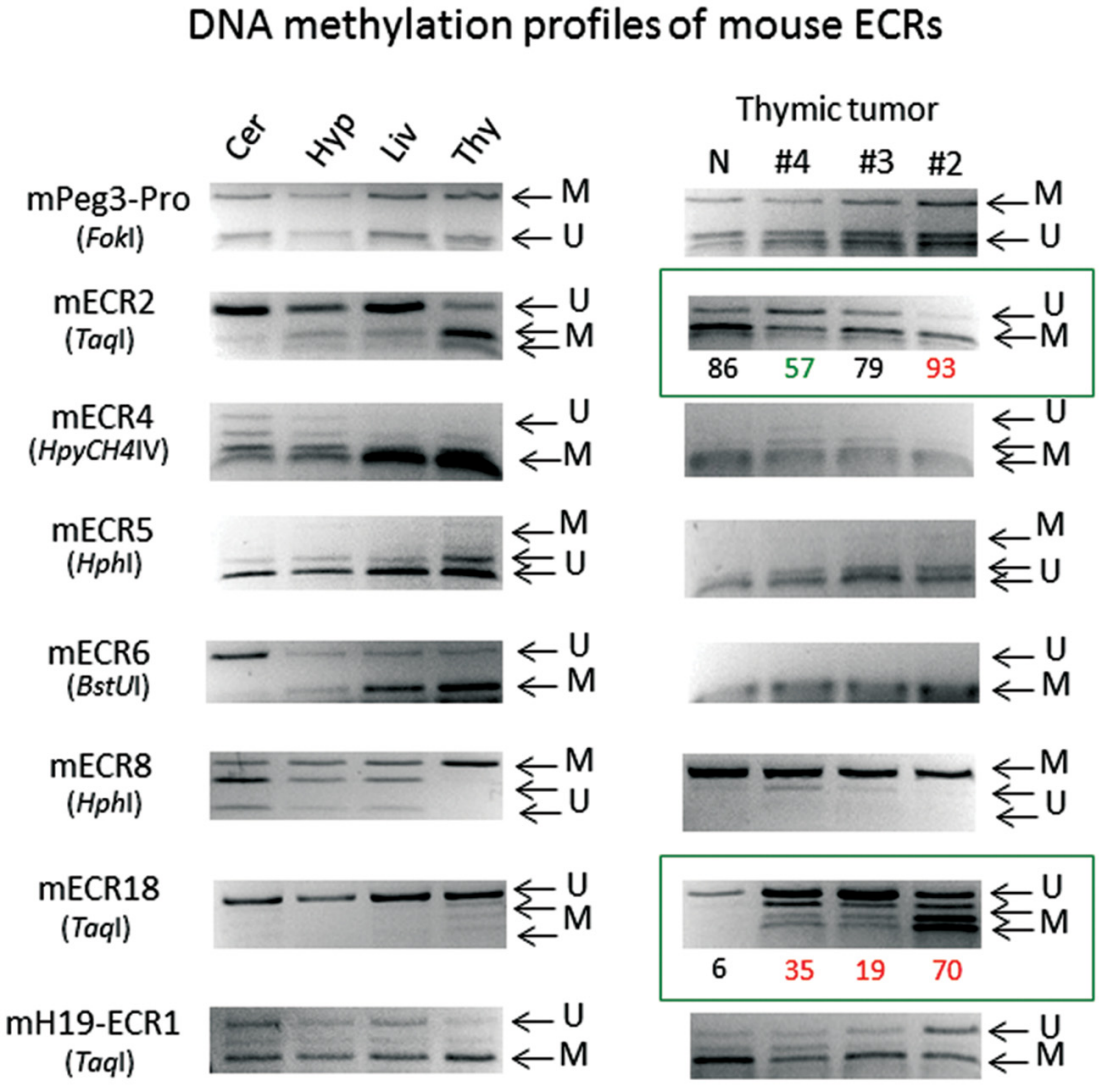
DNA methylation profiles of mouse ECRs. The bisulfite-converted DNA of mouse normal tissues (left) and thymic tumor tissues (right) were amplified, and subsequently analyzed with COBRA (Combine Bisulfite Restriction Analysis). The normal DNA was from cerebellum (Cer), hypothalamus (Hyp), liver (Liv) and thymus (Thy). The thymic DNA set on right was from normal (N), early-stage (#4 and #3) and advanced-stage tumors (#2). Each PCR product from a given ECR was digested with a restriction enzyme, resulting in a mixture of digested and undigested products. Depending upon each enzyme, the status of digestion indicates either unmethylation (U) or methylation (M) of the particular CpG site, which is part of the recognition site of the enzyme. Digestion by two enzymes, *Fok*I and *Hph*I, indicate Unmethylation since these enzymes recognize TG. In contrast, the other remaining enzymes recognize CG, thus indicating Methylation. The ECRs with boxes are the ones showing dramatic changes in their DNA methylation levels in the thymic tumor samples. DNA methylation levels of these ECRs were estimated through calculating the density of DNA bands with the ImageJ program. Compared to the levels of the normal samples, the observed changes in the thymic tumor samples are indicated with either hypomethylation (green) or hypermethylation (red).

**Fig 5:**
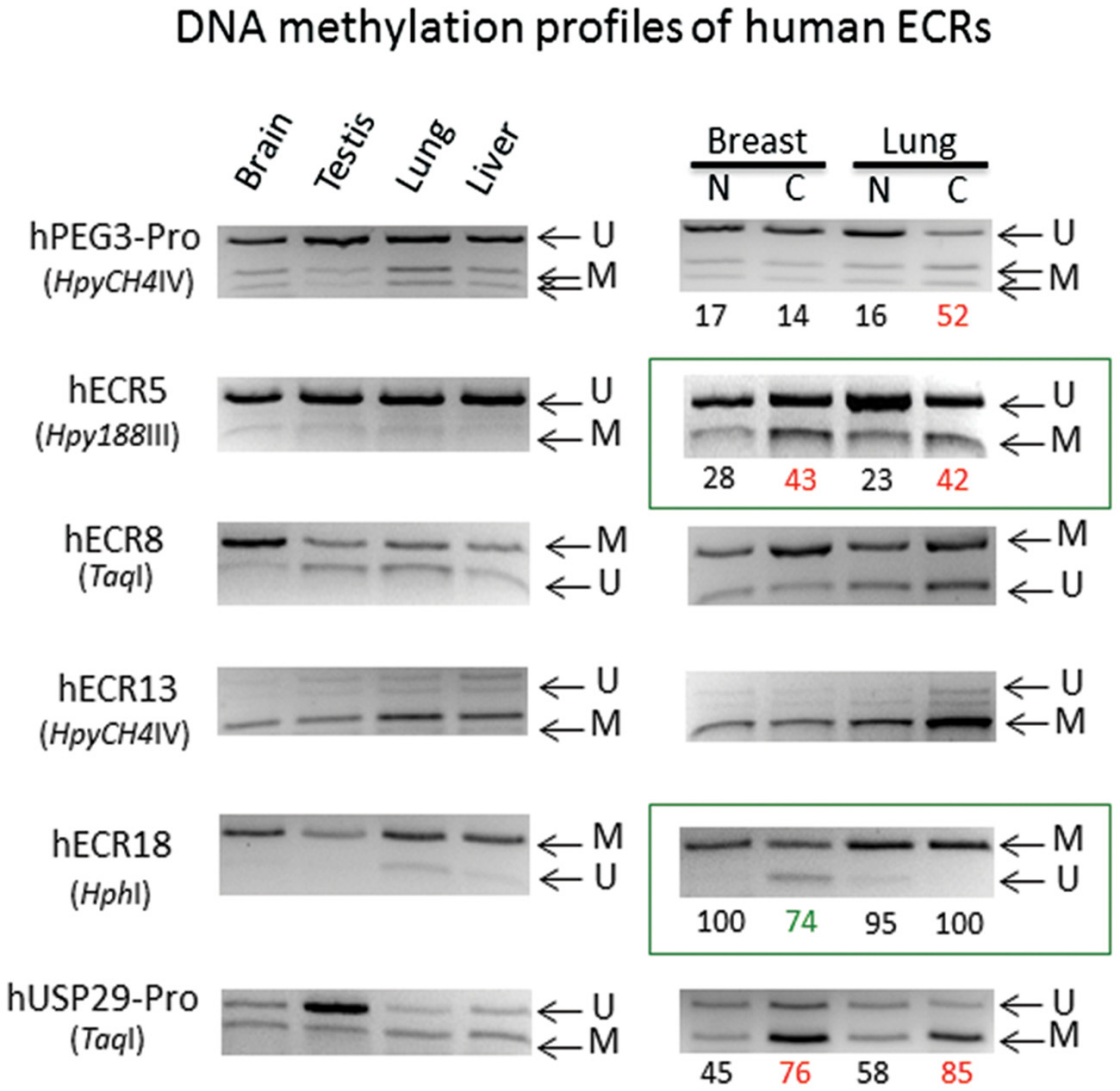
DNA methylation profiles of human ECRs. The bisulfite-converted DNA of human normal tissues (left) and matched pair sets (right) were amplified, and subsequently analyzed with COBRA (Combine Bisulfite Restriction Analysis). The normal DNA was from brain, testis, lung and liver. The matched pair sets of normal (N) and adjacent cancer (C) were from breast and lung. Each PCR product from a given ECR was digested with a restriction enzyme, resulting in a mixture of digested and undigested products. Depending upon each enzyme, the status of digestion indicates either unmethylation (U) or methylation (M) of the particular CpG site, which is part of the recognition site of the enzyme. Digestion by two enzymes, *Fok*I and *Hph*I, indicate Unmethylation since these enzymes recognize TG. In contrast, the other remaining enzymes recognize CG, thus indicating Methylation. The ECRs with boxes are the ones showing dramatic changes in their DNA methylation levels in the matched pair sets. DNA methylation levels of these ECRs were estimated through calculating the density of DNA bands with the ImageJ program. Compared to the levels of the normal samples, the observed changes in the cancer samples are indicated with either hypomethylation (green) or hypermethylation (red).

The DNA methylation patterns associated with the ECRs were further characterized using several sets of genomic DNA derived from normal and cancer samples of mouse and human (Figs 4 and 5). For this series of analyses, individual genomic DNA were first treated with the bisulfite conversion protocol, and later the converted DNA were analyzed with the restriction enzyme-based protocol COBRA (Combined Bisulfite Restriction Analysis). First, a representative set of 6 ECRs was analyzed using 4 normal tissue DNA from the mouse to test the range of tissue specificity in DNA methylation of the ECRs (left panel in Fig 4). This set of analyses also included two regions as controls, the promoter region of *Peg3* (mPeg3-Pro) and an ECR from the *H19*/*Igf2* imprinted domain (mH19-ECR1) [13]. The promoter region of *Peg3* (mPeg3-Pro) showed about 50% methylation levels in all 4 tissues, consistent with the fact that this region is methyated in an allele-specific manner [11,17]. We have also performed a set of control experiments testing the feasibility of the current approach using a series of bisulfite-converted DNAs displaying 0 to 100% methylation levels (**S2 Fig**). As expected, the results demonstrated no major bias during PCR amplification and restriction enzyme digestion. The 6 ECRs can be divided into two groups based on their DNA methylation patterns among the tested tissues. Four ECRs, ECR2, ECR4, ECR6, ECR8, displayed variable DNA methylation levels among the individual tissues. Yet, all of these ECRs tend to show lower levels of DNA methylation in cerebellum and hypothalamus than in liver and thymus. This agrees with the hypomethylation patterns observed from the data set of the Epigenome consortium (Fig 3). On the other hand, the two remaining ECRs, ECR5 and ECR18, displayed almost complete unmethylation among all the tissues tested. This is again consistent with the ubiquitous hypomethylation patterns of the two ECRs observed by the Epigenome consortium (Fig 3).

The DNA methylation analyses were also performed on a set of DNA derived from different-stage thymic tumor samples, which had been prepared through a breeding experiment involving MMTV-Cre and KrasG12D models (right panel in Fig 4) [13]. According to the results, the DNA methylation levels of the two ECRs, ECR2 and ECR18, were affected in the tumor samples. The DNA methylation levels of ECR2 were greater in the final-stage thymic tumor (#2) than those of the milder (#4 and #3) and normal (N) samples. Interestingly, the levels of one of the milder samples (#4) were lower than that of the normal sample. The methylation levels of ECR18 were also increased progressively from no methylation in the normal sample to much greater levels of methylation in the final-stage thymic tumor sample (#2). This progressive change of DNA methylation levels had become particularly obvious since ECR18 is always protected from DNA methylation among all the tissues tested. An independent control from the other imprinted domain, mH19-ECR1, also showed methylation changes in the final-stage tumor sample (#2). In this case, this potential enhancer had become hypomethylated, which is different from the hypermethylation observed from ECR2 and ECR18 of the *Peg3* domain. This indicated that the changes observed from the ECRs of the *Peg3* domain may be an outcome of specific events, but not of global DNA hypo or hypermethylation during tumorigenesis.

A similar series of analyses were also performed using two sets of human DNA (Fig 5). As expected, the promoter regions of two imprinted genes, hPEG3-Pro for *PEG3* and hUSP29-Pro for *USP29*, showed around 50% methylation levels, again consistent with the allele-specific methylation pattern of these two genes [11,17]. The methylation levels at testis were, however, lower than 50% due to the large portion of germ cells included in this tissue. The methylation patterns of human ECRs were similar to those observed from the mouse ECRs. First, two ECRs, ECR5 and ECR18, seem to show static and ubiquitous methylation patterns among the several tissues tested, which are similar to those patterns observed from the mouse counterparts. Second, the two ECRs, ECR5 and ECR18, also turn out to be affected in cancer samples. As shown in the right panel of Fig 5, ECR5 displayed DNA hypermethylation in the breast and lung cancer samples, whereas ECR18 showed hypomethylation in the breast cancer sample. The changes observed from human ECR18 are also similar to those from the mouse thymic tumor samples (Fig 4). Despite these similarities, however, the DNA methylation patterns of ECR18 appear to differ between human and mouse. Human ECR18 is mostly methylated, whereas mouse ECR18 tends to be unmethylated among all the tissues tested. The reason for this difference is currently unknown, but needs to be investigated in the near future. The methylation changes observed from human and mouse ECRs, indicated with boxes in Figs 4 and 5, were confirmed through repeating three independent trials of COBRA analyses, thus their statistical significance have been included as supporting information (S2 Table). Overall, this series of DNA methylation analyses revealed two overall patterns for the ECRs: static and ubiquitous methylation patterns for ECR5 and ECR18 and tissue-specific methylation patterns for the remaining ECRs. Interestingly, the DNA methylation levels of the two ECRs, ECR5 and ECR18, tend to be also sensitive to changes in cancer samples.

### Potential Transcription Factor Binding Sites within ECRs

**Fig 6:**
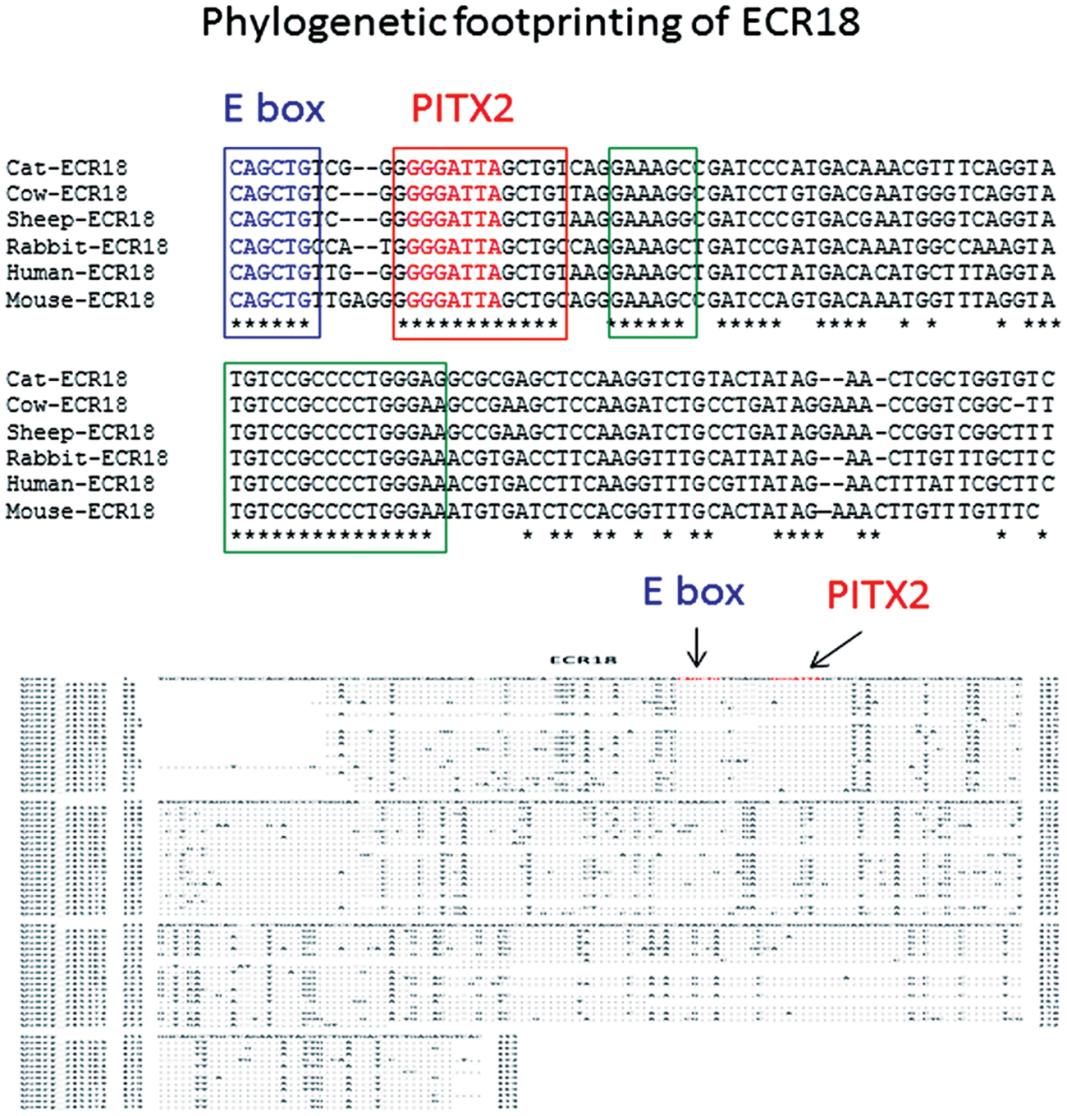
Phylogenetic footprinting of ECRs. For each ECR, the sequences from individual mammals were first aligned using the bl2seq program to identify small regions that have no sequence variations (bottom). A subset of sequences from representative mammals was also aligned using the dcode program to predict potential *cis*-regulatory motifs (top). As an example, these two alignments were derived from the set of ECR18, showing small conserved regions indicated with arrows (E box and PITX2) and also with boxes.

**Fig 7:**
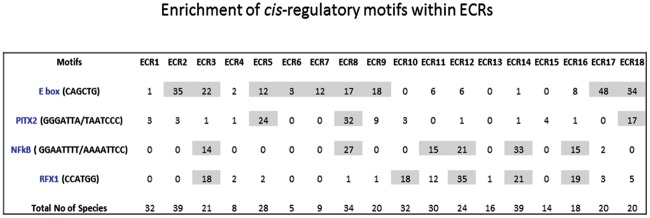
Enrichment of *cis*-regulatory motifs within ECRs. Several *cis*-regulatory motifs (represented in rows) are frequently detected within individual ECRs (represented in columns). The frequency of this detection was first summarized as numeric values in each cell, and later compared against the total number of available mammalian sequences for a given ECR. If the relative ratio of the number of the detection to the total number of available sequences for a given ECR is greater than 0.5, then that cell is marked as grey.

A series of bioinformatics analyses were performed to identify potential transcription factor binding sites within ECRs. First, as part of the phylogenetic footprining process [23,24], we aligned each of 18 sets of ECR sequences that have been derived from individual mammals using the bl2seq program (http://blast.ncbi.nlm.nih.gov/Blast.cgi?PAGE_TYPE=BlastSearch&BLAST_SPEC=blast2seq&LINK_LOC=align2seq) (S3 File). This sequence alignment was designed to identify small regions within each ECR that have been selected during mammalian evolution. For instance, the sequence alignment of the 20 sequences of ECR18 indeed confirmed the presence of several small regions with no sequence variations (bottom section of Fig 6). These small regions are most likely binding sites for unknown transcription factors. These unknown transcription factors were subsequently predicted using the dcode program (http://www.dcode.org/). According to the prediction, two sets of transcription factors can bind to ECR18. The first set of transcription factors include AP4, MYOD, E47 and Myogenin, which all share the E box motif as their core DNA-binding sites. This E box motif (CAGCTG) is well conserved among all the mammalian sequences of ECR18, as shown in the top section of Fig 6. Another transcription factor termed PITX2 can bind to a motif (GGGATTA) found within ECR18. This motif is also well conserved among ECR18 of all the mammals. This series of analyses were performed on all the remaining ECRs, and the results were summarized in the following manner (Fig 7). The frequency of each motif identified within a given ECR was represented with the number of mammalian species harboring the motif. For instance, one motif, termed the E box, was detected 34 times within the 20 mammalian sequences of ECR18, averaging 1.7 times per species.

While inspecting the results from the ECRs, we noticed that four particular motifs are frequently detected among several ECRs. These include E box (CAGCTG), PITX2 (GGGATTA/TAATCCC), NF-κB (GGAATTTT/AAAATTCC), and RFX1 (CCATGG). Each of these motifs has been identified as a conserved motif from more than three ECRs (Fig 7). This frequent sharing of these motifs among ECRs was very unexpected, and also provided an interesting pattern: a given motif tends to be shared by the ECRs that are localized close to each other. The E box motif was frequently detected among the following ECRs, ECR2 and ECR3, ECR 5 through 9, and ECR17 and ECR18. It is interesting to note that the functions of the ECR 5 through 9 have already been predicted to be more ubiquitous and prominent than the others based on their histone modification profiles (Fig 3). Yet, they may all share a common motif, the E box motif. Thus, this further suggests that unknown trans factors binding to this E box motif might play major roles in the transcriptional control for the *Peg3* domain. In the case of NF-κB and RFX1 motifs, they are found within ECR3, ECR10, ECR11, ECR12, ECR14 and ECR16 (Fig 7). In this case, interestingly, these two motifs tend to be localized together within some of the ECRs, for instance, ECR3, ECR12, ECR14 and ECR16. A similar pattern was also observed from the other two motifs, the E box and PITX2 motifs, which were detected together within ECR5, ECR8 and ECR18. Overall, this sharing of *cis*-regulatory motifs among the ECRs of the *Peg3* domain further suggests that the individual ECRs within the *Peg3* domain may be functionally related to each other.

### Identification of MYOD as a Trans Factor for the ECRs

**Fig 8:**
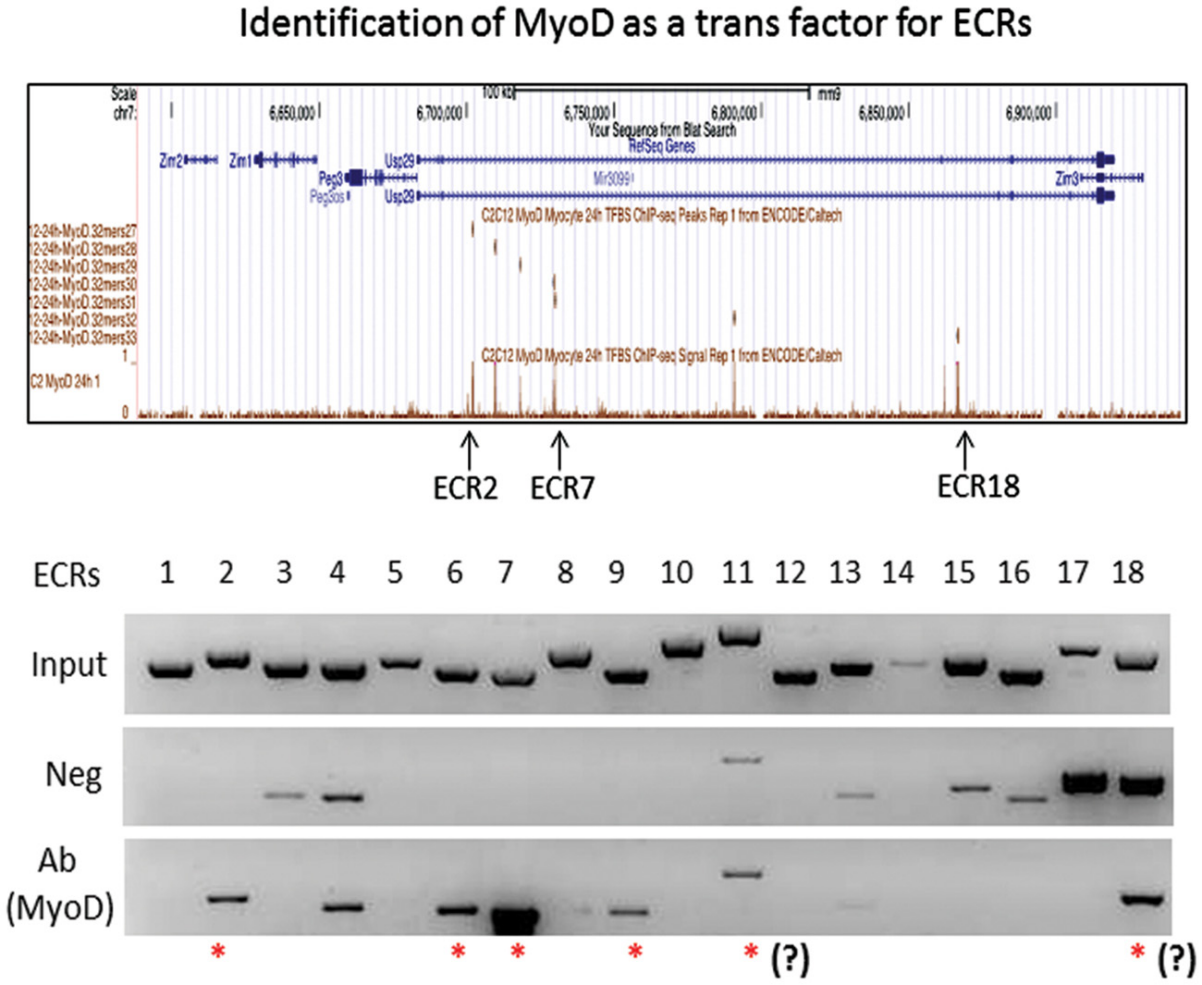
Identification of MYOD as a trans factor for ECRs. (top) A series of ChIP-seq experiments were previously performed using anti-MYOD antibody with the chromatin from C2C12 myoblast cell lines. The upper panel represents part of these ChIP-seq results belonging to the *Peg3* domain. Among several predicted peaks, three peaks were shown to overlap with the ECRs, ECR2, ECR7, and ECR18. (bottom) This panel shows the results derived from an independent series of ChIP experiment using the chromatin prepared from MEF cells. Each row represents a set of PCR targeting 18 ECRs using the Input, no antibody (Neg) and the immunoprecipitated DNA with anti MYOD antibody as templates. The ECRs with the enrichment only in Ab (MYOD) are considered to be true positives, as marked with *.

According to the prediction described above, several *cis*-regulatory motifs are quite frequently shared among the ECRs of the *Peg3* domain, such as E box and NF-κB motifs. In that regard, it is relevant to note that the E box motif is a DNA-binding site for multiple trans factors, including AP4, E47 and MYOD. In fact, the genome-wide targets of MYOD have already been identified through a series of ChIP-seq experiment using C2C12 myoblast cell line [25]. Careful inspection of this data set indeed indicated potential binding of MYOD to several ECRs, including ECR2, ECR7 and ECR18 (Fig 8). Thus, we performed a series of ChIP experiments to test potential binding of MYOD to the ECRs of the *Peg3* domain (Fig 8). For this series of analyses, we used two sets of chromatin that had been prepared from MEF and neonatal brains. These chromatins were immunoprecipitated with the same monoclonal antibody against MYOD that was used for the previous ChIP-seq experiment [25]. This series of ChIP analyses were designed to survey a set of 19 potential targets: 18 ECRs and one independent target from the *H19*/*Igf2* domain. This independent target has been chosen as a control since this target is one of the most prominent peaks in the ChIP-seq data set, and also it has three E box motifs within its 300-bp genomic region (data not shown). As expected, we were able to detect the enrichment of the anti-MYOD antibody-immunoprecipitated DNA at this control locus only from the MEF cells, but not from the neonatal brain, confirming the feasibility and specificity of our ChIP experiments (S4 File). Thus, we repeated a series of PCR-based surveys using 18 primer sets. According to the results, none of ECRs were positive with the chromatin from the neonatal brain (data not shown). On the other hand, several ECRs were indeed bound by MYOD in the MEF cells. The list of positive targets includes ECR2, ECR6, ECR7 and ECR9. This binding of MYOD to these ECRs appears to be consistent with the initial prediction since these are the ECRs with the E box motif. The other ECRs also showed some levels of the enrichment, but their enrichment levels were not that different from those detected from the negative control. This set includes ECR3, ECR4, ECR11 and ECR18. Overall, this series of ChIP experiments confirmed that the E box motif found within several ECRs is an *in vivo* target site of MYOD within the *Peg3* domain, thus further suggests that MYOD may play roles as a trans factor in the transcriptional control of the *Peg3* domain.

## Discussion

In the current study, we characterized the evolutionarily conserved regions (ECRs) of the *Peg3* domain as *cis*-regulatory regions using phylogenetic and epigenetic approaches. The results indicated that the majority of these ECRs are potential enhancers for the transcription of the *Peg3* domain. Also, these potential enhancers can be divided into two groups based on their histone modification and DNA methylation patterns: ubiquitous and tissue-specific enhancers. Phylogenetic and bioinformatic analyses further revealed that several *cis*-regulatory motifs are frequently associated with the ECRs, such as the E box, PITX2, NF-κB and RFX1 motifs. A series of subsequent ChIP experiments demonstrated that the trans factor MYOD indeed binds to the E box motif of several ECRs, thus further suggesting that MYOD may play roles in the transcription control of the *Peg3* domain. Overall, the current study identifies a set of *cis*-regulatory motifs and corresponding trans factors that may be important for the transcriptional regulation of the *Peg3* domain.

The current study provides the following insights regarding potential roles played by the ECRs of the *Peg3* domain. First, the orthologous sequences of the 18 ECRs have been successfully identified from the majority of the mammals (Fig 2). Furthermore, detailed inspection of the genomic organization of the *Peg3* domain indicated that the order, orientation and spacing of individual ECRs are also well conserved (data not shown). Thus, this structural conservation strongly suggests that these ECRs are very critical for the function of the *Peg3* domain. Second, histone modification profiles indicated that the majority of ECRs are most likely enhancers for Pol II transcription (Fig 3). The histone modification profiles also suggest that these ECRs may function as two different types of enhancers: ubiquitous and tissue-specific enhancers. This prediction is further supported by DNA methylation patterns associated with ECRs (Figs 4 and 5). The majority of ECRs tend to be hypomethylated in a tissue-specific manner, yet two of these ECRs show hypomethylation among all the tissues tested. Thus, these two ECRs, ECR5 and ECR18, may be different from the remaining ECRs. These two ubiquitous ECRs may serve as general entry sites, recruiting HAT (Histone Acetyl Transferases) and other basic machineries of Pol II transcription for the promoters and nearby ECRs. In that regard, it is important to note that the genomic interval encompassing ECR18 physically interacts with several promoters of the *Peg3* domain in brain [19]. Interestingly, ECR18 is also the one that displays the most frequent change in DNA methylation levels in both human and mouse cancers (Figs 4 and 5). This further supports an idea that ECR18 may play the most critical roles for the *Peg3* domain. On the other hand, ECR5 may also play critical roles, but in different contexts, given its close proximity to the other ECRs with neuronal and developmental specificity. For instance, several ECRs, including ECR2, ECR4, ECR6 and ECR7, are shown to be very specific in neuronal cells during early development. In that regard, it is relevant to note that a deletion encompassing these ECRs in cows is closely associated with stillbirths [26]. Also, these ECRs are very close to the two recently identified alternative promoters of *Peg3*, which exhibit early-stage specificity. For instance, ECR2 and ECR4 are located right next to these two alternative promoters, U1 and U2, respectively [27]. Overall, it is likely that the majority of the ECRs described above play important roles in the transcription of the *Peg3* domain.

According to the results, several *cis*-regulatory motifs are closely associated with the ECRs of *Peg3* domain, including the E box, PITX2, NF-κB and RFX1 motifs (Figs 6–8). The trans factors binding to these motifs are quite diverse in terms of their known physiological functions. Yet, some of these functions appear to be closely associated with the known functions of the *Peg3* locus. First, the E box motif is a DNA binding site for various basic helix-loop-helix proteins, including MYOD, BMAL1/CLOCK and MYC [28–30]. Among these proteins, MYOD has been a prime candidate given *Peg3*’s close tie to myogenesis [6,31,32]. A series of ChIP experiments indeed demonstrated that MYOD actually binds to several ECRs, including ECR2, ECR7 and ECR9 (Fig 8). Second, several ECRs also contain the binding motif for PITX2, which is a well-known factor involved in the development of heart, eye and pituitary gland [33,34]. It is relevant to note that many imprinted genes, including *Peg3*, are highly expressed in the hypothalamus and pituitary gland [35]. Thus, it might be interesting to pursue whether PITX2 is also involved in the expression of the other imprinted genes. Third, the binding motif for NF-κB is found within several ECRs. The protein complex NF-κB is involved in the signal transduction pathways that respond to various intrinsic and environmental stresses [36,37]. It is well known that *Peg3* is an immediate downstream gene of p53, and also that the expression levels of *Peg3* tend to be up-regulated in response to various stresses, such as hypoxic conditions [38,39]. The ECRs with the NF-κB motif might be the ones that control the transcriptional rate of *Peg3* in this functional context, which is also interesting to pursue in the near future. Taken together, several motifs associated with the ECRs provide very exciting directions for the future study of the *Peg3* domain.

**Fig 9:**
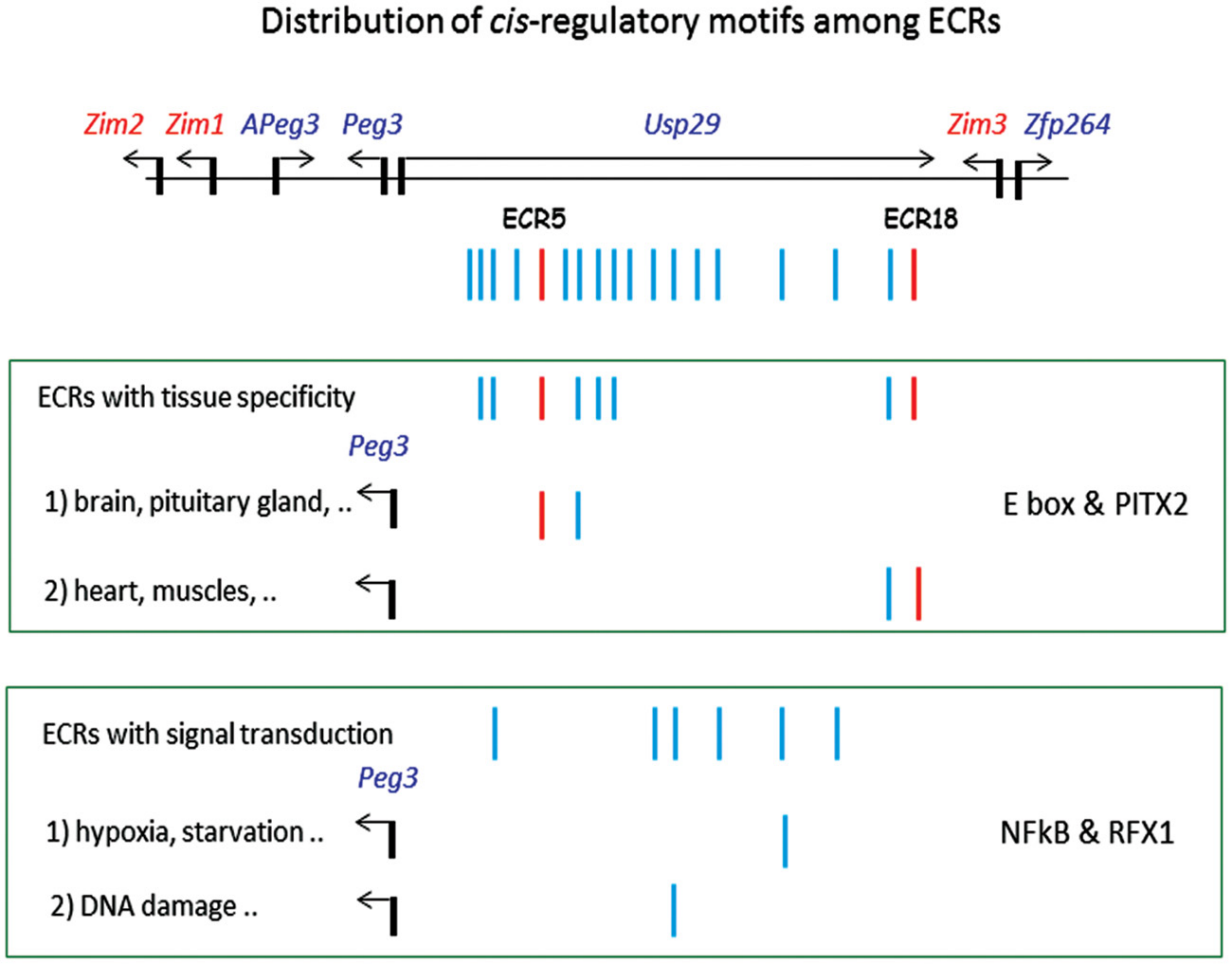
Distribution of *cis*-regulatory motifs among ECRs. The ECRs of the *Peg3* domain may play two different roles in controlling the transcription of *Peg3* and other adjacent genes. The ECRs with the E box and PITX2 motifs may be responsible for the tissue and stage-specific expression of the *Peg3* domain. On the other hand, the ECRs with NF-κB and RFX1 motifs may be responsible for modulating the transcription rate of the *Peg3* domain in response to intrinsic and environmental cues at the cellular and organism levels.

The current study also provides one intriguing observation that adjacent ECRs tend to share similar *cis*-regulatory motifs (Fig 7). The E box motif is frequently detected within the ECRs that are located close to the bidirectional promoter of *Peg3*/*Usp29*, whereas the NF-κB motif tends to be found within the ECRs distal to the promoter (Fig 9). This sharing and distribution pattern might be reflecting the evolutionary history of how a large number of ECRs have been formed for the *Peg3* domain during evolution. The upstream region of the ancestral *Peg3* locus might have only a few ECRs at the beginning, such as ECR5 and ECR18, and later, these ECRs have been duplicated to generate adjacent ECRs. These duplicated ECRs might have diverged from the original ECRs by obtaining new *cis*-regulatory motifs to adapt to new functions, such as providing slightly different spatial expression patterns for different cell populations or modulating transcription rates in response to new environmental challenges. If this is the case, the most commonly shared *cis*-regulatory motifs, such as the E box and NF-κB motifs, should be the ancestral motifs. On the other hand, it is also possible that this unusual sharing of *cis*-regulatory motifs among adjacent ECRs might have been driven by some mechanistic needs for the ECRs to function as long-range enhancers. If a group of ECRs need to work together for one promoter through a *cis*-regulatory motif, it might be easier, topologically, to have several enhancers close together than to have the enhancers scattered throughout a large genomic distance. It would be interesting to test these possibilities in the near future. Overall, the distribution pattern of several motifs clearly suggests that the ECRs located close to the bidirectional promoter might be responsible for the tissue and stage-specific expression patterns of the *Peg3* domain. On the other hand, the ECRs located in the distal region might be involved in controlling the transcription rate of the *Peg3* domain in response to environmental challenges (Fig 9).

## Materials and Methods

### Ethics Statement

All the mouse experiments were performed in accordance with National Institutes of Health guidelines for care and use of animals and also approved by the Louisiana State University Institutional Animal Care and Use Committee (IACUC), protocol #13–061.

### Bioinformatics Analyses of ECRs

A set of 18 ECRs were initially identified through comparing the 500-kb genomic sequences of human and mouse *Peg3* domains with the following criteria: any region longer than 50 bp in length and also showing greater than 75% sequence identity between human and mouse [19]. This initial set of mouse ECRs was used as probes to identify the corresponding orthologous sequences from the other mammals. This screening was performed using the BLAT program (http://genome.ucsc.edu/cgi-bin/hgBlat). The outcome of each ECR’s screening has been summarized as a table with its raw score from the BLAT search (Fig 2). For each ECR, the identified sequences were formatted as fasta files, and subsequently used for sequence alignment with the bl2seq program (http://blast.ncbi.nlm.nih.gov/Blast.cgi?PAGE_TYPE=BlastSearch&BLAST_SPEC=blast2seq&LINK_LOC=align2seq). A subset of ECR sequences was further used for predicting transcription factors using the dcode program (http://www.dcode.org/). The individual sequence and corresponding alignment files are available (S1 and S3 Files).

The histone and DNA modification profiles associated with the ECRs were obtained from the publically available data sets of the UCSC genome browser (http://genome.ucsc.edu/cgi-bin/hgGateway). The genomic interval of each ECR was individually intersected with histone modification peaks and also with DNA hypomethylated regions. This series of scoring has been initially summarized as a table, and subsequently presented as Fig 3.

### DNA Methylation Analyses of Human and Mouse ECRs

The current study used the following sets of genomic DNA for DNA methylation analyses. The mouse DNA of normal tissues was derived from one-month-old female with C57BL/6J genetic background. The DNA of thymic tumor samples was derived from several sets of offspring that had been obtained through the crossing between MMTV-Cre (Stock No. 003553, B6129-Tgn(MMTV-Cre)4Mam-LineD) and KrasG12D strains (Stock No. 08179 B6.129-Krastm4Tyj /Nci (LSL-KrasG12D)) [13]. The three strains used for this study were all obtained from the Jackson Laboratory. All the mice were housed at the DLAM (Division of Lab Animal Medicine) of LSU on a regular 12–12 dark-light cycle under a constant temperature 70°F and 50% humidity. All animals were given ad libitum access to water and Rodent Diet 5001. The nursing females were with Mouse Diet 5015. The mice with thymic tumors were monitored daily by measuring body weight, and any mice showing signs of distress or when reaching 15% weight loss were euthanized by CO2 asphixation in accordance with the rules and regulations set forth by the IACUC. Since the mice in the current study developed thymic tumors, it was not possible to monitor the size of the tumors until their necropsy. Nevertheless, the size of the harvested tumors was on average 10 mm in diameter. The human DNA of normal tissues was obtained from a commercial firm (BioChain): brain (lot# A712209), testis (lot# B104090), lung (lot# A908154) and liver (lot# A908154). The two matched pair sets of cancer DNA were also obtained from the same commercial firm (BioChain): breast (lot#B412015) and lung (lot# A811204).

Each DNA was treated with the bisulfite conversion protocol [40], and the converted DNA was subsequently used as a template for PCR amplification. The amplified product for each sample was analyzed with the restriction enzyme-based method COBRA (Combined Bisulfite Restriction Analysis) [41]. DNA methylation levels of a subset of loci were also measured using the ImageJ software as described before [12,13]. The information regarding restriction enzymes and primer sets has been included as S1 Table.

### Chromatin ImmunoPrecipitation (ChIP) Analyses

The chromatin was prepared from neonatal brains and MEF (Mouse Embryonic Fibroblast) cells according to the protocol previously described [42]. The current study used the following antibody: anti-MYOD monoclonal antibody (SantaCruz, Cat. No. sc-32758X). Potential binding of MYOD to ECRs was surveyed through performing a series of PCR targeting 18 ECRs. The information regarding the sequences and positions of the oligonucleotides is available as S1 Table.

## Supporting Information

S1 File This file contains all the ECR sequences that have been identified from 45 individual mammals. (RTF)

S2 File This file contains the results derived from a set of control experiments testing the feasibility of COBRA-based DNA methylation analyses. (PPTX)

S3 File This file contains the sequence alignment outputs that have been derived from the 18 sets of ECR sequences using the bl2seq program. (PDF)

S4 File This file contains a set of individual ChIP experiments testing the binding of MYOD to one control locus, which is located upstream of the *H19* locus. (PPTX)

S1 Table This table includes the information regarding the positions and sequences of the oligonucleotides used for COBRA and ChIP experiments. (XLSX)

S2 Table This table summarizes the DNA methylation level changes observed through COBRA analyses, which are indicated with boxes in Figs 4 and 5. (XLSX)

## References

[1] Kim J, Ashworth L, Branscomb E, Stubbs L. The human homolog of a mouse-imprinted gene, Peg3, maps to a zinc finger gene-rich region of human chromosome 19q13.4. *Genome Res* 1997;7:532–540. 9149948 10.1101/gr.7.5.532 PMC310658

[2] Li L, Keverne EB, Aparicio SA, Ishino F, Barton SC, Tada M et al Regulation of maternal behavior and offspring growth by paternally expressed Peg3. *Science* 1999;284:330–333. 10195900 10.1126/science.284.5412.330

[3] Kim J, Frey WD, He H, Kim H, Ekram MB, Bakshi A et al Peg3 mutational effects on reproduction and placenta-specific gene families. *PLoS ONE* 2013;8(12):e83359 10.1371/journal.pone.0083359 24391757 PMC3877027

[4] Frey WD, Kim J. Tissue-Specific Contributions of Paternally Expressed Gene 3 in Lactation and Maternal Care of Mus musculus. *PLoS ONE* 2015;10(12):e0144459 10.1371/journal.pone.0144459 26640945 PMC4671625

[5] Kuroiwa Y, Kaneko-Ishino T, Kagitani F, Kohda T, Li LL, Tada M et al Peg3 imprinted gene on proximal chromosome 7 encodes for a zinc finger protein. *Nat Genet* 1996;12:186–190. 8563758 10.1038/ng0296-186

[6] Relaix F, Weng X, Marazzi G, Yang E, Copeland N, Jenkins N et al Pw1, A novel zinc finger gene implicated in the myogenic and neuronal lineages. *Dev Biol* 1996;77:383–396. 10.1006/dbio.1996.0172 8806818

[7] Thiaville MM, Huang JM, Kim H, Ekram MB, Roh TY, Kim J. DNA binding motif and target genes of the imprinted transcription factor PEG3. *Gene* 2012;512:314–320. 10.1016/j.gene.2012.10.005 23078764 PMC3513644

[8] Lee S, Ye A, Kim J. DNA-binding motif of the imprinted transcription factor PEG3. *PLoS ONE* 2015;10(12):e0145531 10.1371/journal.pone.0145531 26692216 PMC4686966

[9] Dowdy SC, Gostout BS, Shridhar V, Wu X, Smith DI, Podratz KC et al Biallelic methylation and silencing of paternally expressed gene 3 (PEG3) in gynecologic cancer cell lines. *Gynecol Oncol* 2005;99:126–134. 16023706 10.1016/j.ygyno.2005.05.036

[10] Feng W, Marquez RT, Lu Z, Liu J, Lu KH, Issa JP et al Imprinted tumor suppressor genes ARHI and PEG3 are the most frequently down-regulated in human ovarian cancers by loss of heterozygosity and promoter methylation. *Cancer* 2008;112:1489–1502. 10.1002/cncr.23323 18286529

[11] Huang JM, Kim J. DNA methylation analysis of mammalian Peg3 imprinted domain. *Gene* 2009;442:18–25. 10.1016/j.gene.2009.04.016 19397955 PMC2693452

[12] Kim J, Bretz CL, Lee S. Epigenetic instability of imprinted genes in human cancers. *Nucleic Acids Res* 2015;43(22):10689–10699. 10.1093/nar/gkv867 26338779 PMC4678850

[13] Bretz CL, Langohr IM, Lee S, Kim J. Epigenetic instability at imprinting control regions in a KrasG12D-induced T-cell neoplasm. *Epigenetics* 2015; 10.1080/15592294.2015.1110672 PMC4844204 26507119

[14] Chen MY, Liao WS, Lu Z, Bornmann WG, Hennessey V, Washington MN et al Decitabine and suberoylanilide hydroxamic acid (SAHA) inhibit growth of ovarian cancer cell lines and xenografts while inducing expression of imprinted tumor suppressor genes, apoptosis, G2/M arrest, and autophagy. *Cancer* 2011;117:4424–4438. 10.1002/cncr.26073 21491416 PMC3137708

[15] Radford EJ, Isganaitis E, Jimenez-Chillaron J, Schroeder J, Molla M, Andrews S et al An unbiased assessment of the role of imprinted genes in an intergenerational model of developmental programming. *PLoS Genet* 2012;8(4):e1002605 10.1371/journal.pgen.1002605 22511876 PMC3325178

[16] Broad KD, Keverne EB. Placental protection of the fetal brain during short-term food deprivation. *Proc Natl Acad Sci USA* 2011;108(37):15237–15241. 10.1073/pnas.1106022108 21810990 PMC3174621

[17] He H, Kim J. Regulation and function of the Peg3 imprinted domain. *Genomics Inform* 2014;12(3):105–113. 10.5808/GI.2014.12.3.105 25317109 PMC4196374

[18] Kim J, Noskov VN, Lu X, Bergmann A, Ren X et al Discovery of a novel, paternally expressed ubiquitin-specific processing protease gene through comparative analysis of an imprinted region of mouse chromosome 7 and human chromosome 19q13.4. *Genome Res* 2000;10(8):1138–1147. 10958632 10.1101/gr.10.8.1138 PMC310910

[19] Thiaville MM, Kim H, Frey WD, Kim J. Identification of an evolutionarily conserved cis-regulatory element controlling the Peg3 imprinted domain. *PloS ONE* 2013;8(9):e75417 10.1371/journal.pone.0075417 24040411 PMC3769284

[20] Creyghton MP, Cheng AW, Welstead GG, Kooistra T, Carey BW et al Histone H3K27ac separates active from poised enhancers and predicts developmental state. *Proc Natl Acad Sci USA* 2010;107:21931–21936. 10.1073/pnas.1016071107 21106759 PMC3003124

[21] Shen Y, Yue F, McCleary DF, Ye Z, Edsall L, Kunan S et al (2012) A map of cis-regulatory sequences in the mouse genome. *Nature* 2012;488:116–120. 10.1038/nature11243 22763441 PMC4041622

[22] Calo E, Wysocka J. Modification of enhancer chromatin: what, how, and why? *Mol Cell* 2013;49(5):825–837. 10.1016/j.molcel.2013.01.038 23473601 PMC3857148

[23] Gumucio DL, Heilstedt-Williamson H, Gray TA, Tarlé SA, Shelton DA, Tagle DA et al Phylogenetic footprinting reveals a nuclear protein which binds to silencer sequences in the human gamma and epsilon globin genes. *Mol Cell Biol* 1992; 12(11):4919–4929. 1406669 10.1128/mcb.12.11.4919-4929.1992 PMC360424

[24] Gumucio DL, Shelton DA, Bailey WJ, Slightom JL, Goodman M. Phylogenetic footprinting reveals unexpected complexity in trans factor binding upstream from the epsilon-globin gene. *Proc Natl Acad Sci USA*. 1993;90(13):6018–6022. 8327477 10.1073/pnas.90.13.6018 PMC46858

[25] Johnson DS, Mortazavi A, Myers RM, Wold B. Genome-wide mapping of in vivo protein-DNA interactions. *Science* 2007;316(5830):1497–1502. 17540862 10.1126/science.1141319

[26] Flisikowski K, Venhoranta H, Nowacka-Woszuk J, McKay SD, Flyckt A, Taponen J et al A novel mutation in the maternally imprinted PEG3 domain results in a loss of MIMT1 expression and causes abortions and stillbirths in cattle (Bos taurus). *PLoS ONE* 2010;5(11):e15116 10.1371/journal.pone.0015116 21152099 PMC2994898

[27] Perera BP, Kim J. Next-generation sequencing-based 5' rapid amplification of cDNA ends for alternative promoters. *Anal Biochem* 2015;494:82–84. 10.1016/j.ab.2015.11.006 26617129

[28] Fong AP, Tapscott SJ. Skeletal muscle programming and re-programming. *Curr Opin Genet Dev* 2013;23(5):568–573. 10.1016/j.gde.2013.05.002 23756045 PMC3775946

[29] Koike N, Yoo SH, Huang HC, Kumar V, Lee C, Kim TK et al Transcriptional architecture and chromatin landscape of the core circadian clock in mammals. *Science* 2012;338(6105):349–354. 10.1126/science.1226339 22936566 PMC3694775

[30] Lin CY, Lovén J, Rahl PB, Paranal RM, Burge CB, Bradner JE et al Transcriptional amplification in tumor cells with elevated c-Myc. *Cell* 2012;151(1):56–67. 10.1016/j.cell.2012.08.026 23021215 PMC3462372

[31] Mitchell KJ, Pannérec A, Cadot B, Parlakian A, Besson V, Gomes ER et al Identification and characterization of a non-satellite cell muscle resident progenitor during postnatal development. *Nat Cell Biol* 2010;12(3):257–266. 10.1038/ncb2025 20118923

[32] Pannérec A, Formicola L, Besson V, Marazzi G, Sassoon DA. Defining skeletal muscle resident progenitors and their cell fate potentials. *Development* 2013;140(14):2879–2891. 10.1242/dev.089326 23739133

[33] Waite MR, Martin DM. Axial level-specific regulation of neuronal development: lessons from PITX2. *J Neurosci Res* 2015;93(2):195–198. 10.1002/jnr.23471 25124216

[34] Suh H, Gage PJ, Drouin J, Camper SA. Pitx2 is required at multiple stages of pituitary organogenesis: pituitary primordium formation and cell specification. *Development* 2002;129(2):329–337. 11807026 10.1242/dev.129.2.329

[35] Ivanova E, Kelsey G. Imprinted genes and hypothalamic function. *J Mol Endocrinol* 2011;47(2):R67–74. 10.1530/JME-11-0065 21798993

[36] Hoffmann A, Natoli G, Ghosh G. Transcriptional regulation via the NF-κB signaling module. *Oncogene* 2006;25:6706–6716. 17072323 10.1038/sj.onc.1209933

[37] Sen R, Smale ST. Selectivity of the NF-{kappa}B response. *Cold Spring Harb Perspect Biol* 2010;2(4):a000257 10.1101/cshperspect.a000257 20452937 PMC2845200

[38] Relaix F, Wei Xj, Li W, Pan J, Lin Y, Bowtell DD et al Pw1/Peg3 is a potential cell death mediator and cooperates with Siah1a in p53-mediated apoptosis. *Proc Natl Acad Sci USA* 2000;97(5):2105–2110. 10681424 10.1073/pnas.040378897 PMC15761

[39] Schwarzkopf M, Coletti D, Sassoon D, Marazzi G. Muscle cachexia is regulated by a p53-PW1/Peg3-dependent pathway. *Genes Dev* 2006;20(24):3440–3452. 17182869 10.1101/gad.412606 PMC1698450

[40] Clark SJ, Harrison J, Paul CL, Frommer M. High sensitivity mapping of methylated cytosines. *Nucleic Acids Res* 1994;22:2990–2997. 8065911 10.1093/nar/22.15.2990 PMC310266

[41] Xiong Z, Laird PW. COBRA: a sensitive and quantitative DNA methylation assay. *Nucleic Acids Res* 1997;25(12):2532–2534. 9171110 10.1093/nar/25.12.2532 PMC146738

[42] Ye A, He H, Kim J. Paternally expressed Peg3 controls maternally expressed Zim1 as a trans factor. *PloS ONE* 2014;9(9):e108596 10.1371/journal.pone.0108596 25265264 PMC4180786

