# Supplementary material for: Phylogenetic and Epigenetic Footprinting of the Putative Enhancers of the Peg3 Domain
Source: PLoS One. 2016 Apr 22;11(4):e0154216. doi: 10.1371/journal.pone.0154216 (PMC4841594; doi:10.1371/journal.pone.0154216)
Supplement: S1 File — (RTF) [file pone.0154216.s001.rtf]

>Mouse-ECR1AGAGAGGTGAGCCTTGAAAGGTTACTGTCTTTTATTCTTTTTTTTTTTAATCCCCCTCCAGGTTACAAGTTAGTGTGGATGGCAGGTCTCGGAATGAATTCCAAATCTGCCAGATGCCTGAAGTCTTCAATAACCACTGGGTACATTTCTAGGCTATTAAATTTGAAAGCCATAATGAAATGTATCATTCTGGAAAGGTAGATTCTAAGAACTTGGCAATGTCAAAGGAATGCCTTCATCAAAATGTGCTCCAAGCCCTTGTCAGCT>Human-ECR1AAGCCTTAAGAGGTATTTTTTCCCAGGTTACAAGCTAAAGGGTGGCAGAGCTAGAAATCAAACTCAAATCTGTTAGATTCCTGAGCCTCAGCCCTCAACCACCACTCAGTACATTTCTAGGCTATTAAATTTGAAAGCCATAATGAAATGGAACGTTCTTGAAAGGAAGGGAGATAATAAACACTTGGCAGACGTCAGGGGAGTACTTTCCTCAAAAAGTTCTCCAAGCCTTTGCCATTTATAGGCAACT>Alpaca-ECR1AGAAGTGACCTTTGGGAGGTATCTTGTCCCAGGTTACATGCTAGTAGATGATAGAGTCAGGAATCAAACCCAGATCTGTCAGACGCCTGAGCTCTCAACCACCACTGGGTACATTTCTAGGCTATTAAATTTGAAAGCCATAATGAAATGGATCATTCTTGAAAGGAAGGCAGATAATAAAAACTTGGCAAATGTCGAGGGAGTACTTTTCAAAAAGTGCTCTAAGCCCCTGCCATTTACCAGCAACTTTAGTCTCACTTCCAAGG>Baboon-ECR1AAGCCTTAAGAGGTATCTTTTCCCAGGTTACAAGCTAAAGGGTGGGAGAGCTAGAAATCAAGCCCAAATCTGTTAGATTCCTGAGCCTCGGCCCTCAACCACCACTAGGTACATTTCTAGGCTATTACATTTGAAAGCCATAATGAAATGGATCATTCTTGAAAGGAAGGGAGATAATAAAAACTTGGCAAATGTCAGGGGAGTACTTTCCTCAAAAAGTTCTCCAAGCCTTTGCCATTTATAGGCAACTTTCTTCACATTTCCAA>Bonobo-ECR1AAGCCTTAAGAGGTATCTTTTCCCAGGTTACAAGCTAAAGGGTGGCAGAGCTAGAAATCAAACTCAAATCTGTTAGATTCCTGAGCCTCAGCCCTCAACCACCACTCAGTACATTCTTAGGCTATTAAATTTGAAAGCCATAATGAAATGGAACGTTCTTGAAAGGAAGGGAGATAATAAAAACTTGGCAGATGTCAGGGGAGTACTTTCCTCAAAAAGTTCTCCAAGCCTTTGCCATTTATAGGCAACT>Chimp-ECR1AAGCCTTAAGAGGTATCTTTTCCCAGGTTACAAGCTAAAGGGTGGCAGAGCTAGAAATCAAACTCAAATCTGTTAGATTCCTGAGCCTCAGCCCTCAACCACCACTCAGTACATTTCTAGGCTATTAAATTTGAAAGCCATAATGAAATGGAACGTTCTTGAAAGGAAGGGAGATAATAAAAACTTGGCAGATGTCAGGGGAGTACTTTCCTCAAAAAGTTCTCCAAGCCTTTGCCATTTATAGGCAACT>Dog-ECR1TTAAGAGGTTACTTATCTCGTCCCAGGTACATGCTAGTAGGTGGTAGAGTCAGGAATCAAATCCCAATCTGTGAGATGTCTGAGCTCTAGGCTATTAATGTCTAGGCTATTAAATTTGAAAGCCATAATGAAATGTATCATTCCTGAAAGGAAGGCAGATAATAAAAACTTGGCAAATGTCAAGGGAGTACTTTCTTGAAATACTTCTCTAAGCCCTTGCCTTTTTCCGGGCAACTTTCTTTACACTTCTG>Dolphin-ECR1TGAGCATTGGGAGGTTAGTATCTTGTCCCAGGTTACATGCTAGTAGTTGGTAGAGTCAGGAATCAAACCCAAATCTGTTAGACGCCTGAGCTCTCACTATCACTGGATACATTTCTAGGCTATTAAATTTAAAAGCCATAATGAAATGGATCATTCTTGAGAGGAAGGCAGATAATAAAAACTTGGCAAATATCAAGGGAGTGCTTTCCTTAAAAAGTGCTGTAAGCCCTTGCCATTTACCGGCAACTTTCTTTTCA>Elephant-ECR1CCTGAGAGGTTAATATCTTGTCCCAGGTTCTTGCTAGCAGGTGACAGACCCAGGATCAAATTCAAATCTCTCAGATGCTTGAGCTCTCAACTACTGCTACGTACATTTTTAGGTTATTAAATTTGAAAGCCATAATGAAATGCATCATTTTTGAAAGGAAGACAGATAATAAAAACGTGGCAAATAATCAAGGGGGTGCTTTTCTCAAAAAGTGGTCTAGGTCCTTGCCATTTATGGGCAACTTTCTTCACATTTCCA>Ferret-ECR1GCAAGCCTTGAGAAGTTAGTTATCTCATCTCAGGTACATGCTAGTAGGTGGTAGAGTCAGGAATCAAACCCAAATTCGTGAGACGCCTGAACTCTCAACCACCATTGGGTACATGTCTAGGCTATTAAATTTGAAAGCCATAATGAAATATATCATTCTTGGAAGGAAGGCAGATAATAAAAATTTGGCAAATATCAAGGGAGTACTTTCTTGAAATATTTCTCTAAGCCCTTGCCTTTTTCTGGCAACTTTCTTCACACTTCC>Gibbon-ECR1ATTACCCAGGTTTTACAGATGAGAGGCAAGCCTTAAGAGGTATCTTTTCCCAGGTTACAAGCTTAAGGGTGGCAGAGCTAGAAATCAAACTCAAATCTCTTAGATTCCTGAGCCTCAGCCCTCAACCACCACTGGGTACATTTCTAGGCTATTAAATTTGAAAGCCATAATGAAATGGAACGTTCTTGAAAGGAAGGGAGATAATAAAAACTTGGCAAATGTCAGGGGAGTACTTTCCTCAAAAAGTTCTCCAAGCCTTTGCCGTTTATAGGCAAC>Gorilla-ECR1AGCCTTAAGAGATGTCTTTTCCCAGGTTACAAGCTAAAGGGTGGCAGAGCTAGAAATCAAACTCAAATCTGTTAAGATTCCTGAGCCTCAGCCCTCAACCACCACTCAGTACATTTCTAGGCTATTAAATTTGAAAGCCATAATGAAATGGAACGTTCTTGAAAGGAAGGGAGATAATAAAAACTTGGCAGATGTCAGAGGAGTACTTTCCTCAAAAAGTTCTCCAAGCCTTTGCCATTTATAGGCAACT>Horse-ECR1AACAATCAAACCCAAATCTGTCAGACAGCTGAGCTCTCAACCACCACTGGGTATATTTCTAGGCTATTAAATTGGAAAGCCATAATGAAATGGATCATTCTTGAAAGGTAGGCAGATAAAAACTTAGCAAATGTCAAGGGCGTGCTTTCTTCAAAAAGTGCTCTAAGCCCGTGCCATTTAAGGGCAACTTTTTTCATACTTCCAAGGAACA>Manatee-ECR1ATGCTATTATTATCCAGGTTTTATAGATGAGAGGGGAGCCTTGAGAGGTTAATATCTTGTCCCAGGTTACACGCTAGTAGGTGACAGATCCAGGATTAAATCCAAATCTCTCAGATGCCTGAGTTCTCAACCATTACTAAGTACATTTCTAGGCTATTAAATTTGAAAGCCATAATGAGATGCATCATTCTTGAAAAGAAGACAGATAATAAAAACATGGCAAATATCAAGGGGGTGCTTTTCTCAAAAAGTGCTCTAGGTCTTTGGCATTTATGGGCAACTTTCTTCACGCTTCC>Marmoset-ECR1CAAGCCTTCAAAGGTATCTTTTCCCAGTTACAAGCTAAAGGGTGGCAGAGCTAGAAATCAAACCCAAATCTGATGGATTCCTGAGCCTGAGCTCTCAGCCACCACTGGGTACATTTCTAGGCTATTAAATTTGAAAGCCACAATGAAATGGATCGTTCTTGAAAGGAAGAGAGAGAATAAAAACTTGGCACATGTCAGGGGAGTACTTTCCTCACAAAGTACTCCAAGCCTTTGCCATGTATAGGCAACT>Megabat-ECR1AGAGGTGAGCCTTGAGAGGTTAGTATCTTGTCCCAGGTTACATGCTACCAGGTGTTAAGTCAAGAATCAAACCCAAATTTGTCAGACACCTGAGCTCTCAACCACCACTGGGTACATTTCTAGGCTATTAAATTTGAAAGCCGTAATGAAATGGATCATTCTTGAAAGGAAGGCACATAATAAAAACTTGGCAAATGTCAAGGGAGTGCTTTCTTCAAAAAGTGTTCTAAGCCCTTGCCATTCATGGGCAATTTCCTTCACACTTCATG>Microbat-ECR1AGGCGAGCCTTGAGAGGTTGGTGTATCGTCCCAGGCTACATGCCAGTAGGTGGTAAATCAGGAATCAAACCAAATCTGTCAGACGCCTGAGCTCTCAACCACCAATGGGTACATTTGTAGGCTATTACATCTGAAAGCCATAATGAAATCGATCATTCTTGAAAGGAAGGCAGATAATAAAAACTTGGCAAATGTCAAGGGAGTGCTTTCTTCAAGAAGTGCTCTAAGCC>Minke-whale-ECR1AGTGAGCACTGGGAGGTTAGTCTCTTGTCCCAGGTTACATGCTGGTAGTTGGCAGAGTCAGGAATCAAACCCAAATCTGTTAGACGCCTGAGCTCTCACTACCACTGGATACATTTCTAGGCTATGAAATTTGAAAGCCATAATGAAATGGATCATTCTTGAGAGGAAGGCAGATAATAAAAACTTGGCAAATATCAAGGGAGTGCTTTCTTTAAAAAGTGCTCTAAGCCCTTGCCATTTACCGGCAACTTTCTTTTCA>Mouse-lemur-ECR1CCTTGAGAGGCTAGCATCTTTTCCCAGGTATAAGCTAATGGCGGCAGAGCTAGGAATCAAACCCAATCTGTCAACTCCTGAGCCTGGACTCTCAACTACTACTGGATACATTTCTAGGCCATTAAATTTGAAAGTCATAATGAAATAGATCATTCTTGAAAGGAAGGCAGATAATAAAAACTTGGCAAATGTCAAGGGAGTGCTTTCCTCAAAAAGCGCTCTAAGCCCTTGCCATTTATAGGCAACTCTCTTCACACTTCCAA>Orangutan-ECR1AGCCTTAAGAGGTATCTTTTCCCAGGTTACAAGCTAAAGGGTGGCAGAGCTAGAAATCAAACTCAAATCTGTTAGATTCCTGAGCCTCAGCCCTCAACCACCACTGGGTACATTTCTAGGCTATTAAATTTGAAAGCCATAATGAAATGGAACGTTCTTGAAAGGAAGGGAGATAATAAAAACTTGGCAGATGTCAGGGGAGTACTTTCCTCAAAAATTTCTCCAAGCCTTTGCCATTTATAGGCAACT>Panda-ECR1ATAGATCAGAGGCAAGCCTTGAGAGGTTAGTTATCTCATCCCAGGTACAAGCTAGTAAGTGGAAGAGTCAGGAATCAAACCCAAATCTGAGCTCTCAACCACCACTGGGTACATGTCTAGACTATTAAATTTGAAAGCCATAATGAAATGTATCATTCTTGAAAGGAAGGCAGATAATAAAAACCTGGCAAATGGCAAGGGAGTACTTTCTTGAAATACTTCTCTAAGCCCTTGTCTTTTTCCAGCAACTTTCTTCACACTTCTGA>Pig-ECR1AGGTTACTGTCTTGTTCCAGGTTACACGCTAGTAGATAGTAGAATCCAGAATCAAACCCAAGTCTGCCAGACACCTGAGCTCTCAAGCACCACTGGGTACATTTCTAGGCTGTTAAATTTGACAGCCATAATGAAATGGATCATTCTTGAAAGGAAGGCAGATAGTAAAAACTTGGCAAATGTCAAAGGAGTGCTTCCTTCCAAAAGTGCTCTAAGCCCTTGTCATTTAC>Pika-ECR1ACAGATGAGAAGTTAATACCTTCTCCTAGGTTATAGACTAATGGGTGGCAGAGCTAGCAGTCAAACTCAAATCTGTTATTTACATGACTGAGCTCCCAAAAACCACTGGGATACATTTCTAGGCTATTAAATTTGAAAGCCGCAAGGAAATGGATCTTTCTTGAAAGGCAGGCAGATAATAAAAGCTTGGCAAATGTCAAGGGAATACTTTCCTTAAAGCACTCAGTACTTGTGGGCTACA>Rabbit-ECR1GATGAGAAGTTAGTTTCTTTCTCTAAGTTATAAACTAATGGGTGGCAGAGCTAGGAATCAAATTCAAGTCTGTCAAAGTTATAAGCCTGAGCTCCCAACCACCACTGGAGTACATTTCTAGGCTATTAAATTTGAAAGCCACAATGAAATGGATCTTTCTTGAAAGGAAGGAAGATAATAAAAGCTTGGCAAATGTCAAGAGAATACTTTCCTAAAATGTGCTCTAAGCCTGATTCACTACTTATAGGTTA>Rat-ECR1AGAGAGGTGAGCCTTGAAAGGTTACTGTCTTTTACTCTTTTTTTTTTTTTTTAATCCCCCTCCAGGTTACAAGTTAGAGTGGATGGCAGGTCTCGGAATGAATTCCAAATCTGCCAGATGCCTGAACTCTTCAATAACCACTGGGTACATTTCTAGGCTATTAAATTTGAAAGCCATAATGAAATGTATCATTCTGGAAAGGTAGATTCTGAGAACTTGGCAATGTCAAAGGAATGCCTTCATCAAAATGGGCTCCAAGCCCTTGTCAGTT>Rhesus-ECR1AGCCTTAAGAGGTATCTTTTCCCAGGTTACAAGCTAAAGGGTGGCAGAGCTAGAAATCAAGCCCAAATCTGTTAGATTCCTGAGCCTCGGCCCTCAACCACCACTAGGTACATTTCTAGGCTATTACATTTGAAAGCCATAATGAAATGGATCATTCTTGAAAGGAAGGGAGATAATAAAAACTTGGCAAATGTCAGGGGAGTACTTTCCTCAAAAAGTTCTCCAAGCC>Sheep-ECR1GTGGGTAGATTGTCTCAGGTCACATGCTAGTGGATGGTGGTCAGGAATCAAATCCAGACCTTCGAGACGCCCAAGCTCTCACCACCAAATATACTTCTGTGCTATGAAATTTGAAAGCCATAATGAAGTGGGTCATTCTTGAGAGGAAGGCAGATAATAAAAACTTGGCAAGTATCAAGGGAGCGCTTTCTTCCGAAAGTGCCCTAAGCCCTTGCCATTTACCAGCAACTTTCTTCTCA>Sloth-ECR1TAAAATCCTGTTCCAGGTTATATGCTAGCAAATGGAAGAGCCAGGAATCAAACCCAAATCTGTCAGACTCCTAAGCCTGAGGTCTCAATCACAACTTGGCACATTTCTAGGCTATTAAATTTGAAATCCATAATGAAATGGATCATTCTTGAAATGAAGACAGATAATAAAAAACTTGGCAAATGCCAAGGGAGTGCTTTCTCCAAATAGAGCTCTAAGCCCTTGCCATTTATGGGCAACTCTCTTCACACTTC>Squirrel-monkey-ECR1CAAGCCTTCAAAGGTATCTTTTCCCAGTTACAAGCTAAAGGGTGGTAGAGCTAGAAATCAAACCCAAATCTGATGGATTCCTGAGCCTGAGCTCTCAGCCACCACTGGGTACATTTCTAGGCTATTAAATTTGAAAGCCATAATGAAATGGATCGTTCTTGAAAGGAAGAGAGATAATAAAAACTTGGCAAATGTCAGGGGAGTACTTTCCTCAAAAAGTACTCCAAGCCTTTGCCATTTATAGGCAACT>Tarsier-ECR1TCTTGAGAGGTTAGTGTCTTTTCCCATGCTACAAGCTAACAGGTGGCAGGACCAGGAATCAAACCCAAATTTGTCAGATTCTAAGCCTGAGCTCTCAACCACCACTGGGAACATTTCTGGGCTATTAATTTTTGAAAGCCATAATGAAATTGATCATTCTTGGAAAGCAGATAATGAAAACTTGGCAAATGTCAAGGGAATGTTTTCCTCAAAAGCACACTAAGCCTTCGCCGTTTATGGGCAGCTTTCT>Three-shrew-ECR1GCCTTGAGAGGTCAGCATCTTTTCCCGGGTTAAGCGAGTGGATGGTGGAACTAGGAATCAAACCAAAATCTGTCAGACTCCTGAGCCTGAACTCTCAACCACCACTGGGTAGTTTCTAAGCTATTAACTTTGAAAGCCATAATGAAACGGATCATTCTGGAAAGAAAAGCAGATATAAGCGCTGGCAAATGTCAAGGAAGTGCTCTCGTCGAAAGGTGCTCTAAGCCCTTTGTCATTATGGAAAACCTTCTTCAC>White-rhinoceros-ECR1TACTATTATTATCCAAGCTTTACAGATGAGAGGCAAGCCTTGAGGGGTTACTATCTTGTCCCAGATTACAAGCTAGTAGGTGGCAGAGTCAAGAATCAAATCCAAATCTGTCAGACACCCGAGCTCTCAACCACCACTGGGTACATTTCTAGGCTATTAAATTTGAAAGCCATAATGAAATGGATCATTCTTGAAAGCAAGGCAGATAATAAAAACTTGGCAAATGTCAAGAGAGTGCTTTCTTCAAAAAGTGCTCTAAGCCCTTGCTATTTAAGGGCAACGTTTTTCACACGTCCA>Mouse-ECR2CTGGCTGACCCCATTCTTAGGATTAAAATTTAAAACTCGAATTTAAATGTCAGGGGACTATTTTAATAAGTATCTCCCCACCCCACCCCTCAAAAGGGAGGAAAAGGCATGCCTGGAAATGGTGACTTTAGTAGGAGCAGAAACTTCCTTCCAACTTTCAAGGAACAGATAACTCATCCGTCCCACAAAGCGTGCCAAGGCCTAGAAACCAGTGGAGTTCTTCCCAGTTCGTTTTAATTAACTGGTTCACAGCGGTTTATGATCCCCGAGTGATTTTAATTGGAAGGAACTCAGCATAATGAAAATAACAACAGCTGACAGTTGCGCAATGTCCCCTC>Human-ECR2ATTTAAAACTCTTATTAAAAGGGAGGAGTAGTGCACAAAAAGTACCCCCCAACCCCCGAAAAAAGGAAGAAAAAGCATGGCCTCAAAAGGTGACTGGAGTAGGAATGGAAACTAACCTCCAACTTTCAAGGAACAAATAATGTCTCTGTTCCATAAAGTGTTCCAAGGCCTAGAAAACAGTGGAGGGCTTCCCAGTTCATTTTAATTAACTGGTTCACAGAGGTTTACAATCCCTAAGCTGTTTTAATTGGAAAGAACGCTGCAGAATGGAAATAACAACAGCTGGCAGTTATGGAACTT>Alpaca-ECR2CCCCATTCTTTAGTATTAAATTTAAAACTCCTAGTGAAGTGGAGAGGGGGTATGTAAAAGTACTCCCCAAAAGGAAGAAAAAGCATATCTTTAAAGGGCAACTGGAATAGGAATGGAAACTTCCTTCCAACTTTCAAAGAACAAATTTCTCTGTTCCACAGTGTGTTCCAAGGCCTAGAAAACAGTGGAGGGCTTCCCAGTTCCTTTTAATTGATTGGTTCACAGAGGTTTACAATCTCTAAACTGTTTTAATTGGAAGGAACTCCGCATGATGAAAATAACGACAGCTGACAGTTATAG>Baboon-ECR2TCCAACTTTCAAGGAACAAATAATGTCTCCGTTCCATAAAGTGTTCCAAGGCCTAGAAAACAGTGGAGGGCTTCCCAGTTCATTTTAATTAACTGGTTCACAGAGGTTTACAATCCCTAAGCTGTTTTAATTGGAAAGAACTCTGCAGAATGGAAATAACAACAGCTGGCAGTTATGGAACTTTCCCTATGCTGCCAGGT>Bonobo-ECR2TCCAACTTTCAAGGAACAAATAATGTCTCTGTTCCATAAAGTGTTCCAAGGCCTAGAAAACAGTGGAGGGCTTCCCAGTTCATTTTAATTAACTGGTTCACAGAGGTTTACAATCCCTAAGCTGTTTTAATTGGAAAGAACTCTGCAGAATGGAAATAACAACAGCTGGCAGTT>Bushbaby-ECR2AAATTTAAAACTCTTAGTGAAATAGAGGAAGAATGTACAAAAAGTACTCCCCCCAAAGGAAGTAAAAGTATGGTTATAGAGGGTGACTAGAGTAGGAATGGAAACTACCTTCTAACTTTCAAGGAACAAATAATTTCTCCATTGCAAAAGGTGTTCCAAGGCCTAGAAAACAGTGAAGGGCTTCCTAGTTCATTTTAATTAACTGGTTCAGAGATTAAACAATCTTTAAGCTGTTTAATTGGAAAGAACTCTGCATAATGAAAATAACAACAGCTGACAGTT>Cat-ECR2GGTGACCAGAGTAGGAACAGAAACTTCCTTCCAACTTTCAAGGAACAAGTAATTTCTCTGTTCCATAATGTGTTCCAGGGCCTAGAGAACAGTGGAGGGCTTCCCAGCTCCTTTTAATTAACTAGTTCACAGAGGTTTACAATCCCCAAGCTGTTTTAATTGGAAGGAATGCTGCAGAATGAAAATAACAACAGCTGATAGTTATGGAACTTTCTCTATGCTGCCAGGTGCTGTGTTAAGTCAAAGATGG>Chimp-ECR2AAGGAAGAAAAAGCATGGCCTCAAAAGGTGACTGGAGTAGGAATGGAAACTAACCTCCAACTTTCCAGGAACAAATAATGTCTCTGTTCCATAAAGTGTTCCAAGGCCTAGAAAACAGTGGAGGGCTTCCCAGTTCATTTTAATTAACTGGTTCACAGAGGTTTACACTCCCTAAGCTGTTTTAATTGGAAAGAACTCTGCAGAATGGAAATAACAACAGCTGGCAGTTATGGAACTTTCCCTGTGCTGCCAGGTGCTGTGGTTAAATAGAAGATTTCCATTTAATCTTCCTGACAACCAGGAAGGTAGGGTGCCATTGTTATCCAATT>Chinese-hamster-ECR2TGGCTGACCCCATTCTTAGGACTAAATTAAAAAAAAAAATCCCTTATTTAAATGTCGGGGAAGTATTCCAAAACAGGCCCCCTCCCCAAAAAGGAAAGAAAAGGCATGACTCGAAATGGTGACTTTAGTGGGAGCAGAAACTTCCTTCCAACTTTCAAGGAACAGATAATTCACCCGTTCCACAAAGCATGCCAAGGCCTAGAAACCAGTGGAGTTCTTCCCAGTTCATTTTAATTAACTGGTTCACAGAGGTTTACAATCCCCCAGTGATTTTAATTGGAAGGAACTCAACATAATGAAAATAACAACAGCTGACGGTTGTGCAATGTTCTCTCCGCTGCCAGGTACTG>Dog-ECR2GCTGACCCCATTCTCTAACATTAAATTGAAAACTCTTAGCAAAATGGAAGGAGAGAATACAAAAAGTACTTTCCAAAAAGAAGTAAAAGCATGACTTTTCAAGAGTGCCTGGAGTAGGAATGGAAACTTCCCTCCAACTTTCAAGGAACAAGTAATTTCTCTGTTCCATAATGTGTTCCAAGGCCTAGAAAACAATGGAGGGCTTCCCAGATCCTTTTAATTAACTAGTTCACAGAGGTTTACAATCCCTAAGCTATTTTAATTGGAAAGAATTCTGCATAATGAAAATAACAGCTGATA>Dolphin-ECR2CCCATTCTTTAGTATTAAATTTAAAACTCTTAGTGAAATGGAGGGGGAGTGTGTAAAAAGTACTCCCCAAAAGGAAGAAAAAGCATGGCTTTAAAGGGTGACTGGAGTAGGAATGGAAACTTCCAACTATCAAGGAACAAATTTCTCTGTTCCATAACGTGTTCCAAGGCCTCGAAAACAGTGGAGGGCTTCCCAGTTCCTTTGAATTAACTGGTTCACAGAGGTTTACAATCCCTAAGCTGTTTTAATTGGAAAGAACTTTGCATAATGAAAATAACAACAGCTGATAGTTATAGAACT>Elephant-ECR2GAAACTTCCTTCCAACTTTCAAGTGACAAATATTTCTGTTCCATAAAGCGTTCCAAGGCCTAGAAAACAGTGGATGGCTTCCCAGTTCATTTTAATTAACTGACTGGTTCACAGAGGTTTACAATACCTAAGCTGTCTTAATTGGAAAGAACCTTGCATAATGAAAAAGCACTGCAGCTGACAGTTACAGAACTTTCCCT>Ferret-ECR2CCCCATTCTCTAGTATTAAATTTAAAACTCTTAGCAAAATGAAAGGGGAGAATATAAAAAATACGTCCCAAAAGGAAGAAAAAGCATGACTTTGAAGGGTGACTGGAGTAGGAATGGGAACTTCCTTCCAACTTTCAAGGAACATGTAATTTATCTGTTCCATAACATGTTCCAAGGCCTAGGAAACAGTGGAGGGCTTCCCAACTTCTTTTAATTAACTAGTTCACAGAGGTTTACAATCCCTAAGCTGTTTTAATTGGAAAGAATTCTGCATAATGAAAATAACAACAGCTGATAGTT>Gibbon-ECR2ATGGAGGAGTAGTACACAAAAATTACTCCCCCCCACCCCCGAAAAAAGGAAGAAAAAGCATGGCCTCAAAAGGTGACTGGAGTAGGAATGGAAACTAACCTCCAACTTTCAAGGAACAAATAATGTCTGTGTTCCATAAAGTGTTCCAAGAACTAGAAAACAGTGGAGAGCTTCCCAGTTCATTTTAATAACTGGTTCACAGAGGTTTACAATCCCTAAGCTGTTTTAATTGGAAAGAACTCTGCAGAATGGAAATAACAACAGCTGGCAGTTATGGAACTTTCCCTATGCTGCCAGGTGCTGTGGTTA>Gorilla-ECR2AGAAAAAGCATGGCCTCAAAAGGTGACTGGAGTAAGAATGGAAACTAACCTCCAACTTTCAAGGAACAAATAATGTCTCTGTTCCATAAAGTGTTCCAAGGCCTAGAAAACAGTGGAGGGCTTCCCAGTTCATTTTAATTAACTGGTTCACAGAGGTTTACAATCCCTAAGCTGTTTTAATTGGAAAGAACTCTGCAGAATGGAAATAACAACAGCTGGCAGTTATGGAACTTTCCCTATGCTACCAGGTGCTGTGGTTAAATAGAAGATTTCCATGTAATCTTCCTGACAACCAGGAAG>Hedgehog-ECR2AAGAAGCACGACTTTGAATTGTGACTGCTGTAGGGATGGCAACTTCTTTTCAACTTTCAAGGAACAAATCATTTTTCTGTTCCATAATATGTTCCAAGGCCTAGCAAATAGTGAAGGGTTTCCCAGTTCCTGTTAATTAACTGGTTCATAGAGGTTTACAATCCCTAAGCTGTTTTAATTGGAATGAACTCTTCATAATGAAAATAACAACAGCTGACAGTTCTGGACCTCTCCCTATGCTGCCAGGTAG>Horse-ECR2AAATTTAAAACTCTTAGTGAAATGGAGGGGGAGTGTATAAAAAGTACACTCCAAAAGGAAGAAAAGCATGGCTTTAAAGGGTGACTGGAGTAGGAATGGAAACTTCCTTGCAACTTTCAAGGAACAAATAATTTCTCTGTTCCACAATGTGTTCCAAAACCTAGAAAACAGTGGAGGACTTTCCAGTTCCTTTTAATTAACTGGTTCACAGAGGTTTACAATCCTTAAACTGTTTTAATTGGAAAGAACTCTGCATAATGAAAATAACAACAGCTGACAGTTCTGGAATGTTCCCTCTGC>Manatee-ECR2ATTAAAATTTAAAACTCTAAGTGAAATGCAGGCGAAGTACATAAAAAAAACAAAACAAAAAATGAAGAAAAAGCATGGCTTTAAAGGGTGCCTGAAGGAAGAATGGAAACTTCCTTCCAACTTTCAAGGAACAAATCATTTCTCTGTTCCATAAAACATTCCAAGGCCTAGAAAACAGTGGAGGGCTTCCCAGTTTGTTTTAATTAACTAACTGGTTCACAGAGGTTTATAATCCCTATCTGTCTTAATTGGAAAGAACCTTGCATAATGAAAAAAACAGCAGTTGACAGTTAAGGAACT>Marmoset-ECR2AAATTTAAAACTCGTATTAAAGTGGAGGAGGAGTACACAAAAAGTACCCCCCAGAAAGGAGGAAAAAGCATTGCTCACAAGGTGACTGGAGGAGGAACGGAAACTAGCTTCCAACTTTCAAGGAACAAATAATGTCTCTTCCATAAAGTGTTCCAAGGCCTCGAAAACAGTGCAAGGCTTCCCAGTTCATTTTAATTAACTGGTTCACAGAGGTTTACAATCCCTAAGCTGTTTTAATTGGAAAGAACTCCCATAATGGAAATAACAACAGCTGGCAGTTATGGAACTTTCCCTATGCTG>Megabat-ECR2AATAAAAAGCATGACTTTAAAAGGTGACTGAAGTAGGAATGGAAATTCTCTTCCAACTTTCAAGGAACAAATAATTTCTCTGTTCCATTATGTGTTCCAAGGCCTAGAAAACAGTGGAGGGCTTCCCAGTTCCTTTTAGTTAACTGGTTCACAGAGATTTACAATCCCTAAGCTGTTTTAATTGGCAAGAACTCTGCATAATGAAAATAACAGCTGACAGTTATGGAACCCTCCCTATACTGCCAGGTG>Minke-whale-ECR2AAAGGAAGAAAAAGCATGGTTTTAAAGGGTGACTGGAGTAGGATTGGAAACTTCCAACTTTCAAGGAACAAATTTCTCTGTTCCATAACGTGTTCCAAGGCCTAAAAAACAGTGGAGGGCTTCCCAGTTCCTTTGAATTAACTGGTTCACAGAGGTTTACAATCCCTAAGCTGTTTTAATTGGAAAGAACTTTGCATAATGAAAATAGCAACAGCTGATAGTTATAGAACTAGCCCTACGATTCCAGGTG>Mouse-lemur-ECR2CCAAAAAGGAAGAAAAAGCATGACTTTAAAGGCTGACTGGCACAGAAATGGAAACTTCCTTCTAACTTTCAAGGAACAAATAATTTTTCTGTTCCATAAGGTGTTCCAAGGCCTAGAAAACAGTGGAGGGCTTCCTAGTTCATTTTAATTAACTGGTTCACAAAGGTTTACAATCTCTAAGCTGTTTTAATTGGAAAGAACTCTGCATAATGAAAATAACAACAGCTGACAGTTATGGAACTTTCTCTGT>Orangutan-ECR2AAAAAAAGCATGGCCTCGAAAGGTGACTGGAGTAGGAATGGAAACTAACCTCCAACTTTCAAGGAACAAATAATGTCTCTGTTCCATAAAGTGTTCCAAGGCCTAGAAAACAGTGGAGGGCTTCCCAGTTCATTTTAATTAACTGGTTCACAGAGGTTTACAATCCCTAAGCTGTTTTAATTGGAAAGAACTCTGCAGAATGGAAATAACAACAGCTGGCAGTTATGGAACTTTCCCTATGCTGCCAGGTGCCGTGGTTAAATAGAAGATTTCTATTTAATCTTCCTGACAACCAGGAAG>Panda-ECR2AAATTTAAAACTCTTAGCAAAATGGAAGGGGAGAATATAAAAAGTACCTCCCAAAAGGAGGAAAAAGCATGACTTTGAAGGGTGACTGGAGTAGGAATGGAAACTTCCTTCCAACTTTCAAGGAACAAGTAATTTCTCTGTTCCATAATGTGTTTCAAGGCCTAGAAAATAGTGGAGGGCTTCCCAGATCCTTTTAATTAACTAGTTCACAGAGGTTTACAACCCTAAGCTGTTTTAATTGGAAAGAATTCTGCATAATGAAAATAACAACAGCTGATAGTTATGGAACTTTCTCTATGC>Pig-ECR2AAAGAAGGAAAAAGCATGGCTTTAACTGATGACTCGAATAGGAATGGAAACTTCCAACTTTCAAGGAACAAATTTCTCTGTTCCATAATGTGTTCCAAGGCCTAGAAAACAGTGGAGAGCTTCCCAGTTCCTTTTAATTAACTGGTTCAGAGGTTTACAATCCTTATGCTCTTTTAATTTGGAAAGAACACTGCACAATGAAAATAACAACAGCTGACGGTTACAGAACTTTCTCTTTCCAGGTGCTGTG>Pika-ECR2TGCCCCACACACCAAGTGTGTGGCTTTCAGGGGTGACTGGAGGAGGAAAGGAAACTTCCTTCCAACTTTCAAGGAACAAATAATTTCTCTGTTCCTTAAAATATTCGGAGGTCTAGAAAACAGTGGAGAGCTTCCCAGATCATTTTAATTAACTGGTTCACAGAGGTTTACAATCCCTAAGCTGTTTTAATTGGAAAGGACTCTGAATAATGAAAATAATAACAGCTGACAGTTATGGAACGCGCCCCGT>Rabbit-ECR2AGTAGGAATGGAAACTTCCTTTCAACTTTCAAGGAACAAATAATTTTTCTGTTCCACAAAATATTCCAAGGTTTAGAAAACAGTGGAGAGCTTCCCAGATCATTTTAATTAACTGGTTCATAGAGGTTTACAATCCCTAAGCTGTTTTAATTGGAAAGAATTCTGCATAATGAAAATAATAACAGCTGACAGTTATGGGA>Rat-ECR2CTGGCTGACCCCATTCTTAGGATTAAAATTTAAAACTCTAATTTAAATGTCAGGGGAGTTTTTTAATAAGTAGCTCCCCACCCCTCCCCTCAAAAGGGAGGAAAAGGCATGACTCGAAATGGTGACTTTAGCAGGAGCAGAAACTTCCTTCCAACTTTCAAGGAACAGATAATTCATCCGTCCCACAAAGCGTGCCAAGGCCTAGAAACCAGTGGAGTTCTTCCCAGTTCGTTTTAATTAACTGGTTCACAGCGGTTTACGATCCCCGAGTGATTTTAATTGGAAGGAACTCAGCATAATGAAAATAACAACAGCTGACGGTTGCGCAACGTCCTCTCT>Rhesus-ECR2AGAGAAAGCATGGCCTCAAAAGGTGACTGGAGTAGGAATGGAAACTAACCTCCAACTTTCAAGGAACAAATAATGTCTCCGTTCCATAAAGTGTTCCAAGGCCTAGAAAACAGTGGAGGGCTTCCCAGTTCATTTTAATTAACTGGTTCACAGAGGTTTACAATCCCTAAGCTGTTTTAATTGGAAAGAACTCTGCAGAATGGAAATAACAACAGCTGGCAGTTATGGAACTTTCCCTATGCTGCCAGGT>Rock-hyrax-ECR2AAATTTAAAACTCTTACTGAAATGAAGTACATTAAAAAAACCAAAATAAAAAATGAAGAAAAAGCTAGGCTTACAAATGTGCCTGGAAGAAGACTGGAAATTTCCTTCCAACTTTCAAGGAATAAATCATTCCTCTGTTCCATAAAGTGTTCCAAGGCCTAGAAAACAGTGGACAGCTTCCCAGTTCATTTTAATTAACT>Sheep-ECR2CCCTCAAAGGAAGAAAAAGCATGCCTTTAAAGGGTGACTGGAGTAGGAATGGAAACTTCCTTCCAAACTTTCAAGGAACAAATTTCTATTTTCCATAACGCATTCCAAGGCCTCAAAAACAGTGGAGGACGTCCCAGTTCCTTTTAATGACTGGTTCATAAAAGTTTACAATCCCTAAGCTGTTTTAATTGGAAAGAACTTTGCATAATGAAAATAACAACAGCTGACAGTCACAGAACTTTTGATTCCA>Shrew-ECR2ATGGTCATAAACTCTACAAAAATAAAAACAAATAAAGCGACGAGGATGGGAGTAGGGAACTTCCCTTCAACTTTCAAGGAACAAATCATTTCTGTTCCATGATATGTTCCAAGGCCTAGAAAACAATGGAGGCCTTCCTAGTTCCTTTTCATTGAGCAGTTCATAGAAATTTACAATCCCTGAGCTGTTTTGATTGGAAAGAACTCTAATGAAAATCACAACAGCTGACTGGGAGGCCACGACCAATTTC>Sloth-ECR2GTGACTTGAGTAGGAATGGAAACTTCCTTAAAACTTTCAAGGAACAAGTAATTTCTCTTCCATAAAATGTTCCAAGGCATAGAAAACAGTGGAAGGTTTCCCAGTTCGTTTTAATTAACTAGTTCACAGAGGTTTACAATCCCTGAGCGGTCTTAATTGGAAAGAACCATGCATAACGAAAATAACAACAGCTGATAGTT>Squirrel-ECR2GAAACTTCCTTCCAACCTTCAAGGAACAAACACTTTCTCTGTTCCATCAAGTATGCCAAGGCCTAGAAAACAGTGGAGGGCTTCCCAGTTCGTCTTAATTAACTGGTTCACAGAGGTTTACAGTCCTGGAGCTGTTTTAATTGGAAAGAGCTCTGCACAATGGAGAAGACAACAGCTGACAGTTCCGAGCCTTCCCGGTG>Squirrel-monkey-ECR2AAATTTAAAACTTGTATTATAATGGAGGAGTACACAAAAAGTACCCCCCAAAAAGGAAGGAAAAGCATGGCCTCAAAAGGTGACTGGAGTAGGAATGGAAACTAACTTCCAACTTTCAAGGAACAAACAATGTCTCTGTTTCATAAAGTGTTCCAAGGCCTAGAAAACAGTGGAAGGCTTCCCAGTTCATTTTAATTAACTGGTTCACAGAGGTTTACAATCCCTAAGCTGTTTTAATTGGAAAGAACTCCCACAATGGAAATAACAACAGCTGGCAGTTATGGAACTTTCCCTATGCTG>Tarsier-ECR2AACTTTCAAGGAACAAATAATTTTCTGTTCCATAAAGTGTTCCAAGGCCTAGAAAATAGTGGAGGGCTTCCCAGTTCATTTTAATTAACTGGTTCACAGAGGTTTACAATCCCTAAGCTGTTTTAATTGGAAAGAATTCTGCATAATGAAAATAACAACAGCTGACAGCTATGGAACTTTCCCTATGCTGCCAGGTGCTG>Tenrec-ECR2GAGGAGTACATCAAAATCTACCCCTCACACTGAAAAAGATGCATGGCTGCAAAGGGTGCCTGGAAGAAGAGCGAAACTTCCTTCCAACTTTCAAGGAACAAATCATTTCTCTGTTCCCTGACGCGTTCCAAGTCCTTGAAAACAGTGGCG>Tree-shrew-ECR2TTGGAGGAGGAGCGTATAAAAAGCACCTCCCCCCAAAAGAAAGAAAAGTATGACTTTAAAGAACGACTGGAGTAGGAATGGAAACTTTCTTCCAACTTTCAAGGAACAAATAATTTCTCTGTTCCATAAAGTATTCCAAGGTCTAGAAAA>White-rhinoceros-ECR2GTGACTGGAGTAGAAACGAAGTTTCCTTGCAACTTTCAAGGAACAAATAATTTCTTTGTTCCACAATGTGTTCCAAGACCTAGAAAACTGTGGAGGGCTTCCCAGTTCCTTTTAATTCACTGGTTCACAGAGGTTTACAGTCCCTAAGCTGTTTTAACTGGAAAGAACTCTGCATAATAAAAATAACAACAGCTGACAAT>Mouse-ECR3CCCGAGAAGATGACGAGGACGGTATGCTGGTCAGATTCCTCTCCCACCTCCTACCTCCTCCCAGCCTGTCGAAGAGCTGGAAAATTTCAATCCCTTCCTTTCCCACTCCAGCTGCAGTTTCCATGGTAATAGAGTGAACCTGACTCTGTGGCTAACAACAGCTAACAGCTCTTGAAAGGGTCTAAGCCAGAACAATTGGAGACAACCTAAATTGGTGGGGGAAGGGAGGGCTCCCTGAATCCCTGACTAACTCACTGTTAGCTTATGCAAG>Human-ECR3ATTTATTGAGAGCTGACTGTGTGCTGCTGGTCTCGGATTTCTATTCCACATAATCTGCCTCCAGAGCTGGAAAATTCCAGTGTCTGACCATTCCTCAGCCTCCCTCCCCTTCCCCACTCCAGCTGCAGTTTCCATGGTAACAAACTGAACCTGCTTCTGTAGCTAACAAGAGCTAAGAGTTTTTGAGAGGGAATAAGCCAGAACAACTGACGTCAGCATACCCTGGCTGGGGGATGGGAAGGACTGCTTGAAGCCCTAAAGCCTTCTATTCCTCCA>Baboon-ECR3ATTTATTGAGAACTGATTGTGTGCTGCTGGTCTCGGATTTCTATTCCATATAATCTGCTTCCAGAGCTGGAAAATTCCAGTGTCTGACCATTCCTCAGCCTCCCTCCCCTTCCCCACTCCAGCTGCAGTTTCTATGGTAACAAACTGAACCTGCCTCTGTAGCTAACAAGAGCTAAGGGTTTTTGAAAGGGAATAAGCCAGAACAATTGACGTCAGCCTACCCTGGCTGGGGGATGGGAAGGACTGCTTGAAGCCCTAAAGCCTTCTATTCCTTCATCAATGGTTTATGCAAGAATTTTGTCTGTGTCA>Bonobo-ECR3ATTTATTGAGAGCTGACTGTGTGCTGCTGGTCTCGGATTTCTATTCCACATAATCTGCCTCCAGAGCTGGAAAATTCCAGTGTCTGACCATTCCTCAGCCTCCCTCCCCTTCCCCACTCCAGCTGCAGTTTCCATGGTAACAAACTGAACCTGCTTCTGTAGCTAACAAGAGCTAAGAGTTTTTGAAAGGGAATAAGCCAGAACAACTGACGTCAGCGTACCCTGGCTGGGGGATGGGAAGGACTGCTTGAAGCCCTAAAGCCTTCTATTCCTCCATCAATGGTTTATGCAAGAATTTTGTATGTG>Chinese-hamster-ECR3GGTATGCTGGTCAGATTTTCGCCCCTCCACACACAGCCTGGAGAGCTGGAAAATTTCAATCCCTGCCCATTCCTTAGCCATCCTTCCCCGAGTCCAGCTGCAGTTTCCATGGTAATAGAACGAACCTGATTCTGGCTAACAACAGCTAACAGCTCTTGAGAGGGACTAAACCAGAACAACTGGAGTCAACCTAAATTGGTGGGGGAAGGGAGCTCTCTCTGAAGCCCTAACTAACTCACTGTTGGCTTCTGCAAGAATTT>Chimp-ECR3ATTTATTGAGAGCTGACTGTGTGCTGCTGGTCTCGGATTTCTATTCCACATAATCTGCCTCCAGAGCTGGAAAATTCCAGTGTCTGACCATTCCTCAGCCTCCCTCCCCTTCCCCACTCCAGCTGCAGTTTCCATGGTAACAAACTGAACCTGCTTCTGTAGCTAACAAGAGCTAAGAGTTTTTGAAAGGGAATAAGCCAGAACAACTGACGTCAGCGTACCCTGGCTGGGGGATGGGAAGGACTGCTTGAAGCCCTAAAGCCTTCTATTCCTCCATCAATGGTTTATGCAAGAATTTTGTATGTG>Elephant-ECR3AGAATTGACTGTGTGCTGGCCTGGGATTTTGAGTTTACACACAAGTCAGCTTCTGGAGCTGGAAAATTCCAGTCTCTGCTGATTCCTCAGTCTCCCTCCTCTTTCCCACTCCAGCTGCAGTTTCCATGGCAACAGACTGAACCTGCTTGTATAGCTAACAAGAGCTAAGAGCTCCTGAAAATGAACAAGCCAGAACCATGAATATCAACCTAGCCCAGCTGGGGGATGGGGAGGATTGCCTGAAACCCTAAAGCCTCTGTTCCTCCATCAGTTTTTATGAAGACATTTATATGGGT>Ferret-ECR3TATTTACTGAGAACTGTGTACTGATTCTGCTTTTCGATTCCAAACACACACAGTCAGCCTCTGGAGCTGGAAAGTTCCAGTCCCCGCCCACTCCTCAACCTCCCTCCTTTTCCCCACTCCAGCTGCAGTTTCCATGGTAACAGACTGAACCTGCCTCTATAGCTAACAAGAGCTAAGCGCTCTTGAGGGGGAACAAGCCAGAACGAACCATTGACGTCATCCTGGCTGACTGGGGGATGGGGAGGACTGCCTGAAGCCCTAAAACCTTCTCCATCAGTGGTTGATGCAAGACTTTTCTGTGTGT>Gibbon-ECR3CATGTCAAAAATAAATAAGTAAAAAAAAAATTATTATTATTTATTGAGAGCTGACTGTGTGCTGCTGGTCTCGGATTTCTATTCCACATAATCTGCCTCCAGAGCTGGAAAATTCCAGTGTCTGACCATTCCTCAGCCTCCCTCCCCTTCCCCACTCCAGCTGCAGTTTCCATGGTAACAAACTGAACCTGCTTCTGTAGCTAACAAGAGCTAAGAGTTTTTGAAAGAGAATAAGCCAGAACAATTGACGTCAGCGTACCCTGGCTGGGGGATGGGAAGGACTGCTTGAAGCCCTAAAGCCTTCTATTCCTCCACCAATGGTTTATGCAAGAATTTTCTATGTGTCA>Gorilla-ECR3ATTTATTGAGAGCTGACTGTGTGCTGCTGGTCTCGGATTTCTATTCCACATAATCTGCCCCCAGAGCTGGAAAATTCCAGTGTCTGACCATTCCTCAGCCTCCCTCCCCTTCCCCACTCCAGCTGCAGTTTCCATGGTAACAAACTGAACCTGCTTCTGTAGCTAACAAGAGCTAAGAGTTTTTGAAAGGGAATAAGCCAGAACAACTGACGTCAGCGTACCCTGGCTGGGGGATGGGAAGGACTGCTTGAAGCCCTGAAGCCTTCTATTCCTCCATCAATGGTTTATGCAAGAATTTTCTATGTG>Hedgehog-ECR3GTCATTGAGAACTGACTGTGTGCAGGCCTGGCTTTTTGATTCTGCACACAGTTTGCCTCCAGAGCTGGAAAATTCCAGCCTCTGTCTTTCTTGCTCAGCCTCCCTCCCCTTCCCCACTCCAGCTGCAGTTTCCATGGTAACAGACTGAACCTGCCTCTATAGCTCACCAGAGCTAAGAGCTCTTGAAGGGGAACAAGCCAGAGCCATTGACGTCAACCTAGCCTGGCTGGGGGAGGGGAGAGCAGGCTGGATCTCTAA>Horse-ECR3TTTATTGAGAACTGACTGTGTGCTGGTCTGGTCTTTTGATTCCACACAGTCTGCCTCCCGAGCTGGAAAATTCCGGTCTCTTCCCATTGTTCCTCAGCCTCCCTCCCCTTCCCCACTCCAGCTGCAGTTTCCATGGTAACAGACTGAACCTGCCTCTCCAACTAACAGGAGCTAAGAGCTCTTGAAGGGGAACAGACCAGAACCATTGACGTCAGCCTAGCCTGGCTGGGGGATGGGGAGGACTGCCTGAAGCCCTAAAGCCTCCTCCATCAGTGGGTTATACAAG>Manatee-ECR3ATTTATTGAGAACTGACTGTGTGCTAGCCTGGGATTTTGAGTTTACACACAAGTCTGCTTCAGGGGCTGGAAAATGCCAGTCTCTGCCGATTCCTCAGTCTCCCTCCCCTTCCCCACTCCAGCTGCAGTTTCCATGGCAACACACTGAACCTGCCTCTATAGCTAACAAGAGCTAAGAGCTCGTGAAAATGAACAGCCTGAACCATTGACGTCAACCTAGCCTAGCTGGGGGATGGGGAGGATTGCCTGAAACCCTAAAGCCCCTGTTCCTCCATCAATAGTTTATCA>Marmoset-ECR3AACTGACTGTGTGCTGCTGGTCCAGAATTTCTTTTCCACATAATCCGCCTCCAGAGCTGGAAAATTCCAGTGTCTGACCATTCCTCGGCCTCCCCACTTCCCCACTCCAGCTGCAGTTTCCATGGTAACAAACTGAACCTGCCTCTGTAGCTAACAAGAGCTAAGAGTTTTTGAAAAGGGAATAAGCCAGAACAATTGACGTCAACCTAGCCTGGCTGGGGGATGGTGAGGACTGCTGGAAGCCCTAAAGCCTTCTATTCCTCCGTCAATGGTTTGTGCAACAATTTGTGTGCGTTCGCATCTGTCCTTGAATGGAGACTCTG>Megabat-ECR3CCACATTTTCATTCAGTCACTTCAAAAATATTTATTGAAAACTGACTCTGTGCTGGTCTGGTCTTTTGATTCTTTTGATTTCACACACAGTCTGCCTCCGGAGCTGGAAAATTCCAGTCTCTGCCCATTCCTCAGCTTCCCTCCTCTTCCCCATTCCAGCTGCAGTTTCCATGGTAACAGACTGAACCTGCATTCTATAGCTAACAAGAGCCAAAGAGCACTTGAAGAAAAGCCAGAACCATTGACATCAACCTAACCTGGTTGGGGGATGGGAAGGACTGCTACAGCCC>Minke-whale-ECR3AGTTATTGAGAATATTTTTGTGTGCTGGTCTGGTATTTTTATTCCACACACAGTCTTTGAAAATTTCAGTCTCTGCTCACTCCTTAGGCTCCCCCGCTTCCCCACTCCAGCTGCAGTTTCCATGGTAACAGACTGAACCTGCCTCTATAGCTAACTAGAGATGAGAGCTCTTGAAGGGGAACAAGCCGGAACCACTGACGTCAACCTAACCTGGCTGGGGGTTGCGGAGGCCTTCCTGAAGCCCTAAAGCCGCCTCCTTCAGTGGTTTATGAAAT>Orangutan-ECR3ATTTATTGAGAGCTGACTGTGCGCTGCTGGTCTCGGATTTCTATTCCACATAATCTGCCTCCAGAGCTGGAAAATTCCAGTGTCTGACCATTCCTCAGCCTCCCTCCCCTTCCCCACTCCAGCTGCAGTTTCTATGGTAACAAACTGAACCTGCTTCTGTAGCTAACAAGAGCTAAGAGTTTTTGAAAGGGAATAAGCCAGAACAATTGACGTCAGCATACCCTGGCTGGGGGATGGGAAGGACTGCTTGAAGCCCTAAAGCCTTCTATTCCTCCATCAATGGTTTATGCAAGAATTTTGTATGTGTCA>Rhesus-ECR3ATTTATTGAGAACTGACTGTGTGCTGCTGGTCTCGGATTTCTATTCCATATAATCTGCTTCCAGAGCTGGAAAATTCCAGTGTCTGACCATTCCTCAGCCTCCCTCCCCTTCCCCACTCCAGCTGCAGTTTCTATGGTAACAAACTGAACCTGCCTCTGTAGCTAACAAGAGCTAAGGGTTTTTGAAAGGGAATAAGCCAGAACAATTGACGTCAGCCTACCCTGGCTGGGGGATGGGAAGGACTGCTCGAAGCCCTAAAGCCTTCTATTCCTTCATCAATGGTTTATGCAAGAATTTTGTCTGTGTCA>Rat-ECR3GTATGCTGGTCAGAGTCCTCCCCCACCCTTTCACCTCCTCCCAGCCTGTCTCAAGAGCTGGAAAATTTCAATCCCTGCCCATTCCTCAGCCTTCCTTCCCCACTCCAGCTGCAGTTTCCATGGTAATAGAGTGAACCTGACTCTGCGGCTAACAACAGCTAACAGCTCTTGAAAGGGGCTAAACCAGAACAATTGGAGACAACCTAAATAGGTGGGGGGAAGGGAGGGCTCCCTGAATCCCAGACTAACTCCCCGTTAGCTTATGCAAGAA>Squirrel-monkey-ECR3CTGACTGTGTGCTTCTGGTCCAAGATTTCTATTCCACATAATCTGCCTCCAGAGCTGGAAAATTCCAGTGTCTGACCATTCCTCAGCCTCCCCGCTTCCCCACTCCAGCTGCTGTTTCCATGGCAACAGACTGAACCTGCCTCTGTAGCTAACAAGAGCTAAGAGTTTTTGAAAGGGAATAAGCCAGAACAATTGACGTCAACCTAGCCTGGCTGGGGGATGGTGAGGACTGCTTGAAGCCCTAAAGCCTTCTATTCCTCCGTCAATGGT>Tree-shrew-ECR3TAACTTGTCCAAGTTCTCGTGTTCTCCTTTAAGTTATCCTGAAAAATACTAATGGAGAACTGACTTTGTGCGGGTTGGAATTTCCACACATTTTGCCTCCAGAGCTGGAAAATTTCAATCTCTGCCCATTCCTCGGCCTCCCTCCCTTTCCCCACTCCAGCTGAGGTTTCCATGGTAACAGACTGACCCTGCCACTCAAGCTAACAAGAGCTAAAAGCTTCTGATGGGGAATCATCCAAAACAATTTACTGCTTAGGTGAGGGGTGGGGAGAACACCC>Mouse-ECR4TCGCCTCGGTGGACACGCTTCTGCAAGCCAAACAATTGCTTTGCTTTGGATAGCCGGGCCCATCAAAGATGGACTCAGTCTGATAAACACGCCTCTGCATGCCAACCAATCCAGCGGGGTCTCACTCCAGAGACCACACTTCTGCAGATGACGTTAATGTCAGTCCTTTTCTGTCTTTGAAAGCTGGACAGCACCTGTCCTCTGCTGCCCCCAGACCCTTCATAGAACAGTACCATGCTTCAGAGAGACTCCGGATTAGCCT>Chinese—hamster-ECR4TCGCCTCAGTGGACACGCTTCTGCAAGCCAAACAATTGCTTTGCTTTGGAGAGCCGAGCCCATCAAAGATGGACGCAGTCTGGTAAACACGCCTCTGCATGCCAACCAATCCAGAGGGGTCTCACTCCAGAGACCACACTTCTGCAGATGACGTCAATGTCAATCCCTTTCTGCCTTTGAAAGCAAAGCGGGTCAGCACTCGTCCTCCGCTGCCCCCAGACCCCTGTAAAACAGTCCCAAGCTTCACAGAGACCCCTGACTAGCCTAGCT>Dolphin-ECR4CCATGGCAACTTGCCTCAGAGAACGCGCTTTTGCGAGCCAAACAATACGTGTCACCACTGTGCTCTGGAAAGTCAGCCAGGGAAAGAGCCTATCAAGGACGGACTCACTCTGATAAACACGCCTCTGGGTGCCAACCAATCAACATGGGTCTCACTTGAGAGACCACAGTCCTACAGACAACGTGAAGCTGTTTATGCCTCCAAAGGCCCGCCAACCTCCGAAAGCCAAACCCTGCATGAAATCAGTCCATTCTGTGCAGAGAGACTG>Horse-ECR4TCAGTTTTAAACCTGTTCAACTGATCAATGACAGCTCGCCTCAGCAAACCCGCTTTTGCAAGCCAAACAATCAGTGTCAGCACTTTGCTTTGGAAAGTCAGCTGGTGAAAGAGCCCATCAAGGATGGACTCACTCTGATAAACACGCCTCTGAGTGCCAACCAATTAACAAGGGTCTCACTTGAGAGACCACAGTTCTACAGATGATGTTGACCTGTTTATGTCTCTGAAGGCCTGCCAACCCCTGAACTCCAAACCCTGTATAAAATCAGTCCATCCTGCACAGGGAGACTGCCTGACTAGTATA>Minke-whale-ECR4CCATGGCAACTTGCCTCAGCGAACGTGCTTTTGTGAGCCAAACAATACGTGTCACCACTGTGCTCTGGAAAGTCAGCCAGGGATAGAGCCTATCAAGGACGGACTCACTCTGATAAACACGCCTCTGGGTGCCAACCAATCAACATGGGTCTCACTTGAGGGACCACAGTCCTACAGACAACGTGAAGCTGTTTATGCCTCCAAAGGCCCGCCAACCTCTGAACGCCAAACCCTGCATGAAATCAGCCCATTCTGTGCAGAGAGACTG>Panda-ECR4TAAACCCATTCAACCGATCAATGGCAACTCGCCTCAGAGAATGCACTTTTTGCAAGCCAAACAATCAGTGTCAGCACTTTGCTTTGGAAAGTCAGCCACTGAAAGAGCCCATCAAGGATGGACTCACTCCGATAAACACGCCTCTAAGTGCCAACCAATCAACAGGGGTCTCACTTGAGAGGCCACAGTTCTACCGATGATGTCAACCTATTTGTGTCCCGGAAGGCCTGCCAACCCCTGAACTCCAGATTCTGTATAAAATCAGTCCATCCTGC>Rat-ECR4TCGCCTCAGTGGACACGCTTCTGCAAGCCAAACAATTGCTTTGCTTTGGAGAGCCGGGCCCATCAAAGATGGACTCAGTCTGATAAACACGCCTCTGCATGCCAACCAATCCAGCGGGGTCTCACTCCAGAGACCACACTTCTGCAGATGACGTTAATGTCAATCCTGTTCTGTCTTTGAAAGCTGGACAGCACCTGTCCTCTGCTGCCCCCAGACCCTCCACAGAACAGTACCACGCTTCAGAGAGACTCCTGATTAGCCT>White-rhinoceros-ECR4CGCTTTTGCAAGCCAAACAATCAGTGTCAGCACTTTGCTTTGGAAACTCAGCTGGTGAAAGAGCCCATCAGGGATGGACTCACTCTGATAAACACGCCTCTGAGTGCCAGCCAATCCACAAAGGCCTCACTTGAGAGAACACAGTTCTACAGATGATGTCGACCTGTTTATGTCTCTGAAGGCCTGCCAACCCCCGAACTACAAACCCTGTATAAAATCAATCCATTCTGCACAGAGAGACTCCCTGACT>Mouse-ECR5GGTTCTTGGGTGGCAGGCCCAAGGATTCAGCTCCCCCTCCCCTTCTGCTGGGGTCAGGGAAATGAGCCTTCTTCCTGGCCCCTGCGGGGCCTCCAGCCGGCAGCTGGAGGGATTAAGGAAAAGAGGTTCCTGGCGTCCCTGAGTCTGGAGTGGCTGCCCAGGATGTCCCAGGAGAGGCGAGTGGGCCAGAGGGCTGGAGGCTCCCAAATCAATCAGAGGTCTGAAGGTCACTGAATGGCGACCTTTGAATCAGGATCCCGGTAACCCTGAGGCTTTCGCACACTCCGAGGGGGTGGAGCAGA>Human-ECR5CCGGCTACCCCTCCTCTGCTGGGGTGGAAGATGGAGAGGTCAAAGGAGCAAGCCTCCTTCCTGGCCCTTGCAAGGGCCTCCAGCCTGGCAGCCGGAGGGATTAAGGACAAGAGGCCCCTGGCGTCCGTGAGTCTGGCGTGGCTGCCCAGGAGCCTCCCAGGGGAGTCAGCGTGGGCCAGAGGGCTGGAGGCTCCCAGATCAATCAGAGGTCTGAAGGTCACTGGAGCTGTGACCTTCAAATTAGGATACTTGTAACCCAGGAGGCTGGCACACCCCCACGGGGGATGGGGCGCATGGTTG>Alpaca-ECR5CCTCAGACCTCACGGGGGTAGAAACCCTGTCCCTGGCTGTCAGGCAGGGGGCTCAGGCAACGCCCCTCGGTGGAGGTGGAAGATGAGGGGAGGTAACAGGCCTTCTTCCTGGCCCCTCCAAGAGCCTCCAGCCCCAGCCGGAGGGATTAAGGACAGGAGGCGCCTGGCGTCCCTGAGTCTGGTGCAGCTGCCTGGGAGTGTCCCGGGAGAGACGGAGGGGCCAGAGGGCTGGAGGCTCCCAGAGGAATCGGGGACCTGAAGGTCGTTAGAGCCGTGACCTTTAAATTAGGTCACCCGTAACCCCGCAGGCAGGCCCGCCCTCCAAGGCTGGGAAGAAGGGC>Baboon-ECR5CCGTCTCTTGGTGTCAGGCAGGGGTCTCAGGCTACCCCTCCTCTGCTGGGGTGGAAGATGGAGAGGTCAAAGGAGCAAGCCTCCTTCCTGAGCCTTGCAAGGGCCTCCAGCCCGGCAGCCAGAGAGATTAAGGACAAGAGGCTCCTGGCGTCCCTGAGTCTGGTGTGGCAGCCCAGGAGCCTCCCAGGAGAGCTGGAGTAGGCCAGAGGGCTGGAGGCTCCCAAATCAATCAGAGGTCTGAAGGTCACTGGAGCTGTGACCTTCAAATTAAGATACTTGTAACCCAGGAGGCTGGCACACCCCCACGGGGGATGGAGCACACAGTTTAAGAGAATCCATCTCCCAGAGAG>Bonobo-ECR5TATACCCCACTGGGATAGAAATCCTGTCTCTTGGTGTCAGGCAGGGGTCTCCGGCTACCCCTCCTCTGCTGGGGTGGAAGATGGAGAGGTCAAAGGAGCAAGCCTCCTTCCTGGCCCTTGCAAGGGCCTGCAGCCTGGCAGCCAGAGGGATTAAGGACAAGAGGCCCCTGGCGTCCCTGAGTCTGGCGTGGCTGCCCAGGAGCCTCCCAGGGGAGCCAGAGTGGGCCAGAGGGCTGGAGGCTCCCAGATCGATCAGAGGTCTGAAGGTCACTGGAGCTGTGACCTTCAAATTAGGATACTTGTAACCCAGGAGGCTGGCACACCCCCACGGGGGATGGGGCGCATAGTTGAAGAGAATCCATCTCCCGGAGAG>Bushbaby-ECR5AAACAGTAATACCTTCTCCCCTCCCTAGCTCCATTGTGTTCCCTGGACCCTGCTGGAACAGAAATCCTGTTTTCTGTGTGTCAAGCTGGACTCAAGCAGCGCTCCCCCTCCCCTGCAGGGCTGGAACATGGAGGGTAACAGGCTTTCTTGCCAGCCCTTCTAAGGGCCTCCAGCTGCAGCAAGAGGGATTAAGGAGAAGAGGTTCCTGGCGTCCCTGTGTCTGGCTGGCAGGGATGTTCAGGAGGGTCTCAGGGGAATTAGAGTGGACTGGAGACTCCCAGATCAATCAGAGGTGTGAAGGTCACAGGAGCTGACCTTCAAATT>Cat-ECR5CCTGTACAGGATCAGGGACAGTGTACAGGACAGGGGGCTTGCCTGAGGTTGCAAGACCAATGGATTGAGGGAACAAGCTTTCTTCCGGCCACCGTAGCAAGGGCCTCCAGCCCCAGCTGGGGGGATTAAGGAAAGGAGGCTCCTGGCGTCCCTCAGTCTGGTGTGGCAGCCTGGGAGTGTCCCAGGAGAGAATGAGTGGGCCAGAGGGCTGGAGGCTCCAGATCAATCCAAGACCTGAAGGTCATTAGAGCTGTGGCCTTCAAATGAAGTTACTTGTAACCCTGGAGGCACGCACGCCCCCCAAGGCAGGGAAGAAAGACAGAAGAAAGGCCGATTGTTTAAGA>Chimp-ECR5CGGCTACCCCTCCTCTGCTGGGGTGGAAGATGGAGAGGTCAAAGGAGCAAGCCTCCTTCCTGGCCCTTGCAAGGGCCTGCAGCCTGGCAGCCAGAGGGATTAAGGACAAGAGGCCCCTGGCGTCCCTGAGTCTGGCGTGGCTGCCCAGGAGCCTCCCAGGGGAGCCAGAGTGGGCCAGAGGGCTGGAGGCTCCCAGATCGATCAGAGGTCTGAAGGTCACTGGAGCTGTGACCTTCAAATTAGGATACTTGTAACCCAGGAGGCTGGCACACCCCCACGGGGGATGGGGCGCATAGTTGAAGAGAATCCATCTCCCGGAGAG>Chinese-hamster-ECR5GGTTCTTGGGTGGCAGGCGGAAGGACTCAGCTCCCCCTTGCCTTCTGCTGGGTTCAGGGAAATGAGCTGTCTTCCTGGCCCTTATTGGGGCCTCCAGCCTGCAGCTGGAGGGATTAAGGAAAAGCTCCTGGCGTCCCTGAGTCTGGAGTGGCTGCCCGGGATATCCCAGGAGAGGCCACTGGGCCAGAGGGCTGGAGGCTCCCAAATCAATCAGAGGTCTGAATGGTGACCTTTGAATCAGGATCTTGGTAACTCTGGAGACTTGTACACCCTCCAAGGGGGTGGGGGAGAAAGGCAGAT>Ferret-ECR5TCTCACTGGTATATAAATCCTGTCGCATGGGGGTCAGGCAGGGGCTCTGGGAGGCCCTCTCTGCTGTGGTACAAGCCTTCCGCCAACCATGGTGGCCAGAGCCTCCAGCCCCAGCCGGAGGGATTAAGGAAAGAAGGCTCCTGGCGTCCCTCAGTCTGGTGTGGCTGCCTGGGAGCGTCCCAGGAGACACTGAGCAGCCCAGAGGGCTTGAGGCTCCAAATCAATCAGCGACCTGAAGGTCATTAGAGCTCTGGCCTTCAAATTAGGTTACTTGTAACCCTGGAGGCCTGTACACCCTGCAGGGCAGGGAAGAAAGGCGGAAGAAAGGCTGATTGTTTAAGG>Gibbon-ECR5CAGGCTACCCCTCCTCTGCTGGGGTGGAAGATGGAGAGGTCAAAGGAGCCAGCCTCCTTCCTGGCCCTTGCAAGGGCCTCCAGCCCAGCAGCCAGAGGGATTATGGACAAGAGGCCCCTGGCGTCCCTCAGTCTGGCATGGCTGCCTGGGAGCCTCCCAGGGGAGCCAGAGTGGGCCAGAGGGCTGGAGGCTCCCAGATCAATCAGAGGTCTGAAGGTCACTGGAGCTGTGACCTTCAAATTAGGATATTTGTAACCCAGGAGGCTGGCACACCCTCATGGGGAATGGGGCGCATAGTTG>Gorilla-ECR5CGGCTACCCCTCGTCTGCTGGGGTGGAAGATGGAGAGGTCAAAGGAGCAAGCCTCCTTCCTGGCCCTTGCAAGGGCCTCCAGCCTGGCAGCCAGAGGGATTAAGGACAAGAGGCTCCTGGCGTCCCTGAGTCTGGCGTGGCTGCCCAGGAGCCTCCCAGGGGAGCCAGAGTGGGCCAGAGGGCTGGAGGCTCCCAGATCAATCAGAGGTCTGAAGGTCACTGGAGCTGTGACCTTCAAATTAGGATACTTGTAACCCAGGAGGCTGGCACACCCCCACGGGGGATGGGGCGCATAGTTG>Horse-ECR5CCTGTCACTTGGGTGTCGGGCAGGGGGCTCAGGCAACCGCCCTCTGCTGAGGCGGAAGACCAGGGGTCAGGGTAACAAGCTTTCTTCCCGGCTTTTGCAAGGGCCTCCAGCCCCAGCTGGAGGGATTAAGGAGAGGAGGCTCCTGGCGTCCCTCAGTCTGGAGTGGCTGCCTGGGAGTGTCCCAGGAGAGACTGAGCGGGCCAGAGGGCTGGAGGCTCCCAAATCCGTCGGATGCCTGAAGGTTATTAGAGCTGTGGCCTTCAAATGAGGTTGCTGGAAGCTTGGAGGCACACAGGCCCCTCCACGGGGAAGAAAGGCAGATTGTTTAAGAGAAGCAATTTCC>Kangaroo-rat-ECR5CGGGGCCTCCTGCCGGCAGCTGGAAGGATTAAGGCAAAGAGGCTCCTGGAGTCCCCAAGTCTGGCTCTGCTGCCAGCGACCTCCCAGGGGAGTTGGTGCTGGCCAGAGGCCCCGAGGCTCCCAAATCAATCAGGGGGTCTGAAGGGCGTGGGGCTGTGACCTTTGAACAGGGATCCCTGGGACCCCTGCAGGCCGGCAGGCAGGCCCCAGCCCAGGGGGAGAGTGCAGGTTGCAGGATCCATTCCTTGCCTTGGAAAAGGGATCCACCCGCTCCTCT>Marmoset-ECR5CTACCGGGAGAGAAATCCTGTGTCTTGGTGTCAGGCAGGGGTCTCAGGCTACCCCTCCTCTGCTGGGGTGGAAGATGGAGAGGTTAAAGGAACAAACCTCCTTCCTGGCCCCACAGCCAGAGGGATTAAGGACAAGACGCTCCTGGCGTCCCTGAGTCTGGCCTGGCTGCCCAGGAGCCTCCCAGGGGAGCCAGAGTGGGCCAGAGGGCTGGAGGCTCCCAAATCAATCAGGGGTCTGAAGGTCACTGGAGCTGTGACCTTGCACACCCTCACGGGGGGATGGGGCGCATTGTTCAACAGAAGCAGCTTCCCAGAGAGGTGCCCTAGGGCCCCCCCTTCTCCATGACC>Megabat-ECR5ATCTTGTGGCTTAAGCGTCAGTGCAGTCGGCTCAGGCAATCCCCCCTCTGCTGAGGGGTGGAGGAGTTGGGGGAAAAGGCCTCCTTCCTTGCCCTTGAGAGGGCCTCCAGCCCCAGCTGGAGGGATTAAGGGAAGGAGGCTCCTGGCGTCCCTCAGTCTGGGTGGCTGCCTGGGAGCATCCCAGGAGAGACTGAGTGGGCCGCAGGGCTGGAGGCTCCCAAATCCATCGGAGACCTGAAGGTCATTAGAGCTGTGGCCTTCAAATTAGGTTACTTGTAACCCTGGAGGCATGCACACCCTGCAAGGGGTGGAAGAAAGGAGGATTGTTTACCAGAAGCAATTTC>Microbat-ECR5GAGCTCAGGCACCCGCCCCCACTCCGCCGAGGTGGAAGACCGAGGGTGGAGGGGTCAGGGGAACAGGCCGGCTTCCTTCCAAGCCCTTGCGAGGGCCTCTCGCCCCAGCTGGAGGGATTAAGGCCAGGAGGCTCCTGGCGTCCGTCAGTCTGGGGTGGCTGCCTGGGAGCGTCCCGGGAGAGACGGCGCGGGCCAGAGGGCTGGAGGCTCCCAAATCATTCTGAGACCCGAAGGTCATTAGAGCTGTGGCCTCCAAATTAGGTTGCCTGTCACCCCGGGGGCAGGCCTGCGTGCCCTCCGAGAGGGCGGAAGAAAGGGGGCTTGTTTATGGGAAGCA>Mouse-lemur-ECR5GTGAGGCAGGGGGCTCAAGCAACCCCCCACTTCCCTGACCTCCTGCTGGGATAGAAGATGGAGAGGTCAGGGTAACAATCCTTCTTTCAGGCTCTTGCAAGGGCCTCCAGCCCGACAGCCAGAGGGATTAAGGATAAGAGGCTCCTGGCGTCCCTGAGTCTGGTGGGGCTGCCTGGGAGTGTCCCAGGGGAGCTGGCATGGGCTGGAGTGCTGGAGGCTCCCAAATCAATCAGAGGTCTGAAGGTCACAGGAGCTCCAGATTAGGGTACTTACAACCCTGGAGGCTTGCACAGCCTCCTCTGGGAGTGGGGGAGAAAGGCAGATTGTTTAGAGAATCAACTTCCCAGA>Orangutan-ECR5TGGGATAGAAATCCTGTCTCTTGGTGTCAGGCAGGGGTCTCAGGCTACCCCTCCTCTGCTGGGGTGGAAGATGGAGAGGTCAAAGGAGCCAGCCTCCTTTCTGGCCCTTGCAAGGGCCTCCAGCCCGGCAGCCAGAGGGATTAAGGACAAGAGGCCCCTGGCGTCCCTGAGTCTGGCGTGGCTGCCCAGGAGCCTCCCAGGGGAGCCAGAGTGGGCCAGAGGGCTGGAGGCTCCCAGATCAATCAGAGGTCTGAAGGTCACTGGAGCTGTGATCTTCGAATTAGGATACTTGTAACCCAGAGGCTGGCACACCCCCACGGGGGATGGGGCGCATAGTTGAAGAGAATCCATCTCCCAGAGAGG>Pig-ECR5GGCTGTGGAAAGAGTAACTCTGTTCCTGGGATGGCCAGGGGTCTCTGCAGGGTGGAAGACGGGAGGTGAAAGGGACCAAGCCTTCCTCCCCCCCCCAGAAGGGCCTCCAGCCCCAGCTGGAGGGATTAAAGAGAGGCCCCCCCTGGCGTCCCTCAGTCTGGGCCGCTGCCCTGGGAGTGTCCCGGGAGAGACGGGGCCGGAAAGAAGGGCAAAGGGCTGGAGCCCCCCAGGGTCGGGGGACCGGAAGGTCACTGAGCCCGTGACCCTCAAA>Pika-ECR5GGGAACAGTCTTCTCCCCGACCCTTGCAAGGGCCTCCAGCCCGCCAGCCAGAGGATTAAGGGAAAAAGGCTCTTGGCGTCCCTGAGTCTGGTGTGGCTGCCGGGCCCGTCCCAGGGGAGGCCCCCAAATCAATCAGAGGTCTGAGGGTCTTTGTAGCTGTGACCTTCAAATTAGGACCCTTGTGACCCCGGAGGCTTGTGCAGCCTGGAAGGGTGAGAGAGAGGGCGGGTGTTTAAGGCTCTGGCCCCT>Rabbit-ECR5GACCCTAGAGATACAGAAACTCTGACCTTGGATGTCAGGCAGGAGGCTGGGGTGGAGGGGAGGGAGGCCAGGGTGAGGAGTCTTCCTCCTGACCCTTGCAGGGGCCTCCAGCCTGGCCGCCAGAGGGATTAAGGGAAAGAGTCCTGGCGTCCCAGAGTCTGGTGTGGCTGCCGGGAATGTCCCAGGGGAGCTGGAGTGGGCCAGAGGGCTGGAGGCCCCCAAATCAATCAGGGGTCTGAGGATCTTTGTAGCTGTAACCTTCAGATTAGGACACTTGTGACCCCTGAGGCTTGCACAGCCAGTGAGAACAGTGTGGGGGAGAAATGCAGGAGAGAGGAGGGCGTTTCCCC>Rat-ECR5GTTCTTGGGTGGCAGGCCCAAGGACTCCGCTCCCCCTCCCCTTCTGCTGGGGTCAGGGAAATGAGCCTTCTTCCTGGCCCTTGCTGGGGCCTCCCGCTGGCAGCTGGAGGGATTAAGGAAAAGAGGCTCCTGGCGTCCCTGAGTCTGGAGTGGCTGCCCAGGATGTCCCAGGAGAGGCGAGTGGGCCAGAGGGCTGGAGGCTCCCAAATCAATCAGAGGTCTGAAGGTCACTGAATGGCGACCTTTGAATCAGGATCCGGGTAACCCTGAGGCTTGTACGCACTCTGAGGGGGTGGAGCA>Rhesus-ECR5CACTGGGATAGAAATCCCGTCTCTTGGTGTCAGGCAGGGGTCTCAGGCTACCCCTCCTCTGCTGGGGTGGAAGATGGAGAGGTCAAAGGAGCAAACCTCCTTCCTGAGCCTTGCAAGGGCCTCCAGCCCGGCAGCCAGAGGGATTAAGGACAAGAGGCTCCTGGCGTCCCTGAGTCTGGTGTGGCAGCCCAGGAGCCTCCCAGGAGAGCCGGAGTAGGCCAGAGGGCTGGAGGCTCCCAAATCAATCAGAGGTCTGAAGGTCGCTAGAGCTGTGACCTTCAAATTAAGATACTTGTAACCCAGGAGGCTGGCACATCCCCACGGGGGATGGAGCACACAGTTTAAGAGAATCCATCTCCCAG>Squirrel-ECR5AGCCCCCCTGTGCTGGGGTGGGAGAGGAGGGTCAGGGTAACGAGCTTGCCTCCTGGCCCCCACCAGGGCCTCCAGCCGGCAGCTGGATTAAGGCCAAGAGGCTCCTGGCGTCCTTGAGTCTGGAGTGGCTGCCTGGGATGTCCCAGGGGAACTGAGGGGGCCAGAGGGCTGGAGGCTCCCAAATCAATCAGAGGTCTGAAGGTCACTGAGCCGTGACCTTCGAATCAGGATCCTTGTAACCCTGGAGGCACGCACCCTCCAAGGGGTGGGGGCAGATTGCTGAGCGGAGCCCGTTTCCCA>Squirrel-monkey-ECR5GTGTCTTGGTGTCAGGCAGGGGTCTCAGGCTAACCCTCCTCTGCTGGGGTGGAAGATGGAGAGGTCAAAGGAACAAGCCTCCTTCCTGGCCCCGCAGCCAGAGGGATTAAGGACAAGACGCTCCTGGCGTCCCTGAGTCTGGCCTGGCTGCCCAGGAGCCTCCCAGGGGAGCCAGAGTGGGCCAGAGGGCTGGAGGCTCCCAAATCAATCAGAGGTCTGAAGGTCACTGGAGCTGTGACCTTCGAATTAGGATACTTGTAACCCAGGAGGCTTGCACACCCCCATGGGGAATGGGGTGCATTGTTTAAGAGAAGCAGCTTCCCAGAGAG>Tree-shrew-ECR5CTGTGTCTTGGGTGTCAGGCAGGGAGCCCAGGTAACCCCCTCAATCCCGGGGAGAGGATGGAGGGGTCAGGGCAGCAAGCCTTCCTCCCAGCCCTTGCAAGGGCCTCCAGTGGGGGAGGGATTAAGGAAAAGAGACTCCTGGCGTCCCTGAGTCTGCTGTGGCTGCCTGGGAGTGTCCCAGAGGAGCTGGAGAGGGCCAGAGGCCTGGAGGCTCCCAAATCAATCAAAGGTCTGAAGGTCACTGGAGCTGCAACCTTCAAAGGAGGATTGCCATCCTAGAAAGGGGGGTGGGGAGAAAGGCTGTTGTTTATTTAAGAAAATCCATTTCCCAGAGGGCCCTGTGT>White-rhinoceros-ECR5CTGTGTCTTGGGTGTCAGGCAGGGAGCCCAGGTAACCCCCTCAATCCCGGGGAGAGGATGGAGGGGTCAGGGCAGCAAGCCTTCCTCCCAGCCCTTGCAAGGGCCTCCAGTGGGGGAGGGATTAAGGAAAAGAGACTCCTGGCGTCCCTGAGTCTGCTGTGGCTGCCTGGGAGTGTCCCAGAGGAGCTGGAGAGGGCCAGAGGCCTGGAGGCTCCCAAATCAATCAAAGGTCTGAAGGTCACTGGAGCTGCAACCTTCAAAGGAGGATTGCCATCCTAGAAAGGGGGGTGGGGAGAAAGGCTGTTGTTTATTTAAGAAAATCCATTTCCCAGAGGGCCCTGTGT>Mouse-ECR6TATGGTGTGGGATCCATCGTAGCTATTCTAAGGAAGGCCTGCTGGGGTATTCAGATGGTGGGATCCTCCTATTCAGCCCCATCGCTGTGTTTTCCCTCTTCTCTCTGATATTTTTGGTGGCACGGCAAATATTTTCTGCCTGCCTCTTTCTATGAAACACTCAGCAAAAGTGGGAGGTGAGCCACAGCTTGTACTTATTTGGGCTTTCCAAACGCGTGGCACGGTTCTGTGTGCTTTGC>Baboon-ECR6ATGTGTTCTGATAGTGAGATACGCGCATCAGCTGAATTCACATCCACGTTTGTGTTTTCCCTCTTCTCTCCTTCACATCGACGTTTGTGTTTTCCCTCTTCTCTCACTTATGTCTAGCACAGTAGCAAATATTTTTGCCTGCTTCTTTCTATTCAATATGCAGCAAATGAGGCTAATCTTAGCTCACTCTTATTTGGGGC>Cat-ECR6ATGTGTTCTGATAGTGAGATACGCGCATCAGCTGAATTCACATCCACGTTTGTGTTTTCCCTCTTCTCTCCTTCACATCGACGTTTGTGTTTTCCCTCTTCTCTCACTTATGTCTAGCACAGTAGCAAATATTTTTGCCTGCTTCTTTCTATTCAATATGCAGCAAATGAGGCTAATCTTAGCTCACTCTTATTTGGGGC>Chinese-hamster-ECR6CATCGTAGCTATTATAAGGAAGGCCTGCTGGGTATTCAGATGGCGAGCTACTCCTATCGGCTCAATTCAGACCCGTCATCGTGTTTTCCCTCTTCTCTCCGATATTTTTGGTGGCACGGCAAATATTTTCTGCCTGCCTCTTTCTATGAAACACTCAGCGAAAGTGGGAGGCGAGTAACAGCTTGTACTTATTTAGGCTTTCCAGACGCGTGGCACGCTCTTGCGTACTTTTTACATCAG>Rhesus-ECR6ACATGGGGCTGGGAACATGGTAGCTATGGTAAGAAAAGCAAGGGACGCGCATGTGTTCTGATAGTGAGATACGCGCATCAGCTGAATTCACATCCACGTTTGTGTTTTCCCTCTTCTCTCACATATGTCTAGCACAGTAGCAAATATTTTTGCCTGCTTCTTTCTAGTCAATATTCAGCAAATGAGGCTAATCTTAGCTCACTCTTATTTGAGGCTTTCGAAACATGAGACACAATTTTGTGTATTTTGCATAGATTTTCTTACTTTACCCTGACCACAATC>Mouse-ECR7GGCTCCAGGAACTGGCTGTCAGGGAGGAGAGGGTGTAGCTGTCCTGGGGTGATGGAATGTAGCCAGCAGCTGTCTCTTCTCTATTTGAGCCTGTACACTGGCAGGGCACTGTCACGCAAAGCTTCCCCATAAGAGCATGGGGATAGATGATCACTCTCAGGTCATTTATCTGTGTGAGGAAGGCCAGCTTCCTTTGCACCTAGAAGAATAGCCTTAGCTGGCCTGCTGTGG>Chinese-hamster-ECR7GGCTCCAGGAACTAGCTGTCAGGGAGGAGAGGGGCGTAGCTGTCCTGGGGTGATGGAATGCAGCCAGCAGCTGTCTCTCCTCCATTTGAGCCTGTACACTGGCAGGGCACTGTCACGAAAAGCTTCCCCAT>Dolphin-ECR7CACCCTGTGTCCATTGGCTCGATGGGTAAGTAGTTCATCATTTAGGCAAAGGGGGGGCATTTCTTGGCTCCAGTAACCATATCCCGGGGAGGGAAGGTAGGGGTGCAGCTGTCCTCGGGGTGATGGAGAGCAGCCAGCAGCTGTCTCTCTACCACTTGAGTCTGAGGGCTGGCAGCGTGCTGTCAGGTAAGGCCTCCCCATGAGGCCACAACGGTGCTACTCCTTGGTCATCCATCTGCGAGGACCAG>Horse-ECR7CAGCTGGTCTAAAAGTTGCGGATGACAGAAACAACTCAAACTAGTTCAGGCAAGGAGGGGGAATTTCTTGGCTCTAGTAACCACATCCTAGAGAGGCAAGGGGTGTAGCTGTCCCCAGGGTGATGGAATGCAGCCAGCAGCTGTCTCTCTACCACTTGAGTCTGCCTGTTGGCAGCGTGCTGTCACGCAAGGCTTCCCCATGAGGTCAGAACCGTGCTATTCCTAGGCTATCCATCTGTGAGGAGGCG>Minke-whale-ECR7CTGAACCAGCCCCACCAGACCTCTGCCCAACTCAAACTAGTTTAGGCAAAGGGGGGGAATTTCTTGGCTCCAGTAACCATATCCCGGGGAGGGAAGGTAGGGGTGCAGCTGTCCTCGGGGTGATGGAAAGCAGCCAGCAGCTGTCTCTCTACCACTTGAGTCTGCGTGCTGTCATGCAAGCCTTCCCCATGAGGTCACAACGGTGCTACGCCTTGGTCATCCATCTGCGAGGACCAGCCTTGCTTCCC>Pika-ECR7TATCAAGGACACAGCTTAGCTCAGCGTTGAGGATGACAGAAACAACTTAAGCTAGTTTAGACAAAGAGGGAGAATTTCTTGCTTCTGGTGACTGAATCCTAGGGAGGGAAGGAGCGTAGCTGTCCTTAGCATGATGGAATGTAGCTAGCAGCTGTCTCTCTGCCACTCGAGTCTATACTTTGACAGAATGCTGCCACACAAAGCTTTCCCATGAGGTCAGGATCGTGCTGTTCCAGCTTGTCCATCTGTAAAGAGGAGA>Rabbit-ECR7TTTGTTGAATACCAGAAGCACAGCTTGTCTCAAAGGGTTGACAGAAATAACTCAAGCTAGTTTAGGCAGAGGGGAATTTCTTGGCTCTGATAACTAAACTAGGGAGGCAAAGGGTGTAGCTGTCCCCAGGGTGATGGAATACAGCCAGCAGCTGTCTCTCTGCCACTTGAGTCTGCATTGGCAGGATGCTGTCACACACAGCTTTCCCATAAGGTCAAATGGTACTGTTCCCAGGTTATCCATCTGCAAAGAGGAGACT>Rat-ECR7GGCTCCAGGAGCTGGCTGTCAGGGAGGAGAGGGGTGTAGCTGTCCTGGGGTGATGGAATGTAGCCAGCAGCTGTCTCTTCTCCATTTGAGCCTGTACACTGGCAGGGCACCGTCACGCAAAGCTTCCCCAAAAGAGCATAGTGGACCACTCTCAGGTCATCTGTGTGTGTGAGAAAGGCCAACTTCCTTTGCGCCTAGAAGAATACCCTTACCTGGCCTGCCGTGG>Tree-shrew-ECR7TTATCTCAGAGGGCTGAGAGTGACAGAAACAACGCCAACAAGTTTAGGCAAAGGGGGAGTTTCTTGGCTCTGGCAACTAAATCTTAGGATGTAACTGTTTTGGGGTGATGGACTGTCACCAGCAGCTGTCTCTCTGCCACTTGAGTCTGCATGTTGACAGCGAACTGTCACGCAAGGCTTCCCCATGAGGTCAGAATGGTACTATTCCTAGGTTATCCATCACCAAGGAGACTTGATTCCCTTGTCCCTAGAAGAAGACTTAATTGGCCTGGCTTGGTCACTCACT>Mouse-ECR8CCTGGAATCCACAAGAGGAAGGGGTTAAGCCTTCTAGCCCATTTGTCGCTGGAAACTCAGTTTGAATCCCACCCTGGAATTTTCCTCCGAGCTACGAAGCCAGGCGAGCTGCCAAGTCATATATACATTGCCGGGTAGCCAGGCAACTCCGTGCTTTTTCTGGGAAGGAACACAATGTGGTCACTGTTTCTGCATCGCCAGGTTCCAGATGGGAGAAAGACTTTAATCCCCAGCTTCAAGGCTGTTTGGCAAAACTCACTCTGGAGCCCCTGCCTGGGCCGGTGTTCGTTTGCTGTGACCCTCCGGTGCTGCCATTTTGTGACATTCTGTTTCTGGTGTTTAAAATTCAATCCTTGCAAACTAGGGAGGTAGGCTCTGTCAGTG>Human-ECR8TTTGCTCACCTGGCCTGAGTTACCTGGCCGTGGAAGGCCCCTGGGCTCAAGTTCCCTGAGAGGCAGGGGTTAAACCATGTGTCCTAGAACCTCAGCTTGCATCCCACCCTGGAATTTTCCTCTGAGTTGCAAAATCAGGCAAGCTGCCAAGCCATATATACATTGCCGGGTAGCCAGGCAACACCGTGCTTTTTCTGGGAAGGAGCACAATGTGGTCACTGTGCTGCCGACGCCAGGTTCCAGATGGAAGAAAGACTTTAATCCCCAGCTTCACGGCTGTTTGGCACACTTCGCTCCTGAAAGCCTGCTCGGGCCAGTTTTCACTCTGACTCTCCAGTGAGGCCATTTTGTCTCATTCCGTTTCTGATGTTTAAATGTTAATCCTT>Alpaca-ECR8GGACAGAGCGGCCTGGGCCCAGGATGCCCCTCAGCTCAAGCGCCCCAAGAAGAAGGGGTTAAACCTTTCAGCCCATTTGTCCTTGGAACCTCAGCATGCATCCCACCCCTGGAATTTCCCCTCTGAGCTACAAAGCCAGGCCGGCTGCCAAGTCCGATATACATTGCCGGGTAGCCAAGCAACGCGGTGCTCTTTCTGGGAAGGCTCACAATGTGGTCACTGTGCTGTCGACGCCAGGTTCCAGATGGAAGAAAGACTTTAATCCCCAGCTTCACGGCTGTTTGGCACATTTCGCCCCTGAGATCGCACTCTGGCCAGTTCTCACCCCGACTCTCCAGCGCGGCCATTTTGTCACAGCCTGTTTCTGATGTTTAGAGCAGGGTCCTTTGCAGGTTGGTCTGTGTAGATGGGATG>Baboon-ECR8TTTGCTCACCTGGCCTGAGTTACCTGGCCGTGGAAGACCCCTGGGCTCAAGTTCCCCGAGAGGCAGGGGTTAAACCATGTGTCCTAGAACCTCAGCTTGCATCCCACCCTGGAATTTTCCTCTGAGTTGCAAAATCAGGCAAGCTGCCAAGCCATATATACATTGCCGGGTAGCCAGGCAACACTGTGCTTTTTCTGGGAAGGAGCACAATGTGGTCACTGTGCTGCCGACACCAGGTTCCAGATGGAAGAAAGACTTTAATCCCCGGCTTCACGGCTGTTTGGCACATTTCGCTCCTGAAAGCCGGCTTGGGCCAGTTTTCACTCTGACTCTCCAGTGAGGCCATTTTGTCTCATTCCATTTCTGATGTGTAAATGTTAATCCTT>Bonobo-ECR8TTTGCTCACCTGGCCTGAGTTACCTGGCCGTGGAAGGCCCCTGGGCTCAAGTTCCCTGAGAGGCAGGGGTTAAACCATGTGTCCTAGAACCTCAGCTTGCATCCCACCCTGGAATTTTCCTCTGAGTTGCAAAATCAGGCAAGCTGCCAAGCCATATATACATTGCCGGGTAGCCAGGCAACACCGTGCTTTTTCTGGGAAGGAGCACAATGTGGTCACTGTGCTGCCAACGCCAGGTTCCAGATGGAAGAAAGACTTTAATCCCCAGCTTCACGGCTGTTTGGCACACTTCGCTCCTGAAAGCCTGCTCGGGCCAGTTTTCACTCTGACTCTCCAGTGAGGCCATTTTGTCTCATTCCGTTTCTGATGTTTCAATGTTAATCCTT>Bushbaby-ECR8AGGCCTGGGTTACCTGGGCCTTGGAACGCCCCTCAGGCGAATCTTCCCAAGAAGAAGAGGCTAAGCCTTCCAGCCCCTCTGTCCTGGGAACTTCAGCTGCATCCCACCCTGGAATTTTCCTCTGAGCTACAGAGCCAGGCTGGCTGCCAAGTCACATATACATTGCTGTGTAGCCAGGCAACGTGGACTTTTTCTGGGAAGGAGCATAATGTGGTCACTGTTTCCCAGACACCAGGTTCCAGATGGAGGAAAGACTTTAATCCCCAGCCTTGCGGCTGTTGGGCACATTTCACTCCTGAGATCCTGCTCTGGCCACTTCCACTCTAACTCGACAGTGTGGCCATTTTGCCACATTCTGTTTCTGATGTTTAAATTTTAATCCTCTACAAGTTAGCTTTAGTGGAGTAGGACATGGGAGGGGACACTGGAGGTGAATTTAAATGCAGTCAGGACAACTGTACACCAGTCCTTCT>Cat-ECR8AAGAAGGGGTTAAACCTTCCGGCCCATTTGTCCTTGGAACCTCAGCTTGCATCCCACCCTGGAATTTTCCTCTGAGCTACAAAGCCAGGCCAGCTGCCAAGCCATATATACATTGCAGGGTAGCCAGGCAACGCGGCGCTCTTTCTGGGAAGGCTCACAATGTGGTCACTGTTTTGTAGACGCCAGGTTCCAGATGGAAGAAAGACTTTAATCCCCAGCTTCAAGGCTGTTTGGCACACTTCACTCCTGAGATCCCGCGCGGGCCAGTTTTCACTCTGACTCTCCAGCGTGGCCATTTTTGTCACATTCTGTTTCTGATGTTTTAGATTTTAATCCTTTGCAAGTTGGGTTTGCGTAGAGAGGGGAGATGGAGACAAGACCGGAGGGAAGTTTAGACACAGAGAGAGGCGGGACCTCCGTGTCACGGTGAAGGCATGAGAGCG>Chimp-ECR8TTTGCTCACCTGGCCTGAGTTACCTGGCCGTGGAAGGCCCCTGGGCTCAAGTTCCCTGAGAGGCAGGGGTTAAACCATGTGTCCTAGAACCTCAGCTTGCATCCCACCCTGGAATTTTCCTCTGAGTTGCAAAATCAGGCAAGCTGCCAAGCCATATATACATTGCCGGGTAGCCAGGCAACACCGTGCTTTTTCTGGGAAGGAGCACAATGTGGTCACTGTGCTGCCAACGCCAGGTTCCAGATGGAAGAAAGACTTTAATCCCCAGCTTCACGGCTGTTTGGCACACTTCGCTCCTGAAAGCCTGCTCGGGCCAGTTTTCACTCTGACTCTCCAGTGAGGCCATTTTGTCTCATTCCGTTTCTGATGTTTCAATGTTAATCCTT>Chinese-hamster-ECR8CCTAGAATCCACAGGATGAAGGGGTTAAGCCTTCCAGCCCATTTGTCGCTGGAAACTCAGTTTGAATCCCACCCTGGAATTTTCCTCTGAGCTACGAAGCCAGGCGAGCTGCCAAGCCATATATACATTGCCGGGTAGCCAGGCAACTCCGTGCTTTTTCTGGGAAGGAACACAATGTGGTCACTGTTTCTGCAGTCGCCAGGTTCCAGATGGGAGAAAGACTTTAATCCCCAGCTTCAAGGCTGTTTGGCAAAACTCACTCTGAAGCCCCTGCCTGGGCCGGTGTTTGTTTGCTGTGACCCTCGTGGGCTGCCATTTTTGGGATGTGCTGTTTCTGGTGTTTCAGTTTT>Dog-ECR8GAAGGGGTTAAACCTTTAGGCCCATTTGTCCTTGGAACCTTAGCTTGTATCCCACCCTGGAACTTTCCTCTGAGCTACAAAGCCAGGACAGCTGCCAAGTCATATATACATTGCTGGGTAGCCAGGCAACGTGGCGCTTTTTCTGGGAAGGCTCACAATGTGGTCACTGTTTTGTAGAAGCCAGGTTCCAGATGGAAGAAAGACTTTAATCCCCAGCTTCGCGGCTGTTTGGCACATTTCACTCCTGAGATCCTGCTTGGGCCAGTTTTCACTCTGACTCTTCAGTGTGGCCATTTTGTTACATTCTGTTTCTGATGTTTAAATTTTAATCTGTTGCAAGTCGGTTTGTG>Elephant-ECR8CTGACCTGGGTTACTGATCCTGGAATGCCCCTGAGTTCCAGATCTCTGAGAAGAAGGGGTTAAACCATGCAGGCTATTTGTCCTTGAAACCTCTGCTTGCATCCCACCCTGGAATTTTCCTCATACCTACAAAGCCAGGCAGCCTGCCAAGTCATATATACATTGCTGGGTAGCCTGGCAACACTGTGCTCTTTCTGGGAAAGCTCACAATGTGGTCACTGTTTTGCAGACACCAGGTTCCAGATGGAAGAAAGACTTTAATCCCCAACTTTGCGGTTGTTTGGCACATCTCGCTCCCGAGGCCTGCTCTGGCCAGTTTTTGCTCTGATTCTGCAGTGCAGCCATCTTTGTTGCATTTGGTTTCTGATTCTTACATTTTAATCTTT>Ferrert-ECR8GCTAAGGTTATCTGGGCCCAGAAGGCCCCTCAGCTTGAGCTCTCTGAAAAGAAGGGGTTAAACCTTTCGGCCCATTTGTCCTTGGAACCTCAGCTTGCATCCCACCCTGGAACTTTCCTCTGAGCTCCAAAGCCAGGACAGCTGCCAAGTCCTATATACATTGCGGGTAGCCAGGCAACACGGAGCTTTTTCTGGGAAGGCTCACAATGTGGTCACTGTTTTGTAGACGCCAGGTTCCAGATGGAAGAAAGACTTTAATCCCCACTTTCGCGGCTGTTTGGCACATTTCACTTCTGAGATCCTGCTCGGGCCAGTTTTCATTCTGACTCTCCAGCGTGGCCATTTTATCCCATTCTGTTTCTGATGTTTAAAACTTTAATCCTTTGCAAGTTGGTTTGTGTAGAGTGGGGAACTGGAGAGTAGATTGGAGGGAAGTTTACGCACAAAGAG>Gibbon-ECR8GGTTTGCTCACCTGGCCTGAGTTACCTGGCTGTGGAAGGCCCCTGGGCTCAAGTTCCCAGAGAGGCAGGGGTTAAACCACGTCCTAGAACCTCAGCTTGCATCCCACCCTGGAATTTTCCTCTGAGTTGCAAAATCAGGCAAGCTGCCAAGCCATATATACATTGCTGGGTAGCCAGGCAACACCGTGCTTTTTCTGGGAAGGAGCACAATGTGGTCACTGTGCTGCCGACGCCAGGTTCCAGATGGAAGAAAGACTTTAATCCCCAGCTTCACGGCTGTTTGGCACACTTCGCTCCTGAAAGCCTGCTTGGGCCAGTTTTCACTCTGACTCTCCAGTGAGGCCATTTTGTCTCATTCCGTTTCTGATGTTTAAATGTTAATCCTT>Gorilla-ECR8TTTGCTCACCTGGCCTGAGTTACCTGGCCATGGAAGGCCCCTGGGCTCAAGTTCCCTGAGAGGCAGGGGTTAAACCATGTGTCCTAGAACCTCAGCTTGCATCCCACCCTGGAATTTTCCTCTAAGTTGCAAAATCAGGCAAGCTGCCAAGCCATATATACATTGCCGGGTAGCCAGGCAACACCGTGCTTTTTCTGGGAAGGAGCACAATGTGGTCACTGTGCTGCCGACGCCAGGTTCCAGATGGAAGAAAGACTTTAATCCCCAGCTTCACGGCTGTTTGGCACACTTCACTCCTGAAAGCCTGCTCGGGCCAGTTTTCACTCTGACTCTCCAGTGAGGCCATTTTGTCTCATTCCGTTTCCGATGTTTAAATGTTAATCCTT>Hedgehog-ECR8TTGTCCCTGGAACCCCTCTCTCTCTCTCTCTCCTTCTCCCTCTCTCTCTCTCTCTCTCTCTCTCTCTCTCCTTCTCCCTCTCTCTCTCCCTTCCTCCTGCATCCCACCCTGGAATTTTCCTCGGAGCTACAAAACCCGGCCAGCTGCCAAGTCATATATACATTTGCCGGGTCACCAGGCAACACCGAGCTTCTCCTGCTTCTTCTGGGAAGGCTGACAATGCGGTCACTGTGCTGCGGTCGCCAGGTTCCAGATGGAGGGGCCCCCCGTCCCAGCCCCGAGGCCACCCGCCCTCTGCCACGTGCGCCCACCCGGCAGCCATGCACCTCGGGCCTGGCCGGGTGTGAGGAGTGGGGG>Horse-ECR8CCTGGCCGGGATTACCTGGGAGGGGAATACCCCTCAGCTGAAGCTCCCTGAGAGGAAAGGGTTAAAACTTTCAGCCCATTTGTCTTGGGACCTCAGCTTGTATCCCACCCTGGAATTTTCCTCTGAGCTACAGGCCAGCTGCCAAGTCATATATACATTGCTGGGTAGCCAAGCAACCCAGTGCTTTTTCTGGGAAGGCTCACAATGCGGTCACTGTTTTGTGGACGCCAGGTTCCAGATGGAAGAAAGACTTTAATCCCCAGCTTCGCGGCTGTTTGGCACATTTCACTCCTGAGATCCAGCTTTGGCCAGTTCTCACCCTGATTCTCCAGTACGGCCATTTTGTCACTTTCTGTTTCTGGATGTTTCTGATGTAAGCTTTAATCCTGTATGGGTTGGTCTGTGTGGAGTGGGAAATTGGAGAGAAGACTGGAGGGAAATTGAGACACAGTAGGGACAGCACCTACG>Manatee-ECR8CACCTGGCCTGGGTTACCAAGCCCAGAATGCCCCTGAGCTCCAGCTCTCTGAGAAGGGGTTAAACTTTGCAGTCTATTCGTCCTTGAAACCTCTGCTTTCATCCCACCCTGGAATTTTCCTCGTAGCTACAAAGCCAGGCGGGCTGCCAAGTCATATATACATTGCTGGGTAGCCAGGCAACGTTGTGCTCTTTCTGGGAAGGCTCACAATGTGGTCACTGTTTTGCAGACACCAGGTTCCAGATGGAAGAAAGACTTTAATCCCCAGCACTGCTGTTTGGCACATCTCGCTCCTGAGGCCTGTTCTGGCCAGTTTTCACTCTGATTCTACAGCGCAGCCATTTTGTTGCATTTGGTTTCTGATTCTTACATTTTAATCTTTT>Marmoset-ECR8GTTTGCCCACCTGGCCTGGGTTACCTGGCCGTGGAATGCCCCTCAGCTAAGTTCCCCGAGAGGCAGGGGTTAAACCGTGAGTCCTAGAACCTCAGCTTGCATCCCACCCTGGAATTTTCCTCCAAGTTGCAAAATCAGGCAAGCTGCCAAGCCATATATACATTGCCGGGTTGCCAGGCAACGCCGTGCTTTTTCTGGGAAGGAGCACAATGTGGTCACTGTGCTGCAGACGCCAGGTTCCAGATGGAAGAGAGACTCTAATCCCCGGTTTCACGGCTGTTTGGCACGTTTCGCTCCTGAAATCTTGCTGAGGCCAGTTTTCACTCTGACTCTCTAGTGAGGCCATTTTGTCTCATTCCGTTTCTGATGTTTAAACTTTAATCGTT>Megabat-ECR8GAAGGGGTTAAACTGTTTAGCCCATTTGTCCTTGGAACATCAGCTTGCATCCCACCCTGGAATTTTTCTCTGAGCTGCAAAGCCAGGCCAGCTGCCAAGTCATATATACATTACTGGGTAGCCAGGCAACGCAGTACTTTTTCTGGGAAGGCTCACAGTGTGGTGACTGTTTTGTAGACGCCAGATTCCAGATGGAAGAAAGACTTTAATCCCCAGCTTGGAGGCTGTTTGGCACATTTCATTCCTGAGATCCTGCGGGGGCCAATTTTTACTCTGACTCTCCAGTGCAGCCATTTTGTCACATTCTGTTTCTGGTGTTTAAATTGTAATCCTCTACAAGTCGGTTTCTGTAGAGTGGGAAATTGGAGAGAAGTCTGGAGGGAAATTGGAAAAAGACATGGGAGCAAAACGATAAATTAGGTG>Microbat-ECR8CTTGAGGAGGAGAAGGGGTTAAACCTTCCATCCCGTTTGTCCCGGGGACCTCCGCTCGCATCCCACCGTGGAATGTTCCTCGGAGCTACAAAGCCCGGCCAGCTGCCAAGTCGTGTATATATACATACGTTGCCTCGTCGCCAGGCAACGCGGCGCTCTTTCTGGGAAGACGCACAATGTGGTCACTGTTCCGGAGACTCCAGGTTCCAGATGGAAAGAGAGACTTTAATCCCCGGCTTCGCGGCTGTTTGGCACGTTTCTCTCCGGAGATGCTGCCGGGGCCCGATTTCCACTCTGACACCCGCGACGCGGCCCTTTCTCCGTCTTGATGTTTAAAAACTGTAATTCGTGGCA>Mouse-lemur-ECR8GGCCTGGGTTACCTGGGCCCCGGAACGCCCCTCCACACAACCTCCCTGAGAAGAGGGGGTTAAACCTTCCAGCCCCTCTGTCCTTGGAACTTCAGCTTGCATCCCACCCTGGAATTTTCCTCTGCGCTCGAAAGCCAGGCCAGCTGCCAAGTCATATACATTGCTGGGTAGCCAGGCAACGCCGTGCTTTTTCTGGGAAGGAGCACAATGTGGTCACTGTTTTGCAGACGCCAGGTTCCAGATGGAAGAAAGACTTTAATCCCCAGCTCCGCGGCTGTTTGGCACATTCCACTCCTGAAATCTGTGCCGGCCTACTCTCACGCTGGCTCTCGTGTGGCCATCTCATCACATTCTGTTTCTGATGTTTAACTTTTCACCCTTTATGAGTTGTCTTTAGTGGAGGAGGAGATGGGAGAAGATGCTGGCAGTGAATTCAGATACAGTAGGGATGCCACCTACATCTTACCTT>Orangutan-ECR8TTTGCTCGCCTGGCCTGAGTTACCTGGCTGTGGAAGGCCCCTGGGCTCAAGTTCCCTGAGAGGCAGGGGTTAAACCATGTGTCCTAGAACCTCAGCTTGCATCCCACCCTGGAATTTTCCTCTGAGTTGCAAAATCAGGCAAGCTGCCAAGCCATATATACATTGCCGGGTAGCCAGGCAACACTGTGCTTTTTCTGGGAAGAAGCACAATGTGGTCACTGTGCTGCCGACGCCAGGTTCCAGATGGAAGAAAGACTTTAATCCCCAGCTTCACGGCTGTTTGGCACACTTCGCTCCTGAAAGCCTGCTTGGGCCAGTTTTCACTCTGACTCTCCAGTGAGGCCATTTTGTCTCATTCCATTTCTGATGTTTAAATGTTAATCCTT>Panda-ECR8GCTGAGGTTACCTGGGCCCAGAAGGCCCCTCAGCTTGAGCTCTCTGAAAAGAAGGGGTTAATCCTTTCGGCCCATTTGTCCTTGGAACCTCAGCTTGCATCCCACCCTGGAACTTTCCTCTGAGCTACAAAGCCAGGACAGCTGCCAAGTCATATATACATTGCCGGGTAACCAGGCAACGCAGCGCTTTTTCTGGGAAGGCTCACAATGTGGTCACTGTTTTGTAGACGCCAGGTTCCAGATGGAAGAAAGACTTTAATCCCCAGCTTCGCGGCTGTTTGGCACATTTCACTCCCGAGATCCTGCTCGGGCCAGTTTTCGCTCTGACTCTCGAGTGTGGCCATTTTGTCCCATTCTGTTTCTGATGTTTAAATTTTAATCCTTTGCAAGTTGGTTTGTGTAGAGTGGGGAATTGGAGAAGAGATTGGAGGGAAGTTTACATACCAAGAC>Pika-ECR8TGCTCAAGTTTGGTTTCTGGCCTGCATTACTTGGGTCTGAAATGCGCCAGGAGTTGGGGTTAAACCTTTTGGGCCATTTGTCACTGGAACTTCAGCCTGTATCCCACCCTGGAATTTCCCTCGGAGCTACAAAGCTGGGCCAGCTGCCAAGTCATATATACATTGCTGAGTAGCCAGGCAACGCTGTGCTTTTTCTGGGAAGGAGCACAATGTGGTCACTGTTTTGCAGACACCAGGTTCCAGATGCAAGCAAGACGTTAATCCCTAGCTCCACGGCCCACTGGCACATCTCACTCCTGAAATCCTGCCGGGGCCAGTTTTCACTCCCACCCTCCAGCGCGGCCATTTTGTCAGATTCTGTTCCTGATGC>Rabbit-ECR8GCCTATTTTACAATGAAGGAAACCAAAGCCCGTTGAAGTTTGATCTCCTGACCTTAGGGGTTAAACCTGTGGACCATTTGTCACTGGAACTTCAGCTTGCATCCCACCCTGGAATTTTCCTCTGAGCTACAAAGCTGGGCCAACTGCCAAATCATATATACATTGCTGGGTAGCCAGGCAACGCTGTGCTTTTTCTGGGAAGGAGCACAATGTGGTCACTGTTTTGCAGACGCCAGGTCCCAGATGGAAGAAAGACTTTAATCCCTAGCTCGGCGGCTGTTTGGCACATTTCACTCCTGAAATCCTGCTGTGGCCAGTTTTCACTCCCACTCTCCAGTGCGGCCATTTTGTCAGATTTTGTTCCTGATGTTTAAAGTTTAATCCTT>Rat-ECR8CCTGGAATCCACAAGAGGAAGGGGTTAAGCCTTCTAGCCCATTTGTCCCTGGAAACTCAGTTTGAATCCCACCCTGGAATTTTCCTCCGAGCTACGAAGCCAGGCGAGCTGCCAAGTCATATATACATTGCCGGGTAGCCAGGCAACTTCGTGCTTTTTCTGGGAAGGAACACAATGTGGTCACTGTTTTTGCAGTCACCAGGTTCCAGATGGGAGAAAGACTTTAATCCCCAGCTTCAAGGCTGTTTGGCAAAACTCACTTTGGAGCCCCTGCCTGGGCCGGTGTTCATTTGCTGTGACCCTCCGGTGCTGCCATTTTGTGACATTCTGTTTCTGGTGTTTTAAACTCAATCCTTGCAAACCAGGGAGGTGGCTCTGACAATGAAGCTTGCTACACAAC>Rhesus-ECR8TTTGCTCACCTGGCCTGAGTTACCTGGCCGTGGAAGACCCCTGGGCTCAAGTTCCCCGAGAGGCAGGGGTTAAACCATGTGTCCTAGAACCTCAGCTTGCATCCCACCCTGGAATTTTCCTCTGAGTTGCAAAATCAGGCAAGCTGCCAAGCCATATATACATTGCCGGGTAGCCAGGCAACACTGTGCTTTTTCTGGGAAGGAGCACAATGTGGTCACTGTGCTGCCGACACCAGGTTCCAGATGGAAGAAAGACTTTAATCCCCGGCTTCACGGCTGTTTGGCACATTTCGCTCCTGAAAGCCGGCTTGGGCCAGTTTTCACTCTGACTCTCCAGTGAGGCCATTTTGTCTCATTCCATTTCTGATGTGTAAATGTTAATCCTT>Rock-hyrax-ECR8CTGGCCTGAGTTACTGAGCCCAGAATGCCCCTGAGCTCCAGCTCTCTGAGAAGAAGAGGTTAAAGCTTGCAGGTTATTTGTCCTTGAAACCTCTGCTTGCATCCCACCCTGGAATTTTCCTCCTACCTACAAAGCCAGGCTGATTGCCAAATCCTATATACATTGCTGGGTAGCCAGGCAACGATGTGCTCTTTCTGGGAAGGCTCACAATGTGGTCACTGTGTTGCAGACACCGGGTTCCAGATGGAAGAAAGACTTTAATCCCTAGCTTTGTGGCTGTTTGGCACATCACACTTGTGAGGCCCACTCTGGCCAGTTTTCACTCTGATTTTGCAGTGCAGTCATCTTGCTGTGTTTGGTTTCTGAGTCTTACATTTTAATCTTTTCATGTCCGATCGAAATAGATTAAAAA>Sloth-ECR8TTTGGTCACCTGGCTGTGCTCACCTGGGCCCAGCACAAGCTCCCTGAGAAGAAGGGGTTAAGCCTTTCAGCCCGTTTGTCCTGGAAACCTCAGCTTGCATCCCACCCTGGAATTTTCCTCTGAGCTACAAAGCCAGGCCAGCTGCCAAGTCACATATACATTGCTGGGTAGCCAGGCAACGCCGTGCTGCTTCTGGGAAGACGCACAATGTGGTCACTGTTCTGCAGACGCCAGGTTCCAGATGCAACAAAGACTTTAAGCCCCGGCTTCGTGGCTGTTTGGCTCATCTCACTCCTGAGAGCCTGCTTCGGCCTCTCTTCTCTCTGATTACCCAGCGTGGCCATTTTGTCACATTTCCTTTCTGATTTTTGAATTTTAGTTCTTTAAAAGTTGGCTTGTGTGTAGTAGAAAATTGGAGAGACGTCTGGGGGAAGTTTAAACACAGCAAGGACAGAACCAGTGTA>Squirrel-ECR8AACCGGCCCACGTTCCCCGGCCCAGCGCCCTCGGCACAGGCCCCCGAGAAGAAGGGGTTAAGCTGCTCAGTCCATCTGTCACTAGAACCTCTGCCTGAATCCCACCCTGGAATTTTCCTCTGAGCGAGGAGCCAGGCCAGCTGCCAAGTCATATATACATTGCCGGGTAGCCAGGCAACGCCGTGCCTGTTCTGGGCAGGAGCACAATGTGGTCACTGTTCTGCAGACGCCAGGTTCCAGATGGAAGAGAGACTTTAATCCCAGCTTCGCGGCTGCTTGGCGCACGTCACTCCCGCAGCCTGCTCCGCCAGGCTCGCCCCACACCAGGCTCCTGCGGCCATTTTCCCCATCCTGCTTCCGATGTTTGA>Squirrel-monkey-ECR8GTTCGCTCACTGGGCCTTGGTTACCTGGCCGTAGAATGCCCCTCAGCTAAGTTCCCTGAGAGGCAGGGGTTAAACCGTGAGTCCTAGAACCTCAGCTTGCATCCCACCCTGGAATTTTCCTCCAAGTTGCAAAATCAGGCAAACTGCCAAGCCATATATACATTGCTGGGTTGCCAGGCAACGCCGTGCTTTTTCTGGGAAGGAGCATAATGTGGTCACTGTGCTGCAGACGCCAGGTTCCAGATGGAAGAGACACTCTAATCCCCGGTTTCACGGCTGTTTGGCACGTTTCGCTCCTGAAATCTTGCTTAGGCCGGTTTTCACTCTGACTCTCTAGTGAGGCCATTTTGTCTCATTCCGTTTCTGATGTTTAAACTTTAATCCTT>Tarsier-ECR8GCCTTGATCACCTGGGCCTGGAAGGCCCCTCAACTCAAGGTCCCCGGGTAGAAGGGGTTAAAGCATTTGTCCCTGGAACCTCAGCTTGCATCCCACCCTGGAATCTTCCTCTGAGCTGCAAAGCCAGGCCAGCTGCCAAGTCATATATACATTGCTGGTAGCCAGGCAACGCCGTGCTTTTTCTGGGAAGGAGCACAATGTGGTCACTGTGCTGCGGACACCAGGTTCCAGATGGAAGAAAGACTTTAATCCCAGCTTCGCGGCTGTTTGGCACATTTCACTCCCTGAAACCCTGCTTGGGCCAGTCTTCGCTGTGACTGTCCAGTGTGGCCATTCTGTCACATACTGTTTCTGATGTTTGAATTTTAGTCCT>Tenrec-ECR8CACTTGGCTCGGGTTCCCCCTCGACCCTCCCGCCCCGCTCTGAGAAGAAGGGGTTACGTCTTGGAGACCATCTGTCAGTGACCCCTCTAGATGCGTTCCTTGGAATTTTCCTCCGAGCCACAGAGCCAGGCGGGTCGCCAAACCATATATACATGGCTGGGTAGCCAGGCAACGCGGTGCTCTTTCTGAGAATGCTCACAATGTGACCACTGTTCCTCAGACCTCGGGTTCCAGATGCGGAGCAAGACTTTAATCCCCAGCCTGGCAGCTGTTTGGCATCTCTCACTCCTGAGACCCGCCTGCTCTGCCCGTCTCCACGCGGATTCCCCAGGGCAGCCCTCTTGTCGCCTTCGGCTTCTGGCTCTTACACGCCACTCTTTTCACATTG>Tree-shrew-ECR8CCTAGCTCGGGTTACCTGGGGCTGGAAGGCCCCTCGGCTCAGTCTCCCATTGAAGAAGGGGTTAAACTTTCAGCCATTTGTCGTTGGAACCTCGGCTTCCATCCCACTCTGGAATTTTCCCTTGAGCTACGAAGCCAGGCCAGCTGCCAAGTCATATATACATTGCTGGGAGCCAGGCGACGCTGCGCTTTTTCCGGGAAGGAGCACAATGTGGTCACTGTTTTGCCGATGCCAGGTTCCAGATGGAAGAAAGACTTTAATCCCCAGTTTCGAGGCTGTTTGGCACGTTTCACTTCTGAGATCCTGCTTTGGCCAGTTTTCACCCTGATTCTCCAGTGTGACCATTTTGTCTCATTCTGTATCGAATGTTTAAGTTTTGATCCTT>Mouse-ECR9CTGGCTACACTGAATCCCACAGCTTTTGCTGATAGTTTTAAACCAAGGACAAACAGCACAACCAAGACAAGATCCTATGTGTGCACGTGTGTGCAGGCAGCTGCATCCAAGGACATGATTAGCCGTTAAGCCCCCTCTGGATCCATGCATCCCTTGTTCAGCACATTCTCTTCCCAGCAAAAAGGCTGGAGCCCCAACGGTGTTGTGTAATTCCCACCACACCCCAAG>Bonobo-ECR9GGCCCACAGCTCCTGCTGGTAGCTTTACACTAAGAACGACTGCGGTCTGCCAAGATCATGTGTGCACGTGCCTCCGGGCAGCTGCATCCAAGGACGTGATTAACTGCTAAGCCCCCCTGGATCTATGCATTCCTTGGTCAGCACATTTTCTTCCCAGTAAAGAGGCTACAGACCCAGCGATGTTATGTAATCCCCACCACACCCCATGTG>Cat-ECR9CCCACAGCCCTGCTGATAGTTTTAAACTAAGAACAAGTCAAGACTGCCAAGATCATGCGCACACACGTGTACCCAGGCAGCTGCGTCCAAGGACATGATTAACTGCTAAGCCCCTCTGGATCGAGGCATCGCCTGGTCAGCACATTCTCTTCCCGGTAAAGCGGCTGGAAGCCCGATGATGTTGAAGAATCCTCACCACA>Chimp-ECR9GGCCCACAGCTCCTGCTGGTAGCTTTACACTAAGAACGACTGCGGTCTGCCAAGATCATGTGTGCACGTGCCTCCGGGCAGCTGCATCCAAGGACGTGATTAACTGCTAAGCCCCCCTGGATCTATGCATTCCTTGGTCAGCACATTTTCTTCCCAGTAAAGAGGCTACAGACCCAGCGATGTTATGTAATCCCCACCACACCCCATGAG>Chinese-hamster-ECR9CCGGCTACACAGAATCCCACAGCTTTTGCTGATAGTTTTAAACCAAGGACAAACAGCACAGCCAAGACCGAGAATCCTACGTGTGCACGTGTATCCAGGCAGCTGCATCCAAGGACATGATTAGCTGTTAAGCCCCCTCTGGATCCATGCAGCCCTTGTTCAGCACATTCCCTTCCCAGCAAAGAGGCTGGAGGCCCAACGGTGTTGTGTAATTCCCACCACACCCCAAG>Cow-ECR9AAATCTGCCAAAAGCCATGTGCACACACGTGTAATCCAGGCAGCTGTGTCCAAGGTCATGATTAACTGCTAAGCCCCCCTGGATCTGTGCGTTCACTCGGTCAGCACATTCTCTTCCCGGGAAAGCGGCTGGAGGCCCAGTGGTGTGATGTAACTCTCACCACACCCCATGAGAAACATG>Dog-ECR9CCCACAGCCCTGCTGATAGTTTTAAACTAAGAACAACCCAAGTCTGCCAAGATCATATGCACACATGTGTACCCGGGCGGCTGAATCCAAGGACATGATTAACTGCTAAGCCACTCTGGATCAATGCATCGCTTGGTCAGCACATTCTCTTCCCAGTAAAGCGGCTGGAAGCCCAGTGATGTTATATAATTCTCACCACA>Elephant-ECR9TGTGTTCTAGATTCATGCATGTCGTGGCTTTGCTGGTAGTTTTAAACTAAGAACAATGTACGTTGGCCAAGATCATGTGTGCTCCCATGCATCCAAGCAACTGCCTCCAAGGACATGATTAACTGCTAAGCCCCCTGGATCTATGCATCACTAGGCCAGCACATTCTCTTCCCAGAGAAGAGGCAAGAGGCCCAATGACACTATTTAATCCCATGACCGACACCCCAGTTAGGCATGCCTTCTAATCATT>Ferret-ECR9TAAAACTAAACCCCAGCTTCCTCTTGCCACATCAAAGCCCACAGCCCTGCTGATAGTTTAAAACTAAGAGGGACGTGAGCCTGCCAAGATCATGTGCACACACGTGTATCCAGGCAGCTGCGTCCAAGGACATGATTAACTGTTAAGCCCCTCTGGATCAATGCATCGCTCAGTCAGCACATTCTCTTCCCAGTAAAGCAGCTGGAAGCCCAATGATATTATATAATTCTCACCACACCCCGTGTGAAGC>Gorilla-ECR9GGCCCACAGCTCCTGCTGGTAGCTTTACACTAAGAACGACTGCGGTCTGCCAAGATCATGTGTGCACGTGCCTCCGGGCAGCTGCATCCAAGGACGTGATTAACTGCTAAGCCCCCCTGGATCTATGCATTCCTTGGTCAGCACATTTTCTTCCCAGTAAAGAGGCTACAGACCCAGCGATGTTATGTAATCCCCACCACACCCCATGTG>Horse-ECR9CCCAGCTTCCTCTTGCTACACCAAAGTCCACAGCCCCCGCTGGCAGTTTTAAACTAAGAACAACCGAAGGCTGCCAAGATCATGTGCACACATGTGTATCCAGGCAGCTGCGTCCAAGGACGTGATTAACTGCTAAGCCCCCCAGGATCTATGCATTACTTGGTCAGCACATTCTCTTCCCAGGAAAGCGGCTAGAAGCCTAATGATGTTATGTAATCCTCACCACACCCCATGTGAAAC>Megabat-ECR9TCTAAACTCCAGCTTCCTCGTGTTACATCAAAGCCCACAGCCCCTGTTGGTGGTTTGAAACTAAAAACAACTGAAGGCTTTCCAGATCATGTGTACGCATGTGTGTCCAGGCAGCTGCATCCAAGGACATAACTAACAGCTAAGCCCCCCTGGATCTATGCATTACTTGGTCAGCACATTCTCTTCCCAATAAAGTGGCTCAGAGCCCAGAGAGGTTTTGCAATCCTCACCACACCCCAT>Mouse-lemur-ECR9AGCACACAGCTCCTGCTGGCAGTTTTAAACTAAGAACTCCTGAAGTCTGCCAAGATCCTGTGCATGCATGTGTGTCCAGGCAGCTGCATCCAAGGACATGATTAACTGCTAAGCCCCCTGGATCTATGCATTCCTTGGTCAGCACATTCTCTTCCCAGCAAAGAGGCTACAGGCCCAATGATGTTATGCAATCGCCACCACACCCCATGTGAGACAAATC>Panda-ECR9CTACATCAAATCCATAGCCCTGCTGATAGTTTTAAACTAAGAACGACTCCAGTCTGCCAAGATCATGTGCACACGCGTGTATAGAGGCAGCTGCGTCCAAGGACATGATTAGCTGCTAAGCCCCTCTGGATCGATGCACTGCTTTGTCAGCACATTCTCTTCCCAGTAAAGCGGCTGGAAGCCCAATGATGTTATATAATTCTCACCACGCCCCATGTGA>Pig-ECR9GCCTCCTCTCGCTGCCCCCAAAGCCCACAGAACCCACCAGCCGTAGTTTTCAACTAGAAACAACAGCAAGGTGCCAAGATCGCATGTGCACACATGTAACCAGGCAGCTGCATCCAAGGACACGATTAACTGCTAAGCCTCCTGCAACTGAGCATCTCCTCGGTCAGCACATTCTCTTCCCGGGAAAGTGGCTCGAAGCCGGGTGGTGCTATGCAGTCCTCACCACACCCCG>Pika-ECR9TAATCTAAACTCCAACTTCCTTTTGCTCCCCTAAAGCCCACGGCTCTTGCTGGAGTTTCTCCACCAGGAACAACTGAAGCCTGCCAAGGTCATGTGCATACACGTGTATCCAAGGACATGATTAGCTGCTAAGCCCCCTGGATCTGTGCATTCCTTGGTCAGCACATTCTCTTCCCAGCATGGAGGCTGGAAGTCCAATGATGTTATGTAATCCCCACCACACCCCATGGGAGGCAAATG>Rabbit-ECR9ATTTTTGAATTAATCTAAACTCAACTTCCTTTTGCTACACCAAAGCCCACAACTCCTGCTGGCGTTTTTTCACCAAGAACAACTGAAGTCTGCCAAGGTCATGTGCATGCACGTGTATCCAGGCAGCTGCATCCAAGGACATGATTAGCTGCTAAGCCCCCTGGATCTATGCATTCCTTGGTCAGCACATTCTCTTCCCAGCAAGGAGGCTAGAAGTCCAATGATGTTATGTAATCCCCACCACACCCCT>Rat-ECR9CTGGCTACACTGAATCCCACAGCTTTTACTGATAGTTTTAAACCAAGGACAAACAGCACAGCCAAGACCAAGATCCTATGTGTGCACGTGTGTGCAGGCAGCTGCATCCAAGGACATGATCAGCCGTTAAGCCCCCTCTGGATCGATGCATCCCTTGTTCAGCACATTCTCTTCCCAGCAAAGAGGCTGGAGCCCCAACGGTGTTGTGTAATTCCCACCACACCCCAAGG>Sheep-ECR9AAATCTGCCAAAAGCCATGTGCACACACGTGTAATCCAGGCAGCTGTGTCCAAGGTCATGATTAGCTGCTAAGCCCCCCTGGATCTGTGCGTTCACTCGGTCAGCACATTCTCTTCCCGGGAAAGCGGCTGGAGGCCCAGTGGTGTGATGTAACTCTCACCACACCCCATG>White-rhinoceros-ECR9CCCAGCTTCCTCTTGCTACACCACAGCCCACAGCCCCTGCTGGCAGTTTTGCACTAAGAACAACCAACGGCTGCCAAGATCATGTGCGCACATGTGTATCCAGGCAGCTGCATCCAAGGACATGATTAACTGCTAAGCCCCCTGGATCTATGCATTACTTGGTCAGCACATTCTCTTCCCAGGAAAGCAGCTAGAAGCCTAATGATGTTATGAAATCCTCACCACACCCC>Mouse-ECR10CCTGAACATCACACATCTCCCAAGTGGAGGGGAATGAACTCCAGTGAAGGGTGGGTGGATTGGAAAGTTGGCCCCAAACAAGACAAAATAGCTTGATTTTTAACACATGGCCCAGTTTCCAGAGACACACTCAAGCTCATCAACCCACCATGGGATTTTTTTCCATCAGGCAGAAAAGCCAGGTTGATTCCAAGAAATATATACATTGCCGCACAATTAGGCAAAGGGAGAGAGTCCTTGGAGCCTTGGGGCCATGTGGTCAGTATTTGGGAGGCATCCACTTCCTGATGGTAAATAGATTTAAGCACGCCATCTGTTGTGTGCTTATTTCCCAGGACCTGTTTTCTTTCTCTAATTTTCAGTTCTGACAACCTTGTTTTCTTTTTCTTGACTTAAAAAAAAAAGTGACATAAACCAGTATTTGAGGGTTTTTATTTTTTCTCATGTGCTAGGCCTGATGAC>Alpaca-ECR10CCCAGTGTCATAGGGCTCCCAAGCAGAGGGGCATGGGTTTGAGCTAAGCAAAGGTAGATTCGAAAGTTGGTGCAAACGCGCGAGACAGTGTGATTGGATGTTTAACACATGGCCTCGTTTCCAGAGGCAAGTTCAACTCATCAACCTACCATGGGATTTTCCTCTAGGCTGAAAAGGCAGGTTTGATTCCAAAAAATATATACATTGCAGTGTAATTAAGCAAGGGAACTGCGTTCTCAGAGCCTCAGCGCCATGTGGTCAGTATTTGGGAGACATTCACTTCCTGACAGTAAACAGATTTAAACACACCGTCTGTTTTGCAGTTATTTCCCTGGACCTGTTTTTAGCCAGTTTCTCTAATTTTCAGGTCCTGACAGCTTTGTTTTCTTTTTCTTTCTTCTTCTTTTGAAAAAGACATAAACCAGTATTTGCTTTTATTTTTTCATGTGC>Armadillo-ECR10ACTATCTGCCTCTGCACCACAAGCTCCCTGAGAAGAGGAAAATGGATCTGAGCTAAGGATGAGCGGATTAGAAGGTTGGTGCAAGTCGAGACACAGCGGTTTGATTTTTAACACATGGCCCAGTTTCCAGAGGCAAATTCGTTCATCAGCCTACTATGGGATTTTCCCCTGGGCTGAAAACCCAGGTTGGAATCCAAGAAATATATACATTGCAGTGTAATTAGGCAAGGGTGCAGTGTTCTTGGAGCCGTAGCGACATGTGGTCAGTATTTGGGAGACATCCACTTCCTGACGGTAAACAGATTTAAATACCCCATCTGTTTTGCAGTTATTTCCCTGGACCTGTTTTTAGTCAGTTTCTCTCTAAATTTTCAGTTTCTGACAACTTCACTTTCTTTTTCTTTCTTGCTTTCTTTTTTTATGACATAAACCAGAATTTCTTTTATTTTTGTTCGTGTGCTAAGTTTGAT>Bonobo-ECR10TCCATATCACATGACTCCTGAGTAGAGGAAAATGGATTTGAGCTAAGGGCAGGTGAATTAAAAAGTTGGTGCAAATCAAGACAAAGCAGTTTGATTGTTGACACATGGCCCAGTTTCCAAAGGCAAACTCAGCTCATCAGCCTACTGTGGGATTTTCCCCTAGGCTGAAAAGCCAGGTTTGATTCCAATAAACATATACATTGCAGTGTAATTAGGCAAGGGAACAGGGGTCTTGGAGCCACAAAGCCCCATGGTCAGCATTTGGGTGACATCTACTTCCTGACTGTAAACAGATTTAAATACACCATCTGTTTTGCAGATATTTCTCAGAACCTGTTTTTAATCAGTTTTTCTCTCTAATTTTCGGATACTGACAATCTTGTTTTCTTTTTCTTTATGTTTTTTAAAAAATGACATAAACCAGTATTGCTTTTATTTTTTTCATGTGCT>Bushbaby-ECR10AGTTATTGCCCTCAATATCATGTGACTCCTGAATAGAAAGAAATGATCTGAACTAAGGGAAGGTGAATTAGAGAGTTGATGCAAACCTAGACAGGGCAGTTTGATTTTTAACACACAGCCCAGTTTCCAAACTCAAATCCAGCTCATCAACTTACCATGAGATTTTCCCCTAGGCTGAAAATCCAGGTCTGATTCCAAGAAATATATACATTACAGTGTGATTATTCAAGGGAGCAGTGTTCTTGGAACCACCAGGCCATGTGGTCGGTATTTGGGAGACGGCCACTTCCTGACAGTAAACAGATTTAAATACACCATCTGTTTTGCAGGTATTTCCCAGGACCTGTTTTTAGTCAGTTTCCCTCTCTAATTTTCAGGTTCTGACAAACCTTGTTTTCTTGTTCTTTACTTTTCAAAAAAAAAAAAAAAAAAGACATAAACCAGTATTTGCTTTTATTTTTTTTTTCTTGTGCTAGACTT>Cat-ECR10AATAGCAGCCTCAATATCACGTGACCCCTGAGTGCAGGGGAATGGGTTTGAGCTAAGGACGGGCTGACTGGAAAGTTGGTGCAAATCAAGGCAGAGGAGTTTGATTTTTAACACATGGCTCAGTTTCCAGAGGCACATCGGCTCATCAGCCTGCCGTGGGATTCTCCCTCCAGGCTGCAAGGCCAGGTTTGAGTCCAAGAAATATATACATCGCAGCATAATTAAGCAAGGGAATCGCGTTCTTGGAGCCACAGTGCCATGTGGTCAGAATTTGGGAGACATCTACTTCCTGACGGTAAACAGATCGAAGTATACACCATCTGTTTTGTAGTTATTTCCCTGGACCTGCTTTTAGTCAGTTTCTCTAATTTTCAGGTTCTGACAACCTTGTTTTCTTTTCCTTTCCTTCTTAAAAAGTGACATAAACCAGTATTTGCTTTTATTTTTTCATGTGCCAGCT>Chinese-hamster-ECR10CCTGGACATCACACATCTCCCAAGTGGAGGGGAATGAACTCCAGCTAAGGGTGGGTGGATTGGAAAGTTGGCCCCAAACAAGACAAAATAACTTGATTTTTAACACATGGCCCAGTTTCCAGAGACACACTCAGGCTCATCAACCCACCATGGGATTTTTTTTTTCCAACCAGGCAGAAAAGCCAGGTTGATTCCAAGAAATATATACATTGCCGTACAATTAGGCAAAGGGGAGAGAGTCCTTGGAGCCTTGGGGCCATGTGGTCAGTATTTGGAAGGTATCCACTTCCTGACGGTAAATAGATTTAAGCCCACCATCTGTTGTGCAGTTATTTCCCAGGACCTGTTTTCACTCAGTTTCTCTCTCTAATTTTCAATTCTGACAACCTTGTTTTCTTTTTCTTGACTTAAAAAAGTGACATAAACCAGTATTTGAGGGTTTTTTTTTTCATGTGCTAGGCCTGATGATT>Dog-ECR10AACTAGGGATGGGCGGTCTGGAAAGTTGGTGCACAGCAAGACGGAGCCGTTTGATTTTTTAACACATGGCTTAGTTTCCAGAGGCAAATCAGCTCATCAACCTACCATGGGATTTTTTCCTTCTAGGCTGCAAGGCCAGGTTGATTCCAAGAAATATATATATCGTGGTGTAATTTAGCAGCGGAACAGTGTTTCTGGAGCCAGGGTGCCATATGGTCAGTATTTGGGAGACATCTACTTCCTGACAGTAAACGGATTGAAATATACCATCTGTTTTACAGTTCTTTCCCTGGACCTGTTTCTAGCCAGTTTCTGTAATTTTTCAGGTTCTTACAACCTTGTTTTCTTTT>Dolphin-ECR10GGGTGTCAGCCTCCATGTCACATGCCTCCCGAGCAGAGGGAAAGGATGGATGGATTGGAAAGTTGGTGCAAACTGAGACAGCGCAGTTTGATTTCTAACACATGGCCCAGTTTCCAGAGGCAAGTTCAGTTCATCAACCTATCATGGGATTTTCCTCCAGGCTGAAAAGCCAGGTTGGATTCCAAGAAATATATACATTGCCGCGTAATTAAGCAACGGAACTGCGTTCTAGGAGCCTCAGCGCCCTGTGGTCAGTGTTAGGAAGACATTCACTTCCTGACAGTAAACAGATTTAAATACACCCTCTGTTTTGCAGTTATTTCCCTGGACCTGTTTTTAGTCAGTTTCTCTAATTTTCAGGTTCTGACAACCTTGTTTTCCTTTTCTTTCTTTTTTTTTTTTGAATGATGTAAAACAGTATTTGCTTTTATTTTTTTCATGTGCCAGGTT>Elephant-ECR10CAGCACAGTTTGATTTTTAACAGATGGCCTCAATTCTAGAGGCAAATTCACCTCATCAACCTACCATGGGACTTTCCCCTAGGGTGAAAAGCAAGGACCAATTCCGAGAAACATATACATTGCAGTGTAATTAGGCAAGGGGACAATGTTCTTGGAGCCACAGCAGTGTGGTCAGTATTTGGGAGAGATTCACTTCCTGACGGTAAGCAAATCTGAAGACACTATCTGTGTTGCAGTTATTTCCCTGGACCTGTTTTTAATCAAGTGTCAGATTCTGACAATGTCCTGTTTTCTTCTTTCTCTCTT>Ferret-ECR10GGTATCAGCCTCAATATAACATGACTCCTGAGTGGAGGGAAATGAGTTTGAGCTAAGGATGGGTGGACTGGAAAGTTGGTGCAAATCAAGACCGAAAGGTTTGATTTTTCACACACGGCTCAGTTTCCAGAGGCAGATCAGCTCATGAGCCTACCATGGGATTTTTCCCTCTGGGCTGCAAGGCCAGGTTTGATTCCAAGAAATATATACATCGTGGTGTAATTAAGCCAGGGAACGATGTTTTTGGAGCCGGGGTGCCATGTGGTCAGTATTTGGGAGACATCTACTTCCTGACGGTAAACAGATCGAAATACACCATCTGTTTTGTAGTTACTTCCCTGGACCTGCTTTTAGTCAGTTTCTCTAATTTTGAGGTTCTGACAACCTTGTTTTCTTTCTC>Gibbon-ECR10TGACTTCTGAGTAGAGGAAAATGGATTTGAGCTAAGGGCAGGTGAATTAAAAAGTTGGTGCAAATCAAGACAAAGCTGTTTGACTGTTAACACATGGCCCAGTTTCCGAAGGCAAACTCAGCTCATCAGCCTACTGTGGGATTTTCCCCTAGGCTGAAAAGCCAGGTTTAATTCCAATAAACATGTACATTGCAGTGTAATTAGGCAAGGGAACAGGGTTCCTGGAGCCACAAAGCCCCATGGTCAGTATTTGGGTGACATCTACTTCCTGACTGTAAACAGATTTAAATACACCATCTGTTTTGCAGATATTTCTCAGAACCTGTTTTTAATCAGTTTGTCTCTCTAATTTTCGGGTACCGACAATCTTGTTTTCTTTTTCTTTATGTTTTTTAAAAAATGACATAAACCAGTATTGCTTTTATTTTTTTCATGTGCTT>Gorilla-ECR10TGACTCCTGAGTAGAGGAAAATGGATTTGAGCTAAGGGCAGGTGAATTAAAAAGTTGGTGCAAATCAAGACAAAGCAGTTTGATTGTTAACACATGGCCCAGTTTCCGAAGGCAAACTCAGCTCGTCAGCCTACTGTGGGATTTTCCCCTAGGCTGAAAAGCCAGGTTTGATTCCAATAAACATATACATTGCAGTGTAATTAGGCAAGGGAACAGGGTTCTTGGAGCCACAAAGCCCCATGGTCAGTATTTGGGTGACATCTACTTTCTGACTGTAAACAGATTTAAATACACCATCTGTTTTGCAGATATTTCTCAGAACCTGTTTTTAATCAGTTTTTCTCTCTAATTTTCGGATACTGACAATCTTGTTTTCTTTTTCTTTATGTTTTTAAAAAAATGACATAAACCAGTATTGCTTTTATTTTTTTCATGTGCTTGGCTTGATTA>Horse-ECR10GATCGCATGACTCCCGAGGAGAGGAACGTGGATTTGAGCTAAGGATGAGCGGATTAGAAAGTTGGTGCAAATCAAGACAGACCAGTTTGATTTTTAACACATGGCCCCATTTCCAGAGGCAAATTCAGCTCATCAACCCACCATGGGATTTTCCTCTAGGCTGAAAAGCCAGGTTTGATTCCAAGAAATATATACATTGCAGTGTAATTAAGCAAGGGAACTGTGTTCTTGGAGCCACAGAGCCATGTGGTCAGTGTTTGGGAGACATCCACTTCCTGACCGTAAACAGATGTAAACCCACCATCTGTTCTGCAGTGACTTCCCTGGACCTGTTTTCGGTCAGTTTCTCTCTCTAATTTTCAGGTTTTGACAACCTTGTTTTCTTATTCTTTCCTTTTTTAAAAATGACATAAACCAGTATTTGCTTTTATTTTTTCATGTGCCAGGTTT>Manatee-ECR10GTAAAGGAAAATGTATTTGAGCAAAGGCTGGGTGGACTAGAGAGTGGGTACAAACCAAGACAACAGAGTTTGATTTTTAACACGTGGCCCCACTTCTAGAGGCAAATGCAGCTCATCAACCTACCATGGTACTTTCCGCTAGGCCAAAAAGCCAGGCTTGATTCCAATAAACATATACATTGCAGTGTAGTTAGGCAAGGAAACAATGTTCTTGGAGCCACAGCATGGTGTGGTCATTATCTGGGAGACATTCACTTCCTGAAGGTAAACAAATTTAAAGACACCATCTGTGTTGTAGTTATTTCCCTGGACCTGTCTTTAGTCAAGTTTCTTTTATTTTCAGACTCTGACAATGTCCTTTTCTTTTTCTTTCTTTCTTTTTTTAGTGACATAAACTAGTATTTCTATTTGCTTTAGCTAATCTACAATC>Marmoset-ECR10GCTAAGGGCAGGCGGATTAGAAGGTCGGTGCAAATCAAGACAGAGCAGTTTGATTTTTAACACATGGCCCCGTTTTCCAAAGGCAAACTCAGCTCATCAGCCTACCGTGAGATTTTTCCCCTAGGCTGAAAAGCCAGGTTTGATTCCAAGAAATATATACATCACAGTGCCGTTAGGCAAAGAACAGGGTTCTTGGAGCCACAAAGCCTCATGGTCGGTATTTGGGAGACATCTACTTCCTGACCGTAAACAGATTTAAATACACCATCTGTTTTGCAGCTATTTCTCAGAACCTGTTTTCAGTCAGTTTTTCTCTCTAATTTTCGGGTTCTGACAATCTTGTTTTCTTTTTCTTTATGTTTTAAAAACATGACATAAGCCAGTATTGCTTTTTTTTTCTTTTTTTCATGTGCTCGGCTT>Megabat-ECR10ACATGACTCCGGAATAGAGAAAAATGCATTTGAGCTAAGGATGAGTAGGCTGGAAAGTTGGTGCAAATCAAGACAGAACAGTTTGAGTTTTAACAGATGGCCCAGTTTCCAGAGGCAAATTCAACTCATCAACCTACCCTGGGAATTTCCTCTGGGCTGAAAAGCCAGGTTTGATTCCAAGAAATATATACATTGCAGTGTGATTAAGCAAGAGAGCAGTGTTCTTGGAGCCACAGCACCATGTGGTCAGTATTTGGGAGACATCCACTTCCTGACAGTAAACAGATTTAAATGCACCATCTGTTTTGCAGTTATTTTTCTGGACCCATTTTTAGTCAGCTTCTCTCTCTAATTTTCAGGTTCTGACAACCTTTTTTTCTTTTTCTTTTTGTTCCTTAAA>Minke-whale-ECR10AATACCAAGGGGTGTCAGCCTCCATGTCACATGCCTCCCGAGCAGAGGGAAAGGATGGGTGGATTGGAAAGTTGGTGCAAACCGAGACAGCGCAGTTTGATTTCTAACACATGGGCCAGTTTCCAGAGGCAAGTTCAGCTCATCAACCTATCATGGGATTTTCCTCCAGGCTGAAAAGCCAGGTTGGATTCCAAGAAATATATACATTGCCACGTAATTAAGCAAGGGAACTGCGTTCTAGGAGCCTCAGTGCCCTGTGGTCAGTGTTAGGAAGACATTCACTTCCTGACAGTAAACAGATTTAAATACACCCTCTGTTTTGCAGTTATTTCCCTGGACCTGTTTTTAGTCAGTTTCTCCAATTTTCAGGTTCTGACAACCTTGTTTTCTTTTTCTTTCT>Mouse-lemur-ECR10CTCCTGAAAGCAGGAAACGGATGTGAGTGAATTAGGGAGTTGGTGCCAGCCAAGACAGAGCAGTTTGATTTTTAACGCAGGGCCCAGTTTCCAAAGGCAAATTCAGCTCATCAGCCTACCATGAGACTTTCCCCTTAGGCTGAAAAGCCCAGTTTGAGTCCAAGAAACGTATACATTGCGGTGTGATTAGGCAAGAAGCAGAGTTCTTGGAGCCGCCAGGCCATGCGGTCAGTATTTGGGAGACAGCCACTTCCTGACAGTGAGCAGATTTAAATACACCATCTGTTCTGCAGTTGTTTCTCAGGACCTGTTTTTAGTCGGTTTCTCCAATTTTCAGGTTCGGACAACCTTCTTTTTCTTTACTTTTCAAAAAATGACATAAACCAGTATGTGCTTTTATGTTTTTCATGCGCAACGCTTGATTGTTTTAAGCTAATTCA>Panda-ECR10GCATGACTCCTGAGTGCAGGGAAATGGGTTGGAGCTAAGTCTGGGAGGACTGGAAAGTTGGTGCAAATCAAGACAGAGCCGTTTGATTTTTAACACATGGCTCAGTTTCCAGAGGCAAATCAGCTCATCAACCTACTATGGGATTTTTCCCTCTAGGCTGCAAGGCCAGGTTTGATTCCAAGAAATATATACATCGTGGTGTAATTAAGCAAAGGAACAGTGTTTTTGGAGCCAGAGTGCCATGTGGTCAGTATTTAGGAGACATCTACTTCCTGACGGTAAACAGATCAAAATACACCGTCTGTTTTGTAGTTATTTCCCTGGACCTGTTTAGTCAGTTTCTCTAATTTTCAGGTTCTGACAACCTTGTTTTCTTTTTCTTTCCTTCTTAAAAAATGAC>Pig-ECR10GTGCAAACCCAGACAGCGCAGTTTGATGTTTAACACGTGGCCCCAGTTCCAGAGGCCAAGTCAGCGCGTCCACCTACTCTGGGATCCTCCCGCGAGCTGCAAAGCCAGGTTTGAGTCCAAGAAATATATACATTGCCATGTAATTAGGCGAGGGAGCGATGTTCCTGGGGCCTCCGTGCTGTGCGGTCAGTGTTTGGAAGACTTTCACTTCCTGATGGTAAACAGATTTAAAGACACCATCTGTTTTGCAGTTATTTGCCCGGATCTGTTTTCAGTCAGTTTCTCTCATTTTCAGGTTCTGACAACCTTGTTTTCTTTTTCTTTCCTCTTTTTTCTTTCTTTTTTTTTTTTTTAAATGCTATACGCCAGCATTCGCTCTTATTCCTTTCATGTGCCAGGT>Pika-ECR10CTCAGGATTGTTCCCTGAGACTGAAAAGTCAGGTTTGAGTCCAGAAAGTCTGTACATTATAGGACAGTTGGGCATCGGGTCAGCACTGGGGGAAGCACGCGGCCATGTGGTCAGTGTTTGGGAGACATCCACTCCCTGGTGGTAAACACGTTCAGATTCACCATCTGCTTTGCAGTTACTGTTGAGGACCTGTTTTAAGTCAGTTTCTTTCTCTAATTTTCAGGTTCTGACAACCTTGTTTTCTTTTTCCTCACTTAAAAAAAATGGCACACACCAGTATTCATTTTTTCATGTCTGAGA>Rabbit-ECR10TTTTGTGGATAACAGCATGGCTGTGAGAACAAAAAGAATCAACCCAGTGTCACATAACTCCCAAGTTGAGGGATATGGCCTTGCACTAAGATTGGGCAGGTTGGAAAGTTGTTGGAAATAAGACAGAGCGGTTTGACTTTTAACACATGGCCCAGTTTCCAAAGGCACAGTCGACTCATCAGCCTGCCATGGGATTGTTCCCTTAGGCTGAAAAGCCAGGTTGATTCCAAAAAATACATACATTGTAGTGCAATTAGGCTTGGGAACAGCACTCACGGAGCCATATGGCCATGTGGTCAGTATTTGGGAGACATCCACTTCCTGGTGGTAAACACATTTATATTCACCATCTGTTTTGCAGTTATTGCCCAGAACCTGTTTTAAGTCAGTTTCTCTCTTAATTTTCAGGTTCTGACAACCTTGTTTTCTTTTTCTTTACTTTTCAGGAAAAAAAAAATGACACACACCAGTATTTGCTTTCATTTTTTCATGTCTGAGAC>Rat-ECR10CCTGGACATCACACATCTCCCAAGTGGAGGGGAATGAACTCCAGTTAAGGGTGGGTGGATTGGAAAGTTGGCCCCAAACAAGACAAAATAGCTTGATTTTTAACACATGGCCCAGTTTCCAGAGACATACTCAAGCTCATCAACCCACCATGGGATTTTTTTCCACTAGGCAGAAAAGCCAGGTTGATTCCAAGAAATATATACATTGCCGCACAATTAGGCAAAGGGAGAGAGTCCTTGGAGCCTTGGGGCCATGTGGTCAGTATTTGGGAGGCATCCACTTCCTGACGGTAAATAGATTTAAGCATACCATCTGTTGTGTGGTTATTTCCCGGGACCTGTTTTCTTTCTCTAATTTTCAGTTCTGACAACCTTGTTTTCTTTTTCTTGACTTAAAAAAAGTGACATAAACCAGTATTTGGCTTTTTTTTTTCCTCATGTGCTAGGCCTGATGA>Rhesus-ECR10GAAAGTTGGTGCAAATCAAGACAAAGCAGTTTCATTGTTAACACACGGCCCCGTTTCCCAAGGCAAAACAGCTCATCAGTCTACTGTGGGATCTTCCCCTAGGCTGAAAAGCCAAGTTTGATTCCAACAAATATATACATTGCAGTGTAATTAGGCAAGGGAACCGGGTTCTTGGAGCCACAAAGCCCCATGGTCAGTATCTGGGAGACAGCTACTTCCTGACTGTAAACAGATTTAAATACACCATCTGTTTTGCAGATATTTCTCAGAACCTGTTTTTAGTCAGTTTTTCTCTCCAGTTTTCGGATACTAACAATCTTGTTTTCTTTTTATGTTTTTTTAAAAGTGACATAAACCAGGGTTGCTTTTATTTTCTTCATGTGCTTGGCTTGATTATTTG>Rock-hyrax-ECR10TAGTATGGACTTCGACATCACATGACTTCTGAGTAAAGAAAATGGATTTGAGTGAAGGCCATGCGAACCAGAGACTTGGTAGAAACCAAAACAGCACAGTTTGATTTTTAACATATGGCCCCATTTCCAGAGGCAAATTTAGCTCATCAACCTACCATGGGACTTTCCCGTAGGCCAAAAGCCAGGCTTGATTCCAAGAGACATATACATTGCAGTGTAATTATGCAAGGGAACAATGTTCTTGGCGCCACAGCACAGTGTGGTCAGTATTTGGGAGACACTCACTTCCTGACAGTAAACAAATTTAAAGACACCATCTGTGTTGCAGTTATTTCCCTGGACCTGTTTTTAGTCAATTTTCTCTCTCATTTTCAAATTCT>Sheep-ECR10AGCTGACCTCAGGATCACAGGACTCCTGAGCAGAGGAGAGGGGACTTGAGCTGAGGATGGATGGATTCGAAAGGCGGTGCAAACACTGAGGCAGCGCGGGTTGATTTTTAACACATGGCCCGGTTTCCAGAGGCAAGTTCAGCTCATCAGCCTACCATGGGATTTCCCTCCAGGCTGGAACGCCAGGTTGGATTCCAAGAAATATATACATTGCAGTGTAATTAAGCAAGGAAACCGTGTTCTTGGAGCCCGGTGCCGTGTGATCGGCTTTTGGGCCACATCGACTTCCTGGCAGTAAACAGATTTAAGTACACCATCTGTTTCGCAGTTATTCCCCTGGACCTGCTTTTAGTCAATTTCTCTCCTTTGCAGGTTCTGACAACCTTGTTTTCTTTTTCTCTCCTTTTTCTTTTTTTTTTAAGCTGATGTAAACCAGTATTTGCTTTTTTTCCCCCCATGTGCCAGGTTAG>Sloth-ECR10TCCACCTCAACGTCACACGACTCCTGAGAAGAGGAAAGCGGATTTGGGCTAAGGATGGGTGGATTGGAAAGTTGGTACAAATCGAGACAGAGCAGTTTGATTCTTAACACATGGCCCAGTTTCCAGAGGCAAATTCAGTTCATCAACCCACCGTGGGATGTTCCCCTGGGCTCACAGGCCAGGCTGGGATCCAGGAATTACATACATCACTGTGCAGTTAGGCGAGCGCCCAGCGTTCCTGGAGCCACAGAGGCATGTGCTCAGGGTTTGGGAGACTTCCACTTCCTGACGGTAAACAGACTTAAATGCACCATCTGTTTTGCAGTTTTTCCCTTGGACTTGATTTGAGTCAGTTTCTCTCTAATTTTCAGGTTCTGACAGCTTCATTTTCTTTTCTCTCTTTTTTTTTAAATGACATAAACCAGTTTTTTGCTTTTATTTTTTCATGGG>Squirrel-ECR10TATCGGCCTGGCTATCCCACGCCTCCCGAGTGCAGGAAAGGGATTTGAGCTGGGGGTGGGGGGGTGATTGGAAAGCTGGTGCGAATCAAGACGGAGCAGTTTGATTTTTAACACATGGCCCAGTTTCCAAAGGCGAATTCGGTTCATCAACCCACCATGGGATTTTCCCCTAGGCTGAAAAGCCAGGTTTGATTCCAAGAAATATATACATTGCAGTGTAATTAGGCCAGGGACGAGCATTGCTGGAGCCGCAGAGCCATGTGGTCAGCGTTCAGGCGACATCCACTTCCTGGTGGTGAGCACGTCTAAATGCACCATCTGTTCCGCAGAGACTCCCCGGACCTGTTTTCAGTCAGTCTTTCTCTCTGATTTTCATTCCTGACAACCTTGTTTTCTTTTTCTTTACTTTTTGAAAAATGACATAAAGCAGTATCTGCCTTTGTTTTTTCA>Squirrel-monkey-ECR10ATGCAAATCAAGACAGGGCAGTTTGATTTCTAACACACGGCCCTGTTTTCCAAAGGCAAACTCAGCTCATCAGCCTACCGTGAGATTTTTCCCCTTGGCTGAAAAGTCAGGTTTGATTCCAAGAAATATATACATTGCAGTGCAATTAGGCAAAGGAACAGGATTCTTGGAGCCACAATGCCCCGTGGTCAGTATTTGGGAGACATCTACTTCCTGACCATAAACAGATTTAAATACACCATCTGGTTTGCAGCGATTTCTTGGAACCTGTTTTCAGTCAGTTTTTCTCTCTAATTTTCGGGTTCTGACAATCTTGTTTTCTTTTTCTTTATGTTTTAAAAACAT>Tarsier-ECR10AGTGGGTTTTGAGCTAGGGGCAGGCGAGTTAGAAAGTTGGTGCAAATCACGGCAGGGCTGTTTGATGTTTAACACACAGCGGGGTTTCCAAGGCAAATTCAGCTCATCAATCCACCATGCCATTTCCCCCAGGTTGAAAAGCCAGATTGGATTCCAAGAAATATATACATTGCAGTGTAATTAGGCAAGGGAACGGTGCTCTTGGAGCCACAGAGACCTGTGGTCAGTATTTGGGAGACAGTCACTTCCTGACGGTAAACAGATTTAAATACACCATCTGTTTTGCAGTTATCTCCTGGGACCTGTTTTTAGTCAGTTTCTCTCTCTAACTTTCAGGTTTTGACAACCTTGTTTTCTTTTTCTTAGAAAGAAAAAAAAAAGAAGACATAAACCAGATGCT>White-rhinoceros-ECR10ATCACGTGACTCCTGAGGCGAGGAACACGGATTTGAGCTAAGGATGAGCAGATTGGAAAGTTGGTGCAAATCAAGACAGAGCAGTTTGATTTTTAACACATGGCCCCGTTTCCAGAGGCAAATTCAGCTCATCAACCTGCCATGGGATTTTCCTCTAGGCTGAAAAGCCAGATTTGATTCCAAGAAATATATACATTGCAGTGCAATTAAGCAAGGGAATGGTGTTCTTGGAGCCACAGCGCCATGTGGTCAGTGTTTGGGAGACATCCACTTCCCGATGGTAAACACATTTAAACACACCATCTGTTTTGTAGTTACCTCCCTGGACCCATTTTTGGTCAGTTTCTCTCTCTAACTTTCAGGTTCTGACAACCTTGTTTTCTTTCTCTTTCCTTTTTTTAAAATGACATAAACCAGTATTTGCCCTTATTTTTTCGTGTGCCAGGCTTG>Mouse-ECR11GGTGGGGGAAGCAAAGCATCTCAGAACACTAGCTGTTAGGATGTCAGTTTCATCAGGTTGCCAAGTAAAACCCCACAGCAACTGCCCACCATGGAATTTTCCCTGATTAATGACCTACGAATGCCCGGCTGGCGTCCAAGCTCTATATACATTGACATCCAACTCAGGAAGGGAAACAATTTTCCCAGAAACAGCACAACATGGCCACTATTCTTCAGATGTCCGCTTCTAAAACCATAAAAATATTTCATAGTAGCCGTCTGTTTGTGGACCATATCCCTGGGTCGTGGACATAAGTGGGTTTTCTCTGGTGTTCTGGGAGGGTGGACTGTCCTCATCTGTAGATGACTTGAATGCCTGTTCCCAGGGCCCTTGCAGCCCATGTGCCACACTCTTCCTGTTCAGTGTTGGCACAGAGCGGTAACCTACTTGAAGACGTGTGGCTTGAACTACATTGTATAAACATTGAAATAATAAGTCAAACAAGCAAGTACATTTTCACTCTTGTGCCTCTGTACCCAACTTTATAAATCTCCAAATGTGTGCATTCAAAGGAGCTGTGTGTTCTCCCCCAATAGCTGCCTTGTGTAACAGTAAACACCATTATGCAAACATGACTGC>Human-ECR11ACTAGCTGTTAGGATGTTCAGCTTGGTCCAGTTGCCAAACAAAACCCCAGGGCAACTACTCACCACGGAATTTTCCCCCAATTAATAACCTACGAATGCCCGGCTTGAGTCCAAGCTCCATATGCACTGACACCCAACTCAGGAAGGCAACGGTTTTCCCAGAAACAGCACAACATAGCCAGTAGTTGGGAGACAACCATTTCTAAACCATAGAAAAGACTTCATACTAGCTATCTGTTTATAGATTGTGTTCCTGGGTCATGTCCATAACTGGATTTTCTCTGGTGTTCTGGGAGCAAAAATCTTCTGTCTTCTTTTTGAGGTGAGTAGAGTCTTCCAAATGTCCTTCATGGCCCCAGCCCACCGTTTTCTTTATGTAGGAAGGTTTTAGATGTTGGCACATATTGACACCTACTTGCAGACAGGTTTGTTGAATTACATTATATAAACGTTGAAATAATAACTCAAGCAGTGAGTACATTTTCACTCTTACTCCCCTGCACCTAGCTTTATAAATCGCTAAATACATGCATTCAGAGAATCTGTGTGC>Alpaca-ECR11TTAGGATGTCAGCGTGGTCCCAGGTGCCAAATAAAGCACCGAGGCAGCCACCCACCGTGGAATTTCCCACTGATTAATGACCCCCGCGTGCCCGGCTCGAGTCCAAGCTCCATATACATTGACATCCAGCGCAGGAAGGGAGCATTTTTTCCCAGAAACGCACAAATGCCCAGTATTCCAGAGACGTCCATTTCTAAACCATAAAAATATTTGACATGAGCCATCTGTTTATGGATTCTATCCCTGGGTCATGTTCGTAACCGGGTTTTCTCCGGTTTGGGGGGTAAAATCTTGTGTCCCTGTTTGGAGGTGAGTTGAGCGTTTGTTCCAAATGCCCTTGATGGCCCGTCGTCCTCAGGTTTCCTTGTGTACGAAGGTCTTAGTTGTTGGCGTGTAAAGACCGTCTACTTCCAGGTGTGTTTGTTGAACTAAGTTACATAAACATGAGGATAGTAACTCAAACAGCGAGTACATTTTCACTCTATTCCTCTGGACCCAGC>Baboon-ECR11ACTAGCTGTTAGGATGTTTGGCTTGGTCCAGTTGCCAAACAAAACCCCAGGGCAACTACCCACCGTGGAATTTTCCCCTGATTAATAACCTACGAATGCCCGGCTTGAGTCCAAGCTCCATATACATTGACATCCAACTCAGGAAGGGAACAGTTTTCCCAGAAACAGCACAACATAGCCAGTATTTGGGAGACAACCATTTCTAAACCATAAAAAGGACTTCCTCGTAGCTCTCTGTTTATAGATGGTGTTCCTGGGTCACGGCCATAACTGGATTTTCTCTGGTGTTCTGGGGCAAAACTCTTCTGTCCTCTTTTTTAGATGAGTTGAGTGTTCCAACTGTCTTTCATGGCCCCAGCCCACCGTTTTCTTTATGTAGGAAGGTTTTAGATGTTGGCACACATTGACACCTACTTGCAGACAGGTTTGTTGAATTACATTACATAAATGTTGAAATAATAACTCAAACAGTGAGTACATTTTCACCCTTATTCCCCTGCCCCTAGCTTTATAAATCTCTAAATACATGCATTCAGAGAATCTGTGTGCTTTTCCCAACAGCTATGTTGTGTAATGGTGAACATCATTATGCAAACACAG>Bonobo-ECR11ACTAGCTGTTAGGATGTTCAGCTTGGTCCAGTTGCCAAACAAAACCCCAGGGCAACTACTCACCACGGAATTTTCCCCCATTTAATAACCTACGAATGCCCGGCTTGAGTCCAAGCTCCATATGCACTGACACCCAACTCAGGAAGGGAACGGTTTTCCCAGAAACAGCACAACATAGCCAGTAGTTGGGAGACAACCATTTCTAAACCATAGAAAAGACTTCATACTAGCTATCTGTTTATAGATTGTGTTCCTGGGTCATGTCCATAACTGGATTTTCTCTGGTGTTCTGGGAGCAAAAATCTTCTGTCCTCTTTTTGAGGTGAGTAGAGTGTTCCAAATGTCCTTCATGGCCCCAGCCCACCGTTTTCTTTATGTAGGAAGGTTTTAGACGTTGGCACATATTGACACCTACTTGCAGACAGGTTTGTTGAATTACATTACATAAACATTGAAATAATAACTCAAACAGTGAGTACATTTTCACTCTTATTCCCCTGCACCTAGCTTTATAAATCGCTAAATACATGCATTCAGAGAATCTGTGTGCTTTTCTCAACAGCTATGTTGTGTAATGGTGAACATCATTATGCAAACACA>Cat-ECR11TCCCGGGTGCCAAATAAAACCCTAGGGCAGTCACCTACCGTGGACTTTCCCCCTGATTAATGACCTACGAGTGCCAGGCTTGGGTCCAAGTTCTATAGACATTGACATCCGACTCAGGAAGGGAACGGTTTTCCCAGAAACAGCACAACATGGCTGTTGTAGCCCCATGTATTCTGGGAGGGGGGGGAACGTCCATTTCTAAACCATAAAAATATTTAATACTAGCCATCTGTTTGTAGATAATATCCCTGGGTCCTGGTTGTCACTCTGGTGTTGGGGGGACGGGGGTGGCGCGGAATC>Chimp-ECR11ACTAGCTGTTAGGATGTTCAGCTTGGTCCAGTTGCCAAACAAAACCCCAGGGCAACTACTCATCACGGAATTTTCCCCCATTTAATAACCTACGAATGCCCGGCTTGAGTCCAAGCTCCATATGCACTGACACCCAACTCAGGAAGGGAACGGTTTTCCCAGAAACAGCACAACATAGCCAGTAGTTGGGAGACAACCATTTCTAAACCATAGAAAAGACTTCATACTAGCTATCTGTTTATAGATTGTGTTCCTGGGTCATGTCCATAACTGGATTTTCTCTGGTGTTCTGGGAGCAAAAATCTTCTGTCCTCTTTTTGAGGTGAGTAGAGCGTTCCAAATGTCCTTCATGGCCCCAGCCCACCGTTTTCTTTATGTAGGAAGGTTTTAGACGTTGGCACATATTGACACCTACTTGAAGACAGGTTTGTTGAATTACATTACATAAACATTGAAATAATAACTCAAACAGTGAGTACATTTTCACTCTTATTCCCCTGCACCTAGCTTTATAAATCGCTAAATACATGCATTCAGAGAATCTGTGTGCTTTTCTCAACAGCTATGTTGTGTAATGGTGAACATCATTATGCAAACACA>Chinese-hamster-ECR11TCTCTGAACACTAGGCTGTTAGGATGTCAGTTTGGTCGAGCTGCCAAGTAAAACCCCACAGCAACTGCCCACCATGGAATTTTCCCTGATTAATGACCTATGAAGGCCAGGCTGCTGTCCAAGCACTATATACGTTGACATCCAACTCAGGAAGGGAAAGTATTTTCCCAGAAACAGCACAACATGGCCACTATTCAGCTGTCTGCTTCGAAACCATAAAAATATTTCATACTAGCTGTCTGTTTGTAGACCATATCCCTGGGTCGTGATTCCCATGGAAAAACTCAAGCCTGCAGAACGATCGATCCCTATCGGGGTAAGGCGGAGGTGAACGGTCGTCATTTTGAGATGAGTCGAGCGTTTGTTCCCAATGCCCTTTGCAGCCCATGCACCGCAGTTTCCCTGCTCAGGGTTGGCACGCAGTGGTAACCTGCTTGCAGACATGTGTATTGAACTACATTCTATAAACATTGAAATAAGAAGCCAAACAAACAGGGACATTTTCACCCTTATACCTCCGTCCCCCAACTTTATAAGCCTCTAAATGCATGCATTCAGAGGACTGTGTGTGTGTGTGTGTTTTCCCCAATAGCCGCCTTGTGTAACAGAGAGCACCATTATGCAAACATGGCTGCCTGGCAGAATCTATA>Dog-ECR11ACCATGGGCCTCCCCCCTGATTAATGACCTCTGAATGCCAGGCTATAGGCCAAGATCTATAGACATTGACATCCAACTCAGTAAAGGAACAGTTTTCCCAGAAACAGCACAACAGGCCAGTATTCTGGGGAAGTCCATTTCTAAGCCATAAAAATATTTAATACCAGCCATGTGTTTCTAGATAATGTACCTGGGTCATGTTTGTAGCTCTGGTGTTCTGGGGGGAAAATCTTGTGTCCCTGTTTTGAGATGATCGTTGAAGGCTTGTTCCAAAAGCCCATCGTGGCCTGTGGTCCACACTTTTCCTTATGTATGAAGGTTTTAGATGTTAGCACATAATAGGAATCTAATTCCAGATGTGTTTGTTGAACTAAATATACAAACACTAGAATAATATCTCAATAATGAGTACATTTTCACCCTTATTCCTCTGTATCTAACTTTGTAAGTCCCTAAATGCATGCATTCAAAGGGGACTGTGTGCCTCTTCCCAATAGCTGCCTTGTGTAATGGAATATGATTGCACAACCATGGCTGCAAGGCAAGCTCC>Dolphin-ECR11AAGCAAATCATCTCTGAACACTAGCCGTTAGGATGTCAGCTTGGTCCCAGTTGCCAAATAAAATGCCAGGGCAGCCACCCACCATGGAATTTCCCCTGATTAATGACCTACAAATGCCAGGCTCAAGTCCAAGCTCCATATACATCGACATCCAACTCAGGACAGGAACCGTTTTGACAGGAACCGTTTTCCCAGAAACCGCACAAATGCCCAGTATTCTGGAGACGTCCATTTCTAAACCATAAAAATACTTAACATTAGCCATCTGTTTATAGATTATATCCCTGGGTCATGTTCATAACTGGGTTTTCTCTGGCTTTCTGGGGTGAAAAATCCTGTGTCCTTGTTTTGAGATGCGTTGAGTGTTTCTTCCAAATGCCCGTGATGACCCGTGGTCCACCGTTTTCCTTATGGATGATGGTTTTAAATGTTGGCACATAACGGCAATCCACTTCCAGGTGTGTTTGTTGAGCTAAGGTACATAAACATTGGGATAATAACTCTAACAGTGTGTACATTTTCACTCCTAGTCCTCTGTACCTAACTTTATAAATCCCTAAATACATGCATTCAAAGGAGTTGTTTGCTTTCTTTCCAAGGGCCGTCTTGTGTAACAGCGAACATCATTGTGCAAACACCCCTGCAAGGCA>Ferret-ECR11CTGTTAGGATGTCAGCTTGGCTTCAGTTGCCAAATAAAACCATAGGGCAGTCACCTACCATGGACTTCTCCCCTGATTAATGACCTCCGAATGCCAGGCATGAGTCCAAGTTCTATAGACGTTGACATCCAACTCAGGAAGGGAATGGTTTTCCCAGAAACAGCACAACATGGCCAGTATTCTGGGGACATCCATTTCTA>Gibbon-ECR11ACTAGCTGTTAGGATGTTCAGCTTGGTCCAGTTGCCAAATAAAACCCCAGGGCAACTACCACCACGGAATTTTCCCCTGATTAATAACCTACGAATGCCCGGCTTAAATCCAAGCTCCATATGCATTGACACCCAACTCAGGAAGGGAACGGTTTTCCCAGAAACAGCACAACATAGCCAGTAGTTGGGAGACAACCATTTCTAAACCATAAAAAAGACTTCATACTAGCTATCTGTTCATAGATTGTGTTCCTGGGTCATGTCCATAACTGGGTTTTCTCTGGTGTTCTGGGGGCAAAAATCTTCTGTCCTCTTTTTGAGATGAGCTGAGTGTTCCAAATGTCCTTCATGGCCCCAGCCCACTGTTTTCTTTATGTAGGAAGGTTTTAGATGTTGGCACATATTGACACCTACTTGCAGACAGGTTTGTTGAGTTACATTACATAAACGTTGAAATAATAACTCAAACAGTGAGTACATTTTCACTTATTCCCCTCCACCTAGCTTTATAAATCTCTAAATACATGCATTCAGAGAATCTGTGTGCTTTTCCCAACAGCTATGTTGTGTAATGGTGAACATCATTATGCAAACACAGCT>Gorilla-ECR11ACTAGCTGTTAGGATGTTCAGCTTGGTCCAGTTGCCAAACAAAACCCCAGGGCAACTACTCACCACGGAATTTTCCCCCAATTAATAACCTACGAATGCCCGGCTTGAGTCCAAGCTCCATATGCACTGACACCCAACTCAGGAAGGGAACGGTTTTCCCAGAAACAGCACAACATAGCCAGTAGTTGGGAGACAACCATTTCTAAACCATAGAAAAGACTTCATACTAGCTATCTGTTTATAGATTGTGTTCCTGGGTCGTATCCATAACTGGATTTTCTCTGGTGTTCTGGGAGCAAAAATCTTCTGTCCTCTTTTTGAGGTGAGTAGAGTGTTCCAAATGTCCTTCATGGCCCCAGCCCACCGTTTTCTTTATGTGGGAAGGTTTTAGATGTTGGCACATATTGACACCTACTTGCAGACAGGTTTGTTGAATTACATTACATAAACGTTGAAATAATAACTCAAACAGTGAGTACATTTTCACTCTTATTCCCCTGCACCTAGCTTTATAAATCGCTAAATACATGCATTCAGAGAATCTGTGTGCTTTTCTCAACAGCTATGTTGTGTAATGGTGAACATCATTATGCAAACACA>Horse-ECR11GTCCAAGCTCTATATACATTAACATCCAACTCAGAAAGGAAATGGTTTTCCCAGAAACAGCACAACATGGCCAGTAATCTGGGGACATCCATTTCTAAACCATAAAAATGTTTAACATGAGACATCTGTTTCTAGATTATATCCCTGGGTTGTGTTTGCAACGGGGTTTTCTCGGGTAGAAACCTTGTGTCCTCCTTTTGAGATGAGTTAAGCGCTTGTTCCAAATGCCCATCATGGCCCACGGCCCACAGTTTTCCTTCCGTATCAAGGTTTTAAATGTTGGCACATAATGACAATCTACTTCCAGGCATGTTGGTTGAACAAAATTACATAAACATTGGAAAAACTCAAATGGTGAGTAAATTTTCACTCTTATTCCTTCACATCTAACTTTATAAATCTCTACATACATGCATTCAAATGAGTTGTCTGCTCTTCCCCAATAGCTAC>Megabat-ECR11GCTGTTAGGATGTCAGCTGGGTCCTGGTTGCCAAATAAAATGCAGGGCAGCCACTCACCATGGAATTTCCCCCTGATTAATGACCTATGAGTGCCCAGCTCAAGTCCAAGCTCTATATACACTGACATCCATCTCAGGAAGGGAACAGTTTTCCCAGAAACAGCACAACATGGCCAGTATTCTGGTGACGTTCATTTCTA>Minke-whale-ECR11AAGCAAACCATCTCTGAACACTAGCCGTTAGGATGTCAGCTTGGTCCCAGTTGCCAAATAAAACGCCAGGGCAGCCACCCACCATGGAATTTCCCCTGATTAATGACCTACAAATGCCAGGCTCAAGTCCAAGCTCCATATACATCGACATCCAACTCAGGACAGGAACCGTTTTCCCAGAAACCGCACAAATGCCCAGTATTCTGGAGACGTCCGTTTCTAAACCATAAAAATACTTAACATTAGCCATCTGTTTATAGATTGTATCCCTGGGTCATGTTCATAACTGGGTTTTCTCTGGCTTTCTGGGATGAAAAATCTCGTGTCCTTGTTTTGAGATGAGTTGAGTG>Mouse-lemur-ECR11TAGCTGTTAGGACGTCAGCTTGGTCCAGTTGCCAAGTAAACCCCCCAGGCAGCTGCTCGCATGGAATCCCCCTGCTTAATGACCTGTGAATGCCCGGCGTCAGTCCAAGCTCTGCATATGTTGGCATCCAACTCAGGAAGGGAATGGTCTTCCCAGAAACAGTACAACGTGACCAGTATTCTGGAGCTGTTCATTTCCAAACCATAAAAATATTTTATACTAGCTATCTGTTTATAGATTATATCCCTGGGTAATGTTCATAACTGGGTTTTTCTGGTGTTCTGGGGGGAAAATCTTGTGTCCTCATTTTGAAATGAGTGAGTGCTTGTTTCAGATGCCCTTCATGGCCCAGTTTTCTTTATGTAGGAAGGTTTTAAATGTTGGCACAAAATGACAGCCTACTTGCAGACATGTTTGTTGAGCTACACTGCATAAACATTGAAATAATAACTCAAACAGAGCACACATTTTCACTCGTGTTTCTCTGTGCCCAACTTTAT>Orangutan-ECR11ACTAGCTGTTAGGATGTTCAGCTTGGTCCAGTTGCCAAACAAAACCCCAGGGCAACTACCCACCACGGAATTTTCCGCTGATTAATAACCTACGAATGCCCGGCTTGAGTCCAAGCTCCATATGCATTGACACCCAATTCAGGAAGGGAACGGTTTCCTCCCAGAAACAGCACACATAGCCAGTAGTTGGGAGACAACCATCTTCTAAACCATAAAAAAGACTTCATACTAGCTATCTGTTTATAGATTGTGTTCCTGGGTCATGTCCATAACTGGGTTTTCTCTGGTGTTCTGGGGACAAAAATCTTCTGTCCTCTTTTTGAGATGAGTTGAGTGTTCCAAATGTCCTTCATGGCCCCAGCCCACCGTTTTCTTTATGTAGGAAGGTTTTAGATGTTGGCACATATTGACACCTACTTGCAGACAGATTTGTTGAATTACATTACACAAACATTGAAATAATAACTCAAACAGTGAGTGCATTTTCACTCTTATTCCCCTGCACCTAGCTTTATAAATCTCTAAATACTTGCATTCAGAGAATCTGTGTGCTTTTCCCAACAGCTATGTTGTGTAATGGTGAACATCATTATGCAAACA>Panda-ECR11CCCTGATTAATGATCTCCGAGTGCCAGGCTTGAGTCCAAGTTCTATAGACGTCGACATCCAACTCAGGAAGGGAACGGTTTTCCCAGAAACAGCACAACATGGCCAGTATTCTGGGGACATCCATTTCTAAACCATAAAAAGATAGAATACTAGCCATCTGTGTATAGATAATGTCCCTGGGTCATGTTCGCAGCTCTGGTGTTCTGGGGGGCAGGGGAATCTTGTGTCCCCGTTTTGCAATGAGTTGAG>Pika-ECR11CCCACCGTGGAATTTTCCCCTGATTAATGACCTTGGACCGCCAGGCTCACGTCCAAGCTCTGTATACGTTGACATCCAACTCAGGAAGGGAACAGTTTTCCCAGAAACTGTACAACATGGCCAGTATTCTTGAGACATCCAGTTATAAAC>Rabbit-ECR11TAGCTGTTAGGATGTCAGTTTGGTCCAGTTGCCAAATAAAACCTCAGGGCAACTACCCACCATAGAATTTTTCCCTGATTAATGACCTTCAAATGCCCGGCTCATATCCAAGCTCTATATACATTGATATCCAACTCAGGAAGGGAACAGTTTTCCCAGAAACAGTACAACATGGCCAGTATTCTTGAGACATCCAGTTA>Rat-ECR11AGCAAAGCGTCTCAGAACACTAGCTGTCAGGATGTCAGCTTCATCAAGTTGCCAAGTAAAACCCCACAGCAACTGCCCACCATGGAATTTTCCCTGATTAATGACCTACGAGTGCCCGGCTGGTGTCCAAGCTCTATATACGTTGACATCCAGCTCAGGAAGGGAAACTATTTTCCCAGAAACTGCACAACATGGCCACTATTCTTCAGACGTCCGCTTCTAAAACCATAAAAATATTTCATACTAGCCGTCTGTTTGTGGACCATATCCCTGGGTCGTGGACATAACTGGGTTTTCTCTGGTGTTCTGGAGGGGGTTGGACCGTCCTCATCTGGAGATGACTTGAACGCCTGTTCCCAGTGCCCTTGCAACCCATGCGCCACACTCTCCCTGTTCGGCGTTGGCACAGAGCAGTAACCTACTTGCAGACGTGTGGATTGAACTACATTATATAAACATTTAAATAACAAGTCAAACAAGCAAGTACATTTTCACTCTTGTGCCTCTGTACCCAACTTTATAAATCTCTAAATGTGTGCATTCAGAGGAGCTGTGTGCTCTCCCCCAATAGCTGCCTTGTGTAACAGTAAACACCATTATGCAAACG>Rhesus-ECR11ACTAGCTGTTAGGATGTTTGGCTTGGTCCAGTTGCCAAACAAAACCCCAGGGCAACTACCCACCGTGGAATTTTCCCCTGATTAATAACCTACGAATGCCCGGCTTGAGTCCAAGCTCCATATACATTGACATCCAACTCAGGAAGGGAACAGTTTTCCCAGAAACAGCACAACATAGCCAGTATTTGGGAGACAACCATTTCTAAACCATAAAAAGGACTTCCTCGTAGCTCTCTGTTTATAGATGGTGTTCCTGGGTCACGGCCATAACTGGGTTTTCTCTGGTGTTCTGGGGCAAAACTCTTCTGTCCTCTTTTTGAGATGAGTTGAGTGTTCCAACTGTCCTTCAT>Sheep-ECR11TTAGGATGTCAGCTCGGACCCAGCTGCCAAATAAAACGCCAGGGCAGACACCCACCGTGGAATTTCCCCCTGATTAATGACCTACGGAGGCCAGGCTCAGGTCCAAGCTCCGTATACATCGACACCCAGCTCAGGAAGGGAATGGTTTTCCCAGAAAAGGCACAAACGCCCAGTATTCTGGCCACGTCCACTTCTAAACCATAAAGAGGTTTAACATTAGCTACCTCTTTACAGATTAGATCCCTGAGCC>Sloth-ECR11GCTTCCCTTGGAGGTGATTATTTCTGAGAAACAGCAAATGTTCTCTGCAAACTAGCTGTTAGAATGTCAGCTTGGTCCAGTTGCCAAATAAAACCCCAGGGCAACCACCCACCATGGAATTTTCCCCTGATGAATAAACTAAGAATGTCCGGCTTGAGTCTAAGCTCTCTATACATTGACATCCAACTCAGGAAGGGAATGTTTTTCCCAGAAACAACACAACGTGGCCAGTATTCTGGCAATATTCATC>Squirrel-ECR11AGGATGTCGGTTTGGTCCAGTTGCCAAACAAAACCCCGAGGCAGCTGCCCACGGTGAATTTCTCCTGATTGATGACCTACCCATGCCCGGCTGGCGTCCAGGCTCGGTGTACACCGCCGTGAACTGGAGAGGAGAGCCTCTTTCCCAGAAACAGCTCAGCGTGGCCAGCATTCTGCAAGTGTCCGCTTCTAAGCCATAAAAATGTCCCACACTGGCCCCCGGTTTATGGCTCACGATCTGGGCTCTGTCCGTAGCTGGAGGTTCTCTGGTGTTCTGGGGTGCAAGTCTGTGTCCTGGCTT>Squirrel-monkey-ECR11CCCTGATTAATGACCTATGAATGCCCGGCTTGAGTCCGAGCTCTGTATACATTGACATCCAACTCAGGAAGGGAAACGGCTTTCCCAGAAACAGCACAACACAGCCAGTATTCATGAGATGACCGTTTCTAAACCATAAAGAAGACTTCATACTAGCTATCTGTTTATAGATCAGGTTCCTGGGTCATGTCCATAACTGGGTTTTCTCTAGTGTTCTGGGGCAAAAATCTCCCACCCTCTTTTTGAGCTGAGTCGAGTGTTCCAAAAGTCCTTCATGGCTGCAGCCCACCGTTTTCTTTATGTAAGAAGGTTTTAGATGTTGGCACACATTGACACCTACTTTCAGACAAGTTTGTTGAATTACATTACATAAAAGTTGCAATAATAACTCAAACAGTGAGTACATTTTCACTCTTTTCCCCTGTACCTGGCTTTATAAAATCTCTAAATATGTGCATTCAGAGAAGCTGAGTGCTTTTCCCAACAGCCATGTTGTATAA>Tarsier-ECR11GTTGCCAAGGAAAACCCGGGGCAGCCACCCACAGAGGAATTTTCCCCTGATTAATGCCCTCCAAATGCCCGGCTCGAGTCCAAGCTCCATATACATTGCCATCCAACTCAGGAAGGGAATGGTTTTTCCAGAAACAGCACAACACAGCCAGTATTCAGGAGACAACCATTTCTCAGCCATAAAAACTATTCCATACTGGCCATCTGTTTATAGATTATATCTCTGGGCCATGTCCATAACTGGGCTTTCTCGGGTGTTCTGGAGTGAAAAAAACTTGTGTCCTCTTTTTGAGATGAGTTGAGTGCTTGTTCCAAATGTCCTTCATGGCCCCCGGTGCACAGTTTTCTCTGTGTAGGAAGGTTTTAAATGTTGGCACCAACTGGCCACCTACTTGCAGACGTGTTTGTTGAACCATACCACATAAACATTGAAATAATAAACAGTGAGCACGTTTTCATGCTCATTCCTCAGCCTCTAGCTGTATAAGGTCTCTAAAGACACATTCAGAGGAGCTGCATGCCTTTTCCAACAGCTGCCTTGTGTAACCTCA>Tenrec-ECR11AGGTCAACATCGGGCCCAGTTGCCAGGTAAAACCTTGGTTCAACAATCCGCCATGGAATTTTCCCCCTGATTAATGAGCTCAACGCTGGATTGGGATCCAAGATATATACACGTTTCAATCCAACTCAGGAAGGGGGATGGCTTTCTCGG>White-rhinoceros-ECR11GTCCAAGCTCTATATACATTGACATTCAACTCAGGAAGGAATGGTTTTCCCAGAAACAGCACAACATGGCCAGCAATCTGGGGACGCACATTTCTAAACCATAAATATGTTTAACATGAGACATCTGTTTATAGAGTATATCCTTGGGTCGTGTTCATAACTGGGTTTTCTCTGGTGGAAATCTGTGTCCTTCTTTTGAGATGAGTTGAGCGCTTGTTCCAAATGCCCATCATGGCCCTTGGTCCACAGTTTTCCTTCCGGATCAAGGCTTTAAATGTTGGCGCATAATGACAGTCTACTAGGCATGTTGGTTGAACGAAATTACGTAAATATCGGAATAATAACTCAAACGGTGAGTACATTTTCACTCTTATTCCTCTGCATCTAACTTTATAAGTCC>Mouse-ECR12CCGGTGTATTCGTGTTCACAAGAGAGACTGCTGAGCCCATCCCCCAGCCAGGACACTTGGCCCTGAGTTGCTCAAGAGCCCTCATCCTCAGTCATTGCAGGGACTTGTTGCCATGGAAACTGCAGCCAGTGTTTCCATGGAGAAAGAGCTGCAGAGTCTGGAATTTTCCAGGTTCTAAAGGTGAATGTATATTCTCTTGAAAACAACTCTGAATGCTTTCCCCCAGG>Human-ECR12TCTGCTGGTTCCTTCCCCCAGCCCATCCCATCCTCCAAACAGACTATTTTGTTGTTAGCTGCTCTGCTCTGTTCATCCCTCAAGAGCTCTCACCCTCTGCCATTGCAGGGGCTGTCTCCATGGAAACTGTAGCCAGCGTTTCCATGGAGAAAGAGCCCGCAGAGTCTGGAATTTTCCAGGCTCTAAAGGTGACTGCAAAAACATCTCAGCACATCTCCAGAACTGGGCTGCCCAACATGGCGGCCACTGGCCACAGGTGGCGACTGACCACTTGAAATGTGGCCGGTCCCCG>Baboon-ECR12CCAGCCCATCCCATCCTCCAGACAGAGTACTTTGTTGTTAGTTACTCTGCTCTGTTCATCCCTCAAGAGCTCTTGCCCTCTGCCATTGAAGAGGCTGTCTCCATGGAAACTGTAGCCAGCGTTTCCATGGAGAAAGGGCCGCAGAATCTGGAATTTTCCAGGCTCTAAAGGTGTTTGCAAAAACATCTCAGCACGCTCCAGACCTGGGCTGCCCAACATGGCAGCCACTGGCCACGGGTGGAGACTGACCATTTGAAATGTGGCCAGTCCCCA>Bonobo-ECR12TGAGGTGTTGATGGGGTGGCTGTCTGCTGGTTCCTTCCCCCAGCCCATCCCATCCTCCAAACAGACTATTTTGTTGTTAGCTGCTCTGCTCTGTTCATCCCTCAAGAGCTCTCCCTCTGCCATTGCAGGGGCTGTCTCCATGGAAACTGTAGCCAGCGTTTCCATGGAGAAAGAGCCCGCAGAGTCTGGAATTTTCCAGGCTCTAAAGGTGACTGCAAAAACATCTCAGCACATCTCCAGAACTGGGCTGCCCAACATGGCGGCCACTGGCCACAGGTGGCGACTGACCACTTGAAATGTGGCCGGTCCCCG>Bushbaby-ECR12CTTTTTGGTGGGCTGCCTCCAGCCTCCAGCCCCCAACCCCTGCCAAGAGGGTCATGGTTAGCCTCTCTGCTCTGTGTGTCCCTTGAGAGCTCTTGCCCCATCATTGCAGGGGCTGTCATCATGGAAACCGTCACTAGAGTTTCCATGGAGAAAGAGCCTCCACAGAGTCTGGAATTTTCCAGGCTCTAAAGGTGAACACAAGCAGGTTCAAAAACACTCCAGACCTGTGCAGGTCAACAGGGTAGCCACTGGCCACACGTGGTGGCTGAGCTACTAAAATGTGGCTGGTCTGAATC>Cat-ECR12TGGAAGAGGCATCGGTGGGGGGCTGTGTGCTGGGCCTTTCCCCAGGCCGCCCCATCCCCCAGCCAGGCTGTTTTGTTTTTTGGTTGCTTTTCTCCGCTCATCCAGGAGCATTGACGCCTCACTGCAGAGCCTAGCTCAGGCTGTTGCCATGGAAACCGTAGCCCTACGTGGGCAGGGGAGCCAGGCTGTGGAGATGGAGAAAGAGCCACAGCATCTGGAATTTTCTGTGCTCTAAAGGTGAATGCGTGTGGCTTCAAAACCATCTCAGGACCCTCTAGACCTGGGCTGCCCCACACACCT>Chimp-ECR12TGCTGGTTCCTTCCCCCAGCCCATCCCATCCTCCAAACAGACTATTTTGTTGTTAGCTGCTCTGCTCTGTTCATCCCTCAAGAGCTCTCACCCTCTGCCGTTGCAGGGGCTGTCTCCATGGAAACTGTAGCCAGCGTTTCCATGGAGAAAGAGCCCGCAGAGTCTGGAATTTTCCAGGCTCTAAAGGTGACTGCAAAAACATCTCAGCACATCTCCAGAACTGGGCTGCCCAACATGGCGGCCACTGGCCACAGGTGGCGACTGACCACTTGAAATGTGGCCGGTCCCCG>Chinese-hamster-ECR12CTGAGCCCATCCCCCAGCCAGGACTCTCTCGGTCCGGAGTTGCTCAAGAGCCCTCATCCGCAGTCATTGCAGGGGCCTGTTGCCATGGAAACTGTAGCCAGCGTTTCCATGGAGAAAGAGCTGCAGAGCCTGGAATTTTCCAGGTTCTAAAGGTGAATGTACATTAGCTTGAAAAACAACTCTGTACATTTCGCACCCAG>Cow-ECR12GGGAGCTGGGTTGTCCCATCTCCCCACACACCCCCACCTCCTCGCGCTGCCCCAGCCCTTGAGGAGTAATTACCTGGTGGAGAAGGAGGCAGGCTCCAGCTGTTGCCATGGAAACTGCAGCCAGGAGCAGGGGAGGGAGGGGGTCTGCACCAGTGAGGCTGGAATTTTCCAGGCTCTGACGTCAGCTGTGTGTGGTGATGGGGATGGTGGGGAGGTTCAGGATGCCAGGGAGCATACTTCAGGTGCATAA>Elephant-ECR12CCCATCCCCCAGCTGGGCTAGGTTGATATTCATGGTTCTGGCTTGTTCATTTTCAGGAGCTCTTAGCTCTTGTTAGCTATACAAGCAGGTTCAGTCTGTTGCCATGGAAACTGCAGCTGGAGTGGGAAAGAGGAGGGAGACTGAGGAATCAGCAGAGACTGGAATTTTCCAGCTCCAGAAGCTGACTTGTGTGTAAACTC>Ferret-ECR12CCCATCCCCCAGCCAGGCTGTTTTGTTTTCCTTGCTTTGCTCTGATCCTCCCAGAGCGTTGATGCCTCACTTCCGGGCGGGCTCAGGCTGTTGCCATGGAAACCATCGCCAGCGAGGGCAGGGGAGCCAGGCTGTATGGATGCAGAAAGAGTCATGGAGTCTGGAATTTTCCAAGTTCCAAAGGTGAATGTGTGTGGCTT>Gibbon-ECR12TGCTGTGCATGAGGTGTTGACAGGGTGGCTGTCTACTGGTTCCTTCCCCCAGCCCATCCCATCCTCCGAACAGACTATTTTGTTGTTAGTTGCTCTGCTCTATTCATCCCTCAAGAGCTCTCACCCTCTGCCATTGCAGGAGCTGTCTCCATGGAAACTGTAGCCAGCGTTTCCATGGAGAAAGAGCCGCAGACTCTGGAATTTTCCAGGCTCTAAAGGTGACTGCAAAAACATCTCAGCACATCTCCAG>Marmoset-ECR12TCTACTGGGTCCTTTCCCCAGCCCGTCCCATCCTCCAAACACACTATTTTGTTGTTAGTTGCTCTGCTCTGTTCATCTCTCAAGAGCTCTCACCCTTTGCCATTGCAGGGGCTGTCTCCATGGAAACTATAGCCAGCGTTTCCATAGAGAAAGAGCCGCAGAGTCTGGAATTTTCCAGCCTCTAAAGGTGATTGCAAAAACATCTCAGCACACTCTAGACCTGGATTGCCCAACATGGCAGCCACTGGCCACAAGTGGTGACCAAGCGTTGAAATGTGGCTGGTCCCCGTA>Megabat-ECR12CCCATCCCCCAACCAGGTTAGGTTGATGTCAATGGTTCTGGCTTTTCTTCAAGTGCTCTTTGGCTCTTGTTAGCTATAGAATGCAGGTTCAGTCTGTTACCATGGAAACTGCAGCTGGAATGGGGAAGAGGAGGGAAGCTGAGGAATGGGCAGAGACTGGAATTTTCCAGCTCCGGAGGCAGACTGTGTGTGAAATCAAA>Mouse-lemur-ECR12TGTGAGGCCTGGTTGGGGTGACTGTCTGGAGGCCCCTTCCCCTAGCACTCCATCCCCCAACCAGGCTATTTTGTTTTTAGTTTCCCTGCTCTGTTCATCCCTCAAGAGCTCTCAACCATCATTGCAGGGAGTGTCACCATGGAAACCATAGCTAGCGTTTCCATGGAGAAAGAGCCTTTGTGGGGTCTCGAATTTTCCAGGCTCTTAAGGTGAATGCATACAAGTTCAAAAACATCTCAGCACCCTCTGCACCTGTGCCAGCCAACATGGTAGCC>Orangutan-ECR12ACTGGTTCCTAATCCCCCAGCCCATCCCATCCTCCAAACAGACTATTTTGTTGTTAGTTGCTCTGCTCTGGTTCATCCCTCAAGAGCTCTCACCCTCTGCCATTGCAGGAGCTGTCTCCATGGAAACGGTAGCCAGCATTTCCATGGAGAAAGAGCCCGCAGAGTCTGGAATTTTCCAGGTTCTGAAGGTGACTGCAAAAACATCTCAGCACATCTCCAGAACTGGGCTGTCCAACATGGCGGCCACTGG>Panda-ECR12CCCATCCCCCAGCCAGGCTGTTTTGTTTTACTTGCTTTGCTCCGTTCATCCTGGAGCATTGATGCCTCCCTGCAGGCCTGCTCTGGCTGTTGCCATGGAAACCGTAGCCAGTGTGGGCAGAGGAGCCTAGCTGTGGGGATGGAGAAAGAGTCGCAGAGTCTGGAATTTTCCATGCTCTAAAGGTGAATGAATGCATGTGG>Pig-ECR12CTGTTCCTCCCTCAAGAGCTTCCTTGCTGTTGCTGCAGGGTTGGCTCCAGCTGTTGCCATGGCAACTGTAGCGCGTGGGCAGAGGAGGAGGGCTGTAGCAATGGAGAAAGAGCTGCAGGGTCTGGAATTTTCCAGCCTCTGAAGGTGAACACATGTGGCTTCAAAAACATGTCAACTACACTCTAGACCTGGGGCGTCCAACGTGGTAGCCACGGGCCGTCACGTGGCTCCTGAGCTCTTAAAATGTGT>Rabbit-ECR12CTACCCAACCCCCAGCCAGGCTATTTCGTTATTAGTTGCTCAGCTCTGTTCATCTCTCAAGAGCTCTCACCCTCTCTTAGCAGGGGCTGTCGCCAAGGAGACTGTAGCCAGTTGTTTCCATGGAGAAAGAGATGCAGAGTCTGAAACTTTCCAGACTCTCAAGGTGAATACTTATGGGTTCAAAAATATCTCAGTGCACTCTGGCCTGAGCTGTCCAGCACAGTAGCCACTGGCCACATGTGGGTACTGAGCACTTGAAACGTGGTCGG>Rat-ECR12TTCACAAGAGAGACTGCCGAGCCCATCCCCCAGCCAGGACACTTGGCCCTGAGTTGCTCAAGAGCCCTCATCTTCAGTCATTGCAGGGACTTGTTGCCGTGGAAACTGCAGCCAGCGTTTCCATGGAGAAAGAGCTGCAGAGTCTGGAATTTTCCAGGTTCTAAAGGTGAATGTATATTCGCTTGAAAACAACTCTGCAT>Rhesus-ECR12TCTGTTCATCCCTCAAGAGCTCTTGCCCTCTGCCATTGCAGAGGCTGTCTCCATGGAAACTGTAGCCAGCGTTTCCATGGAGAAAGGGCCGCAGAATCTGGAATTTTCCAGGCTCTAAAGGTGTTTGCAAAAACATCTCTGCACACTCCAGACCTGGGCTGCCCAACATGGCAGCCACTGGCCACGGGTG>Squirrel-ECR12TCTCTTCTGCCACCCCCTGTCTGACGCCCGGGTCTCTGCAGTGCTGACCCGCGGGAGCCCAGCCCCCACCGCCCACGCCCGGCTCTCTGCCTGCTCATCTCTCAAGAGCCCTCACCCCTGTCACTGCAGAGCTGTTGCCATGGAGACCGCAGCCAGTGTTTCCATGGAGAAGGGCTTCAGAATCTGGAATCTTCCCAGCTCTAAAGGTGAACGCAGGCGGGTTCAGAAACATCTCGGCGCCCTCCACACCTGGGCCGGCCCTGGCCAGCGTGGCCGCCGCCGGCCTGAGAGGCCAGCCAGCCCTGGAGCTG>Squirrel-monkey-ECR12TACTGCGTCCTTTCCCCAGCCCGTCCCATCCTCCAAACAGACTATTTTGTATTGTCAGTTGCTCTGCTCTGTTCATCTCTCAAGAGCTCTCACCCTTTGCCATTGCAGGCGCTGTCTCCATGGAAACTATAGCCAGCGTTTCCATAGAGAAAGAGCCGCAGAGTCTGGAATTTTCCAGGCTCTAAAGGTGATTGCAAAAACATCTCAGCATACTGTAGACCTGGATTGCCCAACATGGCGGCCACTGGCC>Tree-shrew-ECR12CATTCCCCAACCAGGCTATTTTGCTGGTACTTGCTGTGCTCTCTTCATCCCTCAAGAGCTCTCACCCTCATCATTGCAGGGTCTGTCACCATGGAAACAGTAGCCAGTGTTTCTATGGAGAAATTGCCGCAGAGTCTGGAATTTTCCAGCCTCTAAAGGTGAATGCATATGGATTCATTAGAAAATCACCTCAGCATGCTCTAGCCCAAGGCTGTCCAAC>Mouse-ECR13CACCTCTACCCACTCACCCCCAGCCCCAAAAGAGAAAACGAATGGAAAAGCTAAGAAAGACAGACTTGGAAGGATATAGCTGGTAGTCCTGACTGCTTTTAGGGTTTGGAAATGCTTGTCTTCCCAACAGAGGCCAATTGAGATACGTCCAAGAAACCCAACCGCTTCCTGCAGGGGAGGAGATGTATGTATTGCTTGGTTTGTTGCCGAGAGCCAAGCTAAATGAACCACGGTGAGTCCATGAACTAAGTACAATCTGTCGACGGGGGCCAGGAAACAAAGTTCAAAGACTCCTTTTTGAAGAGCACAAGTTTCCAATTAGAAACTTGCGCATTCCTGATCAGCCCAGTCACAATTCAGACCTGGCCTAACCCCTTCC>Human-ECR13CACCCCGGAAAACTAGGAGAAAAGCTAACGAAGGTGAGGGAAGGATGTCGTCTGGAAAGGAGATGCCTGGTATTTGATATTTTCAGGGTTCGGAAACGCATGTCTTCAGCACAGAGGCCAATATTAAGTTGTTTCCAAGAAATCCAACCGCTCCCTGCTAGAGAGGAGATGTACGTAGGGTTTGGTTTCCAGCCGAGCACTGAGCTAATATCAACCACGGTGAGTCTTTGAACTAAAATATAATCTGATGACCAGGCCAGGAGGCAATATCCATTCGGCTCCTTTCCAAGAACCACACGTTTCCAATTAGACATGTTCTCCCGAGGGACCCCTAGGTGCAATTCAGATCTGGTGCCAATCCCTCCTGCAGCAATGGGATCTGGAGGCCTTCATGAGGACTGGAGTTTCTGG>Bonobo-ECR13CACCCTGGAAAACTAGGAGAAAAGCTAACGAAGGTGAGGGAAAGATGTCGTCTGGAAAGGAGATGCCTGGTGTTTGATATTTTCAGGGTTCGGAAACGCATGTCTTCAGCACAGAGGCCAATATTAAGTAGTTTCCAAGAAATCCAGCCGCTTCCTGCTAGAGAGGAGATGCACGAAGGGTTTGGTTTCCAGCCGAGCACTGAGCTAATATCAACCACGGTGAGTCTTTGAACTAAAATATAATCTGATGACTGGGCCAGGAGGCAATATCCAATCGGCTCCTTTCCAAGAACCACACGTTTCCAATTGGACACGTTCTCCCGAGGGACCCCTAGG>Cat-ECR13ACACAGAAAGATATAGAGTCTGGGAAAGGATAGCTAGTGTTAAATACTTTCATGATTTGGAAACTCATGTCTTCAGAACAGAGGTCAATGTTGAGTTGTTTCCAAGAAATCTAACCGCTTCCTGCTAGGGAGGAAATGTATGTATTGTTTGGTTTCCAGCCGAGCACTGAGCTAATGAACCACGGTGAGTCCTTGAACTAAAATATAATCTGGTGATTGGGCCAGGAAGCAACATCCAAACTACTTCTTTTCCAGGACCACAAGTTTCCAATTAGACATGTGTCTCTTAACTGAATTCAGATCTGGTCCAAAAGCCTCCTGTAACACTGAGGTCTGGGGCCTTAATGAGGACCAGAGTTTCTGGGCCCAGGGAGGA>Chimp-ECR13CACCCTGGAAAACTAGGAGAAAAGCTAACGAAGGTGAGGGAAAGATGTCGTCTGGAAAGGAGATGCCTGGTGTTTGATATTTTCAGGGTTCGGAAACGCATGTCTTCAGCACAGAGGCCAATATTAAGTAGTTTCCAAGAAATCCAGCCGCTTCCTGCTAGAGAGGAGATGTACATAGGGTTTGGTTTCCAGCCGAGCACTGAGCTAATATCAACCACGGTGAGTCTTTGAACTAAAATATAATCTGATGACTAGGCCAGGAGGCAATATCCAATCGGCTCCTTTCCAAGAACCACACGTTTCCAATTAGACATGTTCTCCCGAGGGACCCCTAGGTGCAATTCAGATCTGGTGCCAATCCCTCCTGTAGCAATGGGATCTGGAGGCCTTCATGAGGACTGGAGTTTCTGG>Chinese-hamster-ECR13ACCCACTCACCAATCGCCCCCCCCCCAAAAAAAAGAAAACGAATTGAAAAGCTAAAGAAGAGAGACATGGAAGGATATATCTGGTAGCCCTGACTGCTTTTATGGTTTGGAAATGCTTGTCTTCCCAACAGAGGCCAATTGAGATACTTCCAAGAAACCCAACCGCTTCCTGCTGGGGAAGAGATGTATGTATTGCTTGGTTTATTGCCGAGCGCCAAGCTAAATGAACCACGGTGAGTCCATGAACTAAGTACAGTCTGATGACGGGGGCCAGGAAACAACTTCCAAAGACTCCGGTTTTTTTTTGAAGAGCACAAGTTTCCAATTAGGAACTTGCAAGCTCCTGATCAGCGGGGTCACAATTCAGACCTGGCCCAACCCCTTCCCTAAATGGAGTCAG>Dolphin-ECR13AGGGATTTGGGCCAGATCTGAATTCAGTTGGGAGACACATGTCTAATTGGTAACTCATAGTTGCCAATAAACTCTGGAAAAGAAGGAGTTTGGATGTTGCTTCCTGGCCCAATCCTCATTGTATTTTAGTTCAAAGACTCACCGTGGTTCATTAGCTCAGTGCTCGGCTAGAAACCAAAAAATACATACATTGCGTCCTTAGCAGGAAGTGGTTGGATTTCTTGGAAACAACTCAACGCTGACCTCTGTTCTAAAGA>Dog-ECR13CATGGAAGGGTAGAGTCTAGGAAAAGGAAAGCTAGTGTTAAGTATTTTCATGATTTGGAAACCTATGACTTTGGAACAGAGGTCAATGTGGAGTTGTTTTCAAGAAATCCAACCGCTTCCTGCTAGGGAGGAAATGTATGTATTGTTTGGTTTCTAGCCGAGCACTGAGCTAATGAACCACGGTGAGTCCTTGAACTAAAATATAATCTGATGATTGTGCCAGGAAGCAACATCCAAACTCCTTCTTTTCCAGGACCACAAATTTCCAATTAGATGTGTGTCTCTTAACTGAATTCAGATCTGGCCCAGATCCCTTCTATAACACTAAGGTCTCAGGGCTTAATGAGGACCAGAGTTTCTGAACACAGTGAAGA>Ferret-ECR13CATGATTTGGAAACCCATGTCTTTGGAACAGAGGTCAACGTTGAGTTGTTTCCAAGAAATCCAACCGCTTCCTGCTAGGGAGGAAATGTATGTATTGTTTGGTTTCCAGCCGAGCACTGAGCTAATGAACCACGGTGAGTCCTTGAACTAAAATATAATCTGATGATTGGGCCAGGAAGCAACATCCAAACTACTTCTTTTCCAGGACCACAAGTTTCCAATTAGACATGTGTCTCTTAACTGATTTCAGATCTGGCCCTGATCCCTCCTGTAACACTAAGGTCTGGGGCCTTAATGAGGACCAGGGTTTCTGGGCACAGGGAGGA>Gibbon-ECR13CACCCCGGAAAACTAGGAGAAAAGCTAAGGAAGGTGAGGGAAGGATGTCGTCTGGAAAGGAGATGCCTGGTGTTAGATATTTTCAGGGTTCGGAAACGCATGTCTTCAGCACAGAGGCCAATATTAAGTTGTTTCCAAGAAATCCAACCGCTTCCTGCTAGAGAGGAGATGTATGTATGGTTTGGTTTCCAGCCGAGCACTGAGCTAATATGAACCATGGTGAGTCTTTGAACTAAAATATAATCTGATGACTGGGCCAGGAGGCAATATCCAATCGGCTCCTTTCCAAGAACCACACGTTTCCAATTAGACATGTGCTCCCGAGGGACCCCTAGGTGCAATTCAGATCTGGTGCCAATCCCTCCCGTAGCAATGGGATCCGGAGGCCTTCATAAGAACTAGAGTTTCTGG>Marmoset-ECR13CCCGGAAAACTGGGAGAAAAGCTAAGGAAGGCAACAGGGAAGGATGTCGCCTGGGAAGGAGATGGCTGGCGTTCGATGTTTTCCTGGTTCGGAAACACACGTCTTCAGAACAGAGGCCAATATTAATTTGTTTCCAAGAAATCCAACCACTTCCTGCTAGAGAGGAGATGTATGTATGGTTTGGTTTCCAGCAGAGCACTGAGCTAATGAAGCACGGTGAGCCTTTGAACTACAGCATGATCTGATGCCTGGGCCAGGAAGCAATATCCAATCGGTCCTTTTCAAGAACCACAAGGTTCCAATTAGACATGTGCTCCCAAGGGATAGGCACAATTCAGATCTGGCCCCAGTCCCTGTAGCGATGGGATCTGGAGGTCTTCATAAGGACTAGAGTTTCTGGGTGCCGGGATAGGCACGGTGTCCACAGCAGGAAGAGAGTTCCTTTC>Panda-ECR13ATGGCTATTGTGTATAAAAATCTGCAATGTGCACACACAGGCACCAGTCTGGGGATAGGCGTTAGGTCCGTCAGAAAATGATGAGGCTTGGGTGACAGAGAAGCTAAGAAAGAAGAACCTGGAAGGAGAGAGCCTGGGAAACGGATAGCTAGTGTTAAATATTTTCATGATTTGGAAACCCGTGTCTTCGGAACAGAGGTCAATGTTGAGTTGTTTCCAAGAGCTCCAAACGCTTCCTGCTAGGGAGGAAATGTATGTACTGTTTGGTTTCCAGCCGAGCACTGAGCTAATGAACCACGGTGAGTCCTTGAACTAAAACATAATCTGATGACTGGGCCAGGAAGCAACGTCCAAACTACTTCTTTTCCAGGACCACAAGTTTCCAATTAGACATGTGTCTCTTAACTGATTTCAGATCTGGCCCCAATCCCTCCTGTAACACTGAGGTCTGGGGCCTTAATGAGGACCAGAGTTTCTGGGCATGGGGAGGA>Rat-ECR13CACCCCTACCCACTCACCCCTAGCCCCCAAAGCGAAAACAAATTGAAAAGCTAAGGAAGACAGACTTGGAAGGATATAGCTAGTAGTCCTGACTGCTTTTATGGTTTGGAAATACTTGTCTTCCCAACAGAGGCCAATTGAGATACTTCCAAGAAACCCAACCGCTTCCTGCAGGGGGAGAGATGTATGTATTGCTTGGTTTATTGCCGAGCGCCAAGCTAAATGAACCACGGTGAGTCCATGAACTAAGTACGATCTGTTGATGGGGGCCAGGAAACAAAGTTCAAAGACTCCTTTTTAAAGAGCACAAGTTTCCAATTAGAAACTTGCTCATTCCTGATCAGCCCAGTCACAATTCAGACCTGGCCCAACCCCTTCCT>Squirrel-ECR13TCTGGGCTGGGCCAGCTCGGCACCCCGTCCCTCGGAGAGCCAGGGGGGACGGAGGGGAGGACTGAGCCCGGGAAGGGGAGGGCTGGCGTGGAGGGCCCCCGGGTTTGGAAATGCCTGTCTTCAGAACAGAGGCCAACACTGAGTAATTTCCAAGAAATCCAACCGCTTCCTGCAAGGGAGGCGATGTGTGCCGTGCTTGGTCTGCAGCCCAGCCCTGAGCTCAGGAGCCACGGAGCGTCCTTGAACTAGAATGCGCTGCCGTGGCCGGGCCAGGAAGCAACCTCCAAAGGCTCCTCTTGGAGCAGCACAAGTTTCCAATTAGACACGAGCCTCCCCACTGGACCCGGTCACAGTGCTGGACCGGGCCAGCTCCCTCCTGCAGCAGCAGGACTGGGGCCCCGGGAGGAGCAGGCAGGGGAGGCG>Squirrel-monkey-ECR13AGGCTAAGGCTCCACTCCAGAAAACTGGGAGAAAAGCTAAGGAAGGCAACAGGGAAGGATGTCGCCTGGAAAGGAGACAGCTAGTGTTCGATGTTTTCCTGGTTTGGAAACACAAGTCTTCAGAACAGAGGCCAATACTAATTTGTTTCCAAGAAATCCAACCGCTTCCTGCTAGAGAGGAGATGTATATATGGTTTGGTTTCCAGCCGAGCCCTGAGCTAATGAACCACGGTGAGCCTTTGAACTACAACATAATCTGGTAACCGGGCCAGGAAGCAATATCCAATCGGTCGTTTTCAAGAACCACAAGTTTCCAATTAGACATGTGCTCCCAAGGGAGCACAATTCAGATCTGGCCCCAGTCTCTCCTGTAGCAGTGGGATCTGGAGGCCTTCCTAAGGACTAGAGTTTCTGGGTGCAGG>White-rhinoceros-ECR13TGTTACAGGAAGGATTTGGGCCAGATCTGAATTCAGTCGGGGGGCCCATGTCTAATTGGAAACTTGTGGTCCTGAAAAAGAAGTAGTTTGGATGTCACTTCCTGGCCCAATCATCAGATTATATTTTAGTTCAAAGACTCACCGTGGGTCATTAGCTCAGTGCTCGGCTGGAAACCAAATGGCACATGCGTTGCCTCCCTAGCAGGAAGCGGTTGGATTTCTTGGAAACAGCTCAACGTTGGCCTCTGTTCTGAAGACATGGGTTTCCAAATCATGAATATATTTAACACTAGCCATCTCCTTTCCAGACTATATTCTTCCATGTTCTTTCTTAGCTTTTCTCTTAGTTT>Mouse-ECR14CATCTGGTAGTGTGGGGAAGACTTGAGCCTAACCTAAATAAACAGAAAAGGAATTTTGGTGGTCGAAACCTCTCTGACAGGAGACTCTCAGTCTTTCAGAAATAGTTTCAATGTCAACATCTGGTCCAGGTGCCAGATAAAACTTTGCTTCAACAACCCACCTCAGAATTTTTTCCCTGATTAATGAGCTCAGTCGTGGAGTGAAATCCAAGCAGTATATACATACCACTGCCCAGCTCAGGAGGAGACTGGCTTTCTCGGAAAGAGTACAACCCAGCCAGTGTTCTGGGTGC>Human-ECR14AATTTGGGCTCAAATAAATAGAAGTTCAGATGGTAGAAATCTTTCTAACTGGAGACTCTTGGTCTTTGAGAAGTAGTTTGAATGTCAACATCTCACCCAGTTGCCAGATAAAACCTCGGTTCAACAACCCACCATGGAATTTTTCCCTGATTAATGAGCTAAATTCTGTACTGGAATCCAAGCAATATATATGTTGCCATCCAACTCAGGAAGAGACTGGCTTTCTCGGAAAGAGCACAACCCACCCAGTGTTCTGGAGACCTCTATTTCCAAACCATAAAAATATTAAATACTAGCCAT>Alpaca-ECR14TCTAGGGCCCCTCAAGACAGCCTCTGTTGCCGCAGGAAGGATCTGGGCCCAACCTAAATAAACAGAAAAGAGGTCTAGATGGCAGGCACCTTTCTGACTGAAGACTCTTAGTCCACGGGAAGTAGTTTGAATGTCAACCTCTGGCCCAGGTGCCAGATAAAACCTCAGTTCAACAACCCACCATGGAATTTTCCCCTGATTAATGAGCTAAAGGCTGGACTGGAATCCAAGCAATATATATGTTGCCATCCAACTCAGGAAGGGACTGGCCTTCTCGGAAACAGCACAACCAAGCCAGTGTTCTGAAGC>Baboon-ECR14TAATCTCTCAGATTAAAGTCCTTCAAGAAGACCTTGGTTACTGCAGGAAGAATTTGGGCTCAAATAAAGAGAAGTTCAGATGGTAGAAATCTTTCTAACTGGAGACTCTTGGTCTTTGAGAAATAGTTTGAATGTCAACATCTCGCCCAGTTGCCAGATAAAACCTCGGTTCAACAACCCACCATGGAATTTTTCCCTGATTAACGAGCTAAATTCTGTACTGGAATCCAAGCAATATATATGTTGCCATCCAACTCAGGAAGACACTGGCTTTCTCGGAAAGAGCACAACCCACCCAGTGTTCTGGAGACCTCTGTTTC>Bonobo-ECR14TAATCTATCAGACTAAGGTCCTGCAAGAAGACCTTGGTTACTGCAGGAAGAATTTGGGCTCAAATAAATAGAAGTTCAAATGGTAGAAATCTTTCTAACTGGAGACTCTTGGTCTTTGAGAAGTAGTTTGAATGTCAACATCTCACCCAGTTGCCAGATAAAACCTCGGTTCAACAACCCACCATGGAATTTTTCCCTGATTAATGAGCTAAATTCTGTACTGGAATCCAAGCAATATATATGTTGCCATCCAACTCAGGAAGAGACTGGCTTTCTCGGAAAGAGCACAACCCACCCAGTGTTCTGGAGA>Bushbaby-ECR14CTCTAGGGTCCTTCAGGGAGCCTTCAGTTGCTGAAGGAAGGATTTGGGCTTAACCTAAATAAACAGAAGTTTAGACGGTAGAAACCTTTCTGACTGGAGACTCTAAGTCTTTGAGAAGTAGTTTGAATGTCAACAACTAGTCTAGTTGCCAGATAAAACTTTGGTTCAACAACCCACCATGGAACTTTTCCCTGATTAATGAGCTAAATTCTGTGCTGGGGTCCAAGCAATATATATGTTGCGATCCAAGTCAGGAAGAGACAGGCTTTCTCGGAAGGAGCACAACCCAGCCAGTGTTCTGGAGACCTCT>Cat-ECR14TAACCTACAGTGTTGTTGTAGGGTCTTTCCAGACAGTCTCAGTTGCTACAGGAAGGACTTGGGCCCAACCTAAATAAATAGAAAAGAGGTTTAGATGATAGAAACCTTTCTGACTGGAGACTCTTAGTCCTTGAGAAGTAGTTTGAATGTCAACATTGGGCCCAGTTGCCAGATAAAACCTCAGTTCAACAACCCACCATGGAATTTTTCCCTGATTAATGAGCTAAATGCTGGACTGGAATCCAAGCAATATATATGTTGCCATCCAACTCAGGAAGGGACTGGCTTTCTCGGAAAGAGTACAACCCAGCCAGTGTTCTGAAGACCTCT>Chimp-ECR14TAATCTATCAGACTAAGGTCCTGCAAGAAGACCTTGGTTACTGCAGGAAGAATTTGGGCTCAAATAAATAGAAGTTCAAATGGTAGAAATCTTTCTAACTGGAGACTCTTGGTCTTTGAGAAGTAGTTTGAATGTCAACATCTCACCCAGTTGCCAGATAAAACCTCGGTTCAACAACCCACCATGGAATTTTTCCCTGATTAATGAGCTAAATTCTGTACTGGAATCCAAGCAATATATATGTTGCCATCCAACTCAGGAAGAGACTGGCTTTCTCGGAAAGAGCACAACCCACCCAGTGTTCTGGAGA>Chinese-hamster-ECR14CATATGGTAGTGTGGGGAAGACTTGAGCCTAACCTAAATAAATAGAAAAGGAATTTCGGTGGTAGAAGCCTCCCTGACAGGAGACTCTCAGTCTTTCAGAAATAGTTTCAATGTCAACATATGGTCCAGGTGCCAGATAAAACTTTGCTTCAACAACCCACCTCGGAATTTTTTTCCCTGATTAATGAGCTAAGTCATGGACTGAAATCCAAGCAATATATACATGCTGCCGTCCAGCTCAGGAAGAGACTGGCTTTCTCGGAAAGAGTACAACCCAGTCAGTGTTCTGAGCGC>Cow-ECR14TAAATAAAATAATTTTTTAAAAAAAGACAGCATCTGTAGCCACAGGAAGGATCTGGGCCCAACTGAAACAAATCGAAGAGGTTCAGATGGCAGAAGCCGTTCTGACTGGAGACTCTCAGTCCACCAGAAGTCGTCTGAATGTCAACATCAGGCCCCGTTGCCAGATAAAACCCCAGTTCAACGACCCACCACGGAATTTTTCCCCTGATTAATGAGCTGGATGCCGGACTGGAATCCGAGCAATATGTACATTGCCATCCCGCCCGGGGAGGGACCGACTTTCTCAGAAAGAGCACAGCCCAGCCAGTGTTCTGGAAGCC>Dog-ECR14TGTAGGGTCTTTCGAGACAGTCTCGGTTGCTACAGGAAGGACATGGGCCCAACCTAAATAAATAGAAAAGAGGTTTAGATGACAGACCTTTCTGACTGGAGACTCTTAGTCCTTGAGAAGGAGTTTGAATGTCAACATTGGGCCCAGTTGCCAGATAAAACCGCAGTTCGACAACCCACCATGGAATATTTCCCTGATTAATGAGCTAAATGCTGGAGTGGAATCCAAGCAATATATATGTTGCCATCCAACTCATGAAGGGACTGGCTTTCTTGGAATGAGTACAACCCAGCCAGTGTTCTTAAGACTT>Dolphin-ECR14TCTAGGGTCCTTCAAGACAGCGTCTGTTGCCTCAGGAAAGATTCGTGCCCAACCTAAATAAATAGAAAAGAGGTTTCGATGGCAGAAGCCTTTCTGAGTGGAGGCTCTCAGTCCGTGAGAAGTCGTTTGAATGTCAACATCTGACCCAGTTGCCAGATAAAACCTCAGTTCAGCAACCCACCGTGGAGTTTTTCCTTGATTAATGACCTCAATGCTGGACTGCAATCCAAGCAATATATATGTTGCCATCCAACTCAGGAAGGGACTGGCTTTCTCGGAAAGAGCACAACCCAGCCAGTGTTCTGAAGAC>Elephant-ECR14TGCTCTTGGGTCCTCCATGAAGGCCCCAGTTGCTATAGGAAGGATCTAGACCTAACCTAAATAAATATAAAAGATGTTTACATGATAGAAACCTTTCTAACTGGAGGGTTGTAGTCCTTGAGAAGTCGTTTGAATGTCAACATCTGGCCCAGTTGCCAGATAAAACCTCAGTTCAACAACCCACTATGGAATTTTCTCCCTGAGTAATGAGCTAAATGCTGGACTGGAATCCAAGAAATATATACGTTGTGATCCAACTCAGGAAGGGAATGGCTTTCTCGGAAAGAACACAACCCGGCCAGTGTTTCGGAGA>Ferret-ECR14GGTCTTTCAAGACAGGAAGGATTTGGGCCCAACCTAAATAAATAGAAAGAAGTTTAAATGACAAACCTTTCTGACTGGAGACTCTTAGTCCTCAAGAAGGAGTTTGAATGTCAAAATTGGGACCAGTTGCCAGATAAAACCTCAGTTCAACAACCCACCATGGAATTTTTCCCTGATTAATGAGCTAAATGCTGGACTGGAATTCAAGCGGTATATATGTTGCCATCCAGCTCAGGAAGGGACTGGCTTTCTCGGAAAGAGTACAACCCCGCCAGTGTTCTGAAGACCTC>Gibbon-ECR14CTGCAAGAAGACCTTGGTTACTGCAGGAAGAATTTGGGCTCAAATAAATAGAAGTTCAGATGGTAGAAATCTTTCTAACTGGAGACTCTCGGTCTTTGAGAAGTAGTTTGAATGTCAACATCTTGCCCAGTTGCCAGATAAAACCTCGGTTCAACAACCCACCATGGAATTTTTCCCTGATTAACGAGCGAAATTCTGTACTGGAATCCAAGCAATATATATGCTGCCATCCAACTCAGGAAGAGACTGGCTTTCTCGGAAAGAGCACAACCCACCCAGTGTTCTGGAGA>Gorilla-ECR14GCTCACAGAGGAATCTGGGCCAGACTTAAACTGAAAGGAGGGCTCCCATGAGAGGGAAGTTTCTCCCGGAATCTCACGGCTTTTTGGACGAGGCTGTAAGAATGTCAACATTCAGCCAAATGCCAGATAAATCAACCTACTTGGAATTTTCCCTGATTAGTGTCCTGAATGCCAGGGTGAAGCCCAAAATCTCTCTGAATCTCCCCCTCTCGCCTTCTCACTCAGGAAGGGAATGGCTTTCTCGGAAAGAGTAAAATGTGGCCAGGATTCTGGGCCTGTTCATTTTCAATATGTTCATAA>Horse-ECR14GGCTGCCACAGGAAGGATTTGGGCCCGACCTAAATAAATAGGACAGCGGCTTAGACGGCGAGACCTTTCTGACTGGGGACTCTTAGTCCTCGAGAAGTAGTTCAATGTCAACATCTGGCCTAGTTGCCAGATAAAACCTCAGTTCTACAACCCACCATGGAATTTTCTCCTGATTAATGAGCTAAATGCTGAAGTGGAATCCAAGCAATATATACATTGCCATCCAACTCAGGAAGGGACCGGCTTTCTCGGAAAGAGCACAACCCAGCCAGTGTTCTGAGGACCTCCAT>Manatee-ECR14CAAGTTGATTTTGGATCCTCCATGAAGGCCCTGGTTGCTACAGGAAGGATCTTGGCCTAACCTAAATAAATAGAAAAGATGTTTAGATGGTAGAAGTCTTTCTAACTGGAAGCTTGTAGTCCTTAAGAAATCTTTTGAATGTCAACATCTGGCCCAGTTGCCAGATAAAACCTCAGTTCAACAACCCACTATGGAATTTTCCCCCGGATTAATGAGCTAAACACTGGACTGGAATCCAAGAAATATATATGTTGCCATCCAACTCAGGAAGGGAATGGCTTTCTCAGAAAGAGCACAACCTGGCCAGTGTTTCGGAGA>Marmoset-ECR14CTTCAGGAAGACCTTGGTTATTGAAGGAAGAATTTGCGCTCGAATAAATGGAAGTTCGGACGGTAGAAATCTTTCTAACCGGAGACTCTTGGTCTTTGAGAAGTAGTTTGAATGTCAACATCTCTCCCATTGCCAGATAAAACCTCAGTTCAACAACCCACCACGGAATTTTTCCCTGATTAACGAGCTAAATTCTGTACTGGAATCCAAGCAATATATATGTTGCCATCCAACTCAGGAAGAGACTGGCTTTCTCGGAAAGAGCACAACCCACCCAGTGTTCCGGAGAC>Megabat-ECR14CTTCAGGAAGGTCTAAGTTGCTACAGGAAGGATTTGGGTCCAACCCAAACAAACAGAAAAGAGATTCAGAAATGTTTCTGACTAGAGATGCTTAGCCCTTCAGAAGTAGTTTGGATGTCAACATCTGGCCCTGCTGCCAGATAAAACCTCAGTTCAACAACCCGCCATGGAACGTTTCCCTGATTAATGAGCTAAATGCTGTACTGGAGTCCAAACAACATATACATGTTGCCATTGACTCAGGAAGGGACTGGCTTTCTTGGAAAGAGCACAACCCGGCCAGTGTTCTGAGG>Microbat-ECR14CGCTGCAGGAAGGATTTGGGCCCAACCTCAACACGCAGGGGAGGCTCAGATGGCAGCGACCTCTCCCCCCGGAGCCGCCTAGTCCTTGAGAAGAAGTCTGAATGTCAACATCTGGCCCCGCTGCCAGATAAAACCTCAGTTCAACAACCCGCCGCGGAATGCGCCCTGATTAACGAGCTACACGCTGGACTGCAGTCCAAGCAACATATAGACCTGCCATCCGGCCCGGGAAGGGCCCGGCTTTCTCGGAAAGAGCACAACCCGCCAGTGTTCCTCCATT>Minke-whale-ECR14TGTTGCCGCAGGAAAGATCTGTGCCCAACCTAAATAAATAGAAAAGAGGTTTCGATGGCAGAAGCCTTTCTGAGTGGAGACTCTAAGTCCATGAGAAGTCGTTTGAATGTCAACATCTGACCCAGTTGCCAGATAAAACCTCAGTTCAACAGCCCACCGTGGAGTTTTTCCTTGATTAATGACCTCAATGCTGGACTGGAATCCAAGCAATATATATGTTGCCATCCAACTCAGGAAGGGACTGGCTTTCTCGGAAAGAGCACAACCCAGCCAGTGTTCTGAAGACCTCT>Mouse-lemur-ECR14GGTTGCCAATGGAAGGATTGGCTACCTAAATAAATAGAGAAGTTTAGACGTAGACTTTATGACTGTAGCCTCTAGTCTTTGAGAAGTAGTTTGAATGTCAACATCTAGTCTAGTTGCCAGATAAAACCTCAGTTCAACAACCCACCATGGAATTTTTCCCTGATTAATGAGCTAAATTCTGTGCTGGAATCCAAGCAATATATATGCTGCCATCCAACTCAGGAAGAGACTGGCTTTCTTGGAAAGAGCACAACCAGCCAGTGTGCTGGAGAC>Orangutan-ECR14AGAAGACCTTGGTTACTGCAGGAAGAATTTGGGCTGAAATAAATAGAAGTTCAGATGGTAGAAATCTTTTCTAACTGGAGACTCTTGGACTTTGAGAAGCAGTTTGAATGTCAACATCTCGCCCAGTTGCCAGATAAAAACCTTGGTTCAACAACCCACTATGGAATTTTTCCCTGATTAACGAGCTAAATTCTGTACTGGAATCCAAGCAATATATATGTTGCCATCCAACTCAGGAAGAGACTGGCTTTCTCGGGAAAGAGCACAACTCACCCAGTGTTCTGGAGACC>Panda-ECR14TCTCGGTTGCTACAGGAAGGACTTGGGCCCAACCTAAATAAACAGAAAAGAGGTTTAGAAGACAAACCTTCCTGACTGGAGACTCAGTCCTTGAGAAGGAGTTTGAATGTCAACATCGGGCCCAGTTGCCAGATAAAACCTCAGTTCAACAACCCACCATGGAATTTTTCCCTGATTAATGAGCTAAATGTTGGACTGGAATCCAAGCAATATATATGTTGCCATCCAACTCAGGCAGGGACTGGCTTTCTCGGAAAGAGTACAGCCCAGCCAGTGTTCTGAAGACCTCT>Pika-ECR14TCCCTCAAAAAGGCCTTAGTTGTTGCAGGAAGCATTTGGGCCTAACCTAAATAAATAGGAGTTTAGGTGGTAGAAATCTTTCTGACTGGAGACACAGTTTTTCAGAAGTAGTTAGAATGTCAACATCTGGCTCAGTTGCCAGATAAAACCTTGGTTGAACAACCCACCATGGAATTTTGTCTGATTAATGAGCTGCATTCTGGACTGGAATCCAAGCAGCCTATACATTGCCATCCAGCTCAGGAAGGGACTGGCTTTCTCGGAAAGAGCACAGCCCAGCCAGTGTTCTGGAGACCTCAA>Rabbit-ECR14GCTTACAGAGGAATCTGGGCCAGACTTAAAACTGAAAAGTGGTTCATATGGGAGACAAGTTTCTCCCAGAATCTCACGGCCCTTTGGACGAAGCTCTAAGAATGTCAACATTCAGCCCAAGTGCCAGATAAATCAACTTACCTCGGAATTTTCCCTGATTAATGAGCTAAGTGGTAGTGTGGCGTCCAAGTCCTGTATATTCTGCCGTCTTAACTCAGGAAGGGGCTGGCTTTCCTGGAAACAGTAAAATGTGGCCAGTGTTCCGGAG>Rat-ECR14CATCTGGTAGTGCGGGGAAGACTTGAGCCTAACCTAAATAAATAGAAAAGGAATTTTGGTGGTCGAAGACTCTGACAGGAGACTCTCAGTCTTTCAGAAATAGTTTCAATGTCAACATCTGGTCCAGGTGCCAGATAAAACTTTGCTTCAACAACCCACCTCGGAATTTTTTTCCCTGATTAATGAGCTAAGTCGTGGACTGAAATCCAAGCAATATATACATGCCGCCACCCAGCTCAGGAAGAGACTGGCTTTCTCGGAAAGAGAACAACCCAGCCAGTGTTCTGGGTGC>Rhesus-ECR14CTTCAAGAAGACTTTGGTTACTGCAGGAAGAATTTGGGCTCAAATAAATAGAAGTTCAGATGGTAGAAATCTTTCTAACTGGAGACTCTTGGTCTTTGAGAAATAGTTTGAATGTCAACATCTCGCCCAGTTGCCAGATAAAACCTCGGTTCAACAACCCACCATGGAATTTTTCCCTGATTAACGAGCTAAATTCTGTACTGGAATCCAAGCAATATATATGTTGCCATCCAACTCAGGAAGACACTGGCTTTCTCGGAAAGAGCACAACCCACCCAGTGTTCTGGAGA>Rock-hyrax-ECR14AAGGCCCCAGTTGCTACAGGAAAGATCTAGGCCTAACCTAAATAAACAGAAAAGTTGTTTAGCTGGTAGAAACCTTTCTAACTGGAGGCTCTTTAGTCCTTGAGAAGTCGTCTGAACGTCAGTATCTGGCCCAGCTGCCAGATAAAACCTCAGTTCAACAACCCGCTATGGAATTTTCCTCCTGATTAATGAGCTAAATGCTGGACTGGAATCCAACAAATATATATGTTGTGATCCAGCTCAGGAAGTGAATGGCTTTCTCGGAAAAAGCACAACCCAGCCAGTGTTTT>Sheep-ECR14ATAAAATACATTAAAAAAAAAAAAAGACAGTATCTGTAGCCACAGGAAGGATCTGGGCCCAACTGAAACAAATCGAAGAGGTTCAGATGGCAGAAGCCGTTCTGACTGGAGACTCTCAGTCCACCAGAAGTTGTCTGAATGTCAACATCAGGCCCCGTTGCCAGATAAAACCCCAGTTCAACGACCCACCACGGAATTTTTCCCCTGATTAACGAGCTGGATGCTGGGCTGGAATCCGAGCAATATACACATTGCCATCCAACCCAGGGAG>Shrew-ECR14CAGGAAGGATTTTGGGCCCAAGCTAAATAAATGGAGGAGAGATGGCCATGGCAAAAGCCTTGCTGACAGGAGATGCTCAGCCTCGAGGAGCAGTTGTTCGTGTCAACATCTGGTCTGACTGCCAGATAAAACCTCACTTCAACCACTGGGCCTGGAATTTTCCCTTGATTAATGAGCTCAGTCGGGACTGGAATCCAAACCATATAGACAGTCCCAGAATTCTGAGAAAAAGCACTCCCCGCCAGTGTTCCAAAGCCCTGATTCCAAACCCTCTGAGTATGCAG>Sloth-ECR14GGCCCTTAGAGAAAATCTGAGCCAAATTTAAACGGAAAAGAGTTTCATATGGGAGAGAAATTTCTCTCAGAATCTCCACGGCTTTGGACAAAGTTCAAAGAATGTCAACACTCAGCCAAGTGCCAGATAAATCAACTTACTTCGGAATTTTCCCTGATTAATGACCTAACCTGTAGCCTGGAGTCCCAGCTCTCTCTATATATCACCTTCTCACGCAGGAAGGCAATGGCTTTCTCGGAAACAGCAGAATGTGGCCAGTATTCTGGGGACATTCATTTCCAAACCATAAACATATTTAATACTGGGTCTCTGTTTTGTAGATTATATTCTTGGATCCAGTT>Squirrel-ECR14GAGGAGGACGCGAGGGGGAGGGACCCATCTGGCTGAGGCTCAGCCTTGGAGAAACGGCTTGAATGTCAACATCTGGCTCCGCGTCCAGCTAGAACCTTGGTTCAACAACCCACCACGGAATTTTCCCCTGATTAATGAGCTACATTCTGGACGGCGATCCAAGCAATATAGATGTTGCCATCCAACTCCGGAAGGGACCGGCTTTCTGGGAAAGAGCACAGCCCAGCCAGTGTTCTGGGGACCTCGGTTC>Squirrel-monkey-ECR14CTTCAAGAAGACCTTGGTTATTGAAGGACAAATTTGGGCTCAAATAAGTAGAAGTTCAGACGGTAGAAATCTTTCTAACTGGAGACTCTCGGTCTTTGAGAAGTAGTTTGAATGTCAACATCTCGCCCATTGCCAGATAAAACCTCGGTTCAACAACCCACCATGGAATTTTTCCCTGATTAACGAGCTAAATTCTGTACTGGAATCCAAGCAATATATATGTTGCCTTCCAACTCAGGAAGAAGCTGGCTTTCTCGGAAAGAGCACAACCCACCCAGTGTTCTGGAGAC>Tarsier-ECR14CAAGAAGCCCTTGGTTGCTGCAAAGAAGATTTGGACTTAACTTAAATAAACTGAATAGCAGTTTAGATGGTAGGGATCTTTCTGACTGAAGACTCTTAGTCTTTGAGAGTAGTTTGAATGTCAACATCTGGCCCAGTTGCCTAATAAAACCTCGGTTCAACAACCCTCCATGGAATTTTTCCCTGATTAATGAGCTTAAATTCTGTACTGGAATTCAAGCAATATATATGTTGCCATCCAACTCAGGAAGAGACCGGCTTTCTCGGAAAGAGCACAGCCCAGCCAGCATTCTGGAGACCT>Tenrec-ECR14AGAGGTTCCGATGGCAGAAGGCTTTCTGACCAGAGGCTTGAAGTCTTTCGGAAATCGTTTGCAGGTCAACATCGGGCCCAGTTGCCAGGTAAAACCTTGGTTCAACAATCCGCCATGGAATTTTCCCCCTGATTAATGAGCTCAACGCTGGATTGGGATCCAAGATATATACACGTTTCAATCCAACTCAGGAAGGGGGATGGCTTTCTCGGAATGAGCACAGCCCAACCAGTGTTCCAGAGACCTCTAT>Tree-shrew-ECR14AGGCCCTTCAAGAAGGCCTCAGTTGCTATTGAAGGGTTCAGGGCTCGCCTAAATAAACAGGAAAGAAGCTTGGATGGCAGAAACCTTTCTGGCTGGAGACTTAGTCTTTGAGAGGCTGTTTGAATGTCAACATCTGGCCTAGATGCCCGATAAAAGCTTGGTTCAACAACCCACCGTGGAATTTTTCCCTGATTAATGAGCTAAATTCTGGACTCGAATCCAAGCAATATATCTGTTGCCATCCAGTTCAGGGAGGGACTGGCTTTCTCGGAAAGAGCACAACCTAGTCAGTGTTCTGAAGACCTCCACT>White-rhinoceros-ECR14TCCAGGGTCCTTCAAGGAGGCCTCGGTTGCCACAGGAAGGATTCGGGCCCAACCTAAATAAATAGAAAAGAGCTTGAGATGGCAGAAAGCTTTCTGACTGAGAAGTAGTTTCAATGTCAACATCTGGCCCAGTTGCCAGATAAAACCTCAGTTCTACAACCCACCATGGACTTTTTCCTGATTAATGAGCTAAATGCTGGAGTGGAATCCAAGCAATATATATGTTGCCATCCAACACAGGAAGGGACCGGCTTTCTCAGAAAGAGCACAACCCAGCCAGTGTTCTGAGCACCTCCATTT>Mouse-ECR15GTCTGCTGTTTCTCTGTAAGGAATTTGGTCAACAGCTTTTTTTTTCCCTGAAGAAAGTGAAGGATGTGAAATGGTGGGGTTGTGTAGCAGTAGAGACCTCCTAAGAAGACGATGGAGCCTTGGCTTGGCTCTGGGAAGGAAAAATGTTCTGACATTTCACTGATCCAGTGGAAAATGGAACGCCCAACCTCTGTTCAGCTCTGTCTCTGTGCGCCTCATTAATTCCCTAGAAAGAACTTCTGGAAGTCAAATTACTAGGTCACAGGGGATGAAAAGGTCTCATGACTTGCTTGGATTAGTAAATTTTACTCCTTGTCAGGAGGTTCAGC>Human-ECR15AACAGGCACCATTTCTGCCTTAAGGAACTAGGTGGACAACCTCAATCCCTGAGAAAGGGAAGGATTTTTGAAAGCATAATGGGTTCAACTTAGCTGGGTGGAGACCTCCTAAGAAGAGGTAAGAGTCTTGTTCTTTCTCTGTAAAAGGAAAATGGTTTTCTAACGTTTTACTGTTCCAGAGAAAGACAAAACGCCCAACCTCCATTTACATCTGTCTCAGCGCGCCTCATTAATTCCCTAGAAAGAACTGCTGGAAGTTGAATTACTGGGTCAAAGAGGATAAGAGGTCCTCGTGATTTGGTTATGTTACTGAGTTTTGTTCCAAGTCAGGCAGCTTGGCCATAACTCCTGTTTATTTATCACTGCATTTCTG>Alpaca-ECR15GGAAAGGGCTTGGTCTGTCTCTGTGAAAGGGAAATGTTTTTCTAACATTTCACTCTTCCAGAAAGAAAAAAAGAACACCCAGCCTCCATTCAGATGTGTGTCTGTGTGCCTCATTAGTTCCCTAGAAAGAACTTCTGGAAGTCCAGCGACTGGGTCAAAGCGGGTAGAAGTGCTCGTGATTTGCTTGTGTTCCTGCATTTTATCCCCAGCCTGGGGACTCGGCTGCCCTCCCTGTTTGTTAGA>Baboon-ECR15AGAAACAGGCACCGTTTCTGCCTTAAGGAACTAAGTGGGCGACCTCCCTCCCTGAGAAAGGGAAGGATTTTTGAAAGCATAATGGGTTCAACTTAGCTGGGTAGAGACCTCCTAAGAAGAGGTAAGAGTCTTGTTCCGTCTCTGTAAAAGGAAAATGGTTTTCTAACATGTTACTGTTCCAGGGAAAGACAAAACGCCCAACCTCCATTAGATCTGTCTCAGTGCGCCTCATTAATTCCCTAGAAAGAACTGCTGGAAGTTGAATTACTGGGTCAAAGAGGATAAAAGGTCCTCATGATT>Bonobo-ECR15AACGGGCACCATTTCTGCCTTAAGGAACTAGGTGGACAACCTCAATCCCTGAGAAAGGGAAGGATTTTTGAAAGCATAATGGGTTCAACTTAGCTGGGTGGAGACCTCCTAAGAAGAGGTAAGAGTCTTGTTCTTTCTCTGTAAAAGGAAAATGGTTTTCTAACGTTTTACTGTTCCAGAGAAAGACAAAATGCCCAACCTCCATTTACATCTGTCTCAGCGCGCCTCATTAATTCCCTAGAAAGAACTGCTGGAAGTTGAATTACTGGGTCAAAGGGGATAAAAGGTCCTCGTGATTTG>Chimp-ECR15AACAGGCACCATTTCTGCCTTAAGGAACTAGGTGGACAACCTCAATCCCTGAGAAAGGGAAGGATTTTTGAAAGCATAATGGGTTCAACTTAGCTGGGTGGAGACCTCCTAAGAAGAGGTAAGAGTCTTGTTCTTTCTCTGTAAAAGGAAAATGGTTTTCTAACGTTTTACTGTTCCAGAGAAAGACAAAACGCCCAACCTCCATTTACATCTGTCTCAGCGCGCCTCATTAATTCCCTAGAAAGAACTGCTGGAAGTTGAATTACTGGGTCAAAGGGGATAAAAGGTCCTCGTGATTTGGTTATATTACTGAGTTTTGTTCCAAGTCAGGCAGCTTGGCCATAACTCCT>Chinese-hamster-ECR15TGCTGTTTCTCCATAAGGAATTCAGTCTGCAGCTCTGGTTTTTTTTTTTTTTTTCTTTTTTTCCCTGAAGAAAATGAAGGATGTGGAATGGTGGGGTCATATAGCAGGGGAGAGACCTCCTAAGAAGAGGAAGGAGCCTCGGCTTGGCTCTGGGAAGGAAAAATGTTCTGACATTTCACTGATCCAGTGGAAAAAACGGAACGCCCAACCTCTCTCTGTTCAAATCTGCCTCTGTGCTTCTCGTTAATTCCCTAGAAAGAACTTCTGGAAGTCAAGCCGCTGGGTCAAAGGGGACAAAAGGGTCCCATGATCTCCTTGGATTAGTGAATTCTATCCCCTCAGGAGGTCCAGCCT>Dolphin-ECR15GCTGGCAGCCTCCCCCTCTGAGAAAGGGAAGGATTTGGAAGTATAAAGTATAGAGACCTCCTAGGAAGAGGGAAGAGCTTGGTCTGCCTCTGTGATGGGAAAATGTTTTTCTAACATTTTACTCTTTCAACAACAAAAAAACCCGAATGCCCAACCTCCTTTCAGATCTGTCTCTGTGTACCTCATTAATTCCCTAGAAAGAACTTCTGCAAGTCCAATTACTGGGTCAAAGGGGATGCATGGTCCTCGTGATTTGCTTGTATTCCTGAATTTTATTCCC>Gibbon-ECR15AGGAACAGGCACCATTTCTGCCTTAAGGAACTGGGTGGACAACCTCCATCCCTGAGAAAGGGAAGGATTTTTGAAAGCATAGTGGGTTCAACTTAGCTGGGTAGAGACCTCCTAAGAAGAGGTAAGAGTCTTTTTCTGTCTCTGTAAAAGGAAAATGGTTTTCTAACGTTTTACTATTCCAGAGAAAGACAAAAATGCCCAACCTCCATTTACATCTGTCTCAGTGTGCCTCATTAATTCCCCGGAAAGAACTGCTGGAAGTCGAATTACTGGGTCAAAGGGGATAAAAGGTCCTCATGA>Gorilla-ECR15AACAGGCACCATTTCTGCCTTAAGGAACTAGGTGGACAACCTCAATCCCTGAGAAAGAGAAGGATTTTTGAAAGCATAATGGGTTCAACTTAGCTGGGTGGAGACCTCCTAAGAAGAGGTAAGAGTCTTGTTCTTTCTCTGTAAAAGGAAAATGGTTTTCTAACGTTTTACTGTTCCAGAGAAAGACAAAACGCCCAACCTCCATTTACATCTGTCTCAGCGCACCTCATTAATTCCCTAGAAAGAACTGCTGGAAGTTGAATTACCAGGTCAAAGGGGATAAAAGGTCCACGTGATTTGGTTATATTACTGAGTTTTGTTCCAAGTCAGGCAGCTTGGCC>Megabat-ECR15CTGGGTAAAGACTTCCTGGGAAGAGGGAAGAACCTCTGTGAGGGGAAATGTTTTTCTAACATTTCACTCTCCAGAGGAAAAACAGAATGCCCCACCTCCATTCAGATCTGTCTCTGGACCTCATTAATTCCCTAGAAAGAACTGCTGGAAGTTGAATTACTGGGTCAAAGGGAATGCAAAGTCCTGATGACTTGCTCGTATTCCTGAATTTGATTCTGAATCTGGCAGCT>Microbat-ECR15AGCTGGGCAGAAACCCCCTTAGGAATAGGAAGTAGCCTTGGTCTATCTCGGTGAATGGAACATGTTTTTCTAGCATTTCACTCTTCAGAGAAAAACAACAGAACGCCCAGCCTCCACACAGCTCCGTCTCTGGGCCTCATTAATTCCCTAGAAAGAACCGGTGGAAATGGAATTACTGGGTCAATGGGGATGCAAGGGCCCCGTGACTTGCTGGTATTCCTGAATGTTATTCCAAGCCAGGCGGCTCAGCCATAATCC>Minke-whale-ECR15AACTCTGACTGATACTCAATATACACCATTTCTGCTTTAGGGAACTGGGCTGGCAGCCTCCCCCTCTGAGAAAGCGAAGGATTTGGAAGTATAAAGGGTGGAGACCTCCTAGGAAGAGGAAAGAGCTTGGTCTGCCTCTGTGATGGGAAAATGTTTTTCTAACATTTCACTCTTTCAGAAAAAAAAAAAAAAACCGAATGCCCAACCTCCATTCAGATCTGTCTCTGTGTGCCTCATTAATTCCCTAGAAAGAACTTCTGCAAGTCCAGTTCCTGGGTCAAAGGGGATGCAAGGTCCTCGTGATTTGCTTGTATTCCTGAATTTTATTCCCAGCCGGGTGGCTCAGCCAT>Rat-ECR15TGCTGTTTCTCTGTAAGGAATTCAGTCAGCAGCTTTTTTCCCCCGAAGAAAGTGAAGGATGTGAAATGGTGGGGTTGTGTAGCCGTAGAGACCTCCTAAGAAGATGATGGAGCACTGGCTTGGCTCTGGGAAGGAAAAATGTTCTGACATTTCACTGATCCAGTGGAAAATGGAACGCCCAACCTCTGTTCAGCTCTGTCTGTGTGCGCCTCATTAATTCCCTAGAAAGAACTTCTGGAAGTCAAATTACTAGGTCAAAGGGAATGAAAGGGTCTCATGATTTGCTTGGATTAGTAAATTTTATCCCTTGTCAGGAGGCTCAGCC>Mouse-ECR16TCTATGCCCATCACCACTATCTCTGAGGGAGATTTTGCAACTCTGTGGCCTTAGCAACAGAGGCAGGCCCAGACCATCACCATGGAAACTGCATCAGGACTGCATAAAGATGGGAGGAGCTGGGAACTGGAATTTTCCATTTTTTGGATTCAGTTCTAAACCATGACTGGCACACAGTGGTCCTTCATTAAATGTTTATTGAGAAAACAAATACCTGAAACCTAACGCAGACAGCAGGGGCTGGA>Human-ECR16GAGATTAATCAACCTATGTGACCTGCTCCCTCTCCTGACCGTTTATCTGCCCTTAGCAACAGAGGGAGGCTCAGACTATCACCATGGAAACTGCAGCTGGAGGGCGGAAAGGTGGGGGGAGCTGAGGAAGGGGCCGGGAGGGCCCCGGAAACTGGAATTTTCCAGTCTTTGGAGTTGGTTCTTAACCATGACCTCTGACACAGTAGACCTGTATGAAGCATTTATTGAAAGAATAAAGAACTGAGCCCCAAGGAGTGGGAGTCCCCCACTCACTTTTCTCTCCGATGAGTAATGAAGCCC>Alpaca-ECR16CGATTAGTCAACTCGCGTGGCCCCGCTGCCTCCCGAGATTGCTGATCTGCCCTTAGCAACAGAGAGAGGCTCAGACCATCACCATGGAAACTGCAGCCGAGGTGGAGAAGGGGAGGGGAGGAGGAAGGGGCGTGTGGAGCCCTGCAAACTGGAATTTTCCAGACTTTGGAGTCCGTTACGAACCCTGACCTGGCACACAGTGTGCCTTCCTTAGATACTGACTGAAATAAGGAATGATGGAGCCTCAAGG>Baboon-ECR16ATTAATCAACCTATGTGACCTGCTCCCTCTCTTGACCATTTATCTACCTATAGCAACAGAGGGAGGCTCAGACTATCACCATGGAAACTGCAGTTAGAGGGCGGAAAGGTTGGGGGAGCTGAGGAAGGGGCTGGGAGGGCCCCGAAAACTGGAATTTTCCAGTCTTTGGAGTCGGTTCTTAACCATTACCTGTCACACAGTAGACTTGTATGAAGCGTTTATTGAAAGAATAAAGGACTGAGCCCCAAGA>Bonobo-ECR16GAGATTAATCAACCTATGTGACCTGCTCCCTCTCCTGACTGTTTATCTGCCCTTAGCAACAGAGGGAGGCTCAGACTATCACCATGGAAACTGCAGCTGGAGGGCAGAAAGGTGAGGGGAGCTGAGGAAGGGGCTGGGAGGGCCCCGGAAACTGGAATTTTCCAGTCTTTGGATTGGTTCTTAACCATGACCTCTCACACAGTAGACCTGTATGAAGCGTTTATTGAAAGAATAAAGGACTGAGCCCCAAGGAGTGGGAGTCCCCCACTCACTTTTCTCTCCGATGAGTAATGAAGCCCC>Chimp-ECR16GAGATTAATCAACCTATGTGACCTGCTCCCTCTCCTGACTGTTTATCTGCCCTTAGCAACAGAGGGAGGCTCAGACTATCACCATGGAAACTGCAGCTGGAGGGCAGAAAGGTGAGGGGAGCTGAGGAAGGGGCTGGGAGGGCCCCGGAAACTGGAATTTTCCAGTCTTTGGATTGGTTCTTAACCATGACCTCTCACACAGTAGACCTGTATGAAGCGTTTATTGAAAGAATAAAGGACTGAGCCCCAA>Chinese-hamster-ECR16ACCACTATCTTGGAGGAAGATTTAGCAACCCTGTGCCCTTAGCAACAGATTCAGGCCCAGACCATCACCATGGAAACTGCATCAGGACCGCATAGAGGTGGGAGGAGCTGGTAACTGGAATTTTCCAGTCTTTGGATTCAATTCTAAACCATGACTGACACACAGTGGCCCTTCGTTAGATGTCTATTGAGAAAATGAAT>Dolphin-ECR16GAGATTCATCCACCGGTGTGACCCTCTCCCTCCCAAGATTGCTGATCTGCCCTTAGCAACAGAGAGAGGCTCAGACCATCACCATGGAAACTGCAGCCACGAGGAGAAGGGGAGGGGCTGAGGAAGGGGCCCGGGGAGCCCAGCGAACTGGAATTTTCCAGTCTTTGGAGTCCGTTGTGAACCCTGACGTGGCACACAGTGTGCCTTCCTTAAATATTTGTTGAAATAATGAATGGCTGAACCACAAAGC>Ferret-ECR16GAGCCCCTTTTGAGTCTCCGTGCCCACCCCTCCTGTTCCCACCCCCCTGTATTGGTGAGATTAGCCAACCCATGGCCTGCTCCGTCTTGTCTCCAGATTGCTGTGGCCCTTAGCAACAGAGGGAGGCTCAGGCTGTCGCCATGGAAACTGCAGCTGAGTGGAGAAGGGAGGGTGGGAAAGGGGCCCAGAGAGCCCCTAAAACTGGAATGTTCCAGTCTGGAAGCCTGAGCTGATGCACAGTAGGCCTTCCTT>Gibbon-ECR16GAGATTAATCAACCTATGTGACCTGCTCCCTCTCCTGACCGTTTATCTGCCCTTAGCAACAGAGGGAGGCTCAGACTATCACCATGGAAACTGCAGCTGGAGGGCGGAAAGGTGGGGGGAGCTGAGGAAGGGGCTGGGAGGGCCCCGGAAACCGGAATTTTCCAGTCTTTGGAGTTGGTTCTTAACCATGACCTCTCACACAGTAGACCTTTATGAAGCATTTATTGAAAGAATAAAGGACTGAGCCCCA>Gorilla-ECR16CCTCTCCTGACCGTTTATCTGCCCTTAGCAACAGAGGGAGGCTCAGACTGTCACCATGGAAACTGCAGCTGGAGGGCGGAAAGGTGGGGGGAGCTGAGGAAGGGGCCGGGAGGGCCCCAGAAACTGGAATTTTCCAGTCTTTGGAGTTGGTTCTTAACCATGACCTCTCACACAGTAGACCTGTATGAAGCGTTTATTGA>Manatee-ECR16AACCTTTTGGTTAGCAGCCAAGCTCGTTAACCATTGCACCAGCCAGAGACTCCAAACTCCTTAAGGGAGTATCATATTTACCACTGTGTGGTCACTCTGCCTGGCACATAATAGTGATTCATTAAATGTTTATTGAATGAATGAATGAACAAATGACCACCCCCTACAAGATATATTCATACTAGCCTTCCTGTTCTGTATTTGCTGTGGTATCTCTGAATGGATGTACAAATGTGGCAGGGGTGAGAGGTGGG>Marmoset-ECR16GGGAGATTAATCAACCTTTGCAACCTGCTCCCTCTCCTGACTGTTTATCTGCCTTAGCAACAGAGGGAGGCTCAGACTATCACCATGGAAACCACAGCCAGAGTGCGGAAAGGTGGGGGAGCTGAGGAAGGGGCCGGGTGGGCCCCAGAAACTGGAATTTTCCAGTCTTTGGAGTCGGTTCTTAACCATGTCCTGTCACACAGTAGACCTGTAGGAAGCATTTATTGAAAGGGTAAAGGACTGAGCCCCAGAGCCGCCTTCATCAAGTCCCCT>Panda-ECR16CCCTTCCGAACGCCTGTGTCCGTCCCTCCGGCACCCACCACCGTGTCTCAGTGGGATGAGTCAACCTCTTGGCCTGCTCCCTCTCCAGATCGCTTATCGCCCTTAGCAACAGAGGGAGGCTCAGACCATCGCCATGGAAACTGCAGCTGAGCAGGAGAAGGGGAGGGTGAGGAAGGGGCTCACAGAGCCCCTGAAACTGGAATGTTTCAGGAACCCTGAGCTGACACACAGTAGGCCTTCCTTAA>Rabbit-ECR16CACTGTCACCTCTGAGGGTGACCAGTCAGGACTGTGCTTCTCTAGATGCTTGTGGTCCTTAGCAACAGAGGCAGGCACAGACCATCACCATGGGAACTGCAGCTGAGGGCAGAGCTGGGGTAGCTAAGAGGGGCTGCAGAGCTCTGGGAACTGGAATTTTCCAGTCTTTGCAGTCAGTTCTGAACCATGACCTGGCACACAGCGGGTCCTTGTTAGCTATTTACCAAAATAATAAACAGTGCCAAGGCAC>Rat-ECR16CTATGCCCATAACCACTATCTCTGAGGGAGATTTAGCAACTCTGTGCCCTTAGCAACAGAGGCAGGCCCAGACCATCACCATGGAAACTGCTTCAGGACCGCATAAAGGTGGGAGGAGCCAGGAACTGGAATTTTCCACTCTTTGGATTCAGTTCTAAACCATGACAGGCACACAGTGGTCCTTCATTAAATGTTTATTGAGAAAATGAATGACTGAAACATAATGCATACAGCAGGGGC>Squirrel-monkey-ECR16AGGGAGATGAATCAACCTGTGAACCTGCTCCCTCTCCTGACTGTTTATCTGCCTTAGCAACAGAGGGAGGCTCAGACTATCACCATGGAAACCACAGCCAGAATGCCGAAAGGTAGGGGAGCTGAGGAGGGGGCTGGGAGGGCCCCAGAAACTGGAATTTTCCGGTCTTGGGAGTCGGTTCTTAACCATGTCCTGTCACACAGTAGACCTGTATGAAGCATTTATTGAAAGAATAAAGGGCTGAGCCCCACCGAGTAGGGGTC>White-rhinoceros-ECR16CTCCATGGATTAGTCAACCCACGTGGCTTGCTCCCTCCCAAGACTGCAGATCTGTCCTTAGCCACAGAGAGAGGCTCAGACCATCACCATGGAAACCGCAGCCGAGAGGGAAGGGGAGGGGCTGAGGAAGGGGCCCGGGGAGACCAGGAAACTGGAATTTTCCAGGTTTTGGAGTCGGTTATAAACTTGCCCTGGCACACAGTGGACCTTCCTTAAATATTTATTGACAGAATGAATGACTGCGCCTCAAGGCACGTGGTGGGGAGTCCCCCGTCCCCACTGTCCGTTTT>Mouse-ECR17GGCCCAAATGCCAGCCATGTTGAGATTGAGCAAAGCCGTGCTGTACAATAGGAATTTCTCCCACAAGCATATCAGAAAGGAAGTTTGGCATCATGGTGTGGAAATGTGAAACGTGCTCACACTAGAGCCAGCTGTGTACTGAGCTCCTCACAAACACATAGGACAGGAATGCATTGTGTCAGACAAGGGAGAGAAAAGGCAGCCAGCCCCAGGAAATTAAAGAAAAGCTGGCTATCGGCTGCATCTAGGGACACGGCGTGCCGAGCAGACAGCTGCGGTTTCAATACTCTGCTGGCCATGGGGAGCTGGTTCCAAGAAGCTGCTGACCCCCTGAGTCCTGTGGAAATTCAGTTACTTCCTGGACCTCAGGTGAACCTTTAGCTCACCTCCCATAGGATTCCACCCACAGTGAGTCCTGGGAGCCCGG>Human-ECR17AATCATCCACCCAGCTCCAAACGTCATGCTGTAGTTTGGAATTGCCTTCAACAAGCATATCTGAAAACAGGTTTGTATCGTGGCAAGGAAACGTGAAATGTGCTCACATAAGAGCCAGCTGTGCGCTGCACCCCTTTACAAGCATGTAGGACAGGAATTCATTATGTCAGACAATGAGAAGAAAAGACAGCCAGCCCCAGGAAATTAAAGAAAAGCTGGCTATCAACAGCATCTAGGGGCACAATCTGCAAAGCAGACAGCTGCAATTTGAATGTTTGGATGGTTCAGAAATGGACAGTTCTGCACTGCTGGCCATGATGAGCTGTTTCCAAGAGCTACTCCCTTCCTGAGTTTCCTGGAGATGCATATATTTCCTGGACCTCAGCTGAACCCTGGGTTTGTCTCTTGGAGGGAACAATCCGTGGTGGGTCAGT>Alpaca-ECR17ACAGCACCCGGGACAGCTGGCGCCGTCCGTGATGCCGAGGCTGAGAAAAGCCATGCTCTGTGTGGAATTCCCTTCAGCAAGCTTATCTGAAAACAGGCTTGCATCATGGCATTGAAATGTGAACTGCGTTCCCACGAGAGCCAGCTGTGTGCTGAACCCCCTTGCAGGCACGCAGGACAGGCGTCTGTTCTGTCAGACGCGGGGAGGAAAGACAGCCAGCCCCAGGAAACTGGGAAGCACGGGCTGTCGCTGGCATCTAGGGACATCTCTGCAGAGCAGACAGCTGCATTGTGAGTGCTTGGGCGGTTTGAAGATGGACAGCCCCGCGGTGCTGGCCACGGCAAGTTGTTCCCGAGAAGCCACTGTCTGCCTGGGCCTCCGCTCATC>Baboon-ECR17ATGTGCTCACATAAGAGCCAGCTGTGCGCTGCACCCCTTTGCAAGCATGCAGGACAGGAATCCATTATGTGAGACAATGAGGAGAAGAGACAGCCAGTCCCAGGAAATTAAAGAAAAGTTGGCTCTCCGCAGCATCTAGGGACATAATCTGCAAAGCAGACAGCTGCAAGTTAAATGTTTGGATGGTTCAGAAATGGACAGTTCTGCACTGCTGGCCATGATGAGCTGTTTCCAAGAGCTACTCCCTTCGTGAGTTGCTTGGAGATGCATGTATTTCCTGGACCTCAGCTGAACCCTGGGTTTGTCTCTTGGAGGGAACAATCCGTGGTGGGTCAGTGAGCTGAGCTGGA>Bonobo-ECR17TTCAACAAGCATATCTGAAAACAGGTTTGTATCGTGGCAAGGAAACGTGAAATGTGCTCACATAGGAGCCAGCTGTGCGCTGCACCCCTTTACAAGCATGTAGGACAGGAATTCATTATGTCAGACAATGAGGAGAAAAGACAGCCAGCCCCAGGAAATTAAAGAAAAGCTGGCTATCAACAGCATCTAGGGACACAATCTGCAAAGCAGACAGCTGCAATTTGAATGTTTGGATGGTTCAGAAATGGACAGTTCTGCACTGCTGGCCATGATGAGCTGTTTCCAAGAGCTACTCCCTTCCTGAGTTGCCTGGAGATGCATATATTTCCTGGACCTCAGCTGAACCCTGGGTTTGTCTCTTGGAGAGAACAATCAGTGGTGGGTCAGTGAGCTGAGCTTG>Chimp-ECR17ATCATCCACCCAGCTCCAAATGTCATGCTGTAGTTTGGAATTGCCTTCAACAAGCATATCTGAAAACAGGTTTGTATCGTGGCAAGGAAACGTGAAATGTGCTCACATAAGAGCCAGCTGTGCGCTGCACCCCTTTACAAGCATGTAGGACAGGAATTCATTATGTCAGACAATGAGGAGAAAAGACAGCCAGCCCCAGGAAATTAAAGAAAAGCTGGCTATCAACAGCATCTAGGGACACAATCTGCAAAGCAGACAGCTGCAATTTGAATGTTTGGATGGTTCAGAAATGGACAGTTCTGCACTGCTGGCCATGATGAGCTGTTTCCAAGAGCTACTCCCTTCCTGAGTTGCCTGGAGATGCATATATTTCCTGGACCTCAGCTGAACCCTGGGTTT>Chinese-hamster-ECR17TGAGCAAAGCCAGGCTGTACAATAGGAATTTCTCCCACGAGCATATCAGAAAGCAAGTTTGGCATCGTGGCGTGGAAATGTGAAATGTGCTCACACTAAGAGCCAGCTGGGTCCCAAGCCCCTCACAAGCACATAGGACAGGAATGCATTGTGTCAGACAAGGGAGAGGAAAGGCTCCAGCCCCAGGAAATTAAAGAAAAGCTGGCTATCTGCTGCATCTAGGGACATGGCGTGCCGAACAGACAGCTGCGCTTTCGATGCTTTGCTGGCCTCCATGAGCCGTTTCCAAGAAGCCGCTAACCCCCTGAGTCCTGTGGAAAGGCAGCTAGTTCCTGGACCTCAGGTGAACCTTGAGCTCACCTCCCATAGGGTTCCACCCACGGTGAGTCCTGGGAGGCAA>Gibbon-ECR17CACACACAACAAAGAATCATCCAACCAGCTCCAAATGTCATCTGTAGTTTGGAATTTCCTTTAACAAGCATATCTGAAAACAGGTTTGTATCGTGGCAAGGAAACGTGAAATGTGCTCACATAAGAGCCAGCTGTGCACTGCACCCCTTTACAAGCATGTAGGACAGGAATTCATTATGTCAGACAATGAGGAGAAAAGACAGCCAGCCCCAGGAAATTAAAGAAAAGCTGGCTATCAGCAGCATCTAGGGACACAATCTGCAACGCAGACAGCTGCAATTGAATGTTTGGATGGTTCAGAAATGGACAGTTCTGCACTGCTGGCCATGATGAGCTGTTTCCAAGAGATACTCCCTTCCTGAGTTGCCTGGAGAGGCATATATTTCCTGGACCTCAGCTGAACCCTGGGGTTGTCTCTTGGAGGGAACAATCCGTGGTGGGTCAAAGAGC>Gorilla-ECR17AATCATCCACCCATCTCCAAATGTCATGCTGTAGTTTGGAAATGCCTTCAACAAGCATATCTGAAAACAGGTTTGTATCGTGGCAAGGAAACGTGAAATGTGCTCACATAAGAGCCAGCTGTGCGCTGCACCCCTTTACAAGCATGTAGGACAGGAATTCATTATGTCAGACAATGAGAAGAAAAGACAGCCAGCCCCAGAAAATTAAAGAAAAGCTGGCTATCAACAGCATCTAGGGACACAATCTGCAAAGCAGACAGCTGCAATTTGAATGTTTGGATGGCTCAGAAATGGACAGTTCTGCACTGCTGGCCATGATGAGCTGTTTCCAAGAGCTACTCCCTTCCTGAGTTTCTTGGAGATGCATATATTTCCTGGACCTCAGCTGAACCCTGGGTTTGTCTCTTGGAGGGAACAATCCGTGGTGGGTCAGTGAGCTGAGCTTGAGTC>Horse-ECR17AAACCCACACTATGATACGGAATTTCTTTCAACAAGCACATCTGAAAACTAGTTTGCATCATGGCATTGAAATGTGAAACGTGCTCGCCGGAGAGCCGGCTGTGTGCTGGACTCCCTTCCCCGAGGCAGGCCAGGAATCCGCTATATCGGACCATGAGGAGAAAAGACAGCCAGCCCGAGGACGTTAGAGAAAATCTGGCCATCGATGGAATCTAGGGACTTAATCTGCAGACAGCTGCAGTTAAATATTTGGACGGTCTGAAAAGGGACAACTCTGCATCGCTGGCCGTGCTGGGTTGTTCCCAAGAAGCTGCTGTCCTCTGGAGCTCTGTGGGAACGCGTATATTTCCTGGACCTCTGCTGGGCCTTTAGCTCATCATCCTGGGGAAAATTCCACAGGGGGTCATTGAGCCAAGCTGTTGTTTGGCAAGTGGACTCAGTCTGGACATT>Marmoset-ECR17GGACACACACAACAAAGAGTTATCTAGCTCCAAACGTCATGCCATAGCTCGGAATTTCCTTCCACAAGCATATCTGTAAACAGGTTTGTATCGCGGCGAGGAAACGTGAAATGTGCTCCCATAAGGGCCAGCTGTGCGCTGCGCCCATTTACGAGCATGTAGGACAGGAATTCATTATGTCAGACAATGAGGAGCAAAGGCAGCCAGCCCTAGGAAATTGAAGGAAAGCTGGCCATCGGCAGCATCTAGGGACGTGATCTGCAAAGCAGACAGCTGCGATCTAAATGTTTGCGTGGTTCAGAAATGGACAGTTCCGCACTGCTGGTCGTGATGAGCTGTTTCCAAGAGCTACTCCCTTCCTGAGTTGCTTGGAGTTGCATATATTTCCTGGACCTCAGCTGAACCCTGGATTCGTCTCTTGGAGGGAACAATCCCGGGTGGGTCGGTGAG>Megabat-ECR17GAAATGAGCCCCCATGAAAGCCAGTTGTGCGCTGACCCCCTTTCAGGCATGTAAAACAGGGATCAGTTATGTCAGACCGTGAGGAGAAAGGACAGCCGGTCCCAGGAAATTCGGGGGGAACTGGCTATCGGCGGTGTCTGGGGACGTAACCTGCAAAGCAGACAGCTGCGATTAAAATATTTGTACAGTTTGAACATGGACAGCACTGTATCACTGGCCATACTGAGTTTTTCCCA>Mouse-lemur-ECR17AGCCTTCCTACAACAAGGAATTGTCCACCGCGAACTGTCAGGCTGCAGTGTGGAGTCTCCGCCAATAAGCTCATCTGGAAACAGGATTGCATTGTGGCAGGGAAATGTGAAATGTGTTCACACAAGAGCCAGCTGTGCACGGGGCCCCCGTGCAAGCATGCAGGACAGGAACTCATCATGTCAGACAGGGAGGAGGAGAGGCAGCCAGCCCCAGGAAATTAAAGAAAAGCTGGCTATCAGCAGCACCTAGGGACGCCCGCGCAAAGCTGACAGCTGCAACTCACGTGTTTGGACAGTCTGGGAACGGACGGTTCTGCATTGCTGGCTGTGATGGGCTGTTTCCAAAAAGCTAC>Orangutan-ECR17CACACAAAACAAAGAATCATCCACCCAGCTCCAAATGTCATGCTGTGGTTTGGAATTGCCTTCAACAAGCATATCTGAAAACAGGTTTGTACAGTGGCAAGGAAACGTGAAATGTGCTCACATAAGAGCCAGCTGTGCTCTGCACCCCTTTACAAGCATGTAGGACAGGAATTCATTATGTCAGACAATGAGGAGAAAAGACAGCCAGCCCCAGGAAATTAAAGAAAAGCTGGCTATCAGCAGCATCTAGGGACACAATCTGCAAAGCAGACAGCTGCAATTTGAATGTTTGCATGGTTCAGAAATGGACAGTTCTGCACTGCTGGCCATGATGAGCTGTTTCCAAGAGTTACTCCCTTCCTGAGTTGCCTGGAGATGCATATATTTCCTGGACCTCAGCTGAACCCTGGGTTTGTCTCTTGGAGGGAACAATCCATGGTGGGTCAGTGAGCTGAGCTTGAGTTGCAACTGGACTGTCTTGACTTTCTAACTTCATCTTGTTGGAC>Panda-ECR17ACAGAGAACGATCCGGCTTGACATATCCACGGCGCCGAGGCTGAGAAAACCCATGCTATGTGTGGGATTTCCTGCAGCAAGCGTATCTGAAAGCGGGTCTGCATCATGGCAGGGAACTGTGAAACGTGCTTGCGCAGGGGCCAGCTGTGTGCTGAACCCCCTCACAGGCACGTAGGACAGGAATCCGTTATGTCAGGCCATGAGGAGAAAAGACAGCTGGCCCCAGGAAATTAGGGGAAAACTGGCTATCGACAGACAGCCTCTGGGGACATAATCTGCGGGCGGACAGCTGCAATTTGCCGTTTGGCATGTTTGAGAGCAGACAGCCCTGCGTTGCTGGTCATGCCGCAC>Rabbit-ECR17TTCACAGGGCATATCCGAAAGCAGGTTTGCGTCATGGCGTGGAAATGTGGAACGTGCTTCTACGAGAGCCTGCTGTGTGCTGAGCCCCCTGACGAGCATGTAGGACAGGAATTTATTATGTCAGACAAGGAGCAGAAAAGGCAGCCAGGCCAGGAAATGAAAGAAAAACTGGCTGTCGGCAGCATCTAGGGACCCCGTGTGCAGGCAGACAGCTGCAGGCGCAGCGTGGGGACGTGGTGGGAGGGAACTGCTCTGCGCTGCCAGCCGTGATGAGCTCTTTCCGAGAAGATCCCCCCTTCCTGAGTTTTGTGGAAAT>Rat-ECR17GGTCCAAATGCCAGCCATTCTGAGACTGAGCAAAGTCATGCTGTACAATAGGAACTTCTCCCACAAGCATGTCGGAAAGCAAGTTTGGCATCACGGTGTGGAAATGTGAAATGTGCTCACACTAGAGCCAACTGTGTACTGAGCCCCTCACAAACACATAGGACAGGAATGCATTGTGTCAGACAAGGGAGAGAAAAGGCAGCCAGCCCCAGGAAATTAAAGAAAAGCTGGCTATCGGTTGCATCTAGGGACACGGCGTGCCGAGCAGACAGCTGTGGTTTCAATGCTCTGCTGGCCATCGTGAGTTGCTTCCAAGAAGCTGCTGACCCCCTGAGTCCTGTGGAAATGCAGTTACTTCCTGGACCTCAGGTGAACCTTTAGCTCACCTCCCATAGGATTCCACCCACAGTGAGTCCTGGGA>Rhesus-ECR17CCACACACACAACAAAGAATCATCCAGCTCCAAATGTCATGCTGTTGTTTGGAATTTTCTTCAGCAAGCATATCTGAAAACAGGTTTGTATCATGGCAAGGAATCGTGAAATGTGCTCACATAAGAGCCAGCTGTGCGCTGCACCCCTTTGCAAGCATGCAGGACAGGAATTCATTATGTGAGACAATGAGGAGAAGAGACAGCCAGTCCCAGGAAATTAAAGAAAAGTTGGCTCTCCGCAGCATCTAGGGACATAATCTGCAAAGCAGACAGCTGCAAGTTAAATGTTTGGATGGTTCAGAAATGGACAGTTCTGCACTGCTGGCCATGATGAGCTGTTTCCAAGAGCTACTCCCCTCGTGAGTTGCTTGGAGATGCATGTATTTCCTGGACCTCAGCTGAACCCTGGGTTTGTCTCTTGGAGGGAACAATCCGTGGTGGGTCAGTGAGCTGAGCTGGA>Squirrel-monkey-ECR17GAAAGCCCCCAGCACACACACAACAAAGAGTTTTCTAGCTCCAAACGTCATGCTGTAGTTTGGAATTTCCTTCAACAAGCATATCTGTAAACAGGTTTGTATTGAGGCAAGGAAACGTGAAATGTGCTCCCATAAGAGCCAGCTGCGCACTGCACCCATTTACAAGCATGTAGGACAGGAATTCATTATGTCAGACAATGAGGAGAAGGCAGCCAGCCCCAGGAAATTAAAGAAAAGCTGGCCATCGGCAGCATCTAGGGACATAATCGGCAAAGCAGACAGCTGCGATTTCAGTGTTTGCATGGTTCAGAAATGGACAGTTCTGCGCTGCTGGCCATGATGAGCTGTTTCCAAGAGCTACTCCCTTCCTGAGTTGCTTGGAGATGCATATATTTCCTGGACCTCAGCTGAACCCTGGATTTGTCTCTTGGAGGGAACAATCCAGGGTGGGTCGGTGAGCCGAGCTTGGC>White-rhinoceros-ECR17AGCTTGAAATGTGAGCAGTGCTGTGGTTGAGAAAACCCACACTGTCGTACGGAATTTCCTTCCACAAGCGTGTGTGAACACCAGTTTGCATCACGGCGTTGAAATGTGAAACGTGCTCACACCAGAGCCAGCTGTGTGCTGAATGCCCTCACAGGCGTGCCGGACAGGAATTCATTATGTCAGACCAGGAGGAGAAAAGACAGCCAGCCCCAAGAAATTAGACAAAAACTGGCCATCGACGGAATCTGGGGACATAGTCTGCAGACAGCTGCAATTAAATGTTTGGACGGCTTGAAAATGGACAGCTCTGCCTCGCTGGCCATGCTGGGTTGTTCCCAAGAAGCTACACTCTCCCTGAGTTCTGTGGGAACGCACATATTTCCTGGACCTCTGCCGGACTTTTAGCTCATCATGCTAGGGAAAATTCCATGGGGGGTGTTGAGCCGAGGTTTAGTCTGGCAACTGGACTCA>Mouse-ECR18TGCTGCCTGCCTGCCAGCAGAAGCCCCGGGCGGGTCAGAAGCAGTTTGACATACCGCAGCGGCCAACACAGCTGTTGAGGGGGGATTAGCTGCAGGGAAAGCCGATCCAGTGACAAATGGTTTAGGTATGTCCGCCCCTGGGAAATGTGATCTCCACGGTTTGCACTATAGAAACTTGTTTGTTTCAAAAGATGAGGAACATGTTTGTTGTCTGTGTCTCCAGAGAAATCACAACGTTGACAGCTCTGGATATACCCAAAGAGACAGAGTACAAAAAAACCAATTACACGTAGGCTGCGCTCATAATCAACAAATGCTGGTTTGGGAATTGCTGTGTGAAGATTCCTCTCGGCAGCCTGCTCAGAATGTACGTCTGGTGACTTCTGAGATGTCAC>Human-ECR18GCTCAAGGCTATCAACAGCAGCTCCCTTGCCAACAGCTTGCAGCAGCTGCCGCTGATCAAGGCAGTCAGCTCGATCCCCGTAGCCTTCCGTGAAGGACCACGGGCTGGTCAGAAGCAGTCTGACAGGTCACAGAGCCAGCCCAGCTGTTGGGGGGATTAGCTGTAAGGAAAGCTGATCCTATGACACATGCTTTAGGTATGTCCGCCCCTGGGAAACGTGACCTTCAAGGTTTGCGTTATAGAACTTTATTCGCTTCAAAAGATGGAGAACACTTTTGTTTTCTGAGTCCCTGGAGAATCATGATGCAGACAGCTTTGGAGGTACCCAAAGAAATGGAACACAGAATCCCAATTACATATAGGCCGCACACATGATAAACAACTGCTGGTTTGGGAATTGCTGGGCGAAGCTTCCTTTCGGCAGTTTGCTCAGAATATATTGCTGATGATTTCTGAGATGCTCAATTCATCATTCACAATTGCTTTGCAGTAAAATGCTTGCA>Baboon-ECR18GCTCATGGCTATCAACAGCAGCGCCCTTCCCAACAGCTTGCAGCAGCTGCCACTGGTCAAAGCGATCAGCTCGATCCCCGTCGCCTTCCATGAAGGACCACGGGCTGGTCAGAAGCAGTCTGACAGGTCACAGAGCCAGCCCAGCTGTTGGGGGGATTAGCTGTAAGGAAAGCCGATCCTATGACAAACGCTCTAGGTATGTCCGCCCCTGGGAAACGTGACCTTCGAGGTTTGCATTATAGAACTTCGTTCGCTTCAAAAGATGGAGAACACTTTTGTTTTCTGAGTGTCCGGAGAATCATGATGCAGACAACTTTGGAGGTACCCAAAGAAACGGAACACAGAACCCCAATTACATGTAGGCCGCACACATGATAAACAACTGCAGGTTTGGGAATTGCTGGGCGAAGCTTCCTTTCGGCAGTTTGCTCGGAATATATTGCTGATGATTTCTGAGGTGCTCCATCCATCATTCACAATTGCATTGCAGTAAAATGCTTGCG>Bonobo-ECR18GCTCAAGGCTATCAACAGCAGCTCCCTTGCCAACAGCTTGCAGCAGCTGCCGCTGATCAAGGCAGTCAGCTCGATCCCCATAGCCTTCCGTGAAGGACCACGGGCTGGTCAGAAGCAGTCTGACAGGTCACAGAGCCAGCCCAGCTGTTGGGGGGATTAGCTGTAAGGAAAGCTGATCCTATGACACATGCTTTAGGTATGTCCGCCCCTGGGAAACGTGACCTTCAAGGTTTGCGTTATAGAACTTTATTCGCTTCAAAAGATGGAGAACACTTTTGTTCTCTGAGTCCCTGGAGAATCATGATGCAGACAGCTTTGGAGGTACCCAAAGAAACGGAACACAGAATCCCAATTACATATAGGCCGCACACATGATAAACAACTGCTGGTTTGGGAATTGCTGGGCGAAGCTTCCTTTCGGCAGTTTGCTCAGAATATATTGCTGATGATTTCTGAGATGCTCAATTCATCATTCACAATTGCTTTGCAGTAAAATGCTTGCA>Bushbaby-ECR18TGATCAAGGTGGTCAGCGCCACTTCCTGCCGCCATCCATATCAGCCCTGAGGCTGGTCAGAGGCAGTCTGACATGCCGCAGGGCTGGCTCAGCTGCTGGAGGATTAGCTGTAAAGAAAGCTGATCCTATGACAAATGGTTTAAATATGTGCACCCCTGGGAAATGTGACCTTCAAGGTTTGCATTATAGAATTTCTTTGCTTTAGGAGATGGAGAACACGTTTGTTTTCTGAGTTCTAGGAAAATCTCAATGCAGACAACTTTAGACATACACAACAAAACAGAATGCAAAATGCCAATTACATGTAGGCTGAATACATAACAAACAGCCACTGGTTTGGGAATTGCTGCGTGAAGCTTCTCTTTGCTTGGAATGTAGGGCCAGTGATTCCTGAGATGTTACATTCATCACTTGTAATTGCTTTGAGATAAGATGCGTGCAGAAGGGAAAATATCT>Cat-ECR18CCTCGGCAGTTCGGCCGCAGCTGCCGGTGGTCAAGGCGGTCAGGCCGCCCTCGGCCTCCTCCGCTGGGCCCCACGTGCTGCTCAGAGTCAAGGGCCAGAGCAGCTGTCGGGGGGATTAGCTGTCAGGAAAGCCGATCCCATGACAAACGTTTCAGGTATGTCCGCCCCTGGGAGGCGCGAGCTCCAAGGTCTGTACTATAGAACTCGCTGGTGTCGGAAGACGGAGAACATGTTTGTTTTCTGAGTCCTCAGAGGCCCGTGATGGGGACATCCCTGAACATACCCAGAGGATCGGAACACGCAGGCTGCACACACCATAAAGAAGGGGCCGTTTGGGGGTCTC>Chimp-ECR18GCTCAAGGCTATCAACAGCAGCTCCCTTGCCAACAGCTTGCAGCAGCTGCCGCTGATCAAGGCAGTCAGCTCGATCCCCATAGCCTTCCGTGAAGGACCACGGGCTGGTCAGAAGCAGTCTGACAGGTCACAGAGCCAGCCCAGCTGTTGGGGGGATTAGCTGTAAGGAAAGCTGATCCTATGACACATGCTTTAGGTATGTCCACCCCTGGGAAACGTGACCTTCAAGGTTTGCGTTATAGAACTTTATTCGCTTCAAAAGATGGAGAACACTTTTGTTCTCTGAGTCCCTGGAGAATCATGATGCAGACAGCTTTGGAGGTACCCAAAGAAACGGAACACAGAATCCCAATTACATATAGGCCGCACACATGATAAACAACTGCTGGTTTGGGAATTGCTGGGCGAAGCTTCCTTTCGGCAGTTTGCTCAGAATATATTGCTGATGATTTCTGAGATGCTCAATTCATCATTCACAATTGCTTTGCAGTAAAATGCTTGCA>Chinese-Hamster-ECR18GAAGACCTGAGTTTGGGCCTCCAGAAGTCACATAAAAACCTGGCATGGTGGCTCTGGCCTCTAACCCTAGCACTGAGGGCTCAGAGACAGGTTCACTGGCCCAGAGACAAATGGTTTAGGTATGTCCGCCCCTGGGAAACGTGATCTTCAAGGTTTGAGCTATAGAACTTGTTTGTTTCAAAAGATGAGGAACACGTTTGTTGTCTGTGTCTCCAGAGAAATCACAATGTTGACAGATCTTTGGATATACCCAGAGACAGAGTACAAAAAAAAAAAAAAAAAAAAAAAAAGAAAGAAAGAAAGAAAAAAAAAAGGAAAAGAAAAGAAAAGAAAAAAAAAAAAACCAATTACACATAGGCTGCGCTCATAATCCACAAATGCTGGTTTGGGAATTGCCGTGTGAAGATTCCTCTCGGCAGCCTGCTCAGAATGTACGTCTGGTGACTTCCCAGATGTCACAGTCATCACCCACAATTACTATGTAATAAAAACACTTCCCCAAAGGAAGATATCCGTGAGCACGGCTCCTCACGGGAGGAAGCCTGCAGCCACTGGAGCC>Cow-ECR18CGACAGTCTGGCGGCAGCTGCCAGGCGATCAAGGCGGGGACGCGGGCCTCTGCGGCCTTCACGTGCGGCTCAGAATCAGCCTGCGAGCACCGGGGCCGTGCAGCTGTCGGGGGATTAGCTGTTAGGAAAGGCGATCCTGTGACGAATGGGTCAGGTATGTCCGCCCCTGGGAAGCCGAAGCTCCAAGATCTGCCTGATAGGAAACCGGTCGGCTTGCAACAACGGAAAACTCGTTTGTCTTCTGCGTCCTTGTTGGGGAGTCGTGCTGTGGAC>Dog-ECR18GCCGCCTTCAGTGGAGACCACGTGCTGGTCAGGGGCAGCTTGACGTGCCACAGGACCAGTGCAGCTGTCGGGGGATTAGCTGTAAGGAAAGACAATCCCATGACAAACGTTTCAGGTATGTCCGCCCCTGGGAAGCATGTGCTTGAAGGTTTGCATTATAGAACTTGATGGTATCAAAGGATAGAGAACATGTTTGTTTTCTGAGTCCCCGGAGAGTTGCGACGGAGACATCTTTGAACGTACCCAGAGAAACAGAACACTAGGTGTGAATCACACATAGGCTGTGTGCACCATAAAGAGCAG>Gibbon-ECR18GCTCATGGCTATCAACAGCAGCGCCCTTGCCAACAGCTTGCAGCAGCTGCCGCTGATCAAGGCAGTCAGCTTGATCCCCGTCGCCTTCCGTGAAGGACCACGGGCTGGTCAGAAGCAGTCTGACAGGTCACAGAGCCAGCCCAGCTGTTGGGGGGATTAGCTGTAAGGAAAGCTGATCCTATGACAAATGCTTTAGGTATGTCCGCCCCTGGGAAACGTGACCTTCAAGGTTTGCATTATAGAACTTTGTTCGCTTCAAAAGATGGAGAACACTTTTGTTTGCCGAGTCCCCGGAGAATCATGATGCAGACAGCTTTGGAGGTACCCAAAGAAATGGAACACAGAATCCCAATTACACGTAGGCCGCACACATGATAAACAACTGCTGGTTTGGAAATTGCTGGGCGAAGCTTCCTTTCGGCAGTTTACTCAGAATATATTGCTGATGATTTCTGAGGTGCTCAATTCATCCTTCACAATTGCTTTGCAGTAAAATGCTTGCA>Gorilla-ECR18GCTCATGGCTATCAACAGCAGCTCCCTTGCCAACAGCTTGCAGCAGCTGCCGCTGAACAAGGCAGTCAGCTCGATCCCCGTAGCCTTCCGTGAAGGACCACGGGCTGGTCAGAAGCAGTCTGACAGGTCACAGAGCCAGCCCAGCTGTTGGGGGGATTAGCTGTAAGGAAAGCTGATCCTATGACAAATGCTTTAGGTATGTCCGCCCCTGGGAAACGTGACCTTCAAGGTTTGCTTTATAGAACTTTGTTCGCTTCAAAAGATGGAGAACACTTTTGTTTTCTGAGTCCCTGGAGAATCATGATGCAGACAGCTTTGGAGGTACCCAAAGAAACGGAACACAGAATCACAATTACATATAGGCCGCACACATGATAAACAACTGCTGGTTTGGGAATTGCTGGGCGAAGCTTCCTTTCGGCAGTTTGCTCAGAATATATTGCTGATGATTTCTGAGATGCTCAATTCATCATTCACAATCGCTTTGCAGTAAAATGCTTGCA>Marmoset-ECR18TGCCGCAGCTGCCGCTGAGCAGGGCGGTGGATCCCCACGGCCTTCCGTGAAGGAGCAGGGGCTGGTCGGAAGCATGGTCTGATGGGCCACTGGGCCAGCCCAGCTGTTAGGGGGGATTAGCTGTAAGGAAAGCCGCTCCCATGACACATGCCTTCGGTATGTCCGCCCCTGGAAAACGTGACCTTCCAGGTTTGCATTACAGAACTCCGTTCGCTTCAAAAGGTACAGAACACTTTTGTTTTCCGAGTCCCCGGGGTAGCACGATGAACACAACTTTGGAGGTACCCAAAGAAACG>Orangutan-ECR18GCTCATGGCTATCAACAGCAGCGCCCTTGCCAACAGCTTGCAGCAGCTGCCACTGATCAAGGCAGTCAGCTCGATCCCCGTCGCCTTCTGTGAAGGACCACGGGCTGGTCAGAAGCAGTCTGACAGGTCACAGAGCCAGCCCAGCTGTTGGGGGGATTAGCTGTAAGGAAAGCTGATCCTATGACAAATGCTTTAGGTATGTCCGCCCCTGGGAAACGTGACCTTCAAGGTTTGCATTATAGAACTTTGTTTGCTTCAAAAGATGGAGAACACTTTTGTTTTCTGAGTCCCCGGAGAATCATGATGCAGAGAGCTTTGGAGGTACCCAAAGAAACGGAACACAGAATCCCAATTACACGTAGGCCGCACACATGATAAACAACTGCTGGTTTGGGAATTGCTGGGCGAAGCTTCCTTTCGGCAGTTTGCTCAGAATATATTGCTGATGATTTCTGAGATGCTCAATTCATCACTCACAATTGCTTTGTGGTAAAATGCTTGCA>Rabbit-ECR18GCATGGTTTTCAGCAGCAGCGCAGCAGCCAACAGCACTGCAGCAGCTGCCGCTGATCAAGGAGGCCGGTGCAGTCCCCTGTCGCCTTCCATGGAGGGGCGCGGGCTGGTCAGAGGCAGTTTGACATGCCACAGGGCCGCGCAGCTGCCATGGGGATTAGCTGCCAGGAAAGCTGATCCGATGACAAATGGCCAAAGTATGTCCGCCCCTGGGAAACGTGACCTTCAAGGTTTGCATTATAGAACTTGTTTGCTTCAGAAGATGGAGAACGCCTGTGTTTCGGAGTCCCTGGAGAAGCACAATGGAGACAACTTTGGATGTACCCGGAGAAACAGAATACAAAATACCAATTACATGAGGCTGC>Rat-ECR18GACCAACTTTACCCGTGACCGCGATGATCGATCAACAGCTTTCCTGTCACCAACAGGTCTGCAGCTGCTGCCGCTGGGCAGGGCGGTCAGCTCTGTCCCCTGCTGCCTGCTTGCCAGCAGAGGCCCCAGGCGGGTCAGGAGCAGTTTGACATACCGCAGCGGCCAACACAGCTGTTGAGGGGGGATTAGCTGTAGGGAAAACTGATCCAGTGACAAATGGTTTAGGTATGTCCGCCCCTGGGAAACGTGATCTCCACGGTTTGCACTATAGAAACTTGTTTGTTTCAAAAGATGAGGAACATGTTTGTTGTCTGTGTCTCCAGAGAAATCACAACGTTGACAGCTCTGGATATACCCAAAGAGACGGAGTACAAAAAAACCAATTACACGTAGGCTGCGCTCATAATCAACAAATGCTGGTTTGGGAATTGCTGTGTGAAGATTCCTCTCGGCAGCCTGCTCAGAATGTACGTCTGGTGACTTCTGAGCTGTCACAGTCATCACCCACAATTACTATGTAATAAAAACACTTCCCCAAAGGAAGATATATCCACAAGCACTACTCCTCGCGGGAGGAAGCCTGCAGCCGCAGGAG>Rhesus-ECR18AGCAGCTGCCGCTGGTCAAGGCGATCAGCTCGATCCCCGTCACCTTCCGTGAAGGACCACGGGCTGGTCAGAAGCATTCTGACAGGTCACAGAGCCAGCCCAGCTGTTGGGGGGATTAGCTGTAAGGAAAGCTGATCCTATGACAAACGCTCTAGGTATGTCCGCCCCTGGGAAACGTGACCTTCGAGGTTTGCATTATAGAACTTCGTTCGCTTCAAAAGATGGAGAACACTTTTGTTTTCTGAGTGCCTGGAGAATCATGATGCAGACAACTTTGGAGGTACCCAAAGAAACGGAACACAGAATCCCAATTACATGTAGGCCGCACACATGATAAACAACTGCAGGTTTGGGAATTGCTGGGCGAAGCTTCCTTTCGGCAGTTTGCTCAGAATATACTGCTGATGATTTCTGAGGTGCTCTATCCATCATTCACAATCGCGTTGCGGTAAAATGCTTGCG>Sheep-ECR18CGACAGTCTGGCGGCAGCTGCCGGGCGATCGAGGCGGGGACGCGGGCCTCTGTGGCCTTCACGTGCGGCTCAGAATCAGCCTGCGAGTACCGGGGCCATGCAGCTGTCGGGGGATTAGCTGTAAGGAAAGGCGATCCCGTGACGAATGGGTCAGGTATGTCCGCCCCTGGGAAGCCGAAGCTCCAAGATCTGCCTGATAGGAAACCGGTCGGCTTTGCAACAACAGAAAACTCGTTTGTCTTCTGCGTCCTTGTCGGGAAGCCCTGCTAGAAC>Squirrel-monkey-ECR18TGCAGCAGCTGCCTCTGATCAAGGCGGTCGGTCCCCATGGCTTTCCGTGAAGGACCATGGGCTGGTCAGAAGCACAGTCTGATAGGCCATGGGGCCAGCCCAGCTGTTGGGGGGATTAGCTGTAAGGAAAGCTGATCCCATGACAAATGCTTTAGGTATGTCCGCCCCTGGAAAACGTGACCTTCGAGGTTTGCATTATAGAACTTTGTTTGCTTCAAAAGATAGAGAACACTTTTGTTTTCTGAGTCCCCGGGGTATCATGATGAAGACAATTTTGGAGGGACCCAGAGAAATGGAACACAGAATCCCAGTTACACATAGGCTGCACACACGATAAACAACTGCTGGTTTGGGAATTGCTGAGCTCAGCTTCCTTCGGGCAGTTTGCTCAGAATGTACTGCTGATGATTTCTGAGATGCTCTATTCATCATTCACAAGTGCGTTCCAGTAAGATGCTTGCA>Tarsier-ECR18GACATCCGTGAAGGGCCGCAGGGCTGGTCAGAAACAGCCCAACACGCTACAGGGCCAGCCCAGCTGTTGGGGAGATTAGCTATGAGGAAAGCTGATCCTCTGACAAATGGTTTAGGTATGTCCGCCCCTGGGAAATGTGACCTTCGAGGTTTGCATTGTCCAACTTGCTGGCTTCAAAAGATGGAGGACACTTTTTTGTTTCCTGAGTCCCCGGAGAATCGCAATGGAGACAACTTTGGACATGTCCAAAGAAGC
