# Supplementary material for: Phylogenetic and Epigenetic Footprinting of the Putative Enhancers of the Peg3 Domain
Source: PLoS One. 2016 Apr 22;11(4):e0154216. doi: 10.1371/journal.pone.0154216 (PMC4841594; doi:10.1371/journal.pone.0154216)
Supplement: S2 File — (PPTX) [file pone.0154216.s002.pptx]

## Slide 1
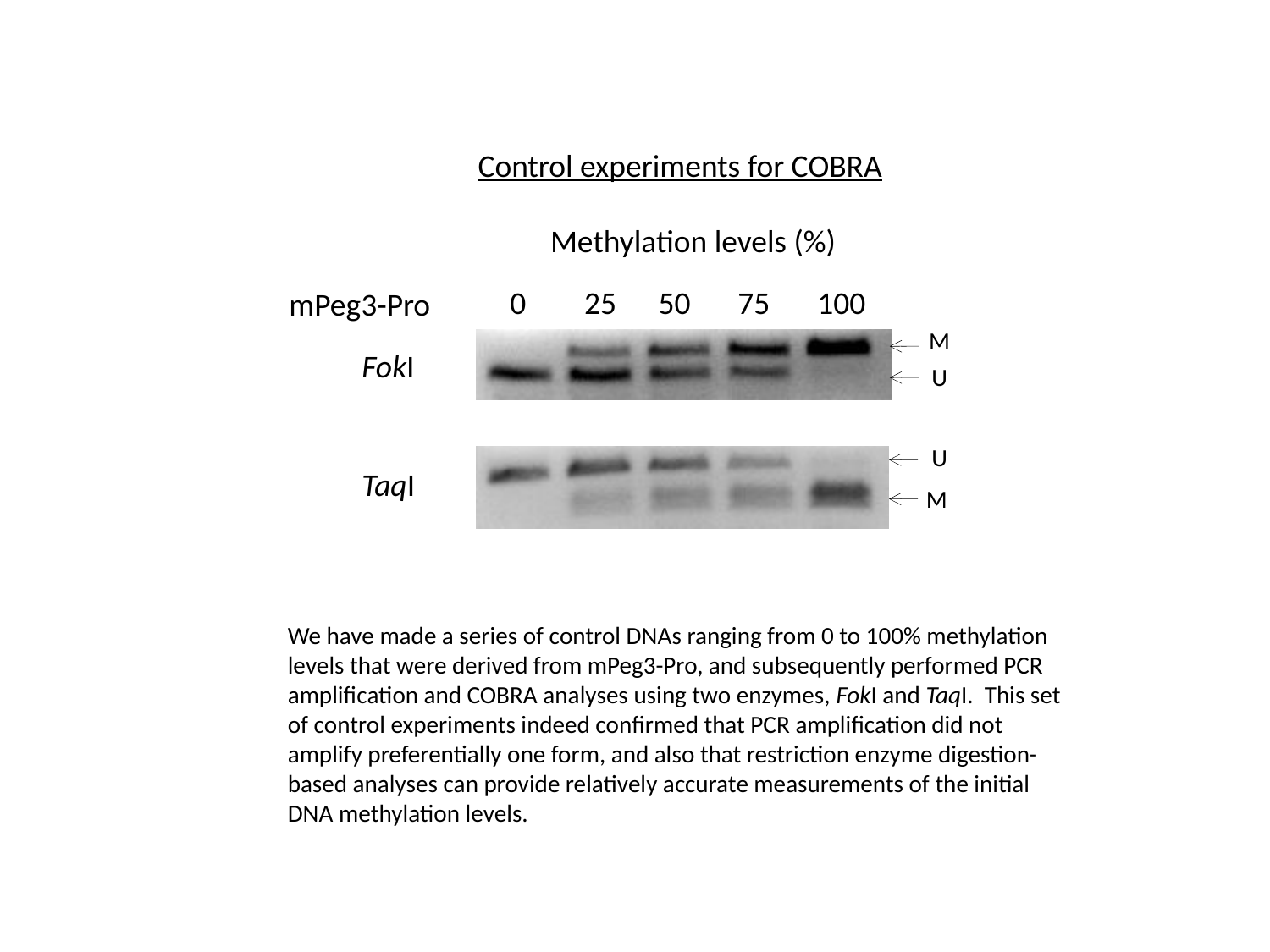

Control experiments for COBRA
Methylation levels (%)
0
25
50
75
100
mPeg3-Pro
M
FokI
U
U
TaqI
M
We have made a series of control DNAs ranging from 0 to 100% methylation levels that were derived from mPeg3-Pro, and subsequently performed PCR amplification and COBRA analyses using two enzymes, FokI and TaqI. This set of control experiments indeed confirmed that PCR amplification did not amplify preferentially one form, and also that restriction enzyme digestion-based analyses can provide relatively accurate measurements of the initial DNA methylation levels.
