# Supplementary material for: Phylogenetic and Epigenetic Footprinting of the Putative Enhancers of the Peg3 Domain
Source: PLoS One. 2016 Apr 22;11(4):e0154216. doi: 10.1371/journal.pone.0154216 (PMC4841594; doi:10.1371/journal.pone.0154216)
Supplement: S3 File — (PDF) [file pone.0154216.s003.pdf]

ECR1

```
Query        1    AAGCCTTAAGAGGT-----ATTTTTTCCCAGGTTACAAGCTAAAGGG--TGGCAGAG-CTAGAAATCAAACTCAAATCTGTTA-GATTCCTGAGCCTCAGCCCTCAACCACCACTC-AG-  109
Query_48935  58                              ............T..GT.T.GA.....GT-..C.G...G..TTC.......CC.-...G....-----..T.T...TA.....G-G.-  141
Query_48936  16            ....-----..C..G...........T....GTA.A--..AT....-TC..G.......C..G.....C.-..CG......T------...........G-G.-  109
Query_48937  1    ..............-----..C.......................--...G...-..........G.C..........-..............G...............A-G.-  109
Query_48938  1    ..............-----..C.......................--.......-....................................-.........................-..-  109
Query_48939  1    ..............-----..C.......................--.......-....................................-.........................-..-  109
Query_48940  21                            ........-...T....GTA..--...T....-TC..G......TC.C.......G.-...GT......TCT..G.T-------------.T-  95
Query_48941  18               ..-----..C..G...........T....GTA.T--...T....-TC..G.......C..........-..CG......T------...-.T.T....G-GA-  107
Query_48942  14                .-----..C..G.........-.TT....GCA..--..A....C-.C..G.-.....T........C.C.-...G.T.....T------.....T..TG..A-C.-  101
Query_48943  28                            ..T.....-...T....GTA..--...T....-TC..G.......C.....TC..G.-..CG....A.T------..........T.G-G.-  109
Query_48944  28   ..............-----..C..................T....--.......-..........................C..-..........................G-G.-  136
Query_48945  1     ...........A.-----G.C.......................--.......-...........................A.............................-..-  109
Query_48946  4                                                               ........C.........C.-..CAG......T------...........G-G.-  51
Query_48947  37    ......G......TAAT-..C..G...........C....GTA..--..A....T-.C..G.T.A..T.-.......C.C.-...G......TT------........TT...A-..-  141
Query_48948  2    .......C.A...-----..C........-..................--.......-...............C.......A.G-............G...T....G.......G-G.-  109
Query_48949  8     ......G......TAGT-..C..G...........T....CCA..--..TT.AGT-.A....-......C.....T...C.-..CA......T------............G-G.-  112
Query_48950  29                            .......C....T..C.GTA..--...T.A.T-.-..G.......-.........C.-..CG......T------..........A.G-G.-  109
Query_48951  25                         ..G...........T...GGTA.T--.........-TC..G.......C..........-..CG......T------...-.T......G-GA-  109
Query_48952  16                      ..C...........-.T.......T..C---........-....G.......C..-.....C.--.C.........GGA.T......T..T...G-GA-  106
Query_48953  1     ..............-----..C.......................--.......-....................................-.........................G-G.-  108
Query_48954  14   .......G......TAGTT..C.CA........-.........GTAA.--...A....-TC..G.......C.........------------------...T............G-G.-  109
Query_48955  17                              ..........C....GTA.A--.A.T...AT.C....-......C...G....CC.-..CA......T------.....G.......G-G.-  97
Query_48956  21                         ..C...T......T.GA....T...--..........-....C.G..................-TT.A.A...--..G...T.C...AA......G-G.A  111
Query_48957  27                                   ....T..A....T...--..........-....G.......T....G.....C.-A.G.TA.A.....G...T.C..........GG..-  110
Query_48958  62                              ............T..G..T.GA.....GT-..C.G...G..TTC.......CC.-...G.....A.T-----..T....TA......G-G.-  145
Query_48959  1     ..............-----..C.......................--.......-..........G.C..........-..............G...............A-G.-  108
Query_48960  80                                                                                                                ......CA-.A-  89
Query_48961  13                              ........T.T....GCAAA--...A....-.C..G.......C.........C.-..C....A.....G..GT.....T...A...T-G.-  99
Query_48962  2    .......C.A...-----..C........-..................--...T....-..............C.......A.G-............G...T....G.......G-G.-  109
Query_48963  19                         ........T.C..........CA..--......GA-.C..G.......C.....T...C.-.....-.A.....G...T.............G-G.-  109
Query_48964  1      .....G......CAGC-..C.......G.....--...G.GT..A--...TG..A-....G.........CA........C.-..C..........G.A.T.............G-G.-  109
Query_48965  35   .......G..G...TACT-..C..G......A..........GTA..--.........-TC.AG.......TC.........C.-..CA..C....T------............G-G.-  141

Query        110  TACATTTCTAGGCTATTAA-ATTTGAAAGCCATAATGAAATGGAACGTTCTTGAAAGGAAGGGAGATAAT-AAACACTTGGCAGA-CGTCAGGGGAGTACTTTCCTCAAAAAGTTCTCCA  226
Query_48935  142  ...................-......................T.T.A...G..----....T....TC.-..GA........-.-T....AA...A.G.C...A......T..G.....  253
Query_48936  110  ...................-.........................T.A...............C.......-...A........A.-T...GA...........--.........G...T.  224
Query_48937  110  .................C-..........................T.A.......................-...A........A.-T.................................  226
Query_48938  110  ......CT...........-...................................................-...A..........-T.................................  226
Query_48939  110  ...................-...................................................-...A..........-T.................................  226
Query_48940  96   ..-..G.............-.....................T.T.A...C...........C.......-...A........A.-T....A...........T.G...T.C.....T.  211
Query_48941  108  ...................-....A....................T.A......G......C.......-...A........A.-TA...A.....G.......T.......G..GT.  224
Query_48942  102  .......T....T.....-.......................C.T.A..T...........AC.......-...A..G.....A.TAA...A...G..G....T..........GG..T.  219
Query_48943  110  .....G.............-.....................AT.T.A.....G.......C.......-...A.T......A.-TA...A...........T.G...T.T.....T.  226
Query_48944  137  ...................-...................................................-...A........A.-T.................................  253
Query_48945  110  ...................-...................................................-...A..........-T....A............................  226
Query_48946  52   ..T............-...G.....................T.A..........T...C..---..-...A....A...A.-T....A...C..G.....T........G...T.  165
Query_48947  142  ...................-....................G...C.T.A........A....AC.......-...A..A.....A.-TA...A...G..G....T..........G...T.  258
Query_48948  110  ...................-............C...........T................A....G..-...A........C.-T......................C....A.....  226
Query_48949  113  ...................-...........G............T.A...............C.C.....-...A........A.-T....A.....G.....T.........GT..T.  229
Query_48950  110  .......G.........C-..C...................C..T.A...............C.......-...A........A.-T....A.....G.....T...G.....G...T.  226
Query_48951  110  ...............G..-.........................T.A......G......C.......-...A........A.-TA...A.....G.....T.T.......G...T.  226
Query_48952  107  ...........C....-.........T..........A..T.A...............C.......-...A........A.-T....A.....G...............CG...T.  223
Query_48953  109  ...................-...................................................-...A..........-T........................T.......  225
Query_48954  110  .....G.....A.......-......................T.T.A...............C........-...A..C.....A.-T.G..A...........T.G...T.C.....T.  226
Query_48955  98   ..............G....-......C.................T.A...............C....G.-..A........A.-T....AA....G...C.T..C......G...T.  214
Query_48956  112  ...................-..........GC..G........T.T..........C...C.......-...AG.......A.-T....A....A........T...  218
Query_48957  111  ...................-...........C...........T.T................A.......-...AG.......A.-T....A.A..A.........-....T..G...T.  226
Query_48958  146  ...................-......................T.T.A...G..----....T....TC.-G.GA........-.-T....AA...A.G.C...A......T.GG.....  257
Query_48959  109  .................C-..........................T.A.......................-...A........A.-T.................................  225
Query_48960  90   ..T.C....GT.....G..-.....................G...GT.A......G......C.......-...A.........AG-TA...A.....CG.....T..CG.....G.C.T.  206
Query_48961  100  C..................-........T.................T.A.........T....AC......A...A........A.-T.C...A.....G.....TC....T..AG...T.  217
Query_48962  110  ...................-.........................T................A.......-...A........A.-T...............................A.....  226
Query_48963  110  A........G.........TT......................T..T.A....----....A.C.......-G..A........A.-T....A....A.GT.......-.....CA.A.T.  222
Query_48964  110  ..G-......A........-C.....................C...T.A....G.....A..A.C....-..-..G.G.-.....A.-T....A..A...G..C..G..G...G..G...T.  223
Query_48965  142  ...................-.........................T.A..........C....C.......-...A........A.-T....A.A....G.....T.........G...T.  258
```

ECR2

```
Query         70   AAAAAGCATGGCCTCAAAAG-GTGACTGGAGTAGGAATGGAAACTAACCTCCAA-CTTTCAAGGAACAAATAATGTCTCTGTTCCATAAAGTGTTCCAAGGCCTAGAAAACAGTGGAGGG  187
Query_175872  102  ....G.....C.TGG...T.-......TT.......GCA......TC.T.....-.............G....CTCA..C..C...C....C..G..............C........TT  219
Query_175873  78   .........AT.T.T...G.-.CA......A..............TC.T.....-.......A........T---...........C.GT..............................  192
Query_175874  1                                                     .....-........................C........................................  70
Query_175875  1                                                     .....-.................................................................  70
Query_175876  64    .....T....TTAT.G.G.-......A..................C.T..T..-...................T....CA..G..A..G.........................A....  180
Query_175877  1                       .-.....CA.........CA......TC.T.....-..............G....T..............T........G........G............  99
Query_175878  8    ....................-.................................-.....C...........................................................  125
Query_175879  99   ....G.....A.TCG...T.-......TT...G...GCA......TC.T.....-.............G.....TCAC.C......C....CA.G..............C........TT  216
Query_175880  85    .........A.T.TTC...A...C....................TC.......-..............G....T..............T......................A.......  202
Query_175881  78   ............T.T...G.-........................----.....-..A.............T---..............C..............C...............  188
Query_175882  1                                           ......TC.T.....-.........TG......T.---..............C.........................T..  77
Query_175883  79   ..........A.T.TG..G.-...................G....TC.T.....-.............TG....T.A............CA..............G..............  196
Query_175884  53   ....................-.................................-.......................G.....................AA................A.  170
Query_175885  3    ....................-............A....................-.................................................................  120
Query_175886  21                        .......CT......G...C....TCTT.T...-................C..T.T.............TA..............C...T....A....  118
Query_175887  62    ...........T.T...G.-........................TC.T.G...-...................T...........C..T.........AA..................A  178
Query_175888  68   ............T.T...G.-...C...A..G.A...........TC.T.....-................C..T..............ACA............................  185
Query_175889  63   .........T..-...C...-..........G.....C........G.T.....-.........................--......................C..........C.A..  177
Query_175890  4    ..........A.T.T.....-.......A..............T.CT.T.....-...................T............T.T..............................  121
Query_175891  9    ...........TT.T...G.-...............T........----.....-................T---..............C...............A..............  119
Query_175892  13   ..........A.T.T...G.-C.......CAC..A..........TC.T..T..-...................T.T............G..............................  130
Query_175893  3    ...............G....-.................................-.................................................................  120
Query_175894  62   ..........A.T.TG..G.-........................TC.T.....-..............G....T..............T.....T..............T.........  179
Query_175895  9    ............T.T..CT.-A.....C..A..............----.....-................T---..............T............................A.  119
Query_175896  32                      .-..........G.....A.......TC.T.....-...................T...........T...A.A..GG...T.................A.  130
Query_175897  1                                 ................TC.T.T...-...................T.T.........C...A.A.......TT................A.  90
Query_175898  102  ....G.....A.TCG...T.-......TT..C....GCA......TC.T.....-.............G.....TCA..C..C...C....C..G..............C........TT  219
Query_175899  5      ..................-.................................-........................C........................................  120
Query_175900  59   .......TA...T.AC...T-...C.....AG.A..C......T.TC.T.....-...........T....C..TC.........................................CA.  176
Query_175901  14   ..........C.T.T...G.-........................TC.T.....A................T---...A.T......C.CA.............CA.............A  129
Query_175902  55                                          ..G....TC...T...-................C..T...--.......G.TA.....................A......C  133
Query_175903  1                         ......T.................TC.T.AA..-..............G....T.....--........A...........A..............A..  96
Query_175904  1                                           ......TC.T.....-.C.............C.C.T............C...A.G..........................  80
Query_175905  61    ...................-...........................T.....-...............C.............T................................A..  177
Query_175906  1                                                        ..-...................T-..................................T.........  66
Query_175907  41        .......TG....G.-...C.....AG.A..GC.-.....TC.T.....-................C..T..........C.G.C.C........T...T...........  148
Query_175908  44    .....T...A.T.T...GA-AC......................TT.T.....-...................T..................A.......T........  150
Query_175909  1                         .............A..C.-..GT.TC.T.G...-...................T...T.......C..T..........A...........T.......  97

Query         188  CTTCCCAGTTCATTTTAAT----TA----ACTGGTTCACAGAGGTTTACAATCCCTAAGCTGTTTTAA-TTGGAAAGAACGCTGCAGAATG-GAAATAACAACAGCTGGCAGTTATGGAA  297
Query_175872  220  ...........G.......----.----............C......TG.....CG..TGA......-......G....T.A...T....-A...............A.....  323
Query_175873  193  ...........C.......----.G----.T......................T....A.........-......G....T.C...TG...-A.......G.......A.......  298
Query_175874  71   ...................----.----.......................................-...........T..........-............................  180
Query_175875  71   ...................----.----.......................................-...........T..........-......................  174
Query_175876  181  .....T.............----.----.........G...-T.AA......TT.......-.....-...........T.....T....-A...............A.....  282
Query_175877  100  ........C..C.......----.----...A......................C............-......G...T...........-A...............AT..........  209
Query_175878  126  ...................----.----.....................C.................-...........T..........-............................  235
Query_175879  217  ...................----.----..........................CC..TGA......-......G....T.AA..T....-A...............A.G...G..C..  326
Query_175880  203  ........A..C.......----.----...A............................A......-..........TT.....T....-A........  293
Query_175881  189  ...........C...G...----.----.......................................-..........TT.....T....-A...............AT......A...  298
Query_175882  78   ...................----..ACTG.......................A..........C....-..........CT.....T....AA...GC..TG......A......CA...  192
Query_175883  197  .......AC.TC.......----.----...A...................................-..........TT.....T....-A...............AT....  300
Query_175884  171  ..................-----.----.......................................-...........T..........-............................  279
Query_175885  121  ...................----.----.......................................-...........T..........-............................  230
Query_175886  119  T..........C.G.....----.----.........T.............................-......T....T..T..T....-A...............A.....C....C  228
Query_175887  179  ...T.......C.......----.----.........................T...A.........-...........T.....T....-A...............A.....C.....  288
Query_175888  186  ..........TG.......TAAC.----...................T.......-.T....C....-..........CT.....T....-A...A....G...T..A......A....  298
Query_175889  178  ...................----.----.......................................-...........T.-C..T....-............................  286
Query_175890  122  ...........C.....G.----.----..............A........................-....C......T.....T....-A...---.........A...........  228
Query_175891  120  ...........C...G...----.----.......................................-..........TT.....T....-A.....G.........AT......A...  229
Query_175892  131  .....T.............----.----...........A............T..............-...........T.....T....-A...............A...........  240
Query_175893  121  ...................----.----.......................................-...........T..........-............................  230
Query_175894  180  ........A..C.......----.----...A..................-................-..........TT.....T....-A...............AT..........  288
Query_175895  120  ...........C.......----.----........--...............T..T...C......T...........A.....C....-A...............A.G....CA...  228
Query_175896  131  ........A..........----.----.......................................-........G..T...A.T....-A......T........A...........  240
Query_175897  91   ........A..........----.----.........T.............................-..........TT.....T....-A......T........A.........  198
Query_175898  220  ...........G.......----.----............C.......G.....CG..TGA......-......G....T.A...T....-A...............A.G...  323
Query_175899  121  ...................----.----.......................................-...........T..........-............................  230
Query_175900  177  ...................----.----..  200
Query_175901  130  .G.........C.......----G.-----........T.A.A.........................-..........TT.....T....-A...............A....C.CA...  238
Query_175902  134  .....T.....C....C.-----.G----.GCA.....T...AA............G.........G.-...........T.----.....-A...C..........  227
Query_175903  97   T..........G.......----.----...A.......................G...G..C....-..........CA.....T..C.-A...............AT....  200
Query_175904  81   ...........G.C.....----.----.....................G...TGG...........-.........G.T.....C....-..G.AG..........A.....  184
Query_175905  178  ...................----.----.......................................-...........T.-C..C....-............................  286
Query_175906  67   ...................----.----.......................................-..........TT.....T....-A...............A...C.......  176
Query_175909  98   ...........C.......----.C----.....................G.................-C..........T.....T...A-A...............A..  198
```

ECR3

```
Query         1    ATTTATTGAGAGCTGACTGTGTGCTG---CTGGTCTCGGATTTCTA--TTC------CACATAA-TCTGCCTCCAGAGCTGGAAAATTCCAGTGTCTGACCAT--T---CCTCAGCCTCC  103
Query_226932  73                                                                            ..............T..                                89
Query_226933  1    ...........A....T.........---.................--..------..T...-.....T...............................--.---...........  103
Query_226934  1    ..........................---.................--..------......-..................................--.---...........  103
Query_226935  26                                                  ..------....C.---.A....GG..............T..A.CC...C....--.---...T....AT.  82
Query_226936  1    ..........................---.................--..------......-..................................--.---...........  103
Query_226937  6                 ..............------.C..G.....TG.GT..A------....C..G..A..T..TG.................C....CTG..--.---......T....  95
Query_226938  2    .....C.....----.......A...ATT...C.T.TC....C.A.--ACA------....C.G-..A.....TG..........G.......CC.C.C...C--.---.....A.....  103
Query_226938  58               ............                                                                                           47
Query_226939  39   ..........................---.................--..------......-..................................--.---...........  141
Query_226940  1    ..........................---.................--..------......-......C...........................--.---...........  103
Query_226941  4        .......A............A.------.C..G.CT...TG.--...TG----....C.G-.T.......................CC....T.TT.CT.---G..........  103
Query_226942  1     ..........A.............------....G.TC...TG.--..------....C.G-.........C...............G..C...TC....--.GTT...........  102
Query_226943  1    ...........A..............A------.C..G.....TG.GT..A------....C..G.....T..AG.G..........G.....C....C.G..--.---......T....  103
Query_226944  3                ..............---......CA.A.....T--..------......-..C.................................--.---....G......  93
Query_226945  30   .........A.A......C.......GT-.......TTTGA....T--..GATTTCA....C.G-.........G.................C....C....--.---.......T...  140
Query_226946  19                       ....-..---.......G.T....T..--..------....C.----------...TCT.T.......T....C....CT..C--.---...T..G....  92
Query_226947  1    ......................C....---.................--..------......-..................................--.---...........  103
Query_226948  1    ...........A..............---.................--..------..T...-.....T...............................--.---...........  103
Query_226949  46                                                                   ...T...A..............T..A.CC...C....--.---.........T.  94
Query_226950  1                 .............T---......CAA........--..------......-..................................--.---...........  91
Query_226951  82                                                 ...------....C.T-.T....................T..A.C....C....--.---....G......  141

Query         104  CTCCCCTTCCCCACTCCAGCTGCAGTTTCCATGGTAACAAACTGAACCTGC-TTCTGTAGCTAACAAGAG-CTAAGAGTTTTTG-AGAGGGAATAAGCCAGAAC-AAC---TGACGTCAG  216
Query_226932  97       ....T..........................T.G.G........A-C.....G........C..-....C..C.C...-.A....TC...........-..T---..GA.A..A  205
Query_226933  104  ............................T....................-C...................-.....G.......-.A.................-..T---..........  216
Query_226934  104  .................................................-....................-..............-.A.................-...---..........  216
Query_226935  83   ..T...----.G.G......................T.G.AC.......A-.....--........C..-....C..C.C...-........C...A.......-...---..GA....A  189
Query_226936  104  .................................................-....................-..............-.A.................-...---..........  216
Query_226937  96   ....T...T........................C....G...........-..G.A.............-.......C.CC..-.A.AT...C..........-C.T---GA.TA...A  208
Query_226938  104  ....TT.............................G...........-C...A.............-.....C.C.C...-..G.....C..........G...CAT........T  220
Query_226939  142  .................................................-....................-..............-.A..A..............-..T---..........  254
Query_226940  104  .................................................-....................-..............-.A.................-...---..........  216
Query_226941  104  ......................................G...........-C...A.....C..C....-.......C.C...-.AG.....C........G.-C.T---........A  216
Query_226942  103  ......................................G...........-C...CC.A......G...-.......C.C...-.AG.....C.GA.......-C.T---..........  215
Query_226943  104  .................................C....C...........-C...A..............-.......C.CG..-.A.AT...-C....T....-C.T---........A  215
Query_226944  94   --..A.............................................-C...................-..............A.A...................-..T---........A  205
Query_226945  141  ....T........T.........................G...........A....A.............C.A.....CAC..-.-..---.A..........-C.T---....A...A  251
Query_226946  93   .-..G.................................G...........-C...A........T....-A.G....C.C...-.AG.....C.....G....-C..---........A  204
Query_226947  104  ............................T....................-....................-..............-.A.................-..T---..........  216
Query_226948  104  ............................T....................-C...................-.....G.......-.A.................-..T---..........  216
Query_226949  95   ..-----........................T.G.G........A-C....CG........C..-....C..C.C...-.A....GC...A.......-..T---..GA.A..A  202
Query_226950  92   .--.G.................T.........C....G...........-C..............................-..............-.A.................-..T---........A  202
Query_226951  142  .....T................AG..............G.....C.....-CA..CA............-....A..C..C..-.TG......C.T...A...-..  244

Query         217  CATACCCTGGCTGGGGGATGGGAAGGACTGCTTGAAGCCCTAAAGCCTTCTATTCCTCCA  276
Query_226932  206  .C..AAT...-.......A...-...G..C.C....T....  244
Query_226933  217  .C.......................................................T..  276
Query_226934  217  .G..........................................................  276
Query_226935  190  .C..AAT...-.......A...-..CT..CTC...........  230
Query_226936  217  .G..........................................................  276
Query_226937  209  .C..G..CA.............G....T...C....A..........-...G........  267
Query_226938  221  .C.-GG...A............G.......C............A......  270
Query_226939  255  .G..........................................................  314
Query_226940  217  .G.....................................G....................  276
Query_226941  217  .C..G.............G....  239
Query_226942  216  .C..G.................G.......C.................C..  266
Query_226943  216  .C..G...A.............G....T...C....A..........-C..G........  274
Query_226944  206  .C..G................TG.........G...........................  264
Query_226945  252  .C..A.....T.....................  283
Query_226946  205  .C..A............T..C.G...C..T.C...............  251
Query_226947  217  ............................................................  276
Query_226948  217  .C............................C..........................T..  276
Query_226949  203  .C..AATA..TG......A...-...G..C.C....T...  241
Query_226950  203  .C..G................TG...................................  261
```

ECR4

```
Query         1    TCGCCTCGGTGGACACGCTTCTGCAAGCCAAACAAT---TG------CTTTGCTTTGGATA----GCCGG-----------GCCCATCAAAGATGGACTCAGTCTGATAAACACGCCTCT  96
Query_44709   1    .......A............................---..------............G.----....A-----------.................G.......G.............  96
Query_44710   88                                                                                    ...T.....G..C.......C..................  126
Query_44710   13     .....A.A.A..G.....T...G...........                                                                                    46
Query_44711   36   .......A.CAA..C.....T...............CAG..TCAGCA............A.----.T.A.CTGGTGAAAGA.........G..........C..................  151
Query_44712   88                                                                                    ...T.....G..C.......C..................  126
Query_44712   13     .....A.C.A..GT....T..TG...........                                                                                    46
Query_44713   48                     ..T...............CAG..TCAGCA............A.----.T.A.CCACTGAAAGA.........G..........C..C...............  145
Query_44714   1    .......A............................---..------............G.----.....-----------.......................................  96
Query_44715   1                   .....T...............CAG..TCAGCA............A.CTCA..T..TGAAAGA----........GG..........C..................  101

Query         97   GCATGCCAACCAATCCAGCGGGGTCTCACTCCAGAGACCACACTTCTGCAGATG  150
Query_44709   97   ..................A...................................  150
Query_44710   127  .GG............A.CAT..........TG..........G.C..A....    178
Query_44711   152  .AG...........TA.CAA..........TG..........G....A......  205
Query_44712   127  .GG............A.CAT..........TG..G.......G.C..A....    178
Query_44713   146  AAG............A.CA...........TG....G.....G....A.C....  199
Query_44714   97   ......................................................  150
Query_44715   102  .AG.....G........CAAA..C......TG.....A....G....A......  155
```

ECR5

```
Query         60    AAATGAGCCTTCTTCCTGGCCCCTGC-G-GG-GCCTCCAGCC-G-G-C-AGCTGGAGGGATTAAGGAAAAGAGGTTCCTGGCGTCCCTGAGTCTGGAGTGGCTGCCCAGG--A-TG-TCC  168
Query_248859  51         .....C...........T...-AA..-..........T.-.-.-...C..............C......CC..........G.........C.............--.-GCC...  157
Query_248860  100         ..................C.-A-A.A..........-C---.-...C..............C.G....CG....................T.CA......TG..--.G..-...  203
Query_248861  78         .....C.......AG..T...-AA..-..........C.-.-.-...CA...A.........C......C.....................T.....A.......--.-GCC...  184
Query_248862  101        .....C...........T...-AA..-....G.....T.-.-.-...CA.............C......CC....................C.............--.GCC-...  207
Query_248863  164                                 ..-.........T-.---.-...AA.............G.....................T..-----.......G.AG..--.-..-.-T  238
Query_248863  177                                                 .-.-.........  167
Query_248864  101                                 ..-..........-C---.-......G.............G....C.............C.......T.....A...TG..--.G..-...  182
Query_248865  50         .....C...........T...-AA..-....G.....T.-.-.-...CA.............C......CC....................C.............--.GCC-...  156
Query_248866  60    ........TG............T.ATT.-..-..........-T-.-........................C---................................G..--.-.A-...  166
Query_248867  101                                   ..........-C---.-...C................GA...C.............C.......T.........TG..--.GC.-...  180
Query_248868  51         .....C...........T...-AA..-..........-CA.-.-...CA.........T...C......CC............C.......CA........TG..--.-GCC...  157
Query_248869  50         .....C...........T...-AA..-..........T.-.-.-...CA.............C......C.....................C.............--.GCC-...  156
Query_248870  78         ...T.......C...TTT...-AA..-..........-C---.-..................G.G....C.............C.................TG..--.G..-...  182
Query_248871  1                              .-.-.-.......T...-.-.-.-.......A.........C.......C......A.....CA.......CTCT......AGC.--.-CC-...  84
Query_248872  114                                              .-...CA.............C....C.C.....................CC............--.-GCC...  182
Query_248872  97           ...C............  112
Query_248873  79          ....C......T....T..A-.A..-..........-C---.-.................G..G....C.............C.......-.........TG..--.GCA-...  181
Query_248874  76               .....AA....T....-.A..-.....TC...-C---.-.................CC.G....C...........G.C.......G.........TG..--.GC.-...  174
Query_248875  80           .......T.A...T.T...-AA..-..........-C-.A.-...CA.............T......C.....................T.G.......TG..--.G..-...  184
Query_248876  91         .....C...T.......T...-AA..-..........C.-.-.-...CA.............C......CC....................C.............--.-GCC...  197
Query_248877  79         .......C...CCC....A.A-A-..-..........-C---.-...............A..G.G.CCCCC............C.......-.CC.......T..GAG-..-...  182
Query_248878  7          ..T....C.C.A.....T...-AA..-..........-C-.-.C...CA....-.......G...A...C..T..................T.........GG.C--C-C.-...  111
Query_248879  78        ...T....C.....A...T...A.-..-..........T.-.-..C..CA............G......--............A........T.........GG.A--.-..-...  182
Query_248879  219                              .-..-..........  207
Query_248880  59    ......................T...T-.-........C..T-.-.-...........................C...................................--.-..-...  168
Query_248881  96           ...C.......AG..T...-AA..-..........C.-.-.-...CA.............C......C.....................T.....A.......--.GCC-...  200
Query_248882  39     ..C...T..G.C.........CA.-CA.-............-.-.-.-.......----......CC......C...........T...................TG..--.-..-...  143
Query_248883  91                                        ..-C-.-.-...CA.............C....C.C.....................CC............--.-GCC...  163
Query_248883  76         .....C............  93
Query_248884  78         .......C...CA....T...-AA..-........TG-.-.-------....................AC....................CT.........TG..--.G..-...  178
Query_248885  78         .......C...CA....T...-AA..-........TG-.-.-------....................AC....................CT.........TG..--.G..-...  178

Query         169   CAGGAG------AG-GC-G---------AGTG--GGCCAGAGGGCTGGAGGCTCCCAAATCAATCA-GAGGTCTGAAGGTCACT-G-AATGGCGACCTTTGAATCAGGATCCC-GGT-AA  264
Query_248859  158   ....G.------..-T.A.---------C...--.......................G........-.................G.-.GCT.T......CA...T.....A.T-T..-..  255
Query_248860  204   .G....------..-A.-.---------GAG.--.......................G.GG....G-.G.AC.........GT.A.-.GCC.T.......A...T...TCA..-C..-..  300
Query_248860  134                                            .............  122
Query_248861  185   ......------..-CT-.G--------...A--................................-.................G.-.GCT.T......CA...T.A...A.T-T..-..  282
Query_248861  113                                            ...........  103
Query_248862  208   ....G.------..-C.A.---------....--.......................G...G....-.................G.-.GCT.T......CA...T.....A.T-T..-..  305
Query_248863  239   ......GGTCTC..-.G-.---------.A.T--A.--..T..A......A......G........-.....G..........  305
Query_248864  183   ......------..AAT-.---------....--.....................-.G.......C-A..AC..........T.A.-.GCT.T.G....CA...G.A.T.A.T-T..-..  279
Query_248864  113                                            ...........  103
Query_248865  157   ....G.------..-C.A.---------....--.......................G...G....-.................G.-.GCT.T......CA...T.....A.T-T..-..  254
Query_248866  167   ......------..-..-C---------.C..--................................-......----------.-.-.....T..................TT-...-..  252
Query_248867  181   ......------.C-A.T.---------..CA--.C..........T....-..............-.C.AC..........T.A.-.GCTCT.G....CA...T...T.A.T-T..-..  277
Query_248867  111                                            .............  99
Query_248868  158   ....G.------..-C.A.---------....--.......................G........-.................G.-.GCT.T......CA...T.....ATT-T..-..  255
Query_248868  86                                             ...........  76
Query_248868  157                                                 ...........  147
Query_248869  157   ....G.------..-C.A.---------....--.......................G........-.................G.-.GCT.T......CA...T.....A.T-T..-..  254
Query_248870  183   ......------..-A.T.---------..C.--...........................CG..G-..T.C........  243
Query_248870  113                                            ...........  103
Query_248871  85    ....G.------..-TT-.----------...CT.........C.CC...................G.G..........G.GTG-.-GGCT.T..........CAG.......T..G-.C  183
Query_248872  183   ....G.------..-C.A.---------....--................................-.G...............-.  248
Query_248873  182   ......------..-A.T.---------....--....GC.....................C...G-...AC..........T.A.-.GCT.T.G....CA...T...T.A.T-T..-..  279
Query_248873  113                                            ...........  103
Query_248874  175   .G....------..-A.G.---------C.C.--............................T..T-...AC.C........T.A.-.GCT.T.G...CCA...T...T.G..-T..-C.  272
Query_248875  185   ....G.------..CTG-.---------CA..--...TG...T.......................-................  248
Query_248875  113                                            ...........  103
Query_248876  198   ....G.------..-C.A.---------....--.......................G........-.................G.-.GCT.T..T...C....T.....A.T-T..-..  295
Query_248876  126                                            ...........  116
Query_248877  183   .G....------..-A.-.GGGCCGGAA..AA--..G..A..........C.C....  229
Query_248877  113                                            ...........  103
Query_248878  112   ...--------------------------------------------.....C.............-.........G....TT.-.T.GCT.T......CA...T....C..T-T..G.C  186
Query_248878  42                                             ...........  32
Query_248879  183   ....G.------..-CT-.G--------....--..................C.............-.G.......  239
Query_248880  169   ......------..-..-.---------....--................................-.................-.-.........................G-...-..  264
Query_248881  201   ......------..-C.G.---------...A--................................-..............G..A.-.GCT.T......CA...T.A...A.T-T..-..  298
Query_248881  129                                            ...........  119
Query_248882  144   ....G.------.A-CT-.---------..G.--................................-.................-.-.GCC.T......C............T-T..-..  239
Query_248883  164   ....G.------..-C.A.---------....--.......................G........-.................G.-.GCT.T......C....T.....A.T-T..-..  261
Query_248884  179   ...AG.------..-CT-.G--------..A--..........C......................-A................-.  244
Query_248885  179   ...AG.------..-CT-.G--------..A--..........C......................-A................-.  244
```

ECR6

```
Query         15   CATCGTAGCTATTCTAAGGAAGGCCTGCTGGGGTATTCAGATGGTGGGATCCTCCTAT---------TCAGCCCCATCGCTGTGTTTTCCCTCTTCTCTCTGATATTTTTGGTGGCAC--  123
Query_242636  86                                                                                   ....................ACT...G.---C.A....AG  122
Query_242636  51                                                                                   ....................                      70
Query_242637  86                                                                                   ....................ACT...G.---C.A....AG  122
Query_242637  51                                                                                   ....................                      70
Query_242638  1    .............A..................-...........C.A.C.A.......CGGCTCAAT....A...G..ATC...................C.................--  117
Query_242639  101                                                                                  ....................AC....G.---C.A....AG  137

Query         124  -GGCAAATATTTTCTGCCTGCCTCTTTCTATGAAACACTCAGCAAAAGTGGGAGGTGAGCCACAGCTTGTACTTATTTG-GGCTTTCCAAACGCGTGGCACGGTTCTGTGTGCTTTGC  239
Query_242636  123  TA...........-.......T.........TC..T.TG.......                                                                        167
Query_242637  123  TA...........-.......T.........TC..T.TG.......                                                                        167
Query_242638  118  -..........................................G...........C...TA.................A-.........G............C.CT..C..A....    231
Query_242639  138  TA...........-.......T........GTC..T.T........----T....CT.AT.TT....CACT........A.......G....AT.A.A...AA..T.....AT.....  250
```

ECR7

```
Query           1    GGCTCCAGGAACTGGCTGTCAGGGAGG-AGAGG-G-T-GTAGCTGTCCT-G-GGG-TGATGGAATGTAGCCAGCAGCTGTCTCTTCTCTATTTGAGCCTGTACACTGGCAGGGCACTGTC  113
Query_226890    1    .............A.............-.....-.-GC...........-.-...-..........C.................C...C...............................  114
Query_226891    101                                 ..-.-.-.C.........C.-...-.......GA.C.................CTA.C.C.....T...AGGG.......C.TG.....  184
Query_226892    101                                 ..-.-.-..........C-CA...-..........C.................CTA.C.C.....T...CCTGT......C.TG.....  184
Query_226893    101                                 ..-.-.-.C.........C.-...-........A.C.................CTA.C.C.....T...                     164
Query_226894    101                     .......-GA...A.-C-...........-T-A.CA..............T.............CTG.C.C.C...T..A...TT..A...AATG...C.  195
Query_226895    101                     .......C.A...-.-.-..........C-CA...-.........AC.................CTG.C.C.....T...--..T.......ATG.....  193
Query_226896    1    ..........G................-.....-.G.-...........-.-...-................................C...........................C...  114
Query_226897    86                             ...-A-.-...A....TT.-.-...-.......C...CA...............CTG.C.C.....T...C.TGT..A...C.A......  169

Query           114  ACGCAAAGCTTCCCCATAAGAGCATGGGGATAGATGATCACTCTCAGGTCATTTATCTGTGTGAGGAAGGCCAGCTTCCTTTGCACCTAGAAGAATAGCCTTAGCTGGCCTGCTGTGG  231
Query_226890    115  ...A.............                                                                                                     131
Query_226891    185  .G.T..G..C.......G..                                                                                                  204
Query_226892    185  ......G..........G..                                                                                                  204
Query_226894    196  ..A........T.....G..                                                                                                  215
Query_226895    194  ..A..C.....T........                                                                                                  213
Query_226896    115  ................A......------....TG..C..............C.G.G........A.......A..........G............C.....C.........C....  226
Query_226897    170  ......G..........G..                                                                                                  189
```

ECR8

```
Query        1    CCTGGAATCCACAAGAGGAAGGGGTTAAGCCTTCT-AGCCCATTTGTCGCTGGAAAC-T-CAGTTTGAATCCCACCC-TGGAATTTTCC-TCCGAGCTACGAAGCCAGGCGAGCTGCCAA  115
Query_29669  75                                          ....G....-..A...C.-.-...C...C.........-...........-..T...T.G.A..AT.....A.........  150
Query_29670  43           ..C.....A...........A....TC-............CT.....C.-.-...CA..C.........C........C..C..T.......A..........CG.......  151
Query_29671  53          ...C.G.....C.........A..----------..G....-..A...C.-.-...C...C.........-...........-..T...T.G.A..AT.....A.........  150
Query_29672  75                                          ....G....-..A...C.-.-...C...C.........-...........-..T...T.G.A..AT.....A.........  150
Query_29673  48              .....A....A..C.........C-.....C.C....-....G...-.T...C.-.C.........-...........-..T.......AG........TG........  150
Query_29674  3                     ...........A.....C-G...........CT.....C.-.-...C...C.........-...........-..T.......A.........C.........  100
Query_29675  75                                          ....G....-..A...C.-.-...C...C.........-...........-..T...T.G.A..AT.....A.........  150
Query_29676  1    ...A.........G..T.................C-.....................-.-.................-...........-..T...........................  115
Query_29677  1                     ...........A...TA-G...........CT.....C.-.-T..C...T.........-.....C.....-..T.......A.........AC........  98
Query_29678  49                ...A...........A..A.GC-..G.T.......CT..A..C.-.-T.C...C.........-...........-..AT.C....A.........AGC.......  150
Query_29679  51                    ...........A....TC-G..........CT.....C.-.-...C...C.........-.....C.....-..T.....C.A.........AC........  148
Query_29680  84                                                    ..A...C.-.-...C...C.........-...........-..T...T.G.A..AT.....A.........  150
Query_29681  75                                          ....G....-..A...C.-.-...C...C.........-...........-..TA..T.G.A..AT.....A.........  150
Query_29682  98                                                                   ..C.........-...........-..G.......A..A..C...C.........  150
Query_29683  51                .......A.......AA...TC-...........T-...G.C.-.-...C...T.........-...........-..T.......------....C.........  145
Query_29684  53                    ...........A.T..GC-..T.T...C...CT..A..C.-.-T.C..TC.........-...........-..GT.....A..........G........  150
Query_29685  84                                                    ..A...C.-.-...C...C.........-...........-...A..T.G.A..AT.....A.........  150
Query_29686  1                     ...........A.TG.T.-...........CT.....CA-.-...C...C.........-........T.-..T.....G.A.........C.........  98
Query_29687  12                    ...........A.....C-.T...G......C.G..G.C.-.-.C.C.C.C........G-......G...-..G.......A.....C...C.........  109
Query_29688  49                ...A..G........A.....C-.....C.C....CT.....CT-.-...C...C.........-...........-..T.C...CGA.........C.........  150
Query_29689  75                                          ....G....-..A...C.-.-...C...C.........-...........-..T...T.G.A..AT.....A.........  150
Query_29689  14                                                                                                          ...........       4
Query_29690  51                    ...........T...TC-G..........CT.....C.-.-...C...C.........-.....C.....-..T.......A.........AC........  148
Query_29691  56                       ........A....T.-G.G.........A......CT-.-...CC..T.........-........C.-..G.......A...TG...C.........  150
Query_29692  56                      .........A...G-.-G.A.........A......CT-.-...C...C.........-...........-..T.......A...TG...C.A.......  150
Query_29693  1    ...................................-............C........-.-.................-...........-..............................  115
Query_29694  53          ...C.G.....C.........A..----------..G....-..A...C.-.-...C...C.........-...........-..T...T.G.A..AT.....A.........  150
Query_29695  49                ...A....A......AG...GC-..GTT.......CT..A..C.-.-T.C...C.........-...........-...T.C....A.........TGAT......  150
Query_29696  47                ...A...............TC-.....G......-.........C-...C...C.........-...........-..T.......A.........C.........  148
Query_29697  47                ...A............-.G..C..T...C...A..A...C.-.-T.CC.........-...........-..T...G.G.-.........C.........  147
Query_29698  84                                                    ..A...C.-.-...C...C.........-...........-...A..T.G.A..AT.....A.A.......  150
Query_29699  64                                        ........C......C.-.-...C...C.........-......C....-..T.....G.A.........C.........  139
Query_29700  43                ...A.........C.T..GG-..A...C...AG..ACCC.-.-.---.A..TG.GTT..T-..........-.......C..AG.........G.TC.....  141
Query_29701  52                 ..A..........A.T...--....-........T.....C.-.-G.C..CC.......T.-...........-CTT...............C.........  150

Query        116  GTC----ATATATACA----TTGCCGGGTAGCCAGGCAACTCCG---------TG--CTTTTTCTGGGAAGGAACACAATGTGGTCACTG-TTTCTG-C--A-TCGCCAGGTTCCAGATG  211
Query_29669  151  .C.----.........----....................A...---------.--................G................--.G...C.--G-A...............  246
Query_29670  152  ...----CG.......----..............A.....G.G.---------.--..C.............CT...............--.G...T.--G-A...............  247
Query_29671  151  .C.----.........----....................A.T.---------.--................G................--.G...C.--G-A.A.............  246
Query_29672  151  .C.----.........----....................A...---------.--................G................--.G...C.--.-A...............  246
Query_29673  151  ...----.C.......----...T.T..............---.---------..GA...............G..T...............-....CC-A--G-A.A.............  245
Query_29674  101  .C.----.........----...A................G.G.---------C.--..C.............CT................-...-..-T--.GA..............  196
Query_29675  151  .C.----.........----....................A...---------.--................G................--.G...C.--.-A...............  246
Query_29676  116  .C.----.........----........................---------.--.................................-.......-.--.G...............  212
Query_29677  99   ...----.........----...T................GTG.---------C.--...............CT................-...-..-T--.GAA.............  194
Query_29678  151  ...----.........----...T........T.......A.T.---------.--..C..........A.CT................-...-..-.--.GA.A.............  246
Query_29679  149  ...----C........----....-...............A.G.---------A.--...............CT................-...-..-T--.GA..............  243
Query_29680  151  .C.----.........----...T................A...---------.--................G................--.G...C.--G-A...............  246
Query_29681  151  .C.----.........----....................A...---------.--................G................--.G...C.--G-A...............  246
Query_29682  151  ...----.......T---.........CA.........A...AGCTTCTCC.--...C..........CTG.....C..........--.G...-.G-G-................  256
Query_29683  146  ...----.........----...T.........A....C.A.---------.--...............CT......C.......T...G..-G--.--...............  241
Query_29684  151  ...----.........----...T................GTT.---------.--..C.............CT................-...-..-.--.GA.A.............  246
Query_29685  151  .C.----.........----........T...........G...---------.--................G................--.G...-.--.GA..............  246
Query_29686  99   ...----.........----..A.T...............G.A.---------.A--...............CT...G.....G...-...-..-T--.GA.....A.........  194
Query_29687  110  ...GTGT.........TACG.....TC..C..........G.G.---------C.--..C...........ACG................-..C.G.-A--G-A.T.............  213
Query_29688  151  ...----....--.----...T.................G...---------.--................G................-...-..-.--.GA..............  244
Query_29688  407                                                                                                                ....     411
Query_29689  151  .C.----.........----....................A.T.---------.--..............A.G................--.G...C.--G-A...............  246
Query_29690  149  ...----.........----..........A.........G.A.---------C.--...............CT................-...-..-T--.GA..............  244
Query_29691  151  ...----.........----...T.A..............G.T.---------.--................G................-...-..-.--.GA.A.............  246
Query_29692  151  A..----.........----...T................G.T.---------.--................G................-...-..-.--.GA.......C.......  246
Query_29693  116  ...----.........----.....................T..---------.--.................................-...T.-.--.G..A.............  212
Query_29694  151  .C.----.........----....................A.T.---------.--................G................--.G...C.--G-A.A.............  246
Query_29695  151  A..----C........----...T................GAT.---------.--..C.............CT................-.G.-.-.--.GA.A..G..........  246
Query_29696  149  ...----.C.......----...T................G...---------.--..GC...........ACG................--.....-.--.GA..............  244
Query_29697  148  ...----.........----....................G...---------.--.C.G......C...G................--.....-.--.GA..............  243
Query_29698  151  .C.----.........----...T....T...........G...---------.--................G..T.............--.G...-.--.GA..............  246
Query_29699  140  ...----.........----...-.T..............G...---------.--................G................--.G...-.G-G-A.A.............  234
Query_29700  142  AC.----.........----.G..T...............G.G.---------.--..C.....A...T.CT.........AC.....-..C.C-A--G-A.CT.G...........  237
Query_29701  151  ...----.........----...T..-.........G..G.T.---------C.--.......C.......G.................-...-..-.CG.-.-...............  245

Query        212  -G-GAGAAAGACTTTAATCCCCAGCTTCAAGGCTGTTTGGCAAAACTCACT-CTGGAGCCCCTGCCTGGGCCGGTGTTCGTTTGCTG-TGACCCTCCGGTGCTGCCA-T-TTTGTGACAT  325
Query_29669  247  -.-A.......................C...........C.CT..G..-.CT..AAG.....TC.....A..T...----A..C-....T....A...AG....-.-......CT...  356
Query_29670  248  -.-A.......................C...........C.TT..G.C-.CT...AT.GCA.TCT.....--A.....--.CA.CC-C...T....A.C..G....-.-......C...G  357
Query_29671  247  -.-A..................G.....C...........C.TT..G..-.CT..AAG..G..T......A..T...----A..C-....T....A...AG....-.-......CT...  356
Query_29672  247  -.-A.......................C...........C.CT..G..-.CT..AAG.....TC.....A..T...----A..C-....T....A...AG....-.-......CT...  356
Query_29673  246  -.-AG.....................C.TGC.......G....C.TT.....-.CT...AT.....TCT....-----A.T.CCA..C-.A..T.GA.A....TG....-.-.....CC....  354
Query_29674  197  -.-A...................................C.CT.....-.CT...AT..C..GC.....A..T...----A..C-....T....A.C.TG....T.-......C....  307
Query_29675  247  -.-A.......................C...........C.CT..G..-.CT..AAG.....TC.....A..T...----A..C-....T....A...AG....-.-......CT...  356
Query_29676  213  -.-.............................................-...A.......................T........-..........GT.G.......T.-.....G..TG.  327
Query_29677  195  -.-A.....................GC............C.TT.....-.CT...AT.....T......A..T...----A..C-....T..T.A....TG....-.-......T....  304
Query_29678  247  -.-A.................A...TGC..T.........C.T...G..-.CC...-G.....TCT....A..----T......C-...TT..G.A....A....-.C......TG...  356
Query_29679  244  -.-A.................CT...GC...........C.TT.....-TCT...AT.....TC.....A..T...A.----.C-....T....A.C.TG....-.-....A.CC...  353
Query_29680  247  -.-A.......................C...........C.CT..G..-.CT..AAG.....T......A..T...----A..C-....T....A...AG....-.-......CT...  356
Query_29681  247  -.-A.......................C...........C.CT.....-.CT..AAG.....TC.....A..T...----A..C-....T....A...AG....-.-......CT...  356
Query_29682  257  -.                                                                                                                      257
Query_29683  242  -.-A.....................GC............C.TT.....-.CT...AT..A..T.T.....--A.....--.CA.CC-...TT....A..A.G....-.-......C..T.  351
Query_29684  247  -.-A..................---..CT...........C.T...G..-.CT...-G....TTCT....A..T...----A..C-...TT..A.A.C..A....-.-......TG...  352
Query_29685  247  -.-A...G.....C........G.T....C...........CGTT..G..-.CT..AAT.T...TGA....A..T...----A..C-....T...TA...AG....-.-......CT...  356
Query_29686  195  -.-A.....................GG............C.TT...T.-.CT...AT.....GG.....AA.----T...A..C-....T....A....A....-.-......C....  304
Query_29687  214  -.AA...G..............G.....GC............CGTT..T..-.C....ATG.....G.....                                                   283
Query_29688  245  -.-A.....................C.GC...........C.TTC.....-.CT..-----AAT...T.....CC.A.TC.CA.GC-..G.T...--...TG....-.-C.CA.C....  351
Query_29688  412  -.-.....                                                                                                                417
Query_29689  247  -.-A.......................C...........C.CT..G..-.CT..AAG.....T......A..T...----A..C-....T....A...AG....-.-......CT...  356
Query_29690  245  -.-A.....................GC............C.TT.....-.CC...AT.....TC.....A..T...----....C-....T...GA....TG....-.-......CC...  354
Query_29691  247  -C-A..C.....G........T....C..C...CCAC.....C.T.......-.CT..AAT......G.....A..T...----A..C-CC.......A.C..G....-.-......C.G..  356
Query_29692  247  -.-A.................T....CGGC...........C.TT.......-.CT..AAT.....TGT....A..T...----A..C-CC..T....A....G....-.-......C.G..  356
Query_29693  213  -.-.............................................-T.........................A.......-.........................-.-.......  326
Query_29694  247  -.-A..................G.....C...........C.TT..G..-.CT..AAG..G..T......A..T...----A..C-....T....A...AG....-.-......CT...  356
Query_29695  247  -.-A.................T.....TGT...........C.T.A....                                                                        295
Query_29696  245  -C-A.C...........G....G.....GT...........TC.T.......-.CT...AG.....T.C....TC.C...--.CT...A.T....AG.-...--....-.-......C....  354
Query_29697  244  -.-A...G.........-.........GC.....C.....GC.CG.....-.                                                                      292
Query_29698  247  -.-A...G.C...C........G.T....C...........CGTT..G..-.CT..AAT.T...T.A.......T...----A..C-....T...TA...AG....-.-......CT...  356
Query_29699  235  -.-A............-.........GC............C.TT.....C.CT..AA......T......A..C...----....-.....TG...A...TG....-.-C...C....  344
Query_29700  238  C.-...C..................C.GGCA...........TCT.......-.CT...A...-.....                                                       302
Query_29701  246  -.-A..................T...G.............CGTT......-TCT...AT.....T.T....A..T...----A.CC-...TT....A...TGA....-.-......CT...  355
```

ECR9

```
Query        1    CTGGCTACACTGAATCCCACAGCTTTTGCTGATAGTTTTAAACCAAGGACAAACAGCACAACCAAGAC-A-AGA-TCCTATGTGTGCACGTGTG-TG--CAG--GCAGCTGCATCCAAGG  112
Query_20777  3                   .........CC.....G...C....C..T...A..G.-.T..GGTCT---.C.-.-...-..--............CC-.C--.G.--................  93
Query_20778  1                   ........CC.-...............T...A....GT----...GACT.C.-.-...-..A.GC.CACA.......A-CC--...--........G.......  92
Query_20778  75                                                                                  ..G..A........-..--.                      59
Query_20779  3                   .........CC.....G...C....C..T...A..G.-.T..GGTCT---.C.-.-...-..--............CC-.C--.G.--................  93
Query_20780  3      ........A.........................................G.......-CG...A.....C............A-.C--...--................  114
Query_20781  26                                                                                       .......AA.C--...--.......TG.......  55
Query_20781  31                                                                                       ........-..--..                   20
Query_20782  1                   ........CC.-...............T...A....C.----...GTCT.C.-.-...-..A....CACA..T....A-CC--.G.--..G....A........  92
Query_20783  29                           .......G..........T...A....T--.T..GTTG--.C.-.-...-..A.G....CT.C.A..CA-.C--..A--...A....C.......  112
Query_20784  85                                                                    .-.-...-..A.G..CACA.......A-.C--...--........G.......  129
Query_20784  35               ..G........CC.-...........A....T...                                                                     68
Query_20784  106                                                                                      ........-..--..                   95
Query_20784  66                                  ...........                                                                          56
Query_20785  3                   .........CC.....G...C....C..T...A..G.-.T..GGTCT---.C.-.-...-..--............CC-.C--.G.--................  93
Query_20786  12   ..T.......CA..GT.......CCCC....GC..........T...A....C.G----..GGCT.C.-.-...-..--.....CA...A....-.ATC...--........G.......  119
Query_20787  84                                                                        ...-..A.G...AC...T.....-.C--...--................  126
Query_20788  5                      .......CC.....GC..........T...----...TC.TG..GTCT.C.-.-...-....G..CA....T.....-.C--...--................  95
Query_20789  21                              .................T...A..G.----.T.C.GTCT.C.-.-...-..--.....CA....C...-.A--T..AG........G.......  102
Query_20790  75                                                                    .-.-...-..GC.....CA...A...A-AC--...--................  119
Query_20791  93                                                                                             ..-..--..T--A..CG..T........  115
Query_20792  34      .......CA..G......A..CC.....GCGT....TC......A....----.TG..GTCT.C.-.-..G-..A.G..CA.........A-.C--...--................  138
Query_20793  1    ..........................A................................G.......C.-...-..................-..--...--................  113
Query_20794  26                                                                                       .......AA.C--...--.......TG.......  55
Query_20794  31                                                                                       ........-..--..                   20
Query_20795  12   ..T.......CAC.G........CCC.....GC......GC..T...A....C..--..GG.T--.C.-.-...-..--.....C....A....-.ATC...--................  119

Query        113  ACATGATTAGCCGTTAAGCCCCCTCTGGATCCATGCATC-C-CTTGTTCAGCACATTCTCTTCCCAGCAAAAAGGCT-GGAGCCCCAACGGTGTTGTGTAATTCCCACCACACCCCAAG  228
Query_20777  94   ..G......A.T.C........-.......T......T-.-....G..........T.........T...G.....-AC..A....G..A....A......C..............  206
Query_20778  93   .........A.T.C........-........G.G.....-G-.C..G.................G.T...GC....-...AG...G.T.A.....AAG...C.T.......  200
Query_20779  94   ..G......A.T.C........-.......T......T-.-....G..........T.........T...G.....-AC..A....G..A....A......C..............  206
Query_20780  115  ..........T......................G.-.-...............C...........G.....-....G..................................  230
Query_20781  56   T........A.T.C........-.......TG...G.T-.A..C.G..................G.G...GC....-....G....GT.....GA.....C..T............  169
Query_20782  93   .........A.T.C......A.-........A.......-G-....G....................T...GC....-...AG....GT.A....A.A......T.......  200
Query_20783  113  .........A.T.C.........--.....T.......-A-..A.GC..................AG..G....A-A...G.....  195
Query_20784  130  .........A.T..........-........A.......-G-..CAG...................T...GCA...-...AG.....T.A.A..A.A......T...........  241
Query_20785  94   ..G......A.T.C........-.......T......T-.-....G..........T.........T...G.....-AC..A....G..A....A......C..............  206
Query_20786  120  ..G......A.T.C........-.A.....T......T-A-....G....................G...GC....-A..AG..T..T.A....A......C.T............  232
Query_20787  127  ....A.C..A.A.C........-.......T......T-A-....G..................AT...GT....CA...-....GA.AG...T..C...C.T............  239
Query_20788  96   .........A.T.C.........--.....T......T-.-....G.......................G.....-AC..G.....T.A....A..C...CG.............  207
Query_20789  103  ...........T.C.......-........G.....CT-G-...TG....................T...GC....-...AG.....T.A....A.A......T......G.....  215
Query_20790  120  ...C.....A.T.C......T.--...C.A.TGA.....T.-..C.G..................G.G...GT....-C..AG..GGGT....C.A..C.G.C.T...........  231
Query_20791  116  ...........T.C.........--.....TG.....T-.-....G.......................TGG.....-...AGT....T.A....A......C..............  227
Query_20792  139  ...........T.C.........--.....T......T-.-....G........................GG.....-A..AGT....T.A....A......C.............  249
Query_20793  114  .......C......................G.......-.-.............................G.....-.......................................  229
Query_20794  56   T..........T.C........-.......TG...G.T-.A..C.G..................G.G...GC....-....G....GT.....GA.....C..T............  169
Query_20795  120  .........A.T.C.........--.....T......T-A-....G....................G...GCA...-A..AG..T..T.A....A..A...C.T...........  230
```

ECR10

```
Query        116  TTT-CCAGAGACACACTCAAGCTCATCAACCCACCATGGGAttttttt----CCATC-AGG-CAGAAAAGCC-AGG-T-T-G-A-TTCCAAGAAATATATACATTGCCGCACAATTAGGC  222
Query_69995  118  ...-......G..AGT....-..........T............CC.----.T.----..-.T.....G.-...-.-.T.-.-......A..............A.TGT.....A..  221
Query_69996  124  ...-......G..A.T..--.T......G..T..T..........C------..C.G--..-.T.....C..-...-.-.G.-A....................A.TGT........  226
Query_69997  114  ...-...A..G..A.....-........G..T..TG.........C------..C.--..-.T.........-..-.-.T.-.-......T...C..........A.TGT........  217
Query_69998  124  ...-...A.CT..A.-..C..........TT......A......C------..C.--...-.T.....T..-...-.C.-.-....................A.A.TGTG....TT.  227
Query_69999  124  ...-......G....-..G-........G..TG..G.......C.C------..TC.-...-.T.C..G...-...-.-.T.-.-G.................C..A...T.....A..  227
Query_70000  116  ...-..............G..........................TTTC.A.C.-..-........-...-.-.-.-.......................T..........  226
Query_70001  75   ...-......G..A.-...-.........T..............-----..T..T...-.T.C..G...-...-.-.-.-.-...............T..C.TG.TGT....TA..  179
Query_70002  111  ...-......G..AGT...-.T.........T.T...........CC.----...----..-.T..........-...-.-.-.G.-.......................GT.....A..  214
Query_70003  33   A..-.T....G..A.T...C-..........T.........C...C------..C.--...-GT.......A-...-A-C-C-.A....G.....C..........A.TGT........  136
Query_70004  124  ...-......G..G.-...-......G.G..T..................------..C..-T..G.T.C..G...-...-.-.T..-..................C.TG.TGT.....A..  228
Query_70005  103  ...-..GA..G..A.....-........G..T..TG.........C------..C.--..-.T.........-..-.-.-T-.A.......T...C..G........A.TGT........  206
Query_70006  103  ...-..GA..G..A.....-.....G...G..T..TG.........C------..C.--..-.T.........-..-.-.T.-.-......T...C..........A.TGT........  206
Query_70007  110  ...-......G..A.T...-.........................CC.----.T.----..-.T.........-..-.-.T.-.-...................A.TGT.....A..  213
Query_70008  93   C..-.T....G..A.TG..-...........T.......T.C...-------..GCT-...-.CA.......-...C.-.-.-.-.......T...C..........A.TGT.G......  196
Query_70009  73   ...T...A..G..A.....-........G..T...G..A.......CC----..T---..-.T.........T.-.-.-...................CA.A.TG.CG.....  178
Query_70010  106  ...-......G..A.T...-.........T...C.....A....CC.----.TG----..-.T.........-..-.-.T.-.-...................A.TGTG....A..  209
Query_70011  120  ...-......G..AGT...-...........T.T...........CC.----...----..-.T..........-...-.-.-.G.-.....................A.GT.....A..  223
Query_70012  88   ...-...A..G..A.T...-........G..T......A..C...C------..C.T-...-.T........C..T-.-.-.-.-G.........CG..........G.TGTG........  192
Query_70013  106  ...-......G..A.-...-...........T..T...........------..C..T...-.T.C..G...-...-.-.T.-.-..................C.TG.TGT.....A..  210
Query_70014  46   G..-......G.CA.G...-..G.G..C...T..TC......CC.-------..CG.-GA.-.T.C..........-..-.-.T.-.-G....................ATGT........  149
Query_70015  8                                                    ..G..----..C.G-..A-.T......T.-...-.-.T.-.-G....GA..G.C.G......ATA.G...G..G....  73
Query_70016  156  ...-...A..G....G..G.-........G..TG..........G..------..C.T-...-.T.........-...-.-.-.-.-.......A.....C.........TA.TG.........  259
Query_70017  116  ...-.........T..............................----...CT-..-.........-...-.-.-.-.-..................................  222
Query_70018  54   ...-..CA..G..A.AC--.........GT.T..TG......C..C------..C.--..-.T.........-.A..-.-.T.-.-.......C..............A.TGT........  156
Query_70019  124  ...-......G..A.T.T..-...........T.........C...C------..G.--...-.C-..........-...C.-.-.-.-.........G.C...........A.TGT.....T..  226
Query_70020  124  ...-......G..-.G.TC.........G..T...........---...C----..TC.-...-.T.G..C.-...-...-.G.-.-...................A.TGT.....A..  227
Query_70021  120  ...-......G..A.T..-..T..............G.....---..G..----..CCT-G..-.TC.C.G...-...C.-G-.-G-A....G...T..C......CA.T.TG..G......  223
Query_70022  124  ...-...A..G.GA.T..G-.T.......................C------..C.--..-.T.........-..-.-.T.-.-...................A.TGT........  227
Query_70023  46   ...T...A..G..A.....-.........G..T...G..A.......CC----..T.---..-.T.........T.-...-.-.T.-.-....................A.TG........  151
Query_70023  266                                                                                          ...........                     256
Query_70024  85   ...-...-..G..A.T...-.........T........CC....--------..CC.-...-TT.........-..A-.-.-.G.-......................A.TGT........  186
Query_70025  109  ...-......G..A.T...-............TG...........CC.----.T.----..-.T.........-..A-.-.T.-.-.......................A.TG......A..  212

Query        223  AAA-GGGAG-AGA-GTCCTTGGAGCCTTGGGGCCATGTGGTCAGTATTTGGGAGGCATCCACTTCCTGATGGTAAATAGATTTAAG--CACGCCATCTGTTGTGTGCTTATTTCCCAGGA  337
Query_69995  222  ..G-..A.C-T.C-..T..CA......CA.C.....................A...T..........CA.....C........A--...A..G.......T..CAG.........T...  336
Query_69996  227  ..G-..TGC-..T-..T.........G.A.C.A....................A.............C......C........A--T..C.........T..CAG.........T...  341
Query_69997  218  ..G-..A.C-..G-.GT.........ACAAA...CCA.......C.......T.A....T.........CT.....C........A--T..A.........T..CAGA......T...A.  332
Query_69998  228  -..-.....C..T-..T......A..ACCA...........G...........A.GG...........CA.....C........A--T..A.........T..CAGG............  342
Query_69999  228  ..G-..A.T-C.C-..T.........ACA.T.............A..........A....T..........C......C....CG...TAT..A..........T...AG.........T...  344
Query_70000  227  ...G.....-...-..................................A...T.............C...............--.C.A..........CAG.............  342
Query_70001  180  .GC-..A.C-..T-..TTC.........AG..T......A.................A....T..........CA.....CG....G..A--T.TA.........T.ACAG..C......T...  294
Query_70002  215  ..C-..A.C-T.C-..T..A.......CA.C...C..........G..A..A..A...T..........CA.....C........A--T..A..C......T..CAG.........T...  329
Query_70003  137  ..G-....C-.AT-..T.........ACA.--.AG..................AG..T...........C.....GC.A..C.G.A--G..A.T......GT..CAG.........T...  249
Query_70004  229  -C.-....A-C..T..TT........GG..T....................A....T..........C......C....CG..A--T..A.........T...AG...C......T...  343
Query_70005  207  ..G-..A.C-..G-..T.C.......ACAAA...CCA..............T.A....T..........CT.....C........A--T..A.........T..CAGA......T...A.  321
Query_70006  207  ..G-..A.C-..G-..T.........ACAAA...CCA..............T.A....T....T....CT.....C........A--T..A.........T..CAGA......T...A.  321
Query_70007  214  ..G-..A.C-T.T-..T.........ACA.A..............G........A.............CC.....C.....G...A--.C.A.........C..CAG.G.C......T...  328
Query_70007  109                                  ............                                                                              98
Query_70008  197  ..G-.AA.C-.AT-..T.........ACA.CATGG........T...C......A...T..........A......C.A......A--G..A........GT...AG.........T...  311
Query_70009  179  ...--.A.C-..G-..T.........ACAAA...TCA.....G...........A....T..........CC.....C........A--T..A.........T..CAGC......T...A.  292
Query_70009  71                                    ...........                                                                             61
Query_70010  210  -..-..A...C..T-..T.........ACA.CA....................A.............CA.....C........A--TG.A.........T..CAG......TT.T...  324
Query_70011  224  ..G-..A.C-T.C-..T..A.......CA.T...C..........G..A..A..A...T..........CA.....C........A--T..A..C......T..CAG.........T...  338
Query_70012  193  ..---.A..C...-..T.........GCCA.......C...............A..G...........CA..G.GC........A--T..A.........C..CAG..G....T.....  306
Query_70013  211  ...-..A.C-..T-..TT........AGA.T.................A....A....T..........C......C.....CA..A--T..A..G......T...AG.........T...  325
Query_70014  150  -G.-.....-C..T..T.C...G....CC.T..TG..C.......G.....A..A.T.T...................C........A--G..A.........T..CAG......G..C...  264
Query_70015  74   .TC-...TC-..C-ACTGGG...AG.AC.C..............G........A.........C....G.......C.CG..C.GA--TT.A.......C.T..CAG...C.GTTG....  188
Query_70016  260  TTG-..A.C-..C-ACT.AC......A.AT.......................A.............G.......C.C......TA--TT.A.........T..CAG......G......A.  374
Query_70017  223  ...-.....-...-.....................................................C................--..TA.................G..........G...  337
Query_70018  157  ..G-..A.C-C.G-..T.........ACAAA...CCA..........C.......A..G.T.........CT.....C........A--T..A.........T..CAGA......T...A.  271
Query_70019  227  ..G-..A.C-.AT-..T.....C...ACA.CA.AG..................A..CT...........CA.....C.A......A--G..A........GT..CAG.........T...  341
Query_70020  228  ..G-.AA.C-C.T-..T.........-C..T...G....A..G.CT......CCA.....G........GCA.....C.........--T..A.........TC.CAG.....C....T...  341
Query_70021  224  G.G-C.CCC-..C-..T.C.......ACA.A.G.......C....GG.........A.T...........C......C....C....A--TG.A.........T..CAG..T..C..TT...  338
Query_70022  228  C.G-..AC.-..C-A.TGC.......GCA.A............CG..CA..C.A............G.....G.GC.CG.C...A--TG.A.........CC.CAGAG.-C.....C...  341
Query_70023  152  ...-..A.C-..G-A.T.........ACAAT...CC...................A....T..........CCA....C........A--T..A.........G.T..CAGCG.....TTG.A.  266
Query_70024  187  ..G-..A.C-G.T-.CT.........ACA.A.A.C..................A..GT...........C......C........A--T..A.........T..CAG....C....TG...  301
Query_70025  213  ..G-..A.T-G.T-..T.........ACA.C.............G.........A..........C.........C.C......A--..A.........T...AG...CC....T...  327
```

ECR11

```
Query          9    AAGCAAAGCATCTCAGAACACTAG-CTGTTAGGATGT-CAGT-TT--C-ATC--A-G-GTTGCCAAGTAAAACCCCACAGCAACTGCCCACCATGGAATTTT-CC-C--TGA-TT-AATG  112
Query_17577    1                       ....-...........T...C-..--G-G..--C-A-.......AC........GG.....A.T....C.......-..-.--CC.A..-...A  87
Query_17578    1                                .........-...C-G.--G-G..CC.-.-..-.....A....G.A..GAG...G.CA.....G.......C-..A.--..-..-....  79
Query_17579    1                       ....-...........-TT.GC..--G-G..--C-A-.......AC........GG.....A.....G.........-..-.C-..-..-...A  87
Query_17580    1                       ....-...........T...C-..--G-G..--C-A-.......AC........GG.....A.T....C.......-..-.--CC.-..T...A  87
Query_17581    5                                                            .-.G.....A.......T.GG...GTCA..T...G...C...C-..-.C-..-..-....  62
Query_17582    1                       ....-...........T...C-..--G-G..--C-A-.......AC........GG.....A.T..T..C.......-..-.--CC.-..T...A  87
Query_17583    1              ....T.........G...........-....-..--G-G..--G-A-.C.....................................-..-.--..-..-....  95
Query_17584    15                                                                                                      ..-.--..-..-....  26
Query_17585    1    .......T......T.........-.C..........-...C-..--G-G..--C-CA.......A....TG...GG...G.CA............C-..-.--..-..-....  105
Query_17586    1                             ...........-...C-..GG.-T..--.-.--......A.......AT.GG...GTCA..T.......C..C.-..-.C-..-..-....  82
Query_17587    1                       ....-...........T...C-..--G-G..--C-A-.......A.........GG.....A..-...C.......-..-.C-..-..-...A  86
Query_17588    1                       ....-...........T...C-..--G-G..--C-A-.......AC........GG.....A.T....C.......-..-.--CC.A..-...A  87
Query_17590    1                           .-.............-...C-.G--G-G..--CT.-.......A....TG.-GG...G.CA.T...........C-..-.C-..-..-....  82
Query_17591    1    .......C......T.........-.C..........-...C-..--G-G..--C-CA.......A.....G...GG...G.CA............C-..-.--..-..-....  105
Query_17592    1                         ...-.........C.-...C-..--G-G..--C-A-............C....CAG...G....T.G.-.......CC--..-.--..C-..-....  81
Query_17593    1                       ....-...........T...C-..--G-G..--C-A-.......AC........GG.....A......C.......-..G.--..-..-...A  87
Query_17594    1                                                                                                       ..-.--..-..-....  12
Query_17595    1                                                                                      .....G........-..-.C-..-..-....  29
Query_17596    1                         ...-.............-....-..--G-G..--C-A-.......A......T..GG.....A.......A......T..-.--..-..-....  84
Query_17597    1     ........G...............-....C.......-...C-..--.-..--.-A-....................................................-..-.--..-..-....  103
Query_17598    1                       ....-...........-TT.GC..--G-G..--C-A-.......AC........GG.....A.....G.........-..-.C-..-..-...A  87
Query_17599    1                                .........-...C-.C--GG.C.--C-A-.C.....A.....G...GG...GACA.....G.......C-..-.C-..-..-....  79
Query_17600    33    ......TGT....T.C.A....-.......A....-...C-..--G-G..--C-A-.......A.........GG.....CA...............-..-.C-...-.G-...A  136
Query_17601    1                                  .......-.G..-..--G-G..--C-A-.......AC.......GAG...G.......GG.-.....C-T.-.--..-..-G...  74
Query_17602    1                                                                                                       ..-.--..-..-....  12
Query_17602    218               ...........                                                                                       208
Query_17603    1                                                              .........G......GGG-...G.CA.....AGA.......-..-.C-..-..-....  56
Query_17604    19                                                             .......G........TTGGTT....AAT..G...........-..-.CC..-..-....  76

Query          113  ACCTACGAATGCCCGGCTGGCGTCCAAGCTCTATATACATTGACATCCAACTCAGGA-AGGGAAAC-----------AA---TTT--TCCCAGAAACAGCACAACATGGC--------CA  207
Query_17577    88   .................T.A.........C....G..C.....C..........-.-..C..-----------GG---..--...................A..---------..  181
Query_17578    80   ...CC..CG........C.A.........C................G.G....-.....GCA----------TT---..--........-......-...C.---------..  172
Query_17579    88   .................T.A.........C...........................-...-..-----------.G---..--...................A..---------..  181
Query_17580    88   .................T.A.........C....G..C.....C..........-...-..-----------GG---..--...................A..---------..  181
Query_17581    63   ........G....A....T.G.......T......G...........G.......-...-..-----------GG---..--.........................TGTTGTAG.C  164
Query_17582    88   .................T.A.........C....G..C.....C..........-...-..-----------GG---..--...................A..---------..  181
Query_17583    96   .....T...G...A.....CT.......A.......G..................-.......G----------T.---..--.................................---------..  190
Query_17584    27   ....CT.......A....ATA.G.....A......G.................T.-.A..-..-----------.G---..--.......................-...---------..  119
Query_17585    106  ......A......A....CAA.........C........C...............-CA....C.GTTTTGACAGG..CCG...--..........C......-...C.---------..  213
Query_17586    83   ....C........A...AT.A.......T......G..G.................-...-..T----------GG---..--..................................---------..  176
Query_17587    87   .................TAAA.........C....G........C...........-...-..-----------GG---..--...................A..---------..  180
Query_17588    88   .................T.A.........C....G..C.....C..........-...-..-----------GG---..--...................A..---------..  181
Query_17589    1                         ...................A..........-..-.A....T----------GG---..--..................................---------..  73
Query_17590    83   .....T..G.....A...CAA.................C.........T........-...-..-----------.G---..--..................................---------..  176
Query_17591    106  ......A......A....CAA.........C........C...............-CA..-..-----------CG---..--..........C......-...C.---------..  198
Query_17592    82   ....GT...........GTCA...........GC...TG...G.............-...-..T----------GG---.C.--............T.....G..A.---------..  175
Query_17593    88   .................T.A.........C....G........C....T.......-...-..-----------GG---...CC...............-....A..---------..  182
Query_17594    13   .T..C...G....A....T.A.......T......G..G.C...............-...-..-----------GG---..--..................................---------..  106
Query_17595    30   ....TG..CC...A....CA...........G.....G..................-...-..-----------.G---..--..........T.T.....................---------..  123
Query_17596    85   ....T.A...........CATA....................T.............-...-..-----------.G---..--..............T.....................---------..  178
Query_17597    104  ........G...........T.................G..........G......-........----------T.---..--..........T......................---------..  198
Query_17598    88   .................T.A.........C...........................-...-..-----------.G---..--...................A..---------..  181
Query_17599    80   .......G.G...A....CAG..........CG.......C....C...G......-....-..T----------GG---..--..........AG......-.C.C.---------..  172
Query_17600    137  .A...A.....T......T.A...T......C........................-....-..T----------GT---..--...............A.....G...---------..  230
Query_17601    75   ......CC..................G....GG.G....CC.C.G.-G....--...G...AG.G.-----------CT---C..--...............T..G.G...---------..  167
Query_17602    13   .....T...........T.A....G......G........................-.......-----------GG---C..--.....................CA..---------..  107
Query_17603    57   C...C.A...........C.A.........C..........C..............-....-..T----------GG---..--.T.................CA..---------..  150
Query_17604    77   .G..C--..C..TG.AT...GA......A.A....C..G..TCA.............-....  135
Query_17605    1                         ........................T..........-...--..T----------GG---..--.....................................---------..  72
```

ECR12

```
Query        15   TTCACAAGAGAGACTGCTGAGCCCATCCCCCAGCCAGGACACTT--GGCCCTGAGTTGCTCAAGAGCCCTCATCCTCAGTCATTGCAGGGACTTGTTGCCATGGAAACTGCAGC--C-AG  129
Query_39765  79                                                             .........T....C....T.C..........G..-..CT............T...--.-..  136
Query_39765  147                                                                                                    ..........GCTG..--T-.C  129
Query_39766  62                                                             .........T..TGC....T.C.....A..A.G..-..CT............T...--.-..  119
Query_39766  130                                                                                                    ..........GCTG..--T-.C  112
Query_39767  101                                                            .........T...--....T.C..........G..-..CT............T...--.-..  156
Query_39767  167                                                                                                    ..........GCTG..--T-.C  149
Query_39768  101                                                                                 ...........G..-..CAT.........C.TCA.--T-..  137
Query_39768  148                                                                                                    ...........CT..T--G-.C  130
Query_39769  142                                                                                               ...............C.T...--.  163
Query_39769  51                        .................  67
Query_39770  77                                                             .........T....C....T.C.G........G..-..CT............T...--.-..  134
Query_39770  145                                                                                                    ..........GCTG..--T-.C  127
Query_39771  1                    ........................T..CTC..T..G.......................G..............G.C.................T...--.-..  101
Query_39772  101                                                                                           .....................--.-..  124
Query_39773  97                                                                                            .....................TGG-..  122
Query_39773  1                         .............  13
Query_39774  125                                                                                                             ...--.-..  130
Query_39774  1                         .................  17
Query_39774  89                                                                                            ...............  103
Query_39775  110                                                            .........T....C....T.C.........AG..-..CT............T...--.-..  167
Query_39775  178                                                                                                    ..........GCTG..--T-.C  160
Query_39776  79                                                             .........T....C...TT.C..........G..-..CT...........AT...--.-..  136
Query_39776  145                                                                                                      ........GCTG..--T-.T  129
Query_39777  95                                                                                            ....A................  115
Query_39777  1                         ...........A.....  17
Query_39778  101                                                            .........T....A..A---............G.-..CA..........CAT...--T-..  155
Query_39779  79                                                             .........T....C....T.C.........AG..-..CT..........G.T...--.-..  136
Query_39779  147                                                                                                    .........-...T.G--.T.C  129
Query_39779  12                           ...........  22
Query_39780  88                                                                                            ...............C.T...--.-..  111
Query_39781  52                                                                                            ...........C.....T...  72
Query_39782  56                                                             .........T....C....--..T.A......G..-..C....A...G....T...--.-..  111
Query_39782  4                         ....A............  20
Query_39783  1    .................C..........................--............................T.........................G.............--.-..  115
Query_39784  12                                                             .........T..TGC....T.C........A.G..-..CT............T...--.-..  69
Query_39784  80                                                                                                     ..........GCTG..--T-.C  62
Query_39785  101                                                            ..............C..-.T....C.....A.C--............G..C.....--.-..  156
Query_39786  79                                                             .........T....C...TT.C.........CG..-..CT...........AT...--.-..  136
Query_39786  145                                                                                                      ........GCTG..--T-.T  129
Query_39787  51                                                             .........T....C.....-...........T..-..CA..........A.T...--.-..  107
Query_39787  118                                                                                                    ....A.....ACTG..--T-.C  100

Query        130  --T-G----TTTCCATGGAGAAAGAG----C---T-GC---A-----GAGTCTGGAATTTTCCAGGTTCTAAAGGTGAATGTAT----ATTCTCTTG-AAAA-CAACTCTGAATGCTTTC  220
Query_39765  137  --C-.----.................C---.---C-..---.-----...................C...........C..  195
Query_39765  128  --A-.----............C..  112
Query_39766  120  --C-.----...............G.----.---C-..---.-----..A................C..........  173
Query_39766  111  --A-.----............C..  95
Query_39767  157  --C-.----.................C---.---C-..---.-----...................C...........C..  215
Query_39767  148  --A-.----............C..  132
Query_39768  138  --A-.----.................----.---C-T.CAC.-----...................C............  196
Query_39768  129  --G-.----........  120
Query_39769  194                ............----.---C-A.---.-----.CA............TGT.C..............  244
Query_39770  135  --C-.----.................C---.---C-..---.-----...................C...........C..  193
Query_39770  126  --A-.----............C..  110
Query_39771  102  --C-.----.................----.---.-..---.-----...C................................C----...AG....A....-........  184
Query_39772  125  --G-A----G--.AGG....GG..G.GGT-.---.-..---.CCAGT...G...............C...  177
Query_39773  123  --.-.GGAAAGAGG.G.....CT...GAAT.---A-..---.-----...A..............C.C.AG...C...  185
Query_39774  131  GC.-.----.A.GG...C........----T---C-AT---G-----.................A....C............  191
Query_39775  168  --C-.----.................----.---C-..---.-----..C................C...........C..  225
Query_39775  159  --A-.----............C..  143
Query_39776  137  --C-.----.......A.........----.---C-..---.-----..................CC...........T..  194
Query_39776  128  --A-.----............C..  112
Query_39777  150                                      ..---.-----...A..............  170
Query_39778  156  --C-.----.................----.CTT.-.T---G-----.G....C............C...T..........C..----.  220
Query_39779  137  --C-A----.................----.---CC..---.-----.......................G.......C..  195
Query_39779  128  --C-.----............C..  112
Query_39780  139                ............----T---C-..---.-----.................T.C..............A..  192
Query_39780  112  --.-.----.  114
Query_39781  101                ............----.---.-..---.-----.G................CC...G........  149
Query_39782  112  --.T.----.................----A---.-..---.-----.......A..C.......AC...C.........  169
Query_39782  159                                           .-----..........AG..T...  141
Query_39783  116  --C-.----.................----.---.-..---.-----.....................................----....G....-....-........C..  200
Query_39784  70   --C-.----...............G.----.---C-..---.-----..A................C..........  123
Query_39784  61   --A-.----............C..  45
Query_39785  157  --.-.----...........-...G.----.---.-T.---.-----..A........C....CA.C............  211
Query_39786  137  --C-.----.......A.........----.---C-..---.-----...................C...........T..  194
Query_39786  128  --A-.----............C..C.C---.---.-..---.  104
Query_39787  108  --.-.----....T.........TT.----.---C-..---.-----..................CC..............C..ATGG....AT.A.-....T..C...A.C.....C.A  203
Query_39787  99   --.-.----.........T..C...  82
```

ECR13

```
Query        1    CACCTCTACCCACTCACCCCCAGCCCCAAAAGA-GAAAACGAATGGAAAAGCTAAGAAAGACAGACTTGGAAGGATATAG-C-TGG------------TAGTCCTGACTGCTTTTAGGGT  105
Query_37303  57                                                                         .....G..GC-.-...------------..T.--...-.AT...C.....  89
Query_37304  81                                                                                                                  ...C.....  89
Query_37305  47                                                                                                                 ....C.T.A.  56
Query_37306  81                                                                                                                  ...C.....  89
Query_37307  10            ...A..G.....CC...A....A.A.........T.........AG....G....A...........T-.-..------------...C.............T...  106
Query_37309  46                                                                                                                  ...C.T.A.  54
Query_37310  6                                                                                                                           .  6
Query_37311  81                                                                                                                  ...C.....  89
Query_37311  8                                      ......T.GGA..........G...                                                            33
Query_37312  51                                                                      ..G......G..G.-.-...------------CGT..--..-..T...CCT...  88
Query_37312  16                                                ..........G...G..                                                         33
Query_37313  98                                                ..G............AGA..C.......G.G..-.C...GAAACGGATAGC....GT.A.A.AT...C.T.A.  171
Query_37314  1    ....C..............T......C....C-......A...T..........G......................-.-.A.------------.................T...  105
Query_37315  101                                                                                                                    ....  104
Query_37316  101                                                                                                                     ...  103
Query_37316  31                                                 ..........G...G..-..AG........                                           60
Query_37317  350                                    ....T..GA...............---..A.....A......T.-...AAAGGAGATGGC....GT.A.A.ATA..C.T.A.  271

Query        106  TTGGAAATGCTTGTCTTCCCAACAGAGGCCAA--T--T--GAGATACGT--CCAAGAAACCCAACCGCTTCCTGCAGGGGAGGAGATGTATGTATTGCTTGGTTTGTTGCCGAGAGCCAA  217
Query_37303  90   .C.....C..A......AGC..........--.AT.--A..T.GTT.--........T........C.....TA.A..........C...GG.T......CCA......CA.TG.  203
Query_37304  90   .C.....C..A......AGC..........--.AT.--A..TAGTT.--........T...G..........TA.A.........C.C.A.GG.T......CCA......CA.TG.  203
Query_37305  57   .......CT.A......AG........T...--.GT.--...T.GTT.--........T.T............TA.......A...........T......CCA......CA.TG.  170
Query_37306  90   .C.....C..A......AGC..........--.AT.--A..TAGTT.--........T...G..........TA.A..........CA..GG.T......CCA......CA.TG.  203
Query_37307  107  ..............................--.--.--.......T.--.......................T.....A......................A.......C.....  218
Query_37308  250                      ........T..GCG.--.--...T.GTT.--........T......A........TAA...C.CA..........TT......C.A......CA.TG.  157
Query_37309  55   .......CCTA..A...TGG........T...--.--GTG...T.GTT.--T.......T..............TA.......A...........T......C.A......CA.TG.  168
Query_37310  7    .......CC.A......TGG........T...CG.--.--...T.GTT.--........T..............TA.......A...........T......CCA......CA.TG.  120
Query_37311  90   .C.....C..A......AGC..........--.AT.--A..T.GTT.--........T..............TA.A...............G.T......CCA......CA.TG.  203
Query_37312  89   .C.....CA.AC......AG..........--.--A--TTA..TT..TT........T......A........TA.A...............G.T......CCA..A...CA.TG.  202
Query_37313  172  .......CC.G.......GG........T...--.GT.--...T.GTT.--......GCT....A..........TA.......A.........C..T......CCA......CA.TG.  285
Query_37314  106  ........A.....................--.--.--.......T.--...........................GA......................A.......C.....  217
Query_37315  105  ..........C.......AG............--CAC.--...TA.TT.--........T...............A......C.....G..CCG........C..CA...C..CC.TG.  218
Query_37316  104  .......CA.AA......AG............--.--A--CTA..TT..TT........T..............TA.A...........A...G.T......CCA......CC.TG.  217
Query_37317  270  .......CC.A.......AG............CG.--.--...C.GTT.--........T..............TA......CA.C.C....GCCAT.......CCA......CA.TG.  157

Query        218  GCTAA-ATGAACCACGGTGAGTCCATGAACT-A-AG-TACAATCTGTCGACG-GGGGCCAGGAAACAAAGTTCA-A-A-G-ACTCC------TTTTTGAAGAGCACAAGTTTCCAATTAG  322
Query_37303  204  .....T..C.............TT......A.-.A-..T......AT...--CA........GG...TA.C..TT-C-.-G....------...CCA.GA.C....C............  310
Query_37303  234                                                                      ......-.-.-.-....                                      222
Query_37304  204  .....T..C.............TT......A.-.A-..T......AT...--T.........GG...TA.C..-.TC-.-G....------...CCA.GA.C....C..........G.  310
Query_37304  234                                                                      ......-.-.-.-....                                      222
Query_37305  171  .....--.................T......-.-.AA..T......GT..TT-...-.......G...CA.C..-.-.-CT...T.------....CC.G..C.................  275
Query_37306  204  .....T..C.............TT......A.-.A-..T......AT...--TA........GG...TA.C..-.TC-.-G....------...CCA.GA.C....C............  310
Query_37306  234                                                                      ......-.-.-.-....                                      222
Query_37307  219  .....-........................-.-..-....G....AT....-...............CT.C..-.-.-.-.....GGTTTT..........................  329
Query_37308  156  ...-.-................TT......A.-.A-......--..AG..-T-T..........G...CA.C..-.-----........------..C..                   80
Query_37308  128                                                                      ......-.-.-.-....                                      140
Query_37309  169  ...-.-..................T......A.-.A-..T......AT..-T-T.T........G...CA.C..-.-.CT-C..T.------....CC.G..C.....A...........  273
Query_37310  121  .....--.................T......-.-.AA..T......AT..TT-...-.......G...CA.C..-.-.-CT...T.------....CC.G..C.................  225
Query_37311  204  .....T........T.........TT......A.-.A-..T......AT...--T.........GG...TA.C..-.TC-.-G....------...CCA.GA.C....C............  310
Query_37311  234                                                                      ......-.-.-.-....                                      222
Query_37312  203  ...-.-.....G.........C.TT......-.C..-C.TG.....AT.-.C-T..........G...TA.C..-.-T-C-GG...------....CA.GA.C......G..........  306
Query_37313  286  .....--.................T......A.-.A-C.T......AT...--T..........G...C..C..-.-.CT-...T.------....CC.G..C.................  390
Query_37314  218  .....-........................-.-..-...G......T..T.-........................-.-.-.-....------.....A....................  322
Query_37315  219  ...-C-.G..G......A.C....T......---..-A.TGCG...C..TG.CC..........G...CC.C..-.-.-.-G....------.C..G..GC..................  322
Query_37316  218  ...-.-.................C.TT......-.C.A-C.T......GTA..--C..........G...TA.C..-.-T-C-GG..G------....CA.GA.C.................  321
Query_37317  156  .....--...C............TT......-.-.AA..T......AT..TT-...-.......GTG.CA.C..-.-.-CT...T.------.....C.G..C.................  52
Query_37317  128                                                                      ......-.-.-.-....                                      140
```

ECR14

```
Query         116  AACATCTGG-TCCAGGTGCCAGATAAAA-CTTTGCTTCAACAACCCACCTCAGAA-TTTT-TTCCCTGATTAATGAGC-T-CAGTCGTGGAGTGAAATCCAAG--CA--GTATATACA--  223
Query_133282  87   .......CA-C....T............-.C.C.G..............ATG..--..-...................-.-A.A.TC..T.C..G.......--.--A......TG--  193
Query_133283  136  ...C.....-C.................-.C.CAG..............ATG..--...-.C................-.-A.AGGC...C..G.......--.--A......TG--  242
Query_133284  137  .......C.-C....T............-.C.C.G..............ATG..--..-.............C....-.-A.A.TC..T.C..G.......--.--A......TG--  243
Query_133285  137  .......CA-C....T............-.C.C.G..............ATG..--..-...................-.-A.A.TC..T.C..G.......--.--A......TG--  243
Query_133286  132  ....A..A.-..T..T............-.....G..............ATG..--C..-..................-.-A.A.TC..TGC..GGG.....--.--A......TG--  238
Query_133287  152  .....TG..-C....T............-.C.CAG..............ATG..--..-...................-.-A.A.GC....C..G.......--.--A......TG--  258
Query_133288  137  .......CA-C....T............-.C.C.G..............ATG..--..-...................-.-A.A.TC..T.C..G.......--.--A......TG--  243
Query_133289  116  .....A...-..................-.......................G...T....-...................-.-A....A....C..........--.--A........--  224
Query_133290  144  ......A..-C..C.T............-.CCCAG......G.......A.G...-.....-.C..............-.-GGA.GCC...C..G.....G..--.--A...G....T-  252
Query_133291  134  .....TG..-C....T............-.CGCAG...G..........ATG...--.A.-..................-.-A.A.GC......G.......--.--A......TG--  240
Query_133292  136  ........A-C....T............-.C.CAG....G.........-GT.G.-G...-....T..........C.-.-..A.GC....C..C.......--.--A......TG--  242
Query_133293  139  .........-C....T............-.C.CAG.............TATG...-.....-C.......G........-.-A.A.GC....C..G........--A.--A.......G--  246
Query_133294  113  ..A..TG..-A....T............-.C.CAG..............ATG...--..-..................-.-A.A.GC....C..G...T.....--.G--.......TG--  219
Query_133295  117  .......T.-C....T............-.C.C.G..............ATG...--..-.............C....-G-A.A.TC..T.C..G.......--.----.....T.--  221
Query_133296  107  .....-.CA-G...AA...........                                                                                          131
Query_133296  147                                                                   ..-...........G..                                   162
Query_133297  110  .........-C.T..T............-.C.CAG...T..........ATG...-.....-C...-.............-.-A.A.GC..A....G.......--.--A........--  216
Query_133298  144  .........-C....T............-.C.CAG.............TATG...-.....-CC...G...........-.-A.ACAC....C..G........--A.--A......TG--  251
Query_133299  117  .......CT-C...T-............-.C.CAG..............A.G...-.....-.-..........C....-.-A.A.TC..T.C..G.......--.--A......TG--  222
Query_133300  120  .........-C..T.C............-.C.CAG..........G..ATG...--CG.-..................-.-A.A.GC..T.C..G.G....A--.--AC.......TG  228
Query_133301  107  .........-C..C.C............-.C.CAG..........G..G.G...---.G-CG..........C....-.A..CG.-....C..C.G.......--..ACA....G..C--  214
Query_133302  112  ........A-C....T............-.C.CAG.......G......-GT.G.-G...-....T..........C.-.-..A.GC....C..G.......--.--A......TG--  218
Query_133303  100  .......A.-..T..T............-.C.CAG..............ATG...--..-..................-.-A.A.TC..TGC..G.......--.----.....T.--  204
Query_133304  113  .......C.-C....T............A.C...G.............TATG...-.....-.-..........C....-.-A.A.TC..T.C..G.......--.--A......TG--  220
Query_133305  112  ......G..-C....T............-.C.CAG..............ATG...--..-..................-.-A.A.GT....C..G.......--.--A......TG--  218
Query_133306  122  .........-CT...T............-.C...G..G...........ATG...-.....-G.--..............-.G..T..-....C..G.......--.--.CC......--  227
Query_133307  107  .....TCA.-C...A............T-.-----------..TT.....G...-.....---.................-.-A...G..A.T...GCG......TC.T--......T.---  202
Query_133308  114  .........-..................-.......................G...-.....T..................-.-A.........C..........--.--A........--  222
Query_133309  117  .......C.-C....T............-.C.C.G..............ATG...--..-.............C....-.-A.A.TC..T.C..G.......--.--A......TG--  223
Query_133310  121  .GT......-C....C............-.C.CAG..........G.TATG...-.....-CCT..............-.-A.A.GC....C..G.......C--A.--A......TG--  228
Query_133311  144  ......A..-C..C.T............-.CCCAG......G.......A.G...-.....-.C..........C....-.-GGA.GC...GC..G.....G..--.--A....C...--  251
Query_133312  105  .........-..TGAC............-.C.CA.......C..TGGG.-.T.G.-A...-.C..T.............-.-.......-....C..G.......A--.C--A....G...  210
Query_133313  107  ....-..CA-G...A............T-.-----------..TT..T..G...-.....---...............C.-.-A.CCT..A.CC..G.G...C..--.T--C.C.C..T.--  200
Query_133314  68   .........C...GC..-....C..G..-.C...G..............A.G...--...-.C...............-.A..T..-.....CG.CG.......--.----.....G.--  172
Query_133315  117  .......C.-C...T-............-.C.C.G..............ATG...-.....-.-..........C....-.-A.A.TC..T.C..G.......--.--A......TG--  222
Query_133316  123  .........-C....T....TA......-.C.C.G...........T..ATG...--..-..................T.-A.A.TC..T.C..G...T....--.--A......TG--  230
Query_133317  68   ......G..-C....T......G.....-.C...G........T..G..ATG...-.....-CC...............-.-..ACGC....T..GG.......--AT--A....C..G--  175
Query_133318  129  .........-C.T..A....C.......-GC...G..............GTG...--..-..................-.-A.A.TC....C.CG.......--.--A.....CTG--  235
Query_133319  119  .........-C....T............-.C.CAG...T..........ATG..C-....-.--...............-.-A.A.GC......G.......--.--A......TG--  224

Query         224  -TACCA--CTGCCCAGCTCAGG-AGGAGACTGGCTTTCTC-GGAAAGAGTACAACCCAGCCAGTGTTCTGGGTGC  293
Query_133282  194  -.TG.C--A.--...A......-.A...............-........C.......C...........      257
Query_133283  243  -.TG.C--A.--...A......-.A.G.......C.....-.....C..C......A............      305
Query_133284  244  -.TG.C--A.--...A......-.A..C............-........C.......C...........      307
Query_133285  244  -.TG.C--A.--...A......-.A...............-........C.......C...........      307
Query_133286  239  -.TG.G--A.--...AG.....-.A.....A.........-....G...C....................      302
Query_133287  259  -.TG.C--A.--...A......-.A.G.............-...........................      321
Query_133288  244  -.TG.C--A.--...A......-.A...............-........C.......C...........      307
Query_133289  225  -.G.TG--.C.T..........-.A...............-..................T.........      289
Query_133290  253  -.G...TC.C....G.----.-...-...C.A........-A.......C...G................      315
Query_133291  241  -.TG.C--A.--...A....T.-.A.G............T-....T.....................      302
Query_133292  243  -.TG.C--A.--...A......-.A.G.............-........C....................      305
Query_133293  247  -.TGTG--A.--...A......-.A.G..A..........-.......AC......G........      306
Query_133294  220  -.TG.C--A.--..........-.A.G.............-................C...........      282
Query_133295  222  -.G.TG--.CAT...A......-.A...............-........C.......C...........      287
Query_133296  221                 ......-.A.G..A..........-..........A..TGTG.....GA.......      274
Query_133297  217  -.TG.C--A.--...A......-.A.G...C.........-........C...................      279
Query_133298  252  -.TG.C--A.--...A......-.A.G..A..........-A.......C......TG.........      311
Query_133299  223  -.TG.C--A.--...A......-.A...............-........C.......C.........C..      286
Query_133300  229  T.G...--T..----A......-.A.G............T-........C......G............      290
Query_133301  215  -.G...-----T..G..C.G..-.A.G.C.C.........-........C......-.........      273
Query_133302  219  -.TG.C--A.--...A......-.A.G.............-........C...................      281
Query_133303  205  -.G.TG--.CAT...A......-.A..............T-........C...-..........G....      269
Query_133304  221  -.TG.C--A.--...A......-.A...............G........C....T..C...........      285
Query_133305  219  -.TG.C--A.--...A......-CA.G.............-............G...............      281
Query_133306  228  -.TG.C--A.--..........-.A.G.............-........C...G...............      291
Query_133307  203  -.G.----.GT.TT.A......-.A.G.G.........CT-.....C....A..TGTG..........C..      266
Query_133308  223  -.G..G--.CA...........-.A...............-........A.........................      292
Query_133309  224  -.TG.C--A.--...A......-.A..C............-........C.......C...........      287
Query_133310  229  -.TGTG--A.--..........-.A.T..A..........-.....A..C................      288
Query_133311  252  -.                                                                           252
Query_133313  201  -..T..C-..T.T..-.G....-.A.GC.A..........-.....C..C.G..TGTG......A.......      267
Query_133314  173  -.GTTG--.CAT...A...C..-.A.G...C........G-........C...G..................      239
Query_133315  223  -.TG.C--T.--...A......-.A..AG...........-........C.......C...........      286
Query_133316  231  -.TG.C--A.--...A......-.A.....C.........-........C...G.........CA......      294
Query_133317  176  -.TT..--A.--...A......A...G.GA..........-....T...C...G....A.........      237
Query_133318  236  -.TG.C--A.--....T.....-GA.G.............-........C......T..T.........      298
Query_133319  225  -.TG.C--A.--...A.A....-.A.G...C........-A.......C...................      287
```

ECR15

```
Query        4    TGCTGTTTCTCTGT--AAGGAATTTGGTCAACAGCtttttttttCCCTGAAGAAAGTGAAGGATGTGAAATGGTGGGGTTGTGTAGCAGT--AGAGACCTCCTAAG-AAGACGATGGAGC  118
Query_59657  98                                                                                           ..--G.............-....G.TAA...T  126
Query_59657  134       .........AA......AA....                                                                                             156
Query_59659  101                                                                                          ..--..............-....G.TAA...T  129
Query_59660  98                                                                                           ..--G.............-....G.TAA...T  126
Query_59660  134       .........AA......AA....                                                                                             156
Query_59661  98                                                                                           ..--G.............-....G.TAA...T  126
Query_59661  134       .........AA......AA....                                                                                             156
Query_59662  56                                        ..................A...........G...........CA.A......GGG..............-....G..A.....  137
Query_59662  1    ...........CA.--........CA...TG.....                                                                                    34
Query_59663  51                                                                                            .--............G.-....G.GAA....  78
Query_59664  101                                                                                          ..--..............-....G.TAA...T  129
Query_59665  98                                                                                           ..--G.............-....G.TAA...T  126
Query_59665  134       .........AA......AA....                                                                                             156
Query_59667  9                                                                                                ...A...C...T..G..T.G..A.T...  36
Query_59668  98                                                                                           ..--G...........G.-....G..AA....  126
Query_59669  1    ..............--........CA....G..........C--...C........................................C.--..............-....T........  113

Query        119  CTTGGCTTGGCTCTGGGAAGGAAAAA--TG---TT---CTGACATTTCACTGATCCAGT--GG--AAAATG--------GAAC-GCCCAAC----CTCTGTTCAGCTCTGTCTCTGTGCG  213
Query_59657  127  ....TTC.TT.....TA..A.G...--..GTT..---..A..G...T....T.....A--.A--..G.CA--------A..-.......----...CA..T.CA.......A.C...  224
Query_59658  9    .....TC..T.....T...A.GG..--..---..TTT..A.........CT....AAA.A--....AA--------....-A...G.----...CA....A.G...G......T.  108
Query_59658  14                                                                                                          ............    25
Query_59659  130  ....TTCC.T.....TA..A.G...--..GTT..---..A....G.T....T.....G--.A--..G.CA--------A..-.......----...CA..-..A.........A.....  226
Query_59660  127  ....TTC.TT.....TA..A.G...--..GTT..---..A..G...T....T.....A--.A--..G.CA--------A..T-.......----...CA..T.CA.......A.C...  224
Query_59661  127  ....TTC.TT.....TA..A.G...--..GTT..---..A..G...T....T.....A--.A--..G.CA--------A..-.......----...CA..T.CA.......A.C...  224
Query_59662  138  ..C......................--..---..---.......................--..AA....C.--------...-.......CTCT.........AA....C........T  238
Query_59663  79   -....TC..C.....T..T..G...--..---..TTT..A......T...CT.T..AC--AA--C...AAAACCC---...T-.......----...CT.....A............TA  180
Query_59664  130  ...TTTC..T.....TA..A.G...--..GTT..---..A..G...T...AT.....A--.A--..G.CA--------A..AT.......----...CA..T.CA.......A...T.  228
Query_59664  135                                                                                                          ...........     145
Query_59665  127  ....TTC.TT.....TA..A.G...--..GTT..---..A..G...T....T.....A--.A--..G.CA--------A..-.......----...CA..T.CA.......A.C..A  224
Query_59666  34             .....T..G..G...TGT.T---..---..A.........C-.....A--..--....ACA-------...T-....C..----...CA.....A..........GA.-  119
Query_59667  37   .....TC.AT...G.T...T.G..C.--..---..TTT..AG.........CT..-..A--.A--....CAACA-----....-.....G.----...CACA......C.......G..-  135
Query_59668  127  -....TC..C.....T..T..G...--..---..TTT..A..........CT.T...A--AA--....AAAAAAAACC...T-.......----...CA.....A............T.  231
Query_59669  114  AC.......................--..---..---.......................--..--....--------...-.......----.................G......  208

Query        214  CCTCATTAATTCCCTAGAAAGAACTTCTGGAAGTCAAATTACTAGGTCACAGGGGATGAAAAGGT-CTCATGACTTGCTTGGATTAGTAAATTTTACTCCTTGTCAGGAGGTTCAGC  329
Query_59657  225  .........................G........TG......G.....A..A....-..G....C...G...T...G..ATG...C.G.G....GT...AA......            331
Query_59658  109  ........G..........................C.GCG...G.....A..C..G.-.G...TG-...G...T.......                                      187
Query_59659  227  .........................G........TG......G.....A..A....-.......C.......                                               298
Query_59660  225  .........................G........TG......G.....A.......-.......C...G...T...                                          300
Query_59661  225  .........................G........TG......G.....A.......-.......C...G...T...G..AT....C.G.G....GT...AA......            331
Query_59662  239  T...G.............................GCCG..G.....A......CA...G...-.C.....TC.C...........G....C..-...-CC.........C....     352
Query_59663  181  .............................C.....C.......G.....A........C.TG.TC-...G...T.......T...CC.G.......T...                  279
Query_59664  229  ..............CG.........G.........G.......G.....A.......-.......C.......                                              300
Query_59665  225  .........................G........TG......C......A.......-.......C.A.G...T...G..AT....C.G.G....GT...AA......            331
Query_59666  120  -........................G........TG.......G.....A....A...C....TC-..G..........C.T...CC.G.....                        211
Query_59667  136  -........................CGG.....A.GG.......G.....AT.......C..G..C-.C.G.........G.T...CC.G...G...T...AA.C....C..C.....  250
Query_59668  232  .............................C.....C.G..C..G.....A........C..G.TC-...G...T.......T...CC.G.......T...CA.C.G..T..C.....  347
Query_59669  209  ...............................................A....A......G...-.......T.....................TC.............C.....  324
```

ECR16

```
Query         2    CTATGCCCATCACCACTATCTCTGAGGGAGATTTTGCAACTCTGTGG-CC-TTAGCAACAGAGGCAGGCCCAGACCATCACCATGGAAACTGCATCA------------G----------  97
Query_163879  48                                               ..C-..-............G....T.....T..................G.T------------.----------  99
Query_163880  48                                               ..C-..-...........AG....T.........................G.C------------.----------  99
Query_163881  51                                                       ...........G....T.....T..................GTT------------A----------  96
Query_163882  48                                               ..C-..-............G....T.....T..................G.T------------.----------  99
Query_163883  48                                               ..C-..-............G....T.....T..................G.T------------.----------  99
Query_163884  1               .........TG....A......A.....C.....C-..-...........TT..............................------------.----------  85
Query_163885  48                                               ..C-..-...........AG....T.........................G.CACGAGGAGAAGG.----------  111
Query_163886  101                                           ......-..C............G....T...G.TG..G.............                           152
Query_163887  48                                               ..C-..-............G....T.....T..................G.T------------.----------  99
Query_163888  20                                               ..C-..-............G....T.....TG.................G.T------------.----------  71
Query_163890  51                                                 .-..-............G....T.....T..............CA..G.C------------A----------  100
Query_163891  101                                                  ..-............G....T........G.............                           145
Query_163892  51                                             .....T..-.................A...............G.......G.T------------.AGGGCA----  111
Query_163893  1    ..........A.......................A..........C-..-......................................T...------------.----------  96
Query_163894  51                                                 .-..-............G....T.....T..............CA..G.C------------A----------  100
Query_163895  45                                     ....GA....---..-.....C......AG....T....................C...G.C------------.AGAGGGAAGG  114

Query         98   -GA-CTGCA-TA---A--AG--ATGGG-----------A---------GG---AG------------------CT----------------------G---------------------  122
Query_163879  100  -..-GG..G-G.---.--..--G...-----------G---------.---.------------------..GAGGAAGGGGCCGGGAGGGCCCC.---------------------  147
Query_163880  100  -AG-G..G.-G.---.GGG.--.G..-----------.---------.---..GAAGGGGCGTGTGGAGCC..----------------------.---------------------  144
Query_163881  97   -..-GG..G-G.---.--..--G.T..-----------G---------.---.------------------..----------------------.---------------------  121
Query_163882  100  -..-GG...-G.---.--..--G..A.-----------G---------.---.------------------..GAGGAAGGGGCTGGGAGGGCCCC.---------------------  147
Query_163883  100  -..-GG...-G.---.--..--G..A.-----------G---------.---.------------------..GAGGAAGGGGCTGGGAGGGCCCC.---------------------  147
Query_163884  86   -..-.C...-..---G--..--G...------------.---------.---.------------------..----------------------.---------------------  110
Query_163885  112  -..-GG.GC-.G---.--G.--.A..-----------GCCCGG----.---.------------------.CCA---------------------.---------------------  143
Query_163887  100  -..-GG..G-G.---.--..--G...-----------G---------.---.------------------..GAGGAAGGGGCTGGGAGGGCCCC.---------------------  147
Query_163888  72   -..-GG..G-G.---.--..--G...-----------G---------.---.------------------..----------------------.AGGAAGGGGCCGGGAGGGCCC  117
Query_163890  101  -..-G...G-G.---.--..--G....GGAGCTGAGGA.---------.---G.------------------.C----------------------.---------------------  136
Query_163892  112  -..G...GGG..GCT.--..--.G..-----------C---------T.CAG..------------------..CT---------------------.---------------------  146
Query_163893  97   -..-.C...-..---.--..--G...------------.---------.---.------------------.C----------------------A---------------------  121
Query_163894  101  -..-A...C-G.---.--..GT.G...------------.GCTGAGGAG.---G.------------------..----------------------.---------------------  136
Query_163895  115  G..-GG.GC-.G---.--G.--.A..-----------GCCCGG----.---..AC----------------.A----------------------.---------------------  147

Query         123  --GG----------------------AACTGGAATTTTCCATTTTTTGGATTCAGTTCTAAACCATG-A-C-TGGCACACAG-TGGTCCTTCATTAAATGTTTATTGAGAAAACAAATA  213
Query_163879  148  --.A----------------------..............G.C......G.TG.....T.......-.C.-.CTG......-.A.A...GT..G..GCA........A.G..T...G.  239
Query_163880  145  --CA----------------------..............GAC......G..C...ACG....C..-.C.-..........-..TG.....C...G..                       216
Query_163881  122  --A.GAAGGGGCTGGGAGGGCCCCGAA..............G.C......G..G.....T......T-.C.-..T.......-.A.A.T.GT..G..GC.........A.G..T...   234
Query_163882  148  --.A----------------------..............G.C........-G.....T.......-.C.-.CT.......-.A.A...GT..G..GC.........A.G..T...   236
Query_163883  148  --.A----------------------..............G.C........-G.....T.......-.C.-.CT.......-.A.A...GT..G..GC.........A.G..T...   236
Query_163884  111  --.T----------------------..............G.C..........A............-.-.-..A.......-...C.....G...G....C..........       195
Query_163885  144  --C.----------------------..............G.C......G..C...G.G....C..-.-.G..........-..TG.....C......A...G....A.T..TG...G  235
Query_163887  148  --.A----------------------...C..........G.C......G.TG.....T.......-.C.-.CT.......-.A.A....T..G..GCA........A.G..T...   237
Query_163888  118  CA.A----------------------..............G.C......G.TG.....T.......-.C.-.CT.......-.A.A...GT..G..GC.........          200
Query_163889  98                                                                    ..-C-.-.......T.A-.A..GA..................          136
Query_163889  90                                                                              .......-....                          80
Query_163890  137  --..TGGGCCCCAGA-----------..............G.C......G..G.....T.......TC-.-..T.......-.A.A...GT.GG..GCA........          228
Query_163892  147  --..----------------------..............G.C....C.G.......G.......-.C.-..........CG.......                             211
Query_163893  122  --..----------------------..............C.C.......................-.-.-A.........-............................TG...G  212
Query_163894  137  --..AGGGCCCCAGA-----------.............GG.C..G...G..G.....T.......TC-.-..T.......-.A.A...GT..G..GCA........A.G..T...   237
Query_163895  148  --.A----------------------..............GG.......G..G...A.....TTGC-C-.-..........-...A.....C......A........         227

Query         214  CCTGAAACCTAACGCAGACAGCAGGGGC  241
Query_163879  240  A....                         244
Query_163885  236  G.....                        241
Query_163893  213  A.......A...T...T...........  240
```

ECR17

```
Query        118  CACACTA-GAGCCAGCTGTGTACTGA--GCTCC--TC-A-CAAACACATAGGACAGGAATGCATTGTGTCAGACAAGGGAGAGAAAAGGCAGCCAGCCCCAGGAAATTAAAGAAAAGCTG  230
Query_85541  105  ....TA.-............CG...C--A.C..--.TT.-...G..TG...........T...A.........T.AGA.......A.............................  218
Query_85542  131  .C...G.-.............G....--A.C..C-.T-G-..GG...GC........CG.CTG..C........GC...-...G....A.................C.-GG...GCA.G.  242
Query_85543  8    ....TA.-............CG...C--A.C..--.T-TG...G..TGC..........C....A...G......T.AG.....G..A.......T....................T..  121
Query_85544  59   ....TAG-............CG...C--A.C..--.TT.-...G..TG...........T...A.........T.AG.......A.............................  172
Query_85545  104  ....TA.-............CG...C--A.C..--.TT.-...G..TG...........T...A.........T.AG.......A.............................  217
Query_85546  92   .......A..........G..C.CA.--..C..--..-.-...G..................................G......T-............................  204
Query_85547  118  ....TA.-............C....C--A.C..--.TT.-...G..TG...........T...A.........T.AG.......A.............................  231
Query_85548  105  ....TA.-............CG...C--A.C..--.TT.-...G..TG...........T...A.........T.AGA.......A.............A................  218
Query_85549  86   .G.CGG.-.....G.......G...G--A....CT..-C-.CG.GG.--...C.......C.GC.A.A..G...C.T.AG........A..........G....CG...G......T...  198
Query_85550  118  .C..TA.-.G..........CG...C--..C.A--.TT.-.G.G..TG...........T...A.........T.AG...C...............T........G...G.......  231
Query_85551  11   .C..TG.-A......T....CG....--C.C..--.T-T-..GG..TG..AA.....G..CAG..A........CGT.AG.....G.A.....G.T...........CGG.GGG.A...  123
Query_85552  119  .....A.-............C..G.G--..C..--CG-TG...G..TGC..........CT...CA.........G..AG...G.G..............................  232
Query_85553  119  ....TA.-............CT...C--A.C..--.TT.-...G..TG...........T...A.........T.AG.......A.............................  232
Query_85554  131  TG.G.AG-.G..........G....--A.C..C-..-.-..GG...G............C.G..A......G.C.T.AG........A....TG..............GG.G...A...  244
Query_85555  59   TCT..G.-.....T.......G....--..C..C-.G-.-.G.G..TG...........TT...A............AGC...............G..-.......G........A...  171
Query_85556  118  .......-......A...........--..C..--..-.-............................................................................  230
Query_85557  118  ....TA.-............CG...C--A.C..--.T-TG...G..TGC..........T...A...G......T.AG.....G..A.......T....................T..  231
Query_85558  129  .C..TA.-..........C.C....C--A.C.A--.TT.-...G..TG...........T...A.........T.AG.....--................................  240
Query_85559  118  .....C.-..............G....AT..-..--..-.-..GG.GTGCC..........T...A........C...AG........A.............A.......G.C....A...  231

Query        231  GCTATC----GGCTGCATCTAGGGACACGGCGTGCCGA-----GCAG----ACAGCTG----------------C-----------------GGTT---------TCA------------  283
Query_85541  219  ......----AA.A..........G...AATC...AA.-----...----.......----------------.-----------------AA..---------.G.------------  271
Query_85542  243  ...G..----.CTG.............TCT.-...A..-----...----.......----------------.                                              287
Query_85543  122  ...C..----C..A.............TAATC...AA.-----...----.......----------------.AAGTTAAATGTTTGGAT....CAGAAATGGA..------------  200
Query_85544  173  ......----AA.A..............AATC...AA.-----...----.......----------------.-----------------AA..---------.G.------------  225
Query_85545  218  ......----AA.A..............AATC...AA.-----...----.......----------------.-----------------AA..---------.G.------------  270
Query_85546  205  ......----T................T.........-----A...----.......----------------.-----------------.C..---------..G------------  257
Query_85547  232  ......----A..A..............AATC...AAC-----...----.......----------------.AATTGAATGTTTGGAT-....CAGAAATGGA..------------  309
Query_85548  219  ......----AA.A..............AATC...AA.-----...----.......----------------.-----------------AA..---------.G.------------  271
Query_85549  199  ..C...-----.ATG.A..........TTAATC...A..CAGCT....TTAA.T.TT..GA--------------.-----------------...C---------.G.------------  262
Query_85550  232  ..C...-----...A............GT.ATC...AA.-----...----.......----------------.-----------------.A.C---------.A.------------  284
Query_85551  124  ......-----...G.TG...G.....GTAA.C...AA.-----...----.......----------------.-----------------.A..---------AA.------------  176
Query_85552  233  ......----A..A...C........G.-C..C..AA.-----..T.----.......----------------.                                              277
Query_85553  233  ......----A..A..............AATC...AA.-----...----.......----------------.-----------------AA..---------.G.------------  285
Query_85554  245  ......GACA.A.A..C...G......TAATC..-..G-----..G.----.......----------------.                                              293
Query_85555  172  ...G..-----...A............C.C.T....A.-----...----.......----------------.                                              216
Query_85556  231  ......-----..T.......................-----...----.......----------------T-----------------...---------..------------  283
Query_85557  232  ...C..----C..A.............TAATC...AA.-----...----.......----------------.AAGTTAAATGTTTGGAT....CAGAAATGGA..------------  310
Query_85558  241  ..C...-----...A............TAATCG..AA.-----...----.......----------------.-----------------.A..----------...GTGTTTGCATGG  305
Query_85559  232  ..C...-----.A.G.A....G......TA.TC.-----------...----......CAATTAAATGTTTGGA.-----------------..C.---------.G.------------  295

Query        284  -----------------A-----------------------------TA-------CT---CT-----GCTGGCCATGGGGAGCTGGTTCCAAGAAGCTGCTGACCCCCTGAGTCCTGTG  342
Query_85541  272  -----------------.TGTTTGGATGGTTCAGAAATGGACAGTTC.G-------.A---.-----..........AT......T.......-....A..CC.TT.......TTCC..  358
Query_85543  201  -----------------G-----------------------------.T-------..GCA..-----..........AT......T.......-....A..CC.TT.G.....TGCT..  261
Query_85544  226  -----------------.TGTTTGGATGGTTCAGAAATGGACAGTTC.G-------.A---.-----..........AT......T.......-....A..CC.TT.......TGCC..  312
Query_85545  271  -----------------.TGTTTGGATGGTTCAGAAATGGACAGTTC.G-------.A---.-----..........AT......T.......-....A..CC.TT.......TGCC..  357
Query_85546  258  -----------------.-----------------------------.G-------..---T.-----.......TCCAT....C.T...........C...A................  316
Query_85547  310  -----------------G-----------------------------.T-------..GCA..-----..........AT......T.......-..A.A..CC.TT.......TGCC..  370
Query_85548  272  -----------------.TGTTTGGATGGCTCAGAAATGGACAGTTC.G-------.A---.-----..........AT......T.......-....A..CC.TT.......TTCT..  358
Query_85549  263  -----------------.AAGGGAC----------------------A.-------..---..GCATC.......G..CT.G.T..T.C.............T..T.TG...CT.....  333
Query_85550  285  -----------------.-----------------------------.                                                                         286
Query_85550  319                                                              ..-----.....T.G..AT......T........-...A..CC.TT.......TGCT..  371
Query_85551  177  -----------------.-----------------------------..                                                                        179
Query_85551  31                                                                                                       ...........            41
Query_85553  286  -----------------.TGTTTGCATGGTTCAGAAATGGACAGTTC.G-------.A---.-----..........AT......T.......-..T.A..CC.TT.......TGCC..  372
Query_85556  284  -----------------.-----------------------------.G-------..---.-----.........C.T...T..C................................  342
Query_85557  311  -----------------G-----------------------------.T-------..GCA..-----..........AT......T.......-....A..CC..T.G.....TGCT..  371
Query_85558  306  TTCAGAAATGGACAGTTC-----------------------------.G-------.G---.-----..........AT......T.......-....A..CC.TT.......TGCT..  380
Query_85559  296  -----------------.-----------------------------A.TGGACAG..---..GCCTC..........CT.G.T..T.C..........A.ACT.T.........T.....  366

Query        343  GAAATTCAGTTACTTCCTGGACCTCAGGTGAACCTTTAGCTCACCTCCCATAGGATTCCACCCACAGTGAGTCCTGGGAG  422
Query_85541  359  ..G..G..TA..T..............C......                                                392
Query_85543  262  ..G..G..TG..T..............C......                                                295
Query_85544  313  ..G..G..TA..T..............C......                                                346
Query_85545  358  ..G..G..TA..T..............C......                                                391
Query_85546  317  ....GG...C..G.......................G................G.........G..............  396
Query_85547  371  ..G.GG..TA..T..............C......                                                404
Query_85548  359  ..G..G..TA..T..............C......                                                392
Query_85549  334  .G..CG.GTA..T............T.C..GG...........                                       376
Query_85550  372  ..GT.G..TA..T..............C......                                                405
Query_85553  373  ..G..G..TA..T..............C......                                                406
Query_85556  343  .....G.......................................................................  421
Query_85557  372  ..G..G..TG..T..............C......                                                405
Query_85558  381  ..G..G..TA..T..............C......                                                414
Query_85559  367  .G..CG..CA..T............T.CC.G..T.........                                       409
```

ECR18

```
Query         1    TGCTGCCTGCCTGCCAGCAGAAGCCCCG-GGCGGGTCAGAAGCA--GTTTGACA-TACCGCAGCGGCCAACACAGCTGTTGAGGGGGGATTAGCTGCAGGGAAAGCCGATCCAGTGACAA  116
Query_245359  98                               ..A..-...T..........--..C.....-GGT.A...A.-...G.C........--............T.A.......T.....TA.....C  187
Query_245360  98                               ..A..-...T..........--..C.....-GGT.A...A.-...G.C........--............T.A.............TA......  187
Query_245361  98                               ..A..-...T..........--..C.....-GGT.A...A.-...G.C........--............T.A.......T.....TA.....C  187
Query_245362  43                             .....T.A...T.......G...--..C.....-.G......-...TGG.T......C..---.A..........T.AA......T.....TA......  134
Query_245363  93                                                                    .....GAG.......C.--............TCA............CA......  146
Query_245364  98                               ..A..-...T..........--..C.....-GGT.A...A.-...G.C........--............T.A.......T.....TA.....C  187
Query_245365  101                                                                                                                 ....A.....  110
Query_245366  109                                                                                       ............TTA......G......T.....G.  145
Query_245367  18                               ..A..-T..T......GG...--.C.....G-.G..A...-.A...GTG.......C.---...........T.A.......A.A....CA......  106
Query_245368  98                               ..A..-...T..........--..C.....-GGT.A...A.-...G.C........--............T.A.......T.....TA......  187
Query_245369  98                               ..A..-...T..........--..C.....-GGT.A...A.-...G.C........--............T.A.......T.....TA......  187
Query_245370  59                                    .-...T....G......TG..C...TG-GG..A.T.-.....G.C........-..............T.A.........C...CA.....C  147
Query_245371  98                               ..A..-...T..........--..C.....-GGT.A...A.-...G.C........-.............T.A.......T.....TA......  187
Query_245372  101                                  ..-...T.......G..--.........-.G..A...-....G-.G......CCAT....--.........CA.......T.....GA......  186
Query_245373  101  ..........T..........G.....A-..........G...--.........-.......................................T.......A.T.............  216
Query_245374  57                               ..A..-...T...........--T.C.....-GGT.A...A.-...G.C........--............T.A.......T.....TA......  146
Query_245375  62                                   ..-T....C......T..--.CC..CG.G.....G---.....TG-.......C.---..........T.A.......G......C.....G.  145
Query_245376  59                                    .-...T..........CA..C...T.-GG..ATG.-.....G.C........--............T.A.......T.....CA......  146
Query_245377  21                                    .-...T.........A..--.CCCA...-CG.TA...-.....G.C........--...A........ATGA.......T.....TC......  106

Query         117  ATGGTTTAGGTATGTCCGCCCCTGGGAA--ATGTGATCTCCACGGTTTGCACTATAGAAACTTGTTTGTTTCAAAAGAT-GAGGA-ACATGTTTGTTGTCTGTGTCTCCAGAGAAATCAC  232
Query_245359  188  ...C.......................--.C....C..T..A.......GT.......CT..A..C.C..........-.GA..-...CT......T....A...C.TG...-.....T  302
Query_245360  188  .C.C.C.....................--.C....C..T.GA........T.......CT.C...C.C..........-.GA..-...CT......T....A..G...G...-.....T  302
Query_245361  188  ...C.......................--.C....C..T..A.......GT.......CT..A..C.C..........-.GA..-...CT......C....A...C.TG...-.....T  302
Query_245362  135  ........AA.....G.A.........--......C..T..A........T.....-..T..C....C..T.GG.....-.GA..-...C.......T....A..-..T..GA.....T.  248
Query_245363  147  .C.T..C...................G--GC.C..G.....A...C..T.......-.....C.C.G..G..GG....C-.GA..-...........T....A....CT.....  254
Query_245364  188  ...C.............A.........--.C....C..T..A.......GT.......CT..A..C.C..........-.GA..-...CT......C....A...C.TG...-.....T  302
Query_245365  111  ...........................--.C.......T..A......AG.......-..................-......-...C...........................  225
Query_245366  146  ....G.C.....................  173
Query_245367  107  .C.T..C....................GC.....--..TG.A.........T.....-......A.G..A.....G...-AGA..-............T....A...C..G...-.G.TG.  220
Query_245368  188  ...C.......................--.C....C..T..A.........T.......CT.....C.C..........-.GA..-...CT......TG.C.A...C..G...-.....T  302
Query_245369  188  ...C.......................--.C....C..T..A.......TT.......CT.....C.C..........-.GA..-...CT......T....A...C.TG...-.....T  302
Query_245370  148  ...CC..C................A..--.C....C..T.CA........T..C....CTCC...C.C.........G.-ACA..-...CT......T..C.A...-..C.G.GT.G...  262
Query_245371  188  ...C.......................--.C....C..T..A.........T.......CT.......C..........-.GA..-...CT......T....A...C..G...-.....T  302
Query_245372  187  ....CCA.A..................--.C....C..T..A.........T.....-..........C....G.....-.GA..-..GCC.G.....-..G.A...C.TG...-..G...  299
Query_245373  217  ...........................--.C..................................................-......-...................................  332
Query_245374  147  .C.C.C.....................--.C....C..T.GA........T.......CT.C...C.C..........-.GA..-...CT......T....A..GC.TG...-.....T  261
Query_245375  146  ....G.C.....................  173
Query_245376  147  ...C....................A..--.C....C..T.GA........T.......CT.......C..........-AGA..-...CT......T....A...-..C.G.GT....T  261
Query_245377  107  ...........................--......C..T.GA........T.G.-CC......C.G.C..........G.....C..T.T......TC...A...C..G...-.....G.  222

Query         233  AACGTTGACAG--CTCTGGATATACCCAAAGAGACAGAGTACaaaaaaaCCAATTACACGTAGGCTGCGCTCATAATCAACAAATGCTGGTTTGGGAATTGCTGTGTGAAGATTCCTCTC  350
Query_245359  303  G.T.CA.....--..T....GG..........A.TG..AC..-.G..TC.........TA.....C..A.A...G..A.....C..................G.C....C.....T..  419
Query_245360  303  G.T.CA....A--..T....GG..........A..G..AC..-.G..CC.........T......C..A.A...G..A.....C...A...............G.C....C.....T..  419
Query_245361  303  G.T.CA.....--..T....GG..........A..G..AC..-.G..TC.........TA.....C..A.A...G..A.....C..................G.C....C.....T..  419
Query_245362  249  ..T.CA....A--..T.A..C....A...CA.A.....A.G.-....TG.........T.........AATA.....CA....GCCA.................C......C..-.....-  363
Query_245364  303  G.T.CA.....--..T....GG..........A..G..AC..-.G..TC.........TA.....C..A.A...G..A.....C..................G.C....C.....T..  419
Query_245365  337                                               .................A..................C.......................C.................  414
Query_245365  226  ..T........AT..T...........--....................  272
Query_245367  221  G...GA....T--..T..A.CG......G...A.....  256
Query_245368  303  G.T.CA.....--..T....GG..........A.TG..AC..-.G..TC...............C..A.A...G..A.....C...........A........G.C....C.....T..  419
Query_245369  303  G.T.CA.....--..T....GG..........A..G..AC...G..TC.-........TA.....C..A.A...G..A.....C..................G.C....C.....T..  419
Query_245370  263  G.T.AAC...A--..T....GG..........A..  295
Query_245371  303  G.T.CA..G..--..T....GG..........A..G..AC..-.G..TC...............C..A.A...G..A.....C..................G.C....C.....T..  419
Query_245372  300  ..T.GA....A--..T.....G.....GG...A.....A...-....T..........T.-.......  363
Query_245373  333  ...........--.........................G.............................................................................  450
Query_245374  262  G.T.CA....A--..T....GG..........A..G..AC..-.G..TC.........T......C..A.A...G..A.....C...A...............G.C....C.....T..  378
Query_245376  262  G.T.AA....A--T.T....GGG.....G...A.TG..AC..-.G..TC...G.......A.........A.A..CG..A.....C..................A.CTC..C.....TCG  378
Query_245377  223  ..T.GA....A--..T....C..GT.......  252

Query         351  GGCAGCCTGCTCAGAATGTACGTCTGGTGACTTCTGAGATGTCAC  395
Query_245359  420  .....TT..........A..TTG...A...T..........      460
Query_245360  420  .....TT.....G....A..TTG...A...T.......G..      460
Query_245361  420  .....TT..........A..TTG...A...T..........      460
Query_245362  364  ------T....TG.......G.G.CA....T.C.........T..  402
Query_245364  420  .....TT..........A..TTG...A...T..........      460
Query_245365  415  ................................CC.........  459
Query_245368  420  .....TT.A........A..TTG...A...T.......G..      460
Query_245369  420  .....TT..........A..TTG...A...T..........      460
Query_245371  420  .....TT..........A..TTG...A...T..........      460
Query_245373  451  ......................................C......  495
Query_245374  379  .....TT..........A...TG...A...T.......G..      419
Query_245376  379  .....TT..............TG...A...T..........      419
```
