# Supplementary material for: Phylogenetic and Epigenetic Footprinting of the Putative Enhancers of the Peg3 Domain
Source: PLoS One. 2016 Apr 22;11(4):e0154216. doi: 10.1371/journal.pone.0154216 (PMC4841594; doi:10.1371/journal.pone.0154216)
Supplement: S4 File — (PPTX) [file pone.0154216.s004.pptx]

## Slide 1
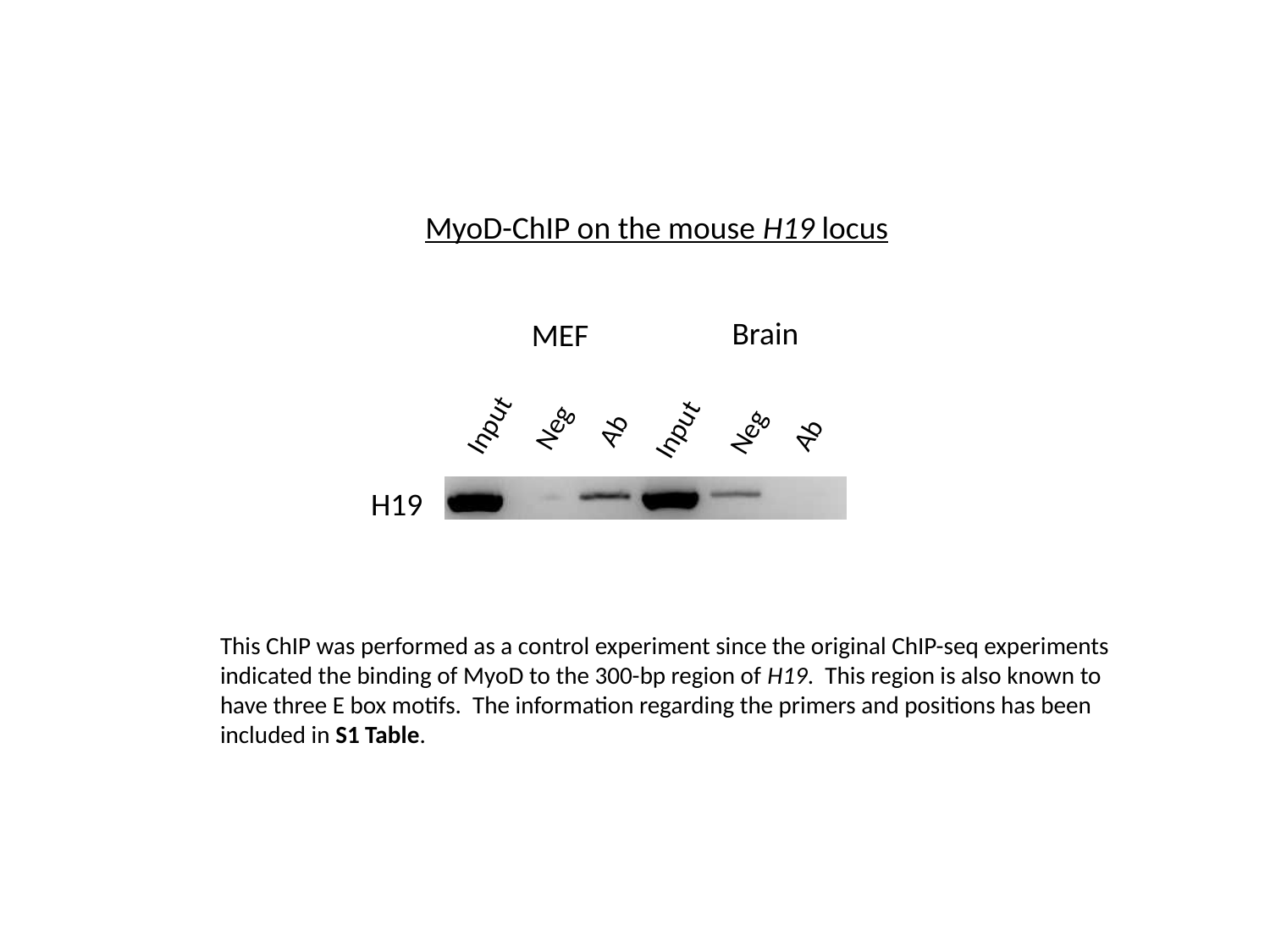

MyoD-ChIP on the mouse H19 locus
Brain
MEF
Ab
Neg
Input
Ab
Neg
Input
H19
This ChIP was performed as a control experiment since the original ChIP-seq experiments
indicated the binding of MyoD to the 300-bp region of H19. This region is also known to
have three E box motifs. The information regarding the primers and positions has been
included in S1 Table.
